# DHODH Blockade Induces Ferroptosis in Neuroblastoma by Modulating the Mevalonate Pathway

**DOI:** 10.1016/j.mcpro.2025.101014

**Published:** 2025-06-11

**Authors:** Jui-Chia Shir, Pin-Yu Chen, Chuan-Hao Kuo, Chiao-Hui Hsieh, Hsin-Yi Chang, Hong-Chih Lee, Chen-Hao Huang, Chun-Hua Hsu, Wen-Ming Hsu, Hsuan-Cheng Huang, Hsueh-Fen Juan

**Affiliations:** 1 Institute of Molecular and Cellular Biology, National Taiwan University, Taipei, Taiwan; 2 Department of Life Science, National Taiwan University, Taipei, Taiwan; 3 Graduate Institute of Medical Science, National Defense Medical Center, Taipei, Taiwan; 4 Graduate Institute of Biomedical Electronics and Bioinformatics, National Taiwan University, Taipei, Taiwan; 5 Department of Agricultural Chemistry, National Taiwan University, Taipei, Taiwan; 6 Department of Surgery, National Taiwan University Hospital and National Taiwan University College of Medicine, Taipei, Taiwan; 7 Institute of Biomedical Informatics, National Yang Ming Chiao Tung University, Taipei, Taiwan; 8 Center for Computational and Systems Biology, National Taiwan University, Taipei, Taiwan; 9 Center for Advanced Computing and Imaging in Biomedicine, National Taiwan University, Taipei, Taiwan

**Keywords:** proteomics, DHODH, neuroblastoma, lipid metabolism, ferroptosis, mevalonate pathway

## Abstract

Neuroblastoma is the most common heterogeneous solid tumor in children, and current treatment options remain limited, especially for high-risk patients. Previous studies have identified dihydroorotate dehydrogenase (DHODH), a key enzyme in pyrimidine synthesis, as a potential therapeutic target in cancer. However, none of the existing FDA-approved DHODH inhibitors have shown effective inhibition of neuroblastoma cell growth. To address this challenge, we employed virtual screening to discover potential DHODH-targeting drugs, identifying Regorafenib as a promising candidate. Regorafenib significantly inhibited neuroblastoma growth in both neuroblastoma cells and patient-derived organoids. To unravel the underlying molecular mechanisms, we conducted Tandem Mass Tag (TMT)-based quantitative proteomics using LC-MS/MS. Our proteomic profiling revealed substantial regulation of lipid metabolism proteins, specifically those in the mevalonate pathway, correlating with ferroptosis induction. Further analysis showed that DHODH inhibition led to a reduction in total cholesterol, cholesterol esters, disrupted lipid droplet formation, and significantly decreased the expression of Squalene Epoxidase (SQLE), a key enzyme in lipid metabolism. Notably, we also observed an increase in nuclear SQLE expression following DHODH inhibition. In summary, our study highlights DHODH blockade as a novel approach to induce ferroptosis through lipid metabolism reprogramming, underscoring DHODH as a viable therapeutic target for neuroblastoma treatment. These insights open new avenues for metabolism-based interventions in aggressive pediatric cancers.

## Highlights

• TMT proteomics shows DHODH inhibition rewires lipid metabolism in neuroblastoma.
• DHODH blockade reduces cholesterol levels, impairing lipid droplets and energy flux.
• FDA-approved regorafenib reduces neuroblastoma growth in cells and patient organoids.
• Regorafenib-mediated DHODH blockade triggers ferroptosis via mevalonate pathway.

## Graphical Abstract

**Figure:**
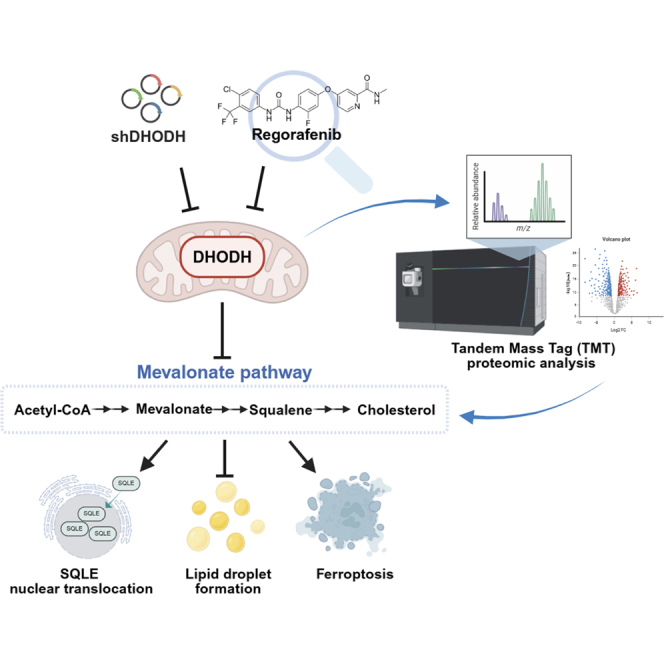

## In Brief

Quantitative TMT-based proteomic analysis revealed that DHODH inhibition by Regorafenib suppresses the mevalonate pathway, disrupts lipid droplet formation, and induces nuclear translocation of SQLE in neuroblastoma cells. These findings uncover a lipid metabolism–linked mechanism of ferroptosis, highlighting DHODH as a promising therapeutic target for this pediatric cancer.

Neuroblastoma is a heterogeneous solid tumor that primarily arises in the sympathetic nervous system and predominantly affects children under the age of 5 (1, 2, 3). While it is rare in adults, neuroblastoma constitutes 5% to 10% of all childhood cancers and is responsible for approximately 15% of pediatric cancer deaths (4). This tumor typically originates in the adrenal glands but can also develop in areas like the neck, chest, abdomen, or spinal cord (5). Survival rates are relatively high for patients with low-risk and intermediate-risk neuroblastoma, but for those with high-risk neuroblastoma, the 5-year survival rate is less than 40% (4, 5, 6). Neuroblastoma is classified into stages based on factors such as the degree of differentiation, patient age, and MYCN oncogene status, with MYCN amplification serving as a critical prognostic marker of risk (6). While low-risk neuroblastoma can spontaneously regress or differentiate into benign tumors, high-risk cases are prone to relapse despite intensive treatment, leading to poor prognosis and high mortality rates (7). Current treatment options, including surgery, radiation therapy, and chemotherapy, are not equally effective for all patients, and there is currently no definitive cure for high-risk neuroblastoma. This underscores the urgent need for novel therapeutic strategies to improve outcomes for these patients.

Mitochondria plays a central role in cellular metabolism, serving as the powerhouse of cells by generating ATP through oxidative phosphorylation. They produce energy by breaking down glucose and fatty acids *via* pathways such as the citric acid cycle, thereby synthesizing ATP (8, 9). In addition to energy production, mitochondria generate metabolites required for the biosynthesis of amino acids, fatty acids, and nucleotides (10, 11, 12). They also maintain cellular redox homeostasis by regulating reactive oxygen species (ROS), which play key roles in cellular signaling and defense mechanisms (13, 14). Cancer cells often reprogram their metabolism to support rapid growth, favoring glycolysis over oxidative phosphorylation even in the presence of oxygen, a phenomenon known as the Warburg effect (15). Mitochondrial ROS are closely linked to cancer progression, low levels of ROS can promote cell proliferation, while high levels can cause DNA damage and mutations (16). Cancer cells also enhance their antioxidant systems to manage ROS and evade apoptosis. Since mitochondria are pivotal in controlling apoptosis, many cancer cells alter mitochondrial functions or signaling pathways to escape cell death, thereby increasing their survival (17). These metabolic adaptations present potential therapeutic targets and have led to the development of drugs designed to disrupt mitochondrial function, inhibit energy production, or induce apoptosis in cancer cells.

Dihydroorotate dehydrogenase (DHODH) is a flavin-dependent mitochondrial enzyme that plays a critical role in the *de novo* synthesis of pyrimidines by catalyzing the production of uridine monophosphate (UMP), which is essential for nucleic acid biosynthesis in rapidly proliferating cells (18, 19, 20). Located in the inner mitochondrial membrane, DHODH consists of 395 amino acids and has a structure designed to facilitate redox reactions (21). Abnormalities in pyrimidine metabolism, which are closely linked to uncontrolled cell proliferation, make DHODH a key contributor to cancer development, particularly in high-risk neuroblastoma, where it is associated with MYCN status (22, 23). Despite its established role in cancer, the precise mechanisms by which DHODH influences neuroblastoma remain unclear. Nonetheless, its involvement in tumor growth has made DHODH an attractive therapeutic target, prompting the development of various DHODH inhibitors. These inhibitors typically bind to the N-terminal site of DHODH, blocking its interaction with ubiquinone (CoQ10) and hindering pyrimidine synthesis (21, 24). Although many DHODH inhibitors have been explored, only a few, such as Leflunomide and its active metabolite Teriflunomide, have received FDA approval for clinical use (25). These drugs, while effective in certain contexts, have limitations such as hepatotoxicity, teratogenicity, and insufficient efficacy in cancer treatment (24). Other inhibitors, like Brequinar, have shown promise but failed in clinical trials due to challenges with dosage and administration (24). Despite ongoing research to develop more potent and safer DHODH inhibitors, there remains an urgent need for new therapeutics, particularly for cancer treatment. Given DHODH’s pivotal role in neuroblastoma, identifying effective DHODH-targeting drugs for this disease is of high priority.

**Fig. 1:**
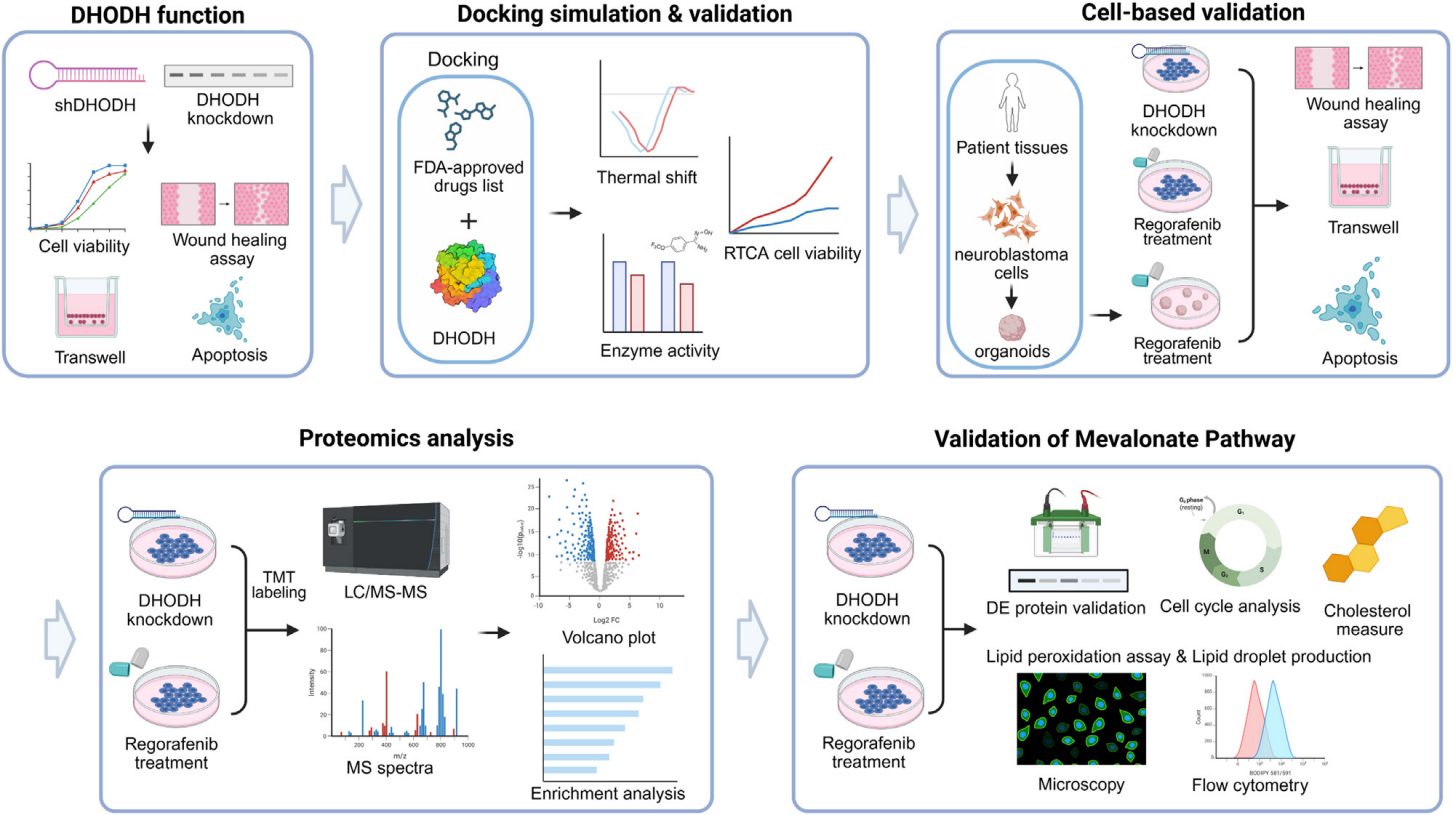
**Overview of the experimental workflow and methodologies used in this study**. We validated DHODH as a potential therapeutic target for neuroblastoma using clinical data and cell-based DHODH silencing experiments. Docking simulations were then employed to identify and evaluate FDA-approved drugs capable of inhibiting DHODH activity in high-risk neuroblastoma. The efficacy of the candidate drug was confirmed through both cell-based assays and patient-derived organoid models. To further explore the molecular mechanisms underlying DHODH inhibition in neuroblastoma, we conducted Tandem Mass Tag (TMT) proteomic analysis to profile changes in the proteome following DHODH inhibition. Our results revealed a novel mechanism involving lipid metabolism and ferroptosis linked to DHODH inhibition, providing new insights into potential therapeutic strategies for treating high-risk neuroblastoma.

In this study (Fig. 1), we utilized clinical data and silencing cell-based experiments to establish that DHODH is a potential therapeutic target for neuroblastoma. We then performed docking simulations to identify and evaluate potential FDA-approved drugs capable of inhibiting DHODH activity in high-risk neuroblastoma, confirming their efficacy through both cell-based assays and patient-derived organoid models. To gain deeper insights into the molecular mechanisms by which DHODH inhibition impacts neuroblastoma, we conducted Tandem Mass Tag (TMT) proteomic analysis to profile proteome changes following DHODH inhibition. Our findings revealed a novel mechanism involving lipid metabolism and ferroptosis linked with DHODH inhibition. These results provide new perspectives on the relationship between DHODH and lipid metabolism and could contribute to the development of innovative cancer treatment strategies.

## Experimental Procedures

### Experimental Design and Statistical Rationale

To validate DHODH as a therapeutic target in neuroblastoma, we first analyzed clinical data and conducted cell-based DHODH silencing experiments. We then employed docking simulations to identify FDA-approved drugs capable of inhibiting DHODH activity specifically in high-risk neuroblastoma. The efficacy of the candidate drug was evaluated through both cell-based assays and patient-derived organoid models to ensure translational relevance. To elucidate the molecular mechanisms underlying DHODH inhibition, we utilized Tandem Mass Tag (TMT) proteomic analysis to identify proteomic changes associated with DHODH inhibition. For TMT proteomic analysis, three independent biological replicates per treatment group (n = 3) were prepared, and each sample was analyzed once (single technical replicate) to maintain experimental consistency and minimize batch effects (26). The use of three biological replicates is a widely accepted standard in proteomics studies, providing sufficient statistical power to detect meaningful changes while balancing resource constraints (27). This approach revealed a novel mechanism involving lipid metabolism disruption and ferroptosis induction, offering new insights into potential therapeutic strategies for high-risk neuroblastoma. Statistical analyses were applied to assess the significance of changes in proteomic profiles, ensuring robust and reliable findings. All data are presented as the mean ± standard deviation (SD), unless stated otherwise, with a minimum of three biological replicates (n = 3). Comparisons between two groups were performed using an unpaired, two-tailed Student’s *t* test. A *p*-value of less than 0.05 was considered statistically significant.

### Clinical Data Analysis of Patients With Neuroblastoma Using the R2 Platform

Patients with neuroblastoma were categorized into two groups based on the median expression level of DHODH. Kaplan-Meier analyses for overall survival and event-free survival were performed using the “Tumor Neuroblastoma public - Versteeg - 88 - MAS5.0 - u133p2” dataset *via* the R2 Genomics Analysis and Visualization Platform (http://r2.amc.nl). This dataset includes expression profiles from 88 neuroblastoma tumor samples, analyzed by Affymetrix microarray (MAS5.0 normalization). It also provides detailed clinical information, including patient age at diagnosis, MYCN amplification status, gender, and survival outcomes. Survival differences between groups were evaluated using the log-rank test. Additionally, DHODH gene expression was analyzed across different patient subgroups, including neuroblastoma risk levels, stages, and MYCN amplification status. Differences in DHODH gene expression between these subgroups were evaluated using the ANOVA analysis to identify significant associations.

### Cell Culture

The human neuroblastoma cell line SK-N-BE(2)C (CRL-2268) was purchased from the American Type Culture Collection (ATCC) and tested negative for *mycoplasma* contamination. SK-N-BE(2)C cells were cultured in Dulbecco’s Modified Eagle Medium (DMEM; Gibco Laboratories) supplemented with 10% fetal bovine serum (FBS; Gibco Laboratories). All cell cultures were cultured in an incubator at 37 °C with 5% CO2, pH 7.4, and passaged routinely when cells reached 80 to 90% confluence. For passaging, cells were trypsinized using a trypsin-EDTA solution (Gibco Laboratories) and transferred to new Petri dishes for continued incubation.

### Stable Knockdown of DHODH Using shRNA

SK-N-BE(2)C cells were seeded at a density of 1.1 × 10^6^ cells in a 6 cm plate and incubated for 24 h. Cells were then transfected with 13.5 μg of shRNA plasmid targeting DHODH or lacZ (control) (RNAi core, Academia Sinica) using Lipofectamine 3000 (Thermo Scientific) in serum-free medium. Six hours post-transfection, the medium was replaced with fresh medium containing FBS. After 72 h, cells were selected using DMEM medium containing 1 μg/ml puromycin. Stable cells expressing puromycin resistance were maintained in DMEM supplemented with 1 μg/ml puromycin and 250 μM uridine. DHODH protein expression levels were subsequently measured in the transfected cells to confirm the efficiency of DHODH knockdown.

### Western Blot Analysis

Cells were lysed with RIPA buffer containing 50 mM Tris-HCl (BioShop), 1% NP-40 (BioShop), 0.1% SDS (BioShop), 150 mM NaCl (BioShop), 0.5% Sodium deoxycholate (SDC; Sigma-Aldrich), adjusted to pH 7.4. Protease inhibitor cocktail (PI; MCE, Monmouth Junction), phosphatase inhibitor cocktail I (PPI I, BioShop), and phosphatase inhibitor cocktail II (PPI II, BioShop) were added to the RIPA buffer to prevent protein degradation. The cell lysates were sonicated on ice for 2 min using a Labsonic M ultrasonic homogenizer (Sartorius AG), followed by centrifugation at 15,000*g* for 30 min at 4 °C. Protein concentrations of the resulting supernatants were determined using the Pierce BCA Protein Assay Kit (Thermo Scientific). amounts of protein (30 μg per sample) were mixed with 1× sample buffer, separated by SDS-PAGE, and transferred onto hydrophobic polyvinylidene difluoride (PVDF) membranes (Millipore). The membranes were blocked using 5% non-fat milk in PBST for 1 h at room temperature and then incubated overnight at 4 °C with primary antibodies against DHODH (1:500 dilution; Santa Cruz, Dallas, Texas, USA), Caspase 3 (1:1000; GeneTex), PARP (1:1000; GeneTex), HMGCS1 (1: 1000 dilution; Proteintech), HMGCR (1:1000; Proteintech), SQLE (1:500; Proteintech), FDFT-1 (1;1000; Proteintech) or β-actin (1:10,000 dilution; Millipore). The membranes were then incubated for 2.5 h at room temperature with an anti-rabbit or anti-mouse IgG-HRP secondary antibody (1:5000; Sigma-Aldrich). Protein bands were visualized using UVP ChemStudio Plus imaging system (Analytik Jena AG).

### MTS Assay

The viability of cells post-treatment was measured using MTS assays (CellTiter 96 AQueous MTS Reagent, Promega Corporation). Cells were seeded onto 96-well plates at 10,000 cells in 100 μl medium per well. If the addition of a drug was required, it was applied 24 h after seeding. At the indicated time point, cells were treated with a 20 μl MTS/PMS (MTS: PMS; 1: 20) solution. The plate was then incubated at 37 °C with 5% CO2 for 2 h, followed by measuring 490 nm absorbance using SpectraMax i3x Multi-Mode Microplate Reader (Molecular Devices). Each experiment included at least three biological replicates, with each biological replicate containing at least three technical replicates. The relative cell viability is presented as histograms.

### Wound Healing Assay

To set up the wound healing assay, a 2-well cell culture insert was placed on a 6 cm dish. A total of 20,000 cells were seeded into the culture insert with 100 μl of medium. The following day, the insert was removed, and the dish was filled with fresh medium. If necessary, the drug treatment was added along with the medium. Images were captured using a microscope (Leica) at 24, 48, and 72 h after scratch formation. The area of the wound gap was quantified using ImageJ software. All experiments were performed in triplicate and repeated at least three times.

### Transwell Migration Assay

After drug treatment, 200,000 cells were resuspended in serum-free DMEM and seeded into the upper chamber of a transwell. Subsequently, the upper chamber was placed into a well containing 600 μl DMEM supplemented with 10% FBS. After incubating for 16 h, the chamber was transferred to a methanol solution to fix the cells for 24 h. Following fixation, the chamber was moved to a well containing crystal violet (Sigma-Aldrich) staining solution and incubated for an additional 24 h. Finally, a microscope was used to capture images. The migrating cells were measured using ImageJ software. All experiments were performed in triplicate wells and repeated at least three times.

### Colony Formation Assay

A total of 1.5 × 10^3^ SK-N-BE(2)C cells and DHODH knockdown SK-N-BE(2)C cells were seeded in six-well plates with culture medium and allowed to grow for 21 days. For experiments involving orotate supplementation, cells were seeded in six-well plates and cultured for 24 h before being treated with orotate. After the incubation period, the resulting colonies were fixed overnight with 100% methanol and then stained using a 1% crystal violet solution.

### ATP Assay

CellTiter-Glo Luminescent Cell Viability Assay (Promega) was used to quantify ATP levels. A total of 10,000 cells were seeded into white 96-well plates in 100 μl medium per well. At the designated experimental time point, 100 μl CellTiter-Glo Reagent was added to each well following the manufacturer’s instructions. After a 10-minute incubation at room temperature, luminescence was measured using a SpectraMax i3x Multi-Mode Microplate Reader (Molecular Devices). Each experiment was performed with at least three biological replicates, each containing a minimum of three technical replicates.

### Mitochondria Morphological Analysis

SK-N-BE(2)C cells (5∗10^5^ cells) were seeded on 18∗18 mm coverslip placed in a 6-well plate and incubated for 24 h. Cells were then treated with the indicated drugs or siRNA for 72 h. Mitochondria were stained using MitotrackerRed (1:2000 in serum-free medium) for 30 min, followed by fixation with 3.7% paraformaldehyde for 10 min. Coverslips were then washed with TBS and counterstained with 16 μl DAPI. All staining procedures were conducted in the dark. Fluorescence signals were captured using an LSM 780 laser scanning confocal microscope, and images were processed using Zen 2010 software. Mitochondrial morphology was analyzed using the ImageJ software with the mitophagy_ver_1_44_final_colocalization_f package.

### Molecular Docking

Molecular docking was performed using AutoDock Vina 4.2, which integrates an empirical free energy force field with a Lamarckian genetic algorithm, allowing for rapid computations of bound conformations and the prediction of free energy of binding. This grid-based method incorporates intramolecular energies into the predicted binding affinities, enabling efficient evaluation of the binding energies for various conformations (28). The crystal structure of DHODH (PDB ID: 6FMD) was obtained from the Protein Data Bank, selected for its high resolution. Prior to docking, water molecules and cofactors were removed using the SwissModel tool (https://swissmodel.expasy.org/). Small-molecule compounds for docking were sourced from the PubChem database (https://pubchem.ncbi.nlm.nih.gov/). We employed global docking to screen the entire DHODH protein structure for potential binding sites. Post-docking, LigPlot+ (version: 2.2) (https://www.ebi.ac.uk/thornton-srv/software/LigPlus/) was used to visualize and analyze the amino acid residues of DHODH that interact with the small molecular compounds.

### Drug Preparation

Regorafenib, Liproxstatib, Lefiunomide, Brequinar, Canagliflozin, Droperidol, Pexidartinib, and Sorafenib (MCE) were dissolved in DMSO at a stock concentration and subsequently diluted to the desired experimental concentration. All drug treatment procedures were conducted under dark conditions to prevent light-induced degradation.

### Protein Expression and Purification of DHODH

A truncated form of human DHODH (Met30-Arg396) was cloned into pET-28b plasmid using Nco1 and EcoR1 restriction sites. The construct was designed to produce DHODH as an N-terminal 10× histidine-tagged fusion protein (DHODH sequence: MGHHHHHHHHHHMATGDERFYAEHLMPTLQGLLDPESAHRLAVRFTSLGLLPRARFQDSDMLEVRVLGHKFRNPVGIAAGFDKHGEAVDGLYKMGFGFVEIGSVTPKPQEGNPRPRVFRLPEDQAVINRYGFNSHGLSVVEHRLRARQQKQAKLTEDGLPLGVNLGKNKTSVDAAEDYAEGVRVLGPLADYLVVNVSSPNTAGLRSLQGKAELRRLLTKVLQERDGLRRVHRPAVLVKIAPDLTSQDKEDIASVVKELGIDGLIVTNTTVSRPAGLQGALRSETGGLSGKPLRDLSTQTIREMYALTQGRVPIIGVGGVSSGQDALEKIRAGASLVQLYTALTFWGPPVVGKVKRELEALLKEQGFGGVTDAIGADHRR). The DHODH plasmid was transformed into *E. coli* BL21(DE3) pLysS cells for protein expression. Cells were cultured at 37 °C in 2× Yeast Extract Tryptone medium with 50 μg/ml kanamycin and 25 μg/ml chloramphenicol. Protein expression was induced with 1 μM isopropyl-β-d-thiogalactoside (IPTG) 20 h at 25 °C when the OD600 reached 0.6 to 0.8. The *E.coli* were harvested by centrifuging at 6000 rpm for 30 min and the resulting cell pellets were resuspended in 50 ml lysis buffer (50 mM HEPES, 300 mM NaCl, 10% glycerol, 0.04 mg/ml lysozyme, 250 μl PMSF, 20 beta-ME, 1% triton-X). A The suspension was sonicated for 30 min and then centrifuged at 25,000*g* for 30 min at 4 °C to separate the supernatant. The supernatant was filtered with a 0.45 μm filter and passed through an activated open column containing HisTag Binding Agarose (BioShop). DHODH was eluted using a step gradient of imidazole (300–500 mM) in buffer A (300 mM NaCl, 50 mM HEPES, 10% glycerol). Due to its characteristic binding with FMN, the eluted DHODH protein appears as a transparent yellow color. Elution was stopped once the yellow color disappeared. Finally, the protein was concentrated and further purified using an Amicon Ultra-15 Centrifugal Filter Unit (Millipore) and PD-10 desalting columns (Cytiva).

### Thermal Shift Assay

DHODH was diluted to a final concentration of a 50 μM in buffer A. For each PCR tube, 19 μl of the DHODH solution and 1 μl of Regorafenib at varying concentrations (dissolved in DMSO) were added. Due to DHODH's intrinsic ability to bind FMN, the use of Sybr Orange was not required. The protein melting temperature was determined by measuring the melt curve using a StepOne Real-Time PCR (Applied Biosystems).

### DHODH Activity Assay

The DHODH activity assay measures the enzyme’s activity by detecting the production of orotate through its reaction with the dihydroorotate (DHO) substrate. The orotate formed is then quantified using the fluorogenic reagent 4-trifluoromethyl-benzamidoxime (4-TFMBAO), which specifically reacts with orotic acid to produce a fluorescent signal (29). First, cells were harvested, washed twice with PBS, and resuspended in RIPA buffer. The cells were lysed by sonication for 2 min. Next, 300 μl of the cell lysate (1 × 10^6^ cells/ml) was incubated in a final reaction volume of 1.0 ml ddH_2_O containing 500 μM DHO, 200 mM K_2_CO_3_-HCl (pH 8.0), 100 μM coenzyme Q10, and 0.2% Triton X-100 at 37 °C for 30 or 60 min. After incubation, 100 μl of the enzyme reaction mixture was mixed with 150 μl ddH_2_O, 250 μl 4.0 mM 4-TFMBAO, 250 μl of 8.0 mM K_3_[Fe(CN)_6_], and 250 μl of 80 mM K_2_CO_3_, followed by heating at 80 °C for 4 min. The reaction was stopped by cooling on ice, and the fluorescence intensity was measured using a SpectraMax i3x Multi-Mode Microplate Reader (Molecular Devices) with excitation and emission wavelengths of 340 nm and 460 nm, respectively. Relative fluorescence intensity values were presented as histograms.

### Real-Time Cell Analysis (RTCA)

A total of 50 μl of growth medium was added to each well of an E-plate and placed in the RTCA machine for 30 min. SK-N-BE(2)C cells were seeded at a density of 5000 cells per well and incubated for 24 h. Subsequently, cells were treated with the specified drugs for 96 h. The drugs were initially dissolved in DMSO and then diluted in the growth medium to reach the desired final concentration.

### Establishment of Primary Cancer Cell Culture from Patient Tissues

Two neuroblastoma tissue samples were obtained with approval from the National Taiwan University Hospital (IRB number: 202311011RIND). All procedures involving human-derived samples were conducted in accordance with the ethical standards of the institutional review board and the principles outlined in the Declaration of Helsinki. Tumor samples were placed in tubes and washed twice with PBS containing 5 μg/ml Primocin (InvitroGen). The tissues were then enzymatically digested using 500 μg/ml Collagenase I and Collagenase IV (Worthington) at 37 °C for 2 h to homogenize the samples. Following digestion, the samples were washed with 10 volumes (w/v) of PBS. To achieve a single-cell suspension, the resulting turbid supernatant was sequentially filtered through cell strainers of progressively smaller pore size, down to 70 μm. The isolated cells were initially cultured in DMEM-F12/Neurobasal medium (Thermo) supplemented with 10% FBS, 1% B27 supplement (Thermo), 0.5% N2 supplement (Thermo), 20 ng/ml bFGF (Thermo), and 20 ng/ml EGF (Thermo). The cells were subsequently maintained in DMEM-F12/Neurobasal medium with 10% FBS, 1% B27 supplement (Thermo), and 0.5% N2 supplement (Thermo), and cultured at 37 °C in a humidified incubator with 5% CO_2_.

### Flow Cytometric Analysis

Cells were washed with 500 μl wash buffer (1× PBS, 1% BSA, 0.1% NaN_3_, 0.1% EDTA, pH 7.4) at 300 × *g* for 5 min. After discarding the supernatant, the cells were resuspended in 50 μl of wash buffer and incubated with 1 μl Fc Blocking reagent (BD Biosciences) for 10 min at room temperature (RT). Next, the cells were resuspended in 50 μl wash buffer and incubated with GD2 antibody (Millipore) at 4 °C for 30 min. The cells were then stained with CD45-PE (Abcam), CD56-APC (Abcam), and Alexa-488 antibodies (1:200) in a total volume of 100 μl for 30 min at 4 °C in the dark. Isotype controls included anti-IgG2a-Alexa488 (Abcam), anti-IgG2a-APC (Abcam), and anti-IgG1-PE (Abcam) antibodies (30). Following two washes with 300 μl of wash buffer, the cells were resuspended in 300 μl sorting buffer (0.04% UltraPure BSA in PBS; Thermo). To exclude dead cells, live/dead blue dye (1:2000) was added to a total volume of 300 μl before the addition of antibodies. For each sample, 20,000 cells were analyzed using a FACSCanto (BD Biosciences), and the data were processed with FlowJo V10 software (Ashland).

### Organoid Culture and Drug Treatment

A total of 10,000 CD45^-^/CD56^+^ single cells were cultured in rotary bioreactors (CelVivo ApS, Blommenslyst, Denmark), with the rotation speed adjusted according to spheroid size during growth. The medium was refreshed three times a week. Once the organoids reached sufficient size, they were transferred along with the culture medium into a 15 ml tube using a P1000 pipette with the tip cut off. The tube was centrifuged at 300 × *g* for 5 min at 4 °C, and the supernatant was carefully removed. The pellet was then resuspended in TrypLE and incubated at 37 °C for 1 to 2 min to dissociate the organoids (31). After dissociation, organoid growth medium was added, and the organoids were seeded into a 96-well plate (1000 organoids/well) or a 12-well plate (2000 organoids/well with Matrigel) for subsequent Regorafenib treatment.

### Cell Apoptosis Assay

Organoids or cells were collected and centrifuged at 1500g for 5 min to obtain 2 × 10^6^ cells. Apoptotic cells were then stained using the Annexin V-FITC Apoptosis Detection Kit (BD Pharmingen) according to the manufacturer’s instructions. Cells were incubated with Annexin V and propidium iodide (PI) for 15 min each and immediately analyzed using a FACSAria III flow cytometer (BD Biosciences).

In the regorafenib treatment group, SK-N-BE(2)C cells were treated with regorafenib at concentrations of 2.5 μl and 5 μl for 72 h. Cells treated with the same volume of DMSO served as controls. For each concentration, three biological replicates of control cells and regorafenib-treated SK-N-BE(2)C cells were obtained, and each biological sample underwent duplicate LC-MS/MS analyses. In the DHODH knockdown group, SK-N-BE(2)C cells transfected with #A4 and #A5 shRNAs were used as DHODH knockdown cells, while SK-N-BE(2)C cells transfected with an empty vector served as controls. For each cell type (LacZ, #A4, and #A5), three biological replicates were obtained, and each biological sample similarly underwent duplicate LC-MS/MS analyses.

### Proteome Sample Preparation and TMT Labeling

After treatment, trypsinized cells were collected and lysed in the phase-transfer surfactant (PTS) lysis buffer 12 mM sodium deoxycholate (SDC; Sigma-Aldrich), 12 mM sodium N-lauroylsarcosinate (SLS; Sigma-Aldrich), 100 mM triethylammonium bicarbonate (TEAB; Sigma-Aldrich), 1% protease inhibitor cocktail. Cells were sonicated for 2 min to ensure complete lysis, and cell debris was removed by centrifugation at 15,000*g* for 30 min and 4 °C. The supernatant was collected, and protein concentration was determined using the Pierce BCA Protein Assay Kit (Thermo Scientific). For mass spectrometry analysis, 50 μg of protein from each sample was reduced with 10 mM TCEP (Millipore) for 30 min and followed by alkylating with 25 mM 2-chloroacetamide (CAA; Sigma-Aldrich) for 30 min. Samples were incubated at 37 °C in a thermomixer set to 1000 rpm. For digestion, samples were diluted 5-fold with 50 mM ammonium bicarbonate (Sigma-Aldrich) and first digested with 0.5 μg Lys-C (Wako) for 3 h at room temperature, followed by overnight digestion with 0.5 μg trypsin (Promega) at 37 °C.

The digestion was terminated by adding trifluoroacetic acid (TFA) to a final concentration of 0.5%. Ethyl acetate (1:1 v/v) was used to extract the detergents from the sample. The mixture was centrifuged at 17,000g for 2 min to remove insoluble material, and the supernatant was desalted using SDB-XC Empore disk membranes reverse-phase StageTips (32). The resulting peptides were quantified using the Pierce Quantitative Colorimetric Peptide Assay (Thermo Scientific). Desalted peptides (10 μg per sample) were dried using vacuum centrifugation and subjected to Tandem Mass Tag (TMT) labeling. Peptides were dissolved in 5 μl of 200 mM HEPES (pH 8.5) and incubated with an equal volume of TMTpro 18-plex Isobaric Label Reagent at room temperature for 1 h. The labeling reaction was quenched by adding 0.33% hydroxylamine and incubating for 15 min at room temperature. TMT-labeled samples were pooled and fractionated into eight fractions using basic reversed-phase fractionation on C18 StageTips (33). Finally, the fractions were desalted using C18 StageTips, dried, and prepared for subsequent LC-MS/MS analysis.

### LC-MS/MS Analysis

Peptides were dissolved in 2% acetonitrile (ACN) with 0.1% formic acid (FA) and analyzed using an Orbitrap Fusion Lumos Tribrid quadrupole-ion trap-Orbitrap mass spectrometer (Thermo Scientific) coupled to an Ultimate 3000 NanoLC system (Thermo Scientific). Peptides were loaded onto a C18 Acclaim PepMap NanoLC column (75 μm inner diameter, 25 cm length, packed with 2 μm particles with a pore size of 100 Å; Thermo Fisher Scientific). Mobile phase A consisted of 0.1% formic acid in water, while mobile phase B was 100% ACN with 0.1% formic acid.

Peptide separation was achieved using a gradient of 2% to 40% ACN containing 0.1% formic acid over 50 min at a flow rate of 300 nl/min. The mass spectrometer was operated in data-dependent acquisition (DDA) mode, automatically switching between MS1 and MS2 scans. MS1 spectra were collected in the range of 350 to 1700 m/z at a resolution of 120,000 (at 200 m/z) in the Orbitrap, with an AGC target of 5 × 10^5^ and a maximum injection time of 50 ms. The instrument operated in the highest speed mode, with a 3-s cycle for MS1 and MS2 scans. Peptide ions with charge states ranging from 2 to 7 were isolated using a 1.4 Da window and fragmented *via* high-energy collision-induced dissociation (HCD) at 38% normalized collision energy (NCE). Fragment spectra were acquired in the Orbitrap at a resolution of 60,000. using an AGC target of 5 × 10^4^. A dynamic exclusion time of 60 s was applied to prevent repeated analysis of previously selected ions.

### Proteome Data Analysis

Raw MS data files were processed using MaxQuant3 software (version 2.4.0.0) (34) for peptide and protein identification and quantification. Peptides were identified against the SwissProt human protein database (version 2023_06), which contains 229,796 protein sequences retrieved from UniProtKB, using the Andromeda peptide search engine. Reporter ion MS2 analysis was performed using corrected 18-plex TMT isobaric labels (35). Variable modifications included methionine oxidation and N-terminal protein acetylation, while carbamidomethylation of cysteine residues was set as a fixed modification. Trypsin/P was specified as the enzyme for digestion, allowing up to two missed cleavage sites. The peptide tolerance for the initial search was set to 20 ppm, while the main search tolerance was adjusted to 4.5 ppm. The FTMS MS/MS matching tolerance was also configured at 20 ppm. The false discovery rate (FDR) for proteins and peptides was set to 1%. The “match between runs” feature was enabled with default settings for improved protein identification. After processing in MaxQuant, ProteinGroups files were further analyzed using Perseus software (version 2.0.10.0) (36). Differentially expressed proteins were identified based on the following criteria: a minimum of one unique peptide, an average log2 fold change ratio of ≥0.3 for upregulation and ≤ −0.3 for downregulation, and a *p*-value <0.05.

### Functional Enrichment

Gene Ontology (GO) analysis of differentially expressed proteins was performed using the DAVID (Database for Annotation, Visualization, and Integrated Discovery; version: v2023q4) tool (37). The Biological Process (BP) category was used to identify distinct functional groups and assess their statistical significance based on differential protein expression.

### Lipid Peroxidation Assay

For confocal microscope analysis, 245,000 cells were seeded in a 6 cm dish. If drug treatment was required, it was applied 24 h after seeding. Following the designated treatment period, the medium was removed, and cells were rinsed once with PBS. Cells were then fixed with 3.75% paraformaldehyde (PFA; Sigma-Aldrich) for 30 min at 37 °C. After fixation, cells were washed with PBS for 10 min and incubated with 1 ml of 5 μM BODIPY 581/591 C11 (Invitrogen) in PBS per dish for 30 min at 37 °C. The cells were subsequently washed twice with PBS (10 min each) and stained with 1 μg/ml Hoechst 33,342 for 30 min at 37 °C. Fluorescence signals from BODIPY and Hoechst 33,342-stained nuclei were visualized using a Zeiss LSM 780 laser scanning confocal microscope.

For flow cytometry analysis, 720,000 cells were seeded in a 6 cm dish. If drug treatment was required, it was administered 24 h after seeding. After treatment period, cells were rinsed once with PBS and then incubated with 5 μM BODIPY 581/591 C11 in PBS for 30 min at 37 °C. Following incubation, cells were rinsed once with PBS, trypsinized, and resuspended in 500 μl of PBS. The cell suspension was passed through a 35 μm cell strainer and analyzed using a FACSAria III flow cytometer (BD Biosciences) with a 488 mm laser for excitation. A minimum of 5000 events were analyzed per sample, and data were processed using FlowJo software (FlowJo).

### Lipid Droplet Staining

For the confocal microscope, lipid droplets were stained using BODIPY 493/503 (Invitrogen) following a similar protocol as BODIPY 561/581 C11 staining. A total of 123,000 cells were seeded onto coverslips in a 12 well plate. If drug treatment was required, it was applied 24 h after seeding. After the designated treatment period, the medium was removed, and cells were rinsed once with PBS. Cells were then fixed with 3.75% paraformaldehyde (Sigma-Aldrich) for 30 min at 37 °C. Following fixation, cells were washed with PBS for 10 min and incubated in 500 μl of 2 μM BODIPY 493/503 (Invitrogen) in PBS per well for 15 min at 37 °C to stain lipid droplets. Cells were then washed three times with PBS (10 min each), and coverslips were mounted using Prolong Gold antifade reagent with DAPI.

For flow cytometry, 720,000 cells were seeded in a 6 cm dish. After the designated treatment period, cells were rinsed once with PBS and incubated with 2 μM BODIPY 493/503 in PBS for 30 min at 37 °C. Cells were rinsed once more with PBS, trypsinized and resuspended in 500 μl of PBS. The resulting cell suspension was filtered through a 35 μm cell strainer and analyzed using a FACSAria III (BD Biosciences) with a 488 nm excitation wavelength. Data from at least 5000 single cells per sample were collected and analyzed using FlowJo software (FlowJo).

### Cholesterol Assay

The concentration of cholesterol and cholesterol esters was measured using the Cholesterol/Cholesterol Ester-Glo Assay (Promega). A total of 10,000 cells were seeded into white 96-well plates with 100 μl medium per well. At the designated experimental time point, 50 μl of cholesterol lysis solution was added to each well, following the manufacturer’s protocol. After a 30-min incubation at 37 °C, 50 μl of cholesterol detection reagent was added and the plates were incubated at room temperature for 1 h. Luminescence was then measured using a SpectraMax i3x Multi-Mode Microplate Reader (Molecular Devices). Each experiment was performed with three biological replicates.

### Immunocytochemistry

Cells were seeded onto glass coverslips and allowed to adhere. If drug treatment was required, it was administered 24 h after seeding. Cells were then fixed with 3.7% paraformaldehyde in PBS for 30 min at 37 °C, followed by permeabilization with 0.1% Triton X-100 (Sigma-Aldrich) for 5 min at room temperature. To block nonspecific binding, cells were incubated in 5% BSA (BioShop) for 1 h at room temperature. After washing with PBS, cells were incubated 4 °C overnight with primary SQLE antibodies (1:100, proteintech). The following day, cells were incubated with Alexa-488 conjugated anti-rabbit secondary antibody (Invitrogen) for fluorescence detection. The endoplasmic reticulum (ER) was counterstained with ER-Tracker Red dye (BODIPY TR Glibenclamide; Invitrogen) and lipid droplets were counterstained with BODIPY 493/503. After mounting the cells onto the glass slides, images were captured using a Zeiss LSM780 confocal laser microscope (Zeiss).

### Nuclear Isolation

Cells were collected and centrifuged at 1500g for 5 min to obtain a total of 5 × 10^6^ cells. The cells were washed twice with cold PBS, and the supernatant was discarded to isolate the cell pellet. The pellet was resuspended in 800 μl of nuclear extraction buffer (20 mM Tris-HCl (BioShop), pH 7.4, 10 mM NaCl, 3 mM MgCl_2_ (Honeywell)) by gently pipetting up and down several times. The suspension was incubated on ice for 15 min, followed by the addition of 40 μl of 10% NP-40 (BioShop). The mixture was vortexed at the highest setting for 5 s and then centrifuged at 3000 rpm for 10 min at 4 °C. The supernatant, which contains the cytoplasmic fraction, was collected and saved. The pellet, representing the nuclear fraction, was resuspended in 500 μl of RIPA buffer and sonicated at 60% amplitude for 30 s. The sonicated sample was centrifuged at 15,000*g* for 15 min at 4 °C. The resulting supernatant (nuclear fraction) was transferred to a clean microcentrifuge tube and stored at −80 °C. Protein concentration was determined using the Bradford Protein Assay Kit (Bio-Rad).

### Mitochondrial Superoxide Measurement

A total of 1 × 10^6^ cells SK-N-BE(2)C were seeded in a 10 cm plate for 24 h, followed by Regorafenib treatment for 72h. A total of 2 × 106 Regorafenib -treated cells were collected and washed with PBS. After that, the cell pellets were re-suspended in PBS that contained 5 μM MitoSOX (Thermo) reagent and then incubated for 15 min at 37 °C in the dark. Samples were then washed with PBS and analyzed using FACSAria III flow cytometer.

## Results

### The Role of DHODH in neuroblastoma Progression

**Fig. 2:**
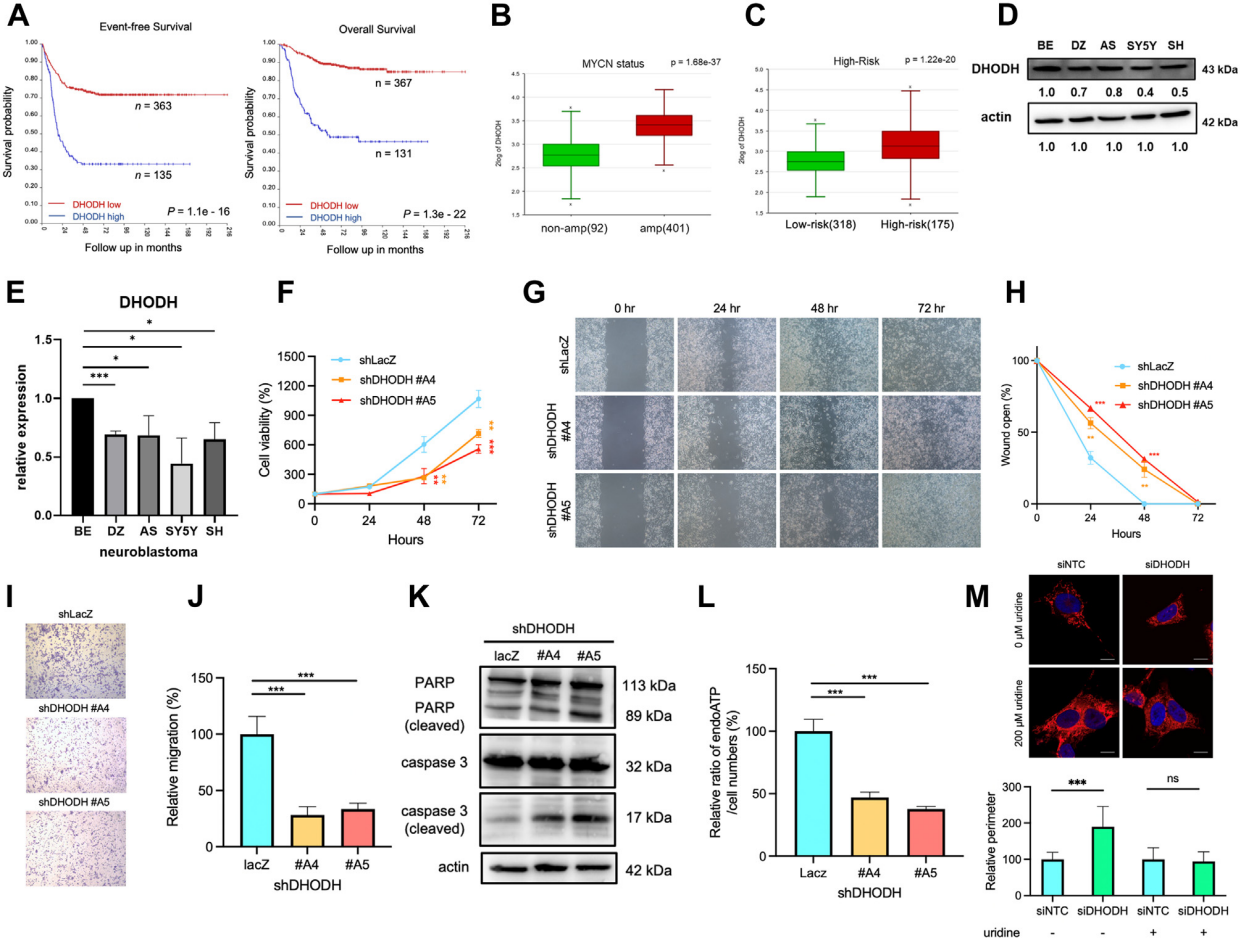
**Identification of DHODH as a potential therapeutic target in neuroblastoma**. *A*, survival probability of neuroblastoma patients categorized by high or low DHODH expression levels, shown for event-free survival (*left panel*) and overall survival (*right panel*), based on neuroblastoma dataset analysis. *B*, DHODH gene expression in neuroblastoma patients, stratified by MYCN status. *C*, DHODH gene expression in neuroblastoma patients, categorized into low-risk and high-risk groups. *D*, Western blot analysis of DHODH protein expression across multiple neuroblastoma cell lines. *E*, quantification of DHODH protein levels normalized to actin expression. *F*, MTS assay results for DHODH-knockdown SK-N-BE(2)C cells at various time point. *G*, representative images of the wound healing assay for DHODH knockdown SK-N-BE(2)C cells. *H*, quantification of wound healing, with the initial wound area (0-h) normalized to 100%. *I*, Transwell migration assay measuring the migration capacity of DHODH-knockdown SK-N-BE(2)C cells. *J*, relative number of migrating cells quantified using ImageJ software. *K*, Western blot analysis showing levels of cleaved caspase-3 and cleaved PARP, with β-actin as the loading control. *L*, measurement of endogenous ATP of DHODH-silenced SK-N-BE(2)C cells using an ATP assay. *M*, mitochondria and nuclei in SK-N-BE(2)C cells stained with MitotrackerRed and DAPI respectively. Images were captured using confocal microscopy, and mitochondrial perimeter was measured using ImageJ. Scale bar, 10 μm. All quantified data are presented as mean ± SD from three independent experiments. Statistical significance: ∗ *p* < 0.05, ∗∗ *p* < 0.01, ∗∗∗ *p* < 0.001.

To validate the importance of DHODH expression in neuroblastoma, we analyzed data from 496 neuroblastoma patients, primarily with high-risk neuroblastoma, using the R2: Genomics Analysis and Visualization Platform (http://r2.amc.nl). The dataset used was the TARGET-Asgharzadeh-249-custom-huex10 t database. Our findings revealed a strong association between elevated DHODH expression and decreased survival rates, both overall and event-free) (Fig. 2*A*). Additionally, high DHODH expression correlated with elevated MYCN status, increased risk probabilities, and more advanced stages of the disease (Fig. 2, *B* and *C* and supplemental Fig. S1*A*). These results suggest that DHODH plays a malignant role in neuroblastoma, aligning with previous research (23).

We first examined the DHODH expression levels in five neuroblastoma cell lines available in our laboratory. Among these, SK-N-BE(2)C exhibited the highest DHODH expression, followed by the SK-N-AS, while the SH-SY5Y showed the lowest expression level (Fig. 2, *D* and *E*). Given its elevated DHODH levels, MYCN amplification status, and clinical relevance as a late-stage, therapy-resistant model, we selected SK-N-BE(2)C for subsequent functional analyses. To further investigate the functional role of DHODH in neuroblastoma cells, we used shRNA to knock down DHODH expression in the SK-N-BE(2)C cell line (supplemental Fig. S1, *B*–*D*). The knockdown significantly reduced neuroblastoma cell growth and clonogenicity, an effect that was rescued by uridine (the end product of pyrimidine biosynthesis) and orotate (the product catalyzed by DHODH) (Fig. 2F and supplemental Fig. S1, *E*–*G*). Moreover, both wound healing and transwell migration assays showed that DHODH inhibition impaired neuroblastoma cell migration (Fig. 2, *G*–*J*). Markers of apoptosis, such as cleaved caspase 3 and cleaved PARP, also increased substantially following DHODH inhibition (Fig. 2*K* and supplemental Fig. S1*H*).

Given that DHODH is a mitochondrial enzyme involved in oxidative phosphorylation, we examined whether its inhibition affects mitochondrial function and dynamics. Our analysis showed that low DHODH expression led to a decrease in cellular ATP levels and induced a tendency towards mitochondrial fusion (Fig. 2, *L* and *M*). Together, these results highlight the crucial role of DHODH in neuroblastoma progression and suggest that targeting DHODH could be a promising therapeutic strategy.

### Identification of Regorafenib as a Potential DHODH Inhibitor

**Fig. 3:**
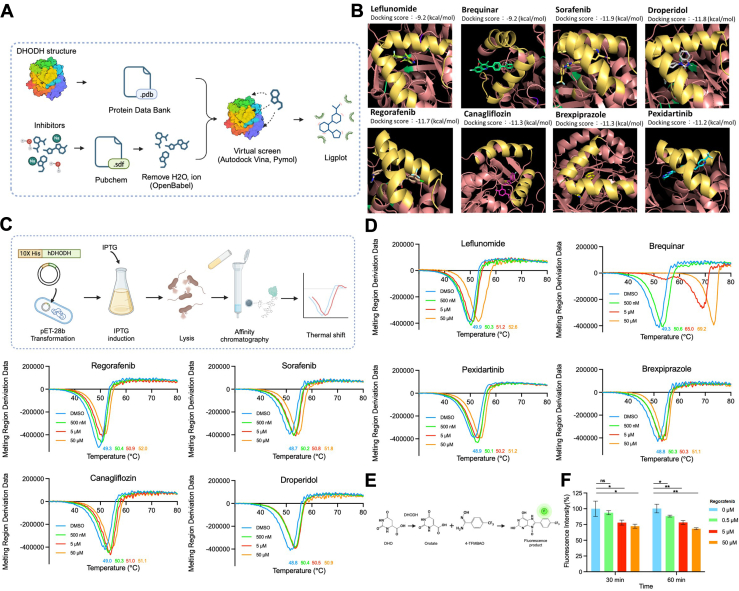
**Workflow and experimental evaluation of potential DHODH inhibitors’ effects on DHODH structural stability and enzymatic activity, including in silico docking simulations, thermal shift assays to assess protein stability, and enzyme activity assays to measure functional inhibition**. *A*, schematic representation of the docking simulation workflow. *B*, visualization of ligand-protein complex structures using Pymol. DHODH inhibitory sites are highlighted by two α-helix motifs in *yellow*, while the remaining DHODH structure is shown in *pink*. Leflunomide and Brequinar are known DHODH inhibitors, and the six other drugs are potential DHODH inhibitors identified through our docking simulation. Affinity scores for each drug are displayed above. *C*, Schematic diagram of DHODH protein purification process. *D*, Thermal shift assay results showing the structural stability of DHODH treated with four different concentrations of six candidate drugs and two known DHODH inhibitors. The melting temperature of DHODH was measured using a qPCR machine. *E*, schematic illustration of the DHODH activity assay principle. *F*, DHODH enzyme activity was quantified using a fluorescence-based DHODH activity assay. All quantified results are expressed as mean ± SD from three independent experiments. Statistical significance: ∗ *p* < 0.05, ∗∗ *p* < 0.01, ∗∗∗ *p* < 0.001.

Drug repositioning is a strategy aimed at identifying new therapeutic uses for existing drugs, significantly reducing the cost and time involved in drug development (38). In our study, we applied this approach to discover potential drugs targeting DHODH from a pool of FDA-approved compounds (supplemental Table S1). We performed global docking on the entire DHODH protein, beyond its known inhibitory site, to identify drugs with high binding affinity (Fig. 3*A*). PyMOL and LigPlot + analyses confirmed that several drugs could bind to DHODH in a manner similar to known inhibitors (Fig. 3*B* and supplemental Fig. S2*A*). Among the identified candidates, these FDA-approved drugs will undergo further validation at the protein and cellular levels.

To confirm the interaction between candidate drugs and DHODH, we purified human DHODH protein using a transmembrane domain-free variant tagged with 10× His-tag. The protein was expressed in *E. coli* BL21(DE3) pLysS and purified using ion metal affinity chromatography (IMAC) on a Fast Protein Liquid Chromatography (FPLC) system. The interaction with FMN was indicated by a color change from transparent to yellow (supplemental Fig. S2*B*). Elution with imidazole between 300 to 500 mM yielded a highly pure protein, as confirmed by SDS-PAGE analysis showing a single band at the expected molecular weight (supplemental Fig. S2*C*), suitable for further testing.

To validate the interaction between DHODH and candidate drugs from docking simulations, we used a thermal shift assay, measuring the intrinsic fluorescence of the FMN cofactor to assess protein stability and ligand binding (Fig. 3*C*). Regorafenib displayed a higher melting temperature (52.0 °C) than other uncharacterized DHODH inhibitors, indicating its superior ability to stabilize DHODH compared to other tested compounds (Fig. 3*D*). A concentration gradient analysis further demonstrated that increasing Regorafenib concentrations correlated with an increase in melting temperature (supplemental Fig. S2, *D* and *E*). To further validate the inhibitory effect of Regorafenib on DHODH enzymatic activity, we performed fluorescence-based assays in parallel with known DHODH inhibitors, Brequinar and Leflunomide. As expected, Brequinar strongly inhibited DHODH activity, although its clinical development has been hindered by toxicity concerns. Leflunomide, an FDA-approved DHODH inhibitor, also significantly reduced enzymatic activity. Notably, Regorafenib exhibited a comparable inhibitory effect to Leflunomide, reinforcing its potential to target DHODH activity (Fig. 3, *E* and *F* and supplemental Fig. S2, *F* and *G*).

We also collected 79 small molecules from the Protein Data Bank that bind to DHODH and conducted docking simulations comparing them to Regorafenib. The results revealed that Regorafenib ranked fourth in binding affinity, outperforming all other FDA-approved drugs (supplemental Table S2). These findings establish Regorafenib as a potent DHODH inhibitor. Given its promising profile, Regorafenib was selected for further experiments to explore its effects on DHODH and its mechanism of action in neuroblastoma cells.

### Regorafenib Inhibits the Proliferation of Neuroblastoma

**Fig. 4:**
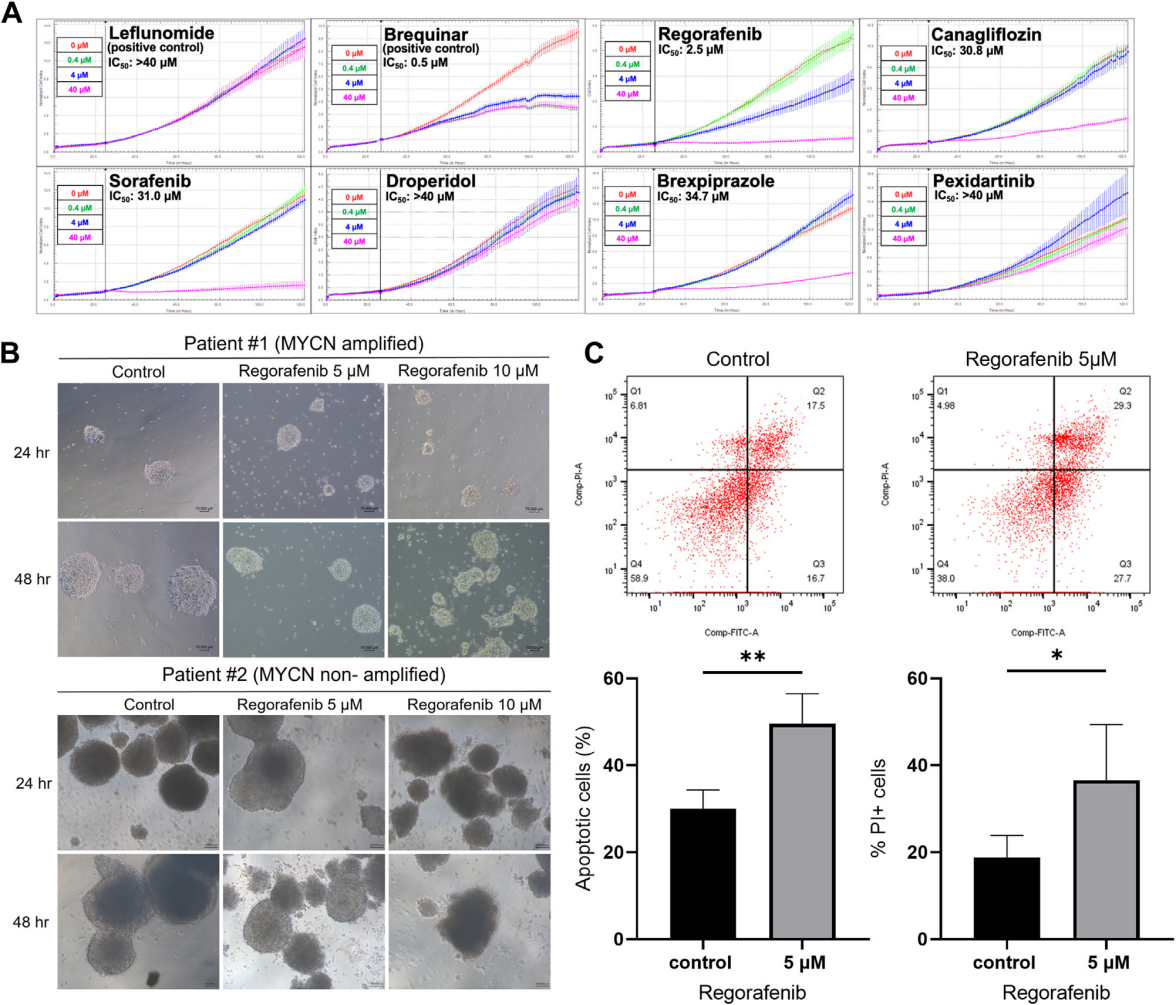
**Regorafenib inhibits neuroblastoma cell growth and suppresses the development of patient-derived organoids**. *A*, SK-N-BE(2)C cell viability following 72-h treatment with Regorafenib, measured using a real-time cell analysis (RTCA). Drug concentrations of 0 μM, 0.4 μM, 4 μM, and 40 μM were used for screening. IC_50_ values for each drug are indicated in the figures. *B*, morphological changes in MYCN-amplified and MYCN non-amplified patient-derived organoid after Regorafenib treatment. *C*, flow cytometry analysis of MYCN non-amplified organoids treated with 5 μM Regorafenib for 72 h, using Annexin V/PI staining. The dot plot shows apoptotic cells in the Q2 (late apoptosis) and Q3 (early apoptosis) quadrants, and PI-positive signals indicating cell death in the Q1 and Q2 quadrants. All quantified results are expressed as mean ± SD from three independent experiments. Statistical significance: ∗ *p* < 0.05, ∗∗ *p* < 0.01.

Through docking simulations, we identified six potential drug candidates, alongside two known DHODH inhibitors (Brequinar and Leflunomide), for further investigation. To evaluate their effects on cell viability, we employed a real-time cell analysis to measure electrical resistance and calculate the viability of neuroblastoma cells. We conducted large-scale screening across a concentration range of 0.4 to 40 μM. Among the candidates, Regorafenib demonstrated a particularly strong ability to reduce cell viability (Fig. 4*A*).

Further analysis confirmed that Regorafenib not only reduced cell viability but also impaired neuroblastoma cell migration, induced apoptosis, and increased mitochondrial ROS levels (supplemental Fig. S3, *A*–*J*). To assess whether the cytotoxic effects of Regorafenib are consistent across neuroblastoma subtypes, we examined its impact on both MYCN-amplified (SK-N-DZ) and MYCN non-amplified (SK-N-SH) cell lines. In both models, Regorafenib induced a concentration-dependent decrease in cell viability, indicating that its cytotoxic effects are not restricted by MYCN amplification status (supplemental Fig. S3*K*). Based on these screening and evaluation results, Regorafenib stands out as a promising therapeutic candidate for neuroblastoma, showing potent DHODH inhibition and significant reduction in cell viability. As a result, we have selected Regorafenib for further experimental investigation.

## Regorafenib Induces Morphological Changes and Apoptosis in Patient-Derived Neuroblastoma Organoids

In addition to cell-based experiments, we cultured organoids from patient samples to observe morphological changes and apoptosis following drug treatment (Fig. 4, *B* and *C*). Using microscopy, we monitored MYCN-amplified and MYCN non-amplified patient-derived organoids at 24 and 48 h post-treatment with Regorafenib. As the drug concentration and exposure time increased, the organoids exhibited signs of disintegration and fragmentation at their boundaries, indicating potential apoptosis. To further investigate this observation, we performed flow cytometry after 72 h of drug treatment. The results showed a significant increase in the proportion of apoptotic and PI-positive cells. These findings confirm that Regorafenib induces morphological alterations and promotes apoptosis in patient-derived organoids.

### Lipid Metabolism as a Potential Mechanism for DHODH inhibition in Neuroblastoma

**Fig. 5:**
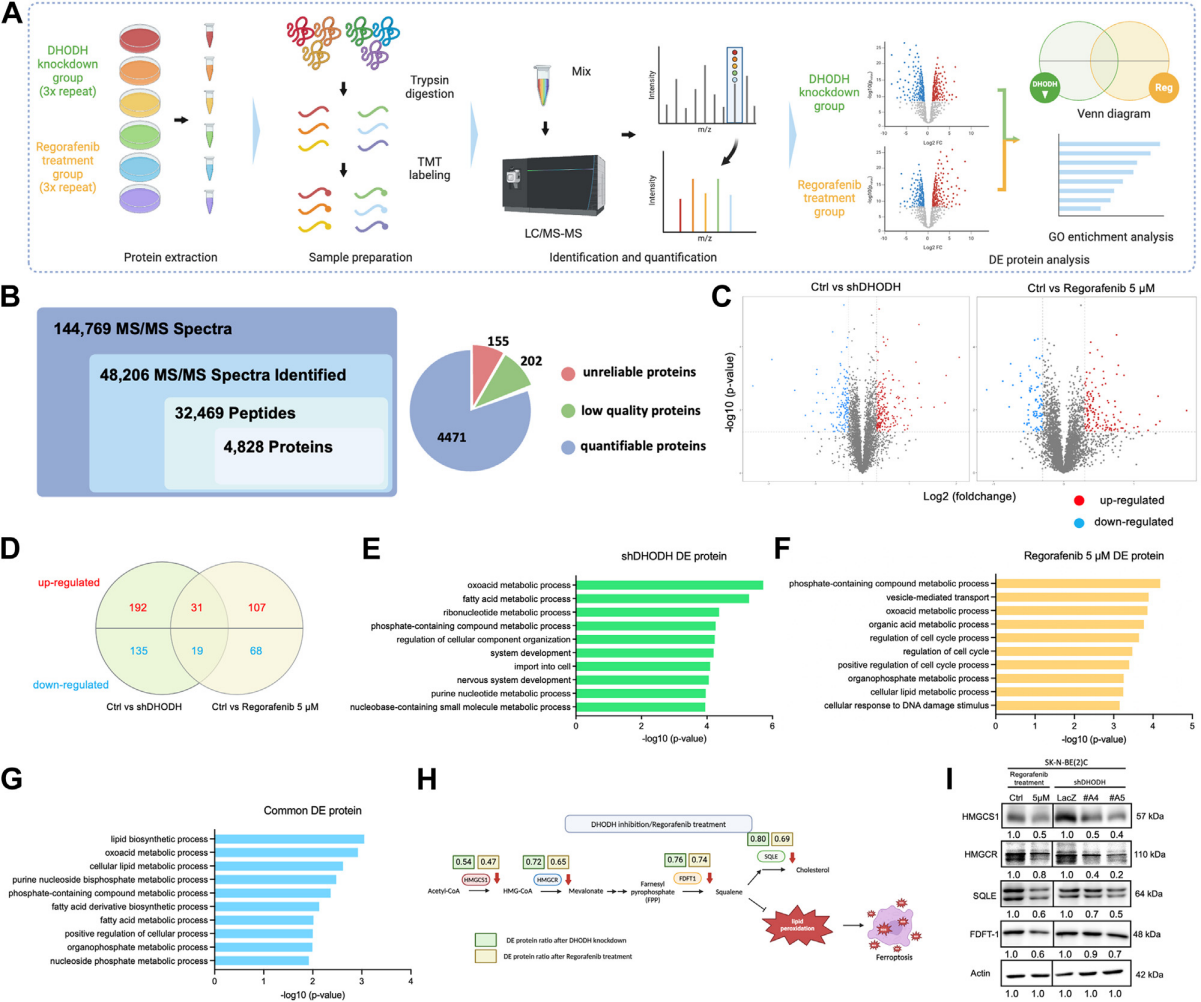
**Proteomic profiling of neuroblastoma cells following DHODH knockdown and Regorafenib treatment**. *A*, schematic overview of the TMT-based proteomics workflow for analyzing neuroblastoma cells subjected to DHODH knockdown and Regorafenib treatment. *B*, total number of identified MS/MS spectra, peptides, and proteins. The pie chart shows the quantitation and qualitative distribution of the global proteome across all groups. *C*, Volcano plots depicting fold change and statistical significance of quantified proteins between the Regorafenib-treated and DHODH knockdown groups (*p*-value <0.05; log2 fold change ≤ −0.3 or ≥0.3). *Blue spots* represented significantly down-regulated proteins, while red spots indicate significantly up-regulated proteins. *D*, Venn diagram illustrating the overlap of differentially expressed proteins (DE proteins) between Regorafenib-treated (5 μM) *versus* control and shDHODH-transfected (#A4 and #A5) vs. control groups. *E*, top GO 10 terms identified from DAVID functional annotation analysis based on biological processes for DE proteins in the DHODH knockdown group. *F*, Top GO 10 terms identified from DAVID functional annotation analysis based on biological processes for DE proteins in the regorafenib-treated group. *G*, Top 10 GO terms identified from DAVID functional annotation analysis based on biological processes for commonly differentially expressed proteins in both the DHODH knockdown and Regorafenib-treated group. *H*, ratios of identified proteins involved in the mevalonate pathway, with *yellow boxes* representing the ratios in the DHODH knockdown group and *green boxes* representing ratios in the regorafenib-treated group. *I*, validation of protein expressions in Regorafenib-treated and DHODH knockdown neuroblastoma cells using western blotting.

To investigate how Regorafenib-mediated DHODH inhibition affects neuroblastoma cells, we conducted TMT-based quantitative proteomics analysis using TMT labeling and LC/MS-MS (Fig. 5*A* and supplemental Fig. S4). SK-N-BE(2)C cells were treated with various concentrations of Regorafenib (5, 2.5, 0 μM) or transfected with shDHODH plasmid (LacZ, #A4, #A5). After data filtration, LC-MS/MS analysis identified 4471 quantifiable proteins (Fig. 5*B* and supplemental Table S3). We focused on the 5 μM Regorafenib treatment group and combined #A4 and #A5 as the DHODH knockdown group. Proteins with a log2 fold change ≥0.3 or ≤ −0.3 and a *p*-value ≤0.05 were considered differentially expressed (Fig. 5*C*).

We identified 377 differentially expressed proteins in the DHODH knockdown group (223 upregulated, 154 downregulated) and 225 in the Regorafenib group (138 upregulated, 87 downregulated) (supplemental Tables S4 and S5). Notably, 31 proteins were commonly upregulated and 19 were downregulated in both groups (Fig. 5D and supplemental Table S6). To further elucidate the biological pathways involved, we performed gene ontology (GO) analysis using DAVID, focusing on the Biological Process category. The analysis revealed that lipid metabolism-related processes were among the top 10 pathways with the lowest *p*-values in both the Regorafenib treatment and DHODH knockdown groups (Fig. 5, *E* and *F* and supplemental Tables S7 and S8). GO analysis of commonly differentially expressed proteins also highlighted lipid metabolism as the top-ranking pathway (Fig. 5*G* and supplemental Table S9). This suggests that lipid metabolism may be a key mechanism through which Regorafenib induces cell death in neuroblastoma by disrupting DHODH activity.

Interestingly, recent studies have highlighted the link between lipid metabolism, cancer and ferroptosis (39, 40, 41, 42, 43, 44). Our KEGG (Kyoto Encyclopedia of Genes and Genomes) analysis further confirmed that DHODH inhibition influences ferroptosis (supplemental Table S10), suggesting that DHODH may regulate ferroptosis through previously unknown mechanisms. To explore this, we examined proteins related to lipid metabolism among the commonly differentially expressed proteins. Our data showed that key enzymes involved in lipid metabolism, including HMGCS1, HMGCR, FDFT1, and SQLE, were downregulated in both Regorafenib-treated and DHODH knockdown groups. These proteins are critical components of the mevalonate pathway, which is responsible for producing cholesterol and other sterol isoprenoids. We validated this finding through Western blot analysis (Fig. 5, *H* and *I*). Inhibition of the mevalonate pathway has been shown to promote ferroptosis, as squalene accumulation prevents ferroptosis, while HMGCS1 activation enhances resistance to it (43, 45, 46, 47). Thus, we propose that Regorafenib may induce ferroptosis in neuroblastoma by disrupting DHODH activity and downregulating key proteins in the mevalonate pathway.

### Regorafenib Induces Ferroptosis Through DHODH Inhibition in Neuroblastoma Cells

**Fig. 6:**
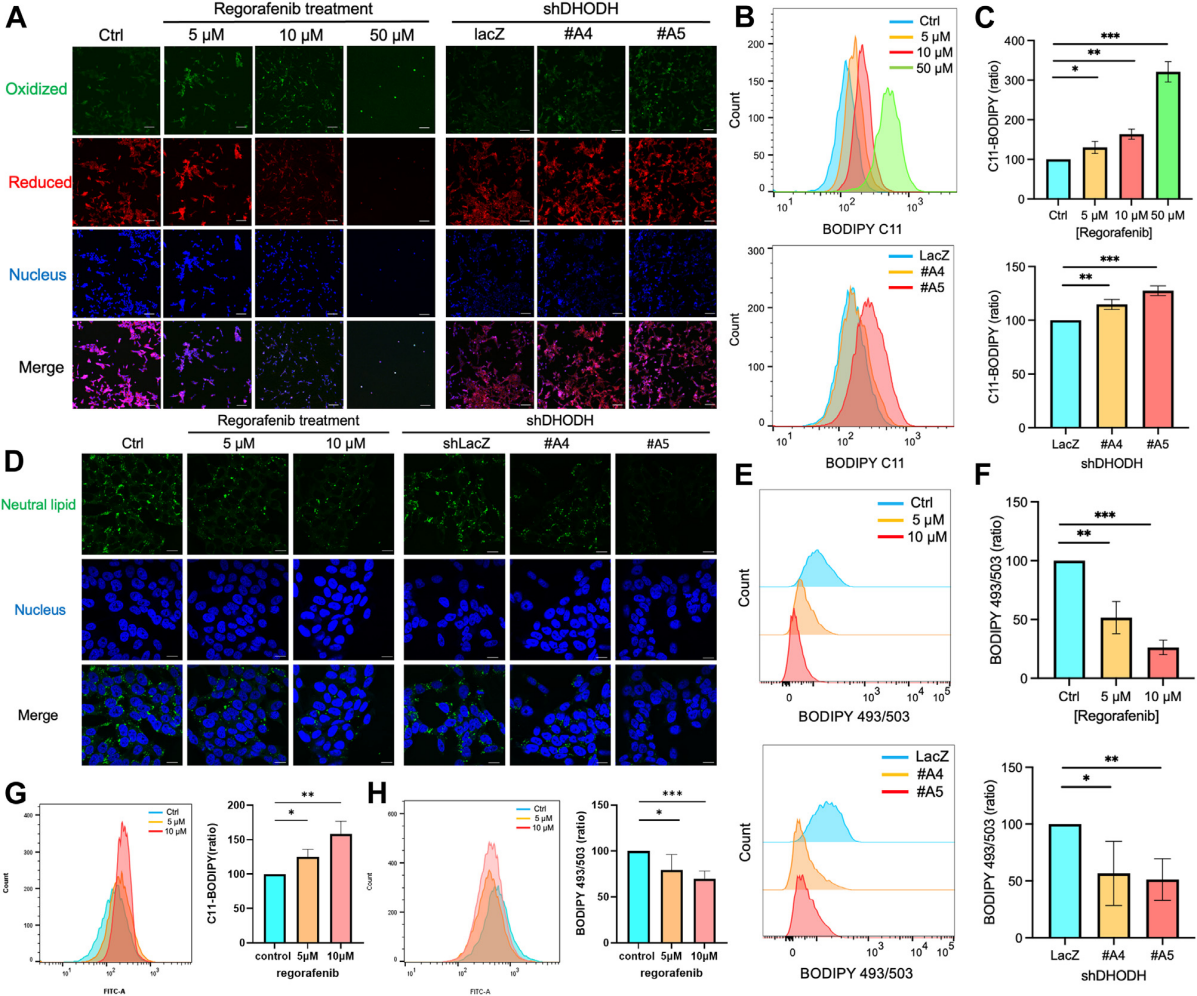
**Regorafenib induces ferroptosis by inhibiting DHODH and reduces lipid droplet production**. *A*, confocal images of neuroblastoma cells treated with Regorafenib for 48 h and DHODH knockdown neuroblastoma cells, stained with C11-BODIPY (*green*: oxidized form, *red*: non-oxidized form) and DAPI (*blue*: nuclei). Scale bar = 100 μm. *B*, evaluation of lipid peroxidation in neuroblastoma cells using C11-BODIPY staining. The *top panel* shows cells treated with DMSO, 5 μM Regorafenib, 10 μM Regorafenib, or 50 μM Regorafenib for 48 h, while the bottom panel shows cells with DHODH knockdown. Images are representative of three independent experiments. *C*, quantified fluorescence intensity of BODIPY 581/591 C11 from three independent experiments. *D*, confocal imaging of neuroblastoma cells treated with Regorafenib 48 h and DHODH knockdown neuroblastoma cells, stained with BODIPY 493/503 (*green*: neutral lipids) and DAPI (*blue*: nuclei). Scale bar = 20 μm. *E*, evaluation of neutral lipid content in neuroblastoma cells using BODIPY 493/503 staining. The *top panel* shows cells treated with DMSO, 5 μM Regorafenib, or 10 μM Regorafenib for 48 h, while the bottom panel shows cells with DHODH knockdown. *F*, quantified fluorescence intensity of BODIPY 493/503 from three or four independent experiments. *G*, assessment of lipid peroxidation in MYCN non-amplified patient-derived organoids using C11-BODIPY staining and flow cytometry analysis. *H*, quantification of neutral lipid content in MYCN non-amplified patient-derived organoids using BODIPY 493/503 staining and flow cytometry analysis. All quantified results are presented as mean ± SD from three independent experiments. Statistical significance: ∗*p* < 0.05, ∗∗*p* < 0.01, ∗∗∗*p* < 0.001.

GO enrichment analysis of differentially expressed proteins suggested that Regorafenib may induce ferroptosis in neuroblastoma cells by inhibiting DHODH and disrupting the mevalonate pathway. To validate this hypothesis, we assessed lipid peroxidation and ferroptosis using BODIPY 581/591 C11 staining. Confocal microscopy and flow cytometry revealed increased lipid peroxidation in cells treated with Regorafenib or subjected to DHODH knockdown (Fig. 6, *A*–*C* and supplemental Fig. S5, *A*–*C*). Moreover, co-treatment with the ferroptosis inhibitor liproxstatin-1 rescued cells from Regorafenib-induced cell death, confirming that Regorafenib triggers ferroptosis in neuroblastoma cells (supplemental Fig. S5*D*).

In addition, we observed that lipid droplets, which serve as storage for cholesterol and polyunsaturated fatty acids (PUFAs), might play a role in this process. Cholesterol, a key product of the mevalonate pathway, is stored within lipid droplets. Therefore, we hypothesized that Regorafenib treatment and DHODH knockdown might reduce lipid droplet production, thereby releasing sequestered PUFAs and increasing the cells’ susceptibility to ferroptosis. We initially used confocal microscopy (63× magnification) to assess lipid droplet formation in neuroblastoma cells treated with Regorafenib or subjected to DHODH knockdown. The images revealed a clear reduction in lipid droplet abundance within the observed fields (Fig. 6*D* and supplemental Fig. S5*E*). However, visual estimation alone may introduce bias due to variability in cell density and imaging conditions. To obtain quantitative data, we performed BODIPY 493/503 staining followed by flow cytometry analysis. This approach confirmed a significant, dose-dependent reduction in lipid droplet formation with both Regorafenib treatment and DHODH knockdown, although complete inhibition was not observed (Fig. 6, *E* and *F* and supplemental Fig. S5, *F*–*G*). To further validate our *in vitro* findings in a clinically relevant context, we analyzed MYCN non-amplified neuroblastoma patient-derived organoids. Following Regorafenib treatment, we observed consistent phenotypes, including increased lipid peroxidation and reduced lipid droplet (Fig. 6, *G* and *H*), supporting the induction of ferroptosis in this *ex vivo* model. This suggests that Regorafenib influences the production of neutral lipids by inhibiting DHODH, which subsequently reduces lipid droplet formation in neuroblastoma cells and promotes ferroptosis. These findings reveal a novel mechanism through which DHODH inhibition affects ferroptosis, highlighting Regorafenib’s potential as a therapeutic agent in neuroblastoma treatment.

### SQLE as a Key Protein Regulating Lipid Droplet Formation

**Fig. 7:**
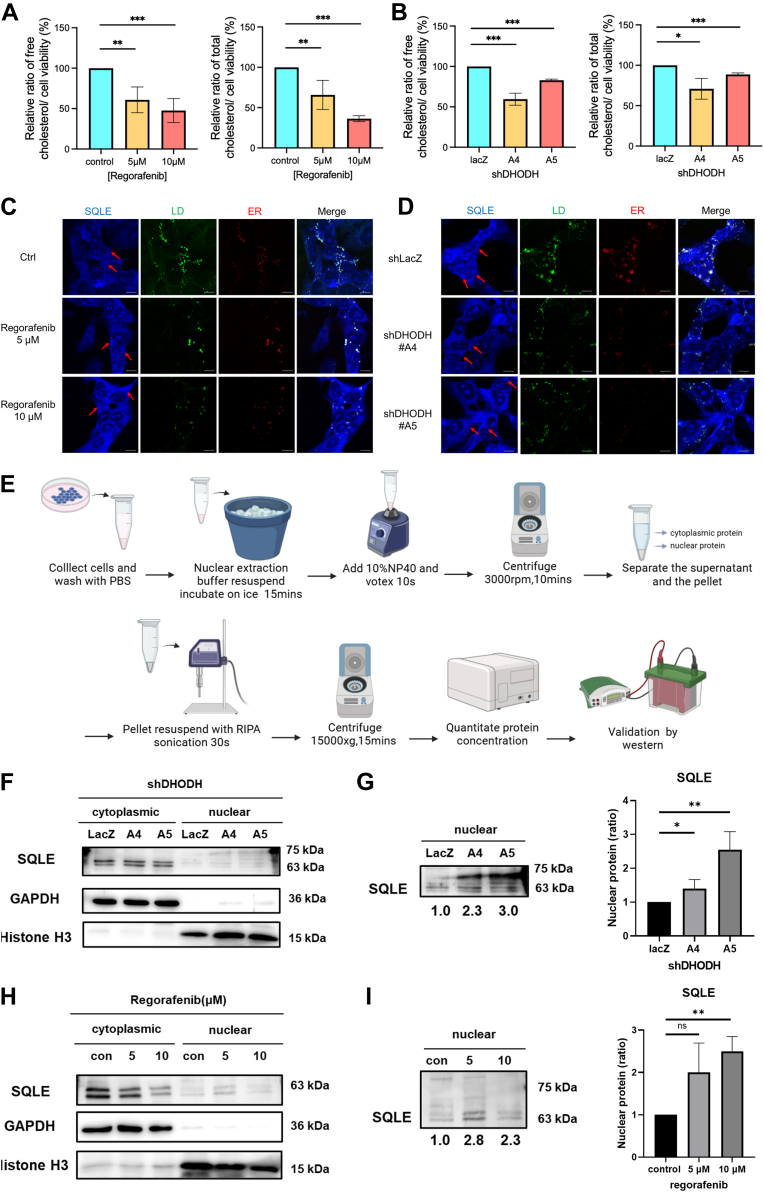
**DHODH inhibition reduces cholesterol production by disrupting the mevalonate pathway, leading to decreased lipid droplet formation.***A* and *B*, cholesterol concentration in neuroblastoma cells following Regorafenib treatment (*A*) and in DHODH-knockdown neuroblastoma cells (*B*), measured using the Cholesterol/Cholesterol Ester-Glo Assay. The *left panel* display free cholesterol levels, while the *right panel* shows total cholesterol levels. *C* and *D*, spatial distribution of SQLE in relation to lipid droplets, and the ER in neuroblastoma cells treated with Regorafenib (*C*) or subjected to DHODH knockdown cells (*D*), visualized by confocal fluorescence microscopy at 100 × magnification. Cells were stained with Alexa Fluor 405 to visualize SQLE, BODIPY 493/503 to visualize lipid droplets, and ER-tracker to visualize the ER. *Red arrows* indicate SQLE aggregation around lipid droplets. Scale bar = 10 μm. All quantified results are presented as mean ± SD from three independent experiments. Statistical significance: ∗*p* < 0.05, ∗∗*p* < 0.01, ∗∗∗*p* < 0.001. DHODH inhibition reduces cholesterol production by disrupting the mevalonate pathway, leading to decreased lipid droplet formation. *E*, Workflow for nuclear and cytoplasmic protein fractionation. *F*, Western blot analysis of SQLE in nuclear and cytoplasmic fractions of neuroblastoma cells following DHODH knockdown (SQLE exposure time: 4000 ms). *G*, enhanced detection of nuclear SQLE using extended exposure time (15,000 ms), normalized to Histone H3. *H*, western blot analysis of SQLE in nuclear and cytoplasmic fractions following Regorafenib treatment (SQLE exposure time: 4000 ms). *I*, detection of increased nuclear SQLE after Regorafenib treatment using extended exposure time (15,000 ms), normalized to Histone H3.

Previously, we hypothesized that DHODH inhibition would lead to reduced cholesterol production, thereby decreasing lipid droplet formation. Our recent findings confirmed that lipid droplet production indeed decreases following DHODH inhibition (Fig. 6, *D*–*F*). To further validate this, we performed a cholesterol assay, which showed a significant reduction in cholesterol levels after DHODH inhibition (Fig. 7, *A* and *B*).

Considering the observed reduction in lipid droplet formation (Fig. 6, *D*–*F*), we explored whether the composition of lipid droplet-associated proteins was also altered. By comparing our proteomic data with previously reported lipid droplet proteins (48), we identified several commonly expressed proteins and analyzed their expression levels changes. Among these, SQLE, a key enzyme in the mevalonate pathway, showed significant differential expression and was also detected in the lipid droplet fraction. Immunocytochemical analysis further confirmed that SQLE localized around lipid droplets in neuroblastoma cells (Fig. 7, *C* and *D*). Interestingly, we also detected the nuclear translocation of SQLE in neuroblastoma cells, a phenomenon not previously reported. To examine changes in SQLE subcellular distribution, we isolated nuclear and cytoplasmic fractions using a nuclear extraction buffer and NP-40 (Fig. 7*E*). Given the substantially lower levels of SQLE in the nuclear fraction, the exposure time for the nuclear protein sample (right half of the membrane) was extended to 15 s using the UVP imaging system to enhance detection sensitivity. This approach revealed a clear increase in nuclear SQLE levels following DHODH inhibition, consistent with our previous immunofluorescence observations (Fig. 7, *F*–*I*).

**Fig. 8:**
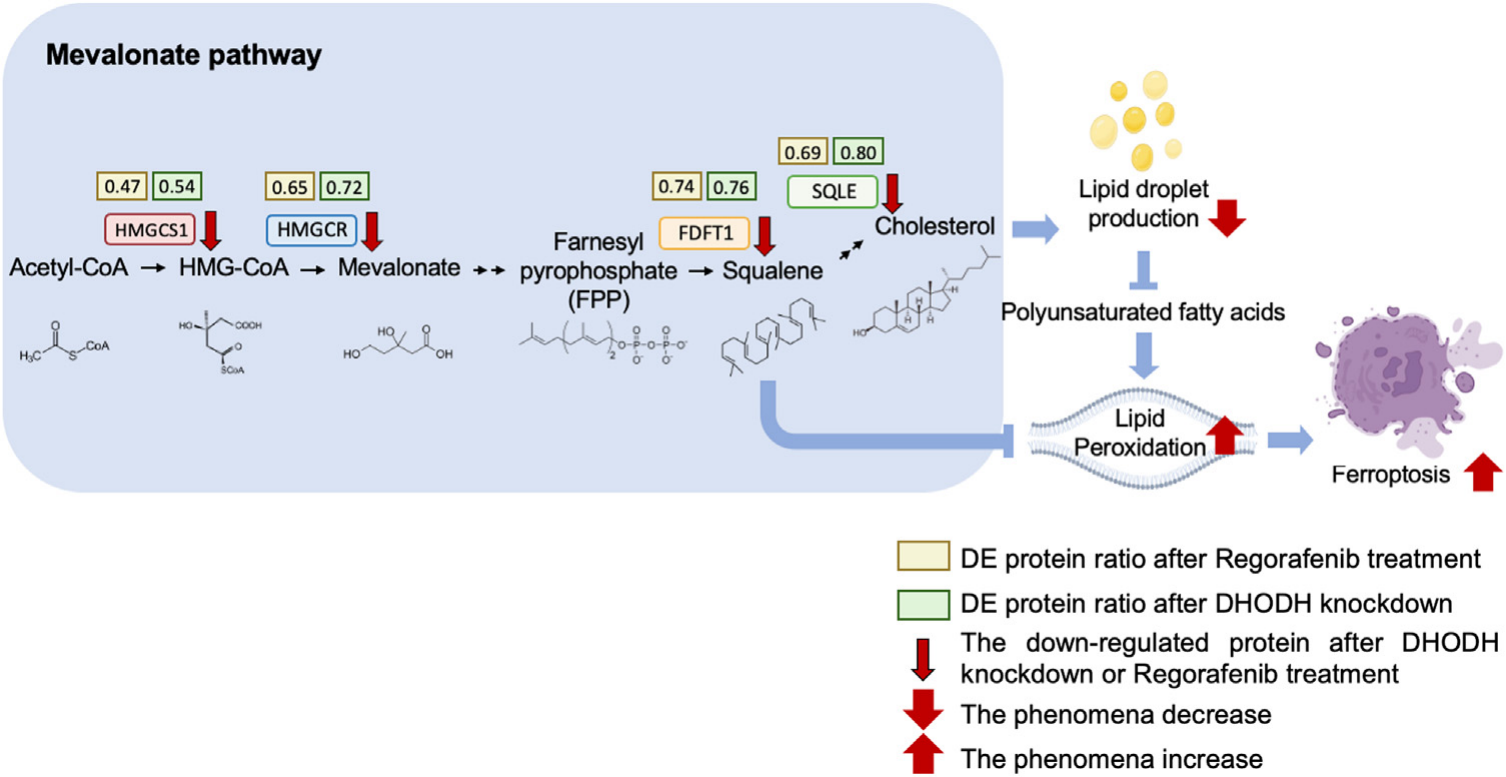
**DHODH inhibition triggers ferroptosis by downregulating key proteins in the mevalonate pathway in neuroblastoma**. Our results demonstrate that DHODH inhibition disrupts the activity of the mevalonate pathway in neuroblastoma cells, leading to ferroptosis. Moreover, DHODH inhibition reduces cholesterol production as a downstream product of the mevalonate pathway, resulting in decreased lipid droplet formation and further promoting ferroptosis.

In summary, our study underscores the potential of Regorafenib as a repurposed treatment for neuroblastoma. By inhibiting DHODH and subsequently disrupting the mevalonate pathway, Regorafenib induces ferroptosis through the reduction of lipid droplet production (Fig. 8). This research not only presents a promising therapeutic avenue for neuroblastoma but also reveals a novel role of DHODH as a key regulator of ferroptosis. Future investigations will focus on elucidating the underlying mechanisms and exploring the clinical applicability of Regorafenib in neuroblastoma therapy to improve treatment outcomes for patients with this challenging malignancy.

## Discussion

Our study highlights the critical role of DHODH, an enzyme essential for the *de novo* pyrimidine synthesis pathway, as a key non-oncogene dependency in neuroblastoma (49). We identified that FDA-approved drug Regorafenib induces ferroptosis in neuroblastoma cells by inhibiting DHODH. Thermal shift assays confirmed that Regorafenib directly affects the structure of DHODH, although its efficacy was slightly lower compared to Leflunomide. We attribute this to the high Regorafenib tends to precipitate during dosing, which may lead to suboptimal drug performance. Nevertheless, Regorafenib remained the top-performing compound among the top six drugs tested. Regorafenib is a multi-kinase inhibitor that targets VEGFRs, PDGFR, RET, RAF kinases, and other signaling molecules (50), suggesting that its impact on neuroblastoma cells may not be solely due to DHODH inhibition. To explore this further, we examined its effects on mitochondrial function, particularly mitochondrial ROS (mtROS) production. As shown in supplemental Fig. S3*J*, Regorafenib treatment resulted in a marked increase in mtROS levels, indicating that oxidative stress may contribute to its cytotoxic effects. Literature evidence also suggests that inhibition of the mevalonate pathway reduces CoQ10 synthesis, which disrupts mitochondrial function by impairing turnover and utilization. This leads to the accumulation of dysfunctional mitochondria, elevated mtROS production, mtDNA release, and diminished mitochondrial membrane potential (51, 52). These findings support a model in which DHODH inhibition may lead to suppression of the mevalonate pathway, thereby promoting mitochondrial ROS accumulation and contributing to the induction of ferroptosis. Enrichment analysis revealed that lipid metabolism was significantly disrupted in both Regorafenib-treated and DHODH knockdown groups. Apart from lipid metabolism, the oxoacid metabolic process was also commonly affected, though it was not explored in depth due to less pronounced enrichment. Despite this, the oxoacid metabolic process is closely associated with cancer progression. Oxoacids are organic acids that contain both a carbonyl group (*e.g.*, ketone or aldehyde) and a carboxyl group, participating in numerous metabolic pathways that are essential for normal cellular function and energy production. These metabolic pathways are crucial for the development and progression of cancer (53).

Cancer cells often exhibit highly active metabolic pathways to support their rapid proliferation, utilizing oxoacid metabolism to generate energy and produce metabolic intermediates. For example, pyruvate, a key end product of glycolysis, enters the citric acid cycle to produce ATP. Cancer cells, which frequently rely on glycolysis for energy production even in the presence of oxygen (known as the Warburg effect), require elevated levels of pyruvate for energy generation and biosynthesis. Thus, reprogramming of the oxoacid metabolic pathways is essential for sustaining cancer cell growth. Understanding how these metabolic pathways are reprogrammed and regulated following DHODH inhibition by Regorafenib is crucial for developing novel therapeutic strategies. Our findings underscore the importance of further investigating the role of the oxoacid metabolic process in neuroblastoma and suggest that targeting DHODH may present a promising approach for disrupting the metabolic networks that sustain cancer cell viability and progression.

Ferroptosis is a type of programmed cell death characterized by iron-dependent lipid peroxidation. Our findings demonstrate that decreased DHODH activity results in the downregulation of key proteins in the mevalonate pathway, including HMGCS1, HMGCR, FDFT-1, and SQLE, leading to reduced lipid droplet production and inducing ferroptosis. Lipids, which include a broad range of fats and oils, serve as crucial sources of energy and essential fatty acids required for human body functions (54). Recent studies have highlighted the connection between lipid metabolism and the development and progression of cancer. In cancer cells, lipid metabolism plays a crucial role in maintaining cellular functions and supporting rapid cell proliferation (55). Previous studies have demonstrated that DHODH can suppress ferroptosis by reducing oxidized lipids through the activity of CoQ10 (56). In contrast, our findings reveal a novel mechanism whereby DHODH inhibition disrupts lipid metabolism and promotes ferroptosis in neuroblastoma. Although DHODH and the mevalonate pathway operate in distinct cellular compartments and participate in separate metabolic functions, they may be metabolically linked *via* CoQ10. Serving as both an electron acceptor for mitochondrial DHODH and a biosynthetic product of the mevalonate pathway, CoQ10 represents a potential metabolic bridge. This dual role suggests a previously unrecognized crosstalk between pyrimidine biosynthesis and isoprenoid production, warranting further investigation. Cholesterol, a major product of the mevalonate pathway, is closely related to tumor growth and can directly influence cancer cell proliferation (54, 57). Excess cholesterol is stored in lipid droplets as cholesterol esters (58). When cholesterol levels are reduced, lipid droplet formation decreases correspondingly. The mevalonate pathway is the primary pathway responsible for cholesterol synthesis (59). Although our findings suggest a correlation between DHODH inhibition, reduced cholesterol levels, and ferroptosis induction, it remains unclear whether the decrease in ferroptosis is directly related to disruption of the mevalonate pathway. To clarify the involvement of DHODH in this process, additional studies are needed to explore its potential link with the mevalonate pathway. Additionally, we hypothesize that the reduction in lipid droplets may lead to the release of PUFAs from these droplets, which could increase the cells’ susceptibility to ferroptosis. Understanding the expression levels and roles of PUFAs in ferroptosis are crucial for elucidating their contribution in this context. Moreover, previous studies have suggested that cell cycle arrest can promote lipid droplet formation, potentially contributing to resistance against ferroptosis. Therefore, it is essential to investigate whether DHODH knockdown affect the cell cycle and how these changes influence lipid droplet formation and ferroptosis sensitivity to ferroptosis. Further research in these areas will provide deeper insights into the mechanisms underlying DHODH inhibition and its potential therapeutic applications in neuroblastoma.

Having confirmed that DHODH inhibition leads to a reduction in lipid droplet production, we sought to understand the underlying molecular mechanisms responsible for this decline. Specifically, we aimed to investigate whether changes in the expression of s lipid droplet associated protein contribute to this outcome. To address this, we compared our proteome data with previously published lipid droplet protein data by Dr Olzmann’s research (48). Through this comparison and subsequent screening, we identified seven differentially expressed lipid droplet-related proteins: ACSL4, FAR1, SQLE, OPA1, PKMYT1, RAB5B, and ARF5. Each of these proteins has distinct roles in lipid metabolism and lipid droplet formation. Acyl-CoA Synthetase Long Chain Family Member 4 (ACSL4) is involved in fatty acid metabolism and is known for activating PUFAs, which are crucial components of lipid droplets (60, 61). Fatty Acyl-CoA Reductase 1 (FAR1) is essential for converting fatty acyl-CoA into fatty alcohols, a key step in lipid biosynthesis (62). Squalene Epoxidase (SQLE) is a key enzyme in the cholesterol biosynthesis pathway, suggesting it may influence lipid droplet formation by modulating cholesterol metabolism. Optic Atrophy 1 (OPA1) is a mitochondrial protein that is associated with mitochondrial dynamics and bioenergetics, indicating a potential connection between mitochondrial function and lipid droplet formation (63, 64). Protein Kinase, Membrane Associated Tyrosine/Threonine 1 (PKMYT1) is a cell cycle regulator, which could influence lipid droplet formation through its effects on cell cycle progression (65). RAB5B and ADP ribosylation factor 5 (ARF5) are small GTPases involved in vesicular trafficking and may play roles in lipid droplet formation and intracellular distribution (66, 67).

SQLE (squalene monooxygenase) is a 64-kDa enzyme that catalyzes the conversion of squalene to 2,3(S)-oxidosqualene, a critical step in cholesterol biosynthesis. The SQLE gene, located on chromosome 8q24.13, spans approximately 23.8 kb and comprises 11 exons. Its expression is tightly regulated at multiple levels: transcriptionally by SREBP2 binding to sterol regulatory elements (SREs) in its promoter; post-transcriptionally by miRNAs and lncRNAs that affect mRNA stability; and post-translationally through cholesterol feedback and squalene-mediated degradation. Despite its well-characterized role in the cytoplasmic cholesterol biosynthesis machinery, no prior studies have reported SQLE localization within the nucleus or explored its functional dynamics in this compartment (68). Among these seven proteins, SQLE emerged as particularly noteworthy due to its role in the mevalonate pathway, where it converts squalene into cholesterol. Our research shows that inhibiting DHODH affects the activity of the mevalonate pathway and found that SQLE is present in lipid droplets, where it influences their formation (48, 69, 70). Thus, it is critical to determine whether the reduction of SQLE following DHODH inhibition is linked to its presence in lipid droplets and to understand SQLE’s specific role in this context.

Interestingly, we also observed nuclear translocation of SQLE in neuroblastoma cells, a phenomenon not previously reported. To further explore the nuclear localization of SQLE, we used the cNLS Mapper tool (https://nls-mapper.iab.keio.ac.jp) to predict potential nuclear localization signals (NLS) within its protein sequence. A putative NLS motif, "DKETGDIKELHAPLTVVADGLFSKFRK," was identified under low-stringency analysis (cut-off score: 5.5), suggesting that SQLE may have the capacity for nuclear import under specific cellular contexts. Classical NLS motifs are typically characterized by sequences such as K(K/R) X(K/R) and clusters of positively charged residues like KKKRK (71). Interestingly, the N-terminus of SQLE contains a positively charged sequence, “RRRRK,” which resembles this canonical NLS pattern. Based on this, we hypothesize that this region may serve as a functional NLS, facilitating SQLE nuclear translocation. Additionally, a recent study by Coates *et al*. (2024) (72) investigating the localization of truncated SQLE reported observations consistent with our findings of SQLE nuclear translocation, although the phenomenon was not explicitly discussed. In their experiments, SQLE was tagged at either the N-terminus with an HA epitope or at the C-terminus with a V5 epitope. Notably, nuclear localization of SQLE was observed in the C-terminal V5-tagged constructs, whereas nuclear presence was less apparent in the N-terminal HA-tagged versions. This differential localization suggests that the N-terminal region of SQLE may contain a NLS or structural elements critical for nuclear import. These findings support our hypothesis that the N-terminus of SQLE plays a role in its nuclear translocation and may have functional implications within the nucleus. Supporting this, Western blot analysis of nuclear and cytoplasmic fractions revealed increased SQLE levels in the nucleus following DHODH inhibition, along with the appearance of multiple additional bands. These bands may indicate structural modifications such as alternative splicing, proteolytic processing, or post-translational modifications that contribute to its nuclear localization and possibly to altered functional roles within the nucleus. Together, these findings suggest that SQLE may have additional, previously uncharacterized regulatory functions beyond its canonical role in cholesterol biosynthesis. Future investigations into the mechanisms driving SQLE’s nuclear translocation and its potential involvement in nuclear lipid metabolism, transcriptional regulation, or cancer-associated metabolic rewiring may uncover novel facets of SQLE function in tumor biology.

By identifying these proteins and understanding their contributions, we can gain deeper insights into how DHODH inhibition leads to reduced lipid droplet production and its downstream effects on ferroptosis and overall cell metabolism. Further experimental validation and functional studies of these proteins will help elucidate the mechanisms driving lipid droplet dynamics and their implications in neuroblastoma therapy.

## Data Availability

All mass spectrometry data have been deposited to ProteomeXchange *via* the jPOSTrepo repository under the dataset identifier PXD054654.

## Supplemental data

This article contains supplemental data.

## Declaration of generative AI and AI-assisted technologies

No AI or AI-assisted technologies were used in this work.

## Conflict of interest

The authors declare that they have no conflicts of interest with the contents of this article.

## Supplemental Data

Supporting Information

Filtered_spectra

Clinical-Checklist
