# Supplementary material for: DHODH Blockade Induces Ferroptosis in Neuroblastoma by Modulating the Mevalonate Pathway
Source: Mol Cell Proteomics. 2025 Jun 11;24(7):101014. doi: 10.1016/j.mcpro.2025.101014 (PMC12275935; doi:10.1016/j.mcpro.2025.101014)
Supplement: Supplementary Figure [file mmc11.docx]

**Supplementary Figure S1-S5**

**DHODH blockade induces ferroptosis in neuroblastoma by modulating the mevalonate pathway**

Jui-Chia Shih^1,#^, Pin-Yu Chen^1,#^, Chuan-Hao Kuo ^1^, Chiao-Hui Hsieh^2^, Hsin-Yi Chang^3^, Hong-Chih Lee^2^, Chen-Hao Huang^4^, Chun-Hua Hsu^5^, Wen-Ming Hsu^6^, Hsuan-Cheng Huang^7*^, Hsueh-Fen Juan^1,2,4,8,9*^

^1^Institute of Molecular and Cellular Biology, National Taiwan University, Taipei, Taiwan

^2^Department of Life Science, National Taiwan University, Taipei, Taiwan

^3^Graduate Institute of Medical Science, National Defense Medical Center, Taipei, Taiwan

^4^Graduate Institute of Biomedical Electronics and Bioinformatics, National Taiwan University, Taipei, Taiwan

^5^Department of Agricultural Chemistry, National Taiwan University, Taipei, Taiwan

^6^ Department of Surgery, National Taiwan University Hospital and National Taiwan University College of Medicine, Taipei, Taiwan

^7^Institute of Biomedical Informatics, National Yang Ming Chiao Tung University, Taipei, Taiwan

^8^enter for Computational and Systems Biology, National Taiwan University, Taipei, Taiwan

^9^Center for Advanced Computing and Imaging in Biomedicine, National Taiwan University, Taipei, Taiwan
^#^Both the authors contributed equally

^*^Correspondence: H.-C.H ([hsuancheng@nycu.edu.tw](mailto:hsuancheng@nycu.edu.tw) ) or H.-F.J. ([yukijuan@ntu.edu.tw](mailto:yukijuan@ntu.edu.tw)).

**Supplementary Figures and Figure legends**


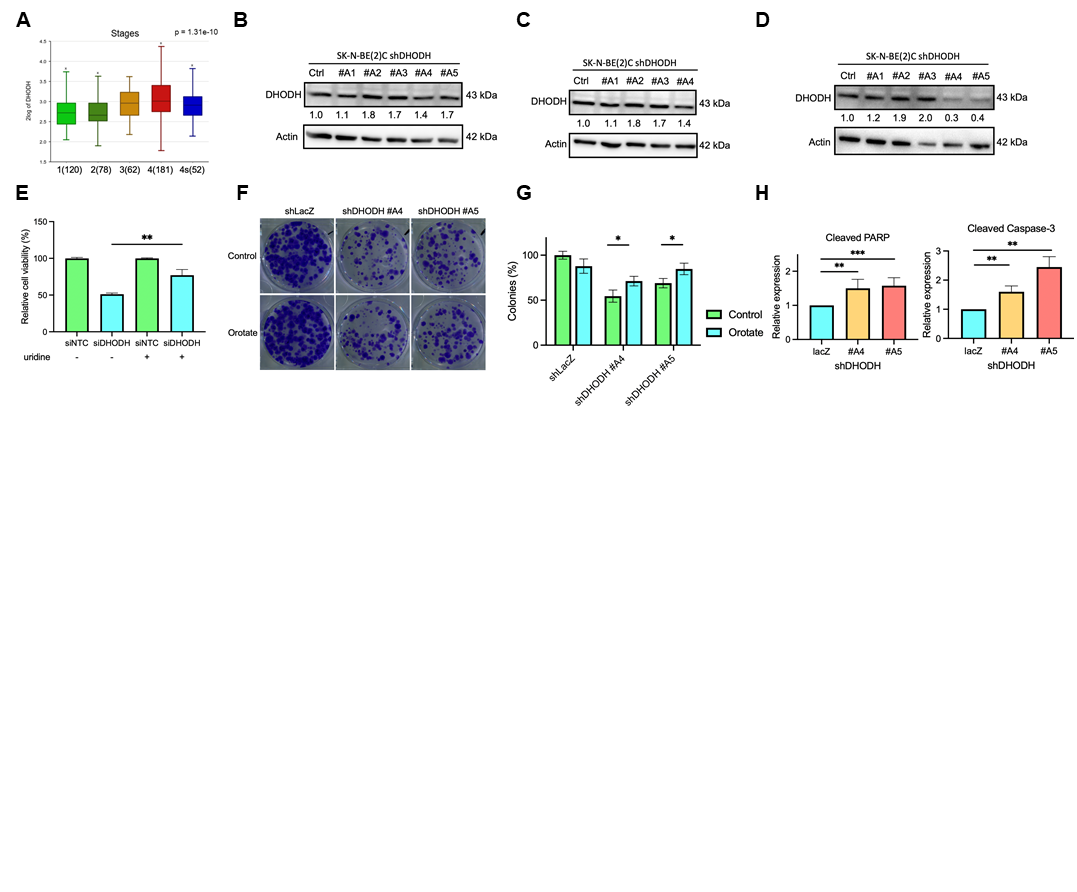


**Supplementary Figure S1. DHODH expression and its impact on neuroblastoma cell viability and apoptosis.** (A) Analysis of DHODH gene expression in neuroblastoma patients, categorized based on MYCN amplification status. (B-D) Western blot analysis displaying DHODH protein levels after 24 hr (B), 48 hr (C), and 72 hr (D) of treatment, with actin used as a loading control. (E) MTS assay evaluating the viability of DHODH knockdown SK-N-BE(2)C cells, with uridine supplementation. (F and G) Colony formation assay showing the number of colonies formed by DHODH knockdown neuroblastoma cells supplemented with orotate after 21 days. (H) Quantification of caspase 3 and Poly (ADP-ribose) polymerase (PARP) levels, with actin serving as the loading control. All quantified data are presented as mean ± SD from three independent experiments. * P < 0.05, ** P < 0.01, *** P < 0.001.

**
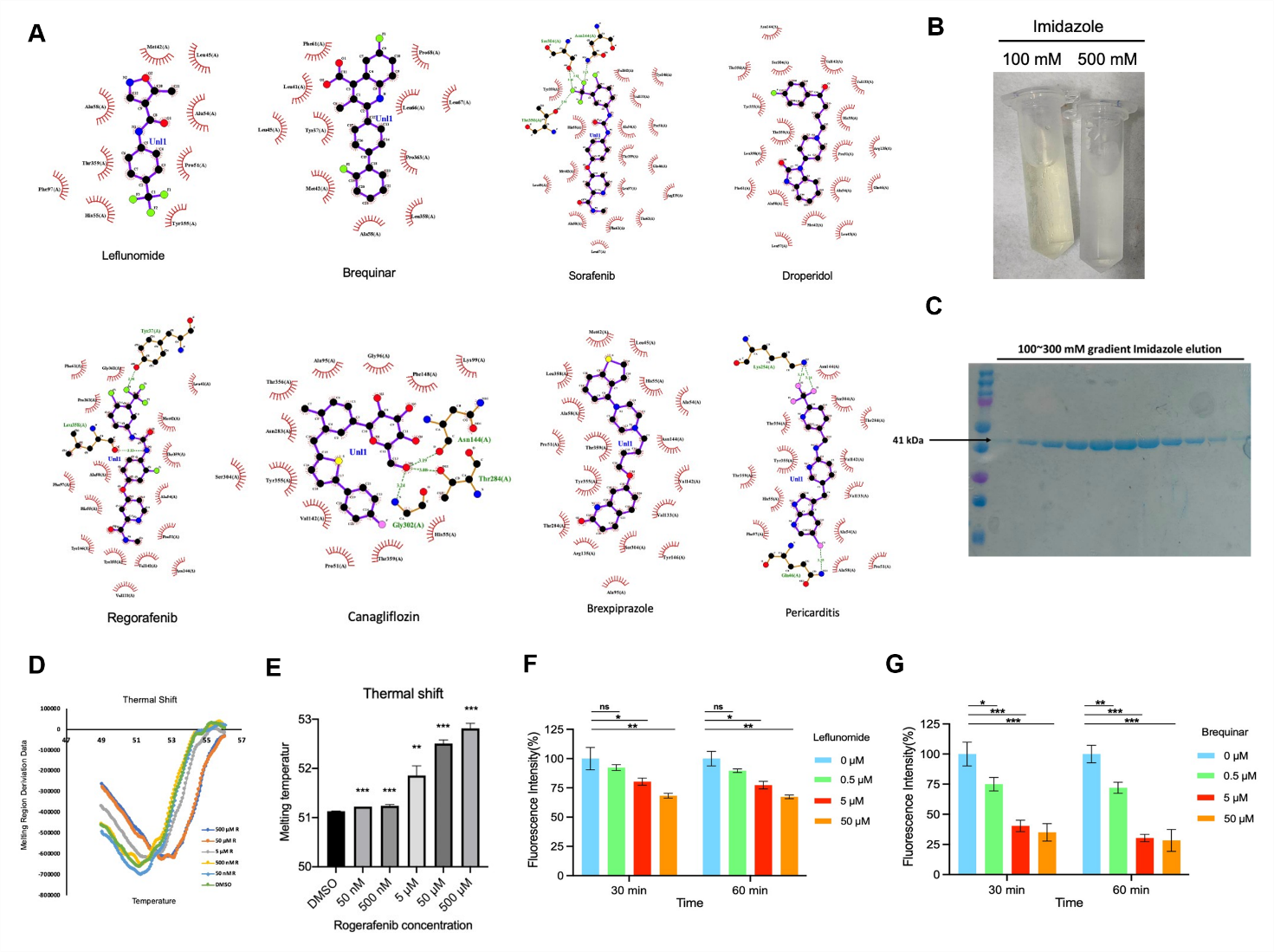
**

**Supplementary Figure S2. Analysis of DHODH binding, elution, and structural stability following Regorafenib treatment.** (A) Binding interactions between ligands and DHODH were analyzed using LigPlot+. (B) DHODH eluted with 100 mM imidazole displayed a distinct yellow color, unlike elution with 500 mM imidazole, which lacked DHODH. (C) SDS-PAGE analysis of DHODH eluted at various imidazole concentrations. (D and E) Thermal shift assay to assess the structural stability of DHODH. DHODH was treated with five concentrations of Regorafenib, and its melting temperature was measured using a qPCR machine. (F and G) Fluorescence-based enzymatic assays measuring DHODH enzyme activity after treatment with Leflunomide and Brequinar. All quantified data are presented as mean ± SD from three independent experiments. * P < 0.05, ** P < 0.01, *** P < 0.001.

**
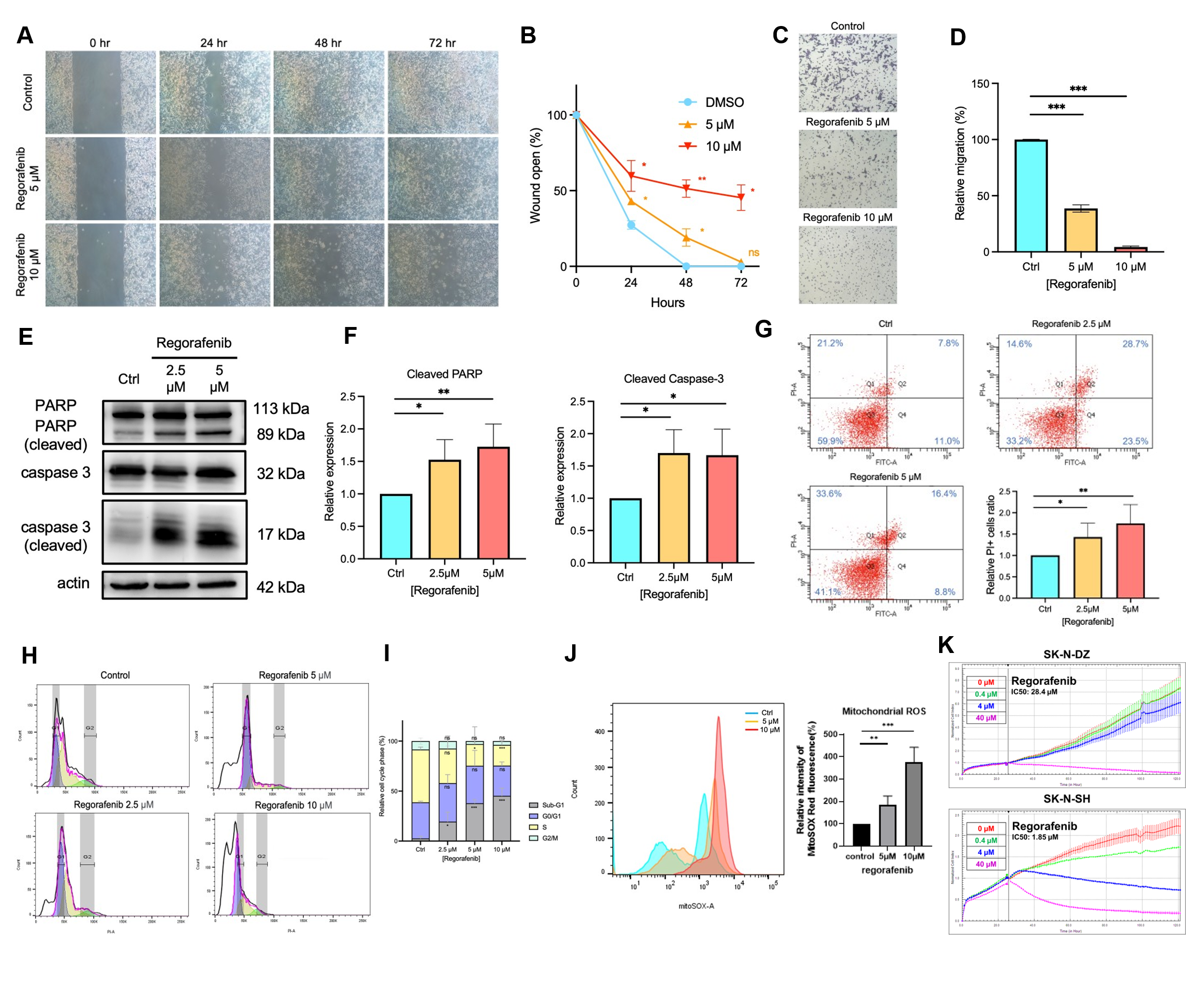
**

**Supplementary Figure S3. Regorafenib inhibits neuroblastoma cell migration, induces apoptosis, and affects cell cycle progression.** (A) Representative images from the wound healing assay show the impact of Regorafenib treatment on SK-N-BE(2)C cells. (B) Quantification of the wound area, represented by the remaining uncovered area, with the scratch size at 0 hours normalized to 100%. (C) Transwell migration assay illustrating the migratory behavior of cells following Regorafenib treatment. (D) Transwell migration assay demonstrating the migration of SK-N-BE(2)C cells following Regorafenib treatment. (E) Western blot analysis depicting the levels and activation status of caspase 3 and Poly (ADP-ribose) polymerase (PARP), with actin as the loading control. (F) Quantification of caspase 3 and Poly (ADP-ribose) polymerase (PARP), normalized to actin. (G) Flow cytometry analysis of neuroblastoma cells after 48 hr of Regorafenib treatment, showing PI-positive signals in the Q1 and Q2 quadrants of the dot plot. (H and I) Cell cycle analysis of SK-N-BE(2)C cells treated with Regorafenib for 48 hr. (J) Quantification of mitochondrial ROS levels following 72 hours of Regorafenib treatment. (K) Cell viability of MYCN-amplified SK-N-DZ and MYCN non-amplified SK-N-SH cells following 72-hour Regorafenib treatment, assessed using a real-time cell analyzer (RTCA). Regorafenib was tested at concentrations of 0 µM, 0.4 µM, 4 µM, and 40 µM. All quantified results are expressed as mean ± SD from three independent experiments. * P < 0.05, ** P < 0.01, *** P < 0.001.

**
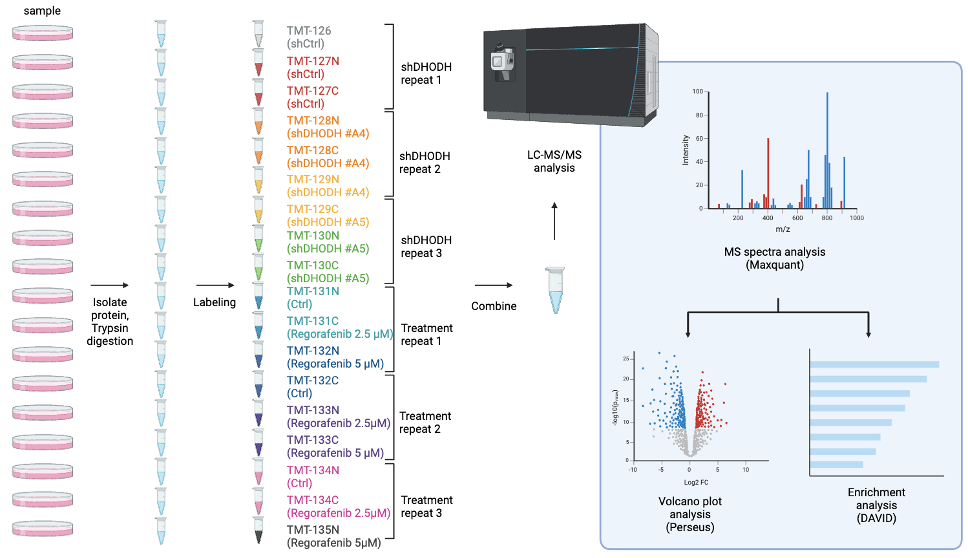
**

**Supplementary Figure S4. Experiment workflow of TMT-based quantitative proteomics.**

**
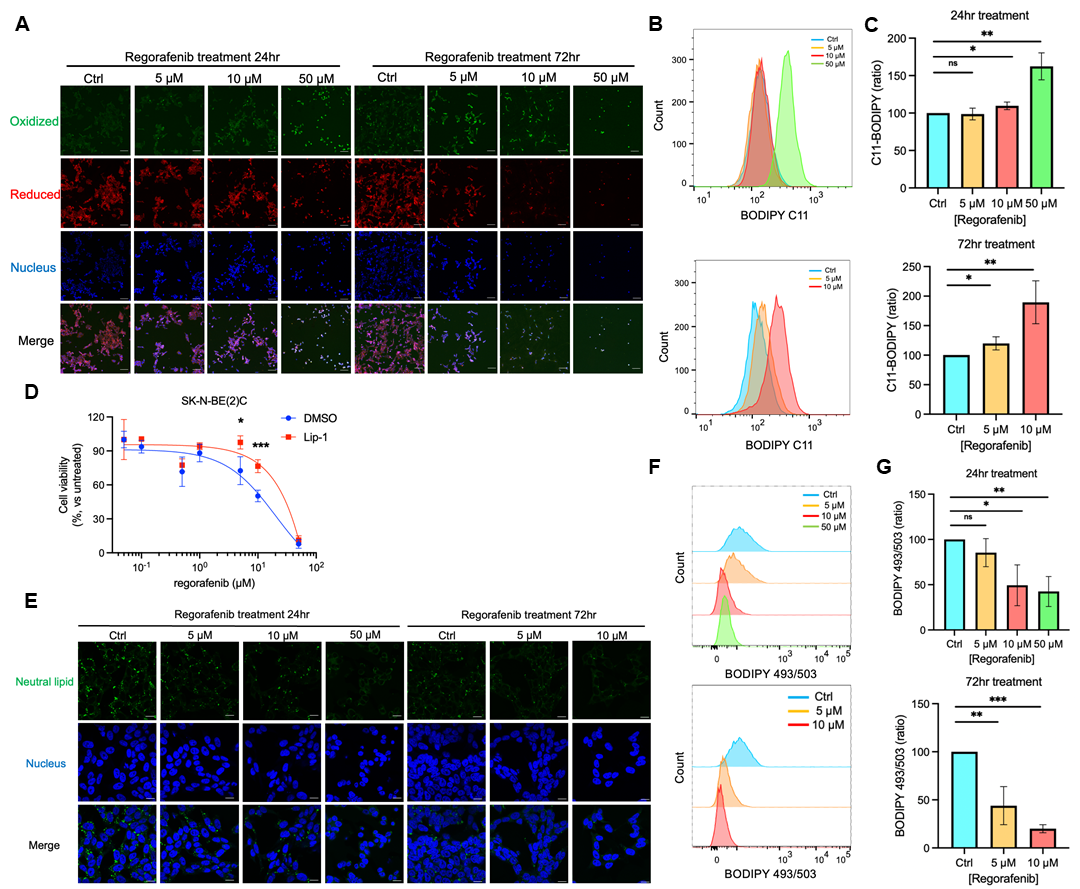
**

**Supplementary Figure S5. Regorafenib-induced lipid peroxidation and neutral lipid alterations in neuroblastoma cells.** (A) Confocal microscopy images of neuroblastoma cells treated with Regorafenib for 24 hr and 72 hr, stained with C11-BODIPY and DAPI. The green signal represents the oxidized form of C11-BODIPY, the red indicates the non-oxidized form, and the blue represents cell nuclei. Scale bar: 100 μm. (B and C) Lipid peroxidation assessed by C11-BODIPY staining. The images show neuroblastoma cells treated with DMSO or Regorafenib at concentrations of 5 μM, 10 μM, or 50 μM for 24 hr (top panel) and 72 hr (bottom panel). Both panels are representative of three independent experiments. (D) Neuroblastoma cell viability after 72 hr of treatment with Regorafenib alone or in combination with the ferroptosis inhibitor liproxstatin-1 (Lip-1; 0.5 μM). (E) Confocal imaging of neuroblastoma cells treated with Regorafenib for 48 hr and 72 hr, stained with BODIPY 493/503 and DAPI. Green represents neutral lipid, and blue indicates nuclei. Scale bar: 20 μm. (F) Neutral lipid levels analyzed by BODIPY 493/503 staining. Images display neuroblastoma cells treated with DMSO, 5 μM Regorafenib, or 10 μM Regorafenib for 24 hr and 72 hr. These images are representative of three independent experiments. (G) Quantified fluorescence intensity of BODIPY 493/503 from three independent experiments. All quantified results are expressed as mean ± SD from three independent experiments. * P < 0.05, ** P < 0.01, *** P < 0.001.
