# Supplementary material for: DHODH Blockade Induces Ferroptosis in Neuroblastoma by Modulating the Mevalonate Pathway
Source: Mol Cell Proteomics. 2025 Jun 11;24(7):101014. doi: 10.1016/j.mcpro.2025.101014 (PMC12275935; doi:10.1016/j.mcpro.2025.101014)
Supplement: Supporting Information [file mmc12.docx]

**Supplementary Data 1. Raw data of PARP, PARP (cleaved), Cleaved caspase 3, Caspase 3 and Actin expression in SK-N-BE(2)C neuroblastoma cell line.**

These data correspond to Figure 2i.

**Supplementary Data 2. Raw data of HMGCS1, HMGCR, SQLE, FDFT-1 and Actin expression in SK-N-BE(2)C neuroblastoma cell line.**

These data correspond to Figure 5i.
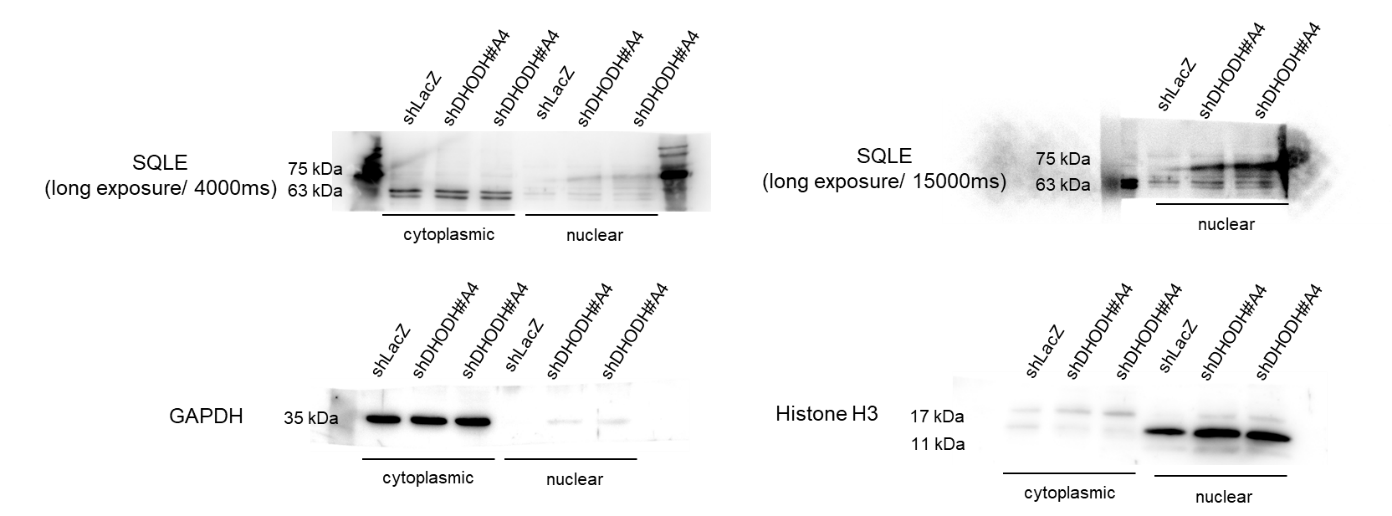


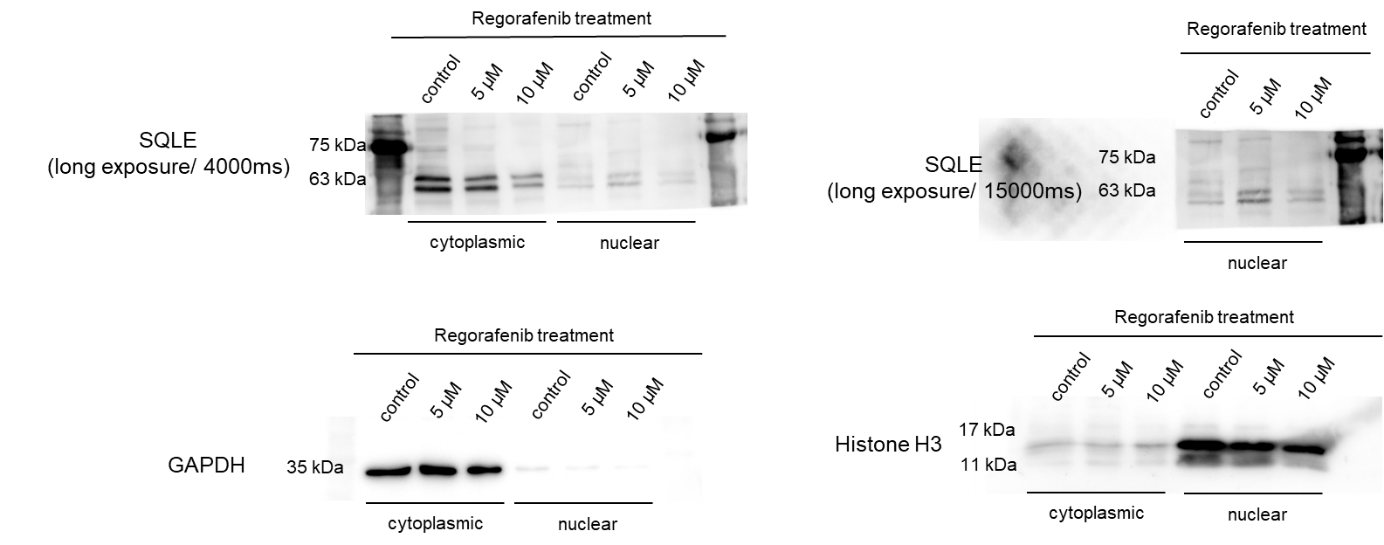


**Supplementary Data 3. Raw data of SQLE, SQLE (long exposure) GAPDH and Histone H3 expression in SK-N-BE(2)C neuroblastoma cell line.**

These data correspond to Figure 7f, g, h, i

**Supplementary Data 4. Raw data of DHODH and Actin expression in SK-N-BE(2)C neuroblastoma cell line.**

These data correspond to Supplementary Fig. 1b, c, d.

**Supplementary Data 5. Raw data of PARP, PARP (cleaved), Cleaved caspase 3, Caspase 3 and Actin expression in SK-N-BE(2)C neuroblastoma cell line.**

These data correspond to Supplementary Fig. 3e.
