# Supplementary material for: DHODH Blockade Induces Ferroptosis in Neuroblastoma by Modulating the Mevalonate Pathway
Source: Mol Cell Proteomics. 2025 Jun 11;24(7):101014. doi: 10.1016/j.mcpro.2025.101014 (PMC12275935; doi:10.1016/j.mcpro.2025.101014)
Supplement: Filtered_spectra [file mmc13.pdf]

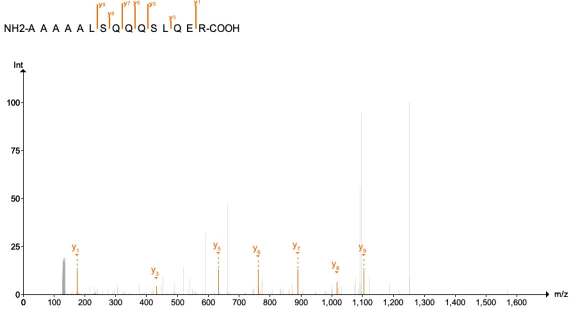

NH2-A A A A A L S Q Q Q S L Q E R-COOH
y9 y8 y7 y6 y5 y3 y1
Int
100
75
50
25
0
y1 y3 y5 y6 y7 y8 y9
0 100 200 300 400 500 600 700 800 900 1,000 1,100 1,200 1,300 1,400 1,500 1,600
m/z

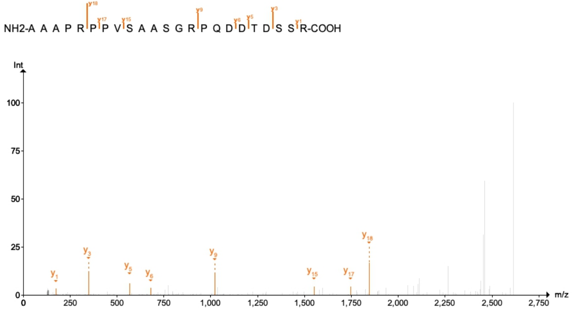
NH2-A A A P R P P V S A A S G R P Q D D T D S S R-COOH
y18
y17
y15
y9
y6
y5
y3
y1
Int
100
75
50
25
0
250
500
750
1,000
1,250
1,500
1,750
2,000
2,250
2,500
2,750
m/z
y1
y3
y5
y6
y9
y15
y17
y18

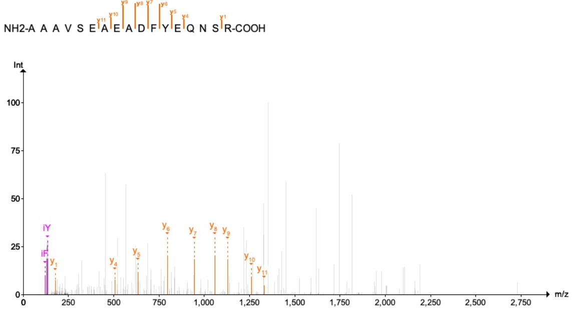

NH2-A A A V S E A E A D F Y E Q N S R-COOH
y11 y10 y9 y8 y7 y6 y5 y4 y1
Int
100
75
50
25
0
iY
iF
y1 y4 y5 y6 y7 y8 y9 y10 y11
0 250 500 750 1,000 1,250 1,500 1,750 2,000 2,250 2,500 2,750
m/z

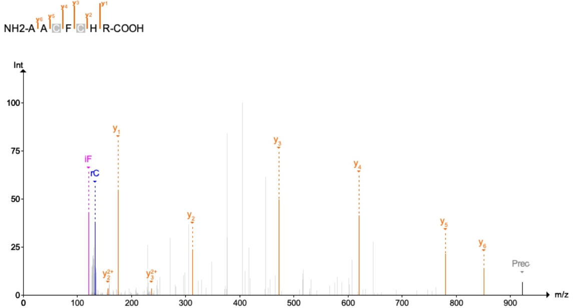

NH2-A A C F C H R-COOH
y6 y5 y4 y3 y2 y1
Int
m/z
iF
rC
y_1
y_2^{2+}
y_3^{2+}
y_2
y_3
y_4
y_5
y_6
Prec
0
25
50
75
100
0
100
200
300
400
500
600
700
800
900

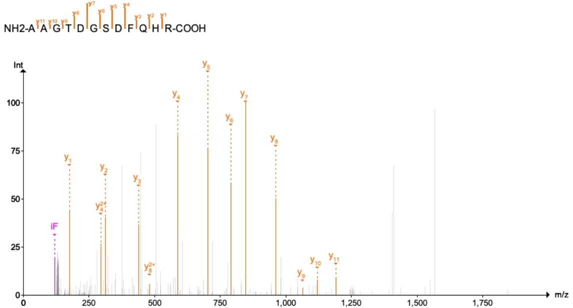
NH2-A A G T D G S D F Q H R-COOH
y11 y10 y9 y8 y7 y6 y5 y4 y3 y2 y1
Int
m/z
iF
y1
y2
y4 2+
y3
y8 2+
y4
y5
y6
y7
y8
y9
y10
y11
0
25
50
75
100
250
500
750
1,000
1,250
1,500
1,750

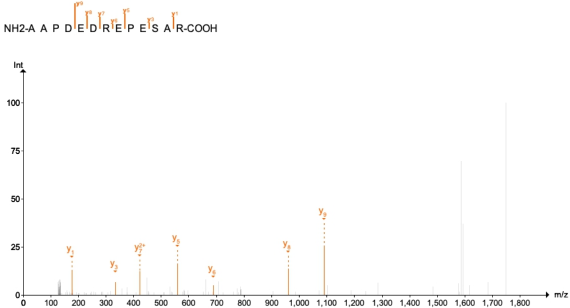
NH2-A A P D E D R E P E S A R-COOH
y9
y8
y7
y6
y5
y3
y1
Int
100
75
50
25
0
m/z
0
100
200
300
400
500
600
700
800
900
1,000
1,100
1,200
1,300
1,400
1,500
1,600
1,700
1,800
y1
y3
y7 2+
y5
y6
y8
y9

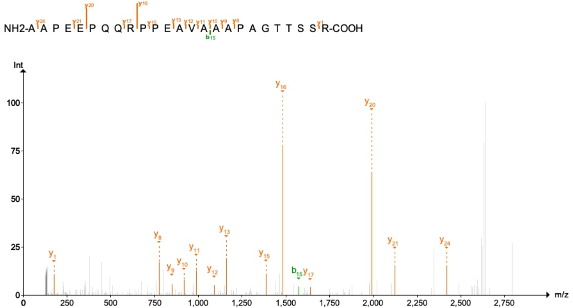

NH2-A A P E E P Q Q R P P E A V A A A P A G T T S S R-COOH
y24 y21 y20 y17 y16 y15 y13 y12 y11 y10 y9 y8 y1
b15
Int
100
75
50
25
0
y1 y8 y9 y10 y11 y12 y13 y15 y16 b15 y17 y20 y21 y24
0 250 500 750 1,000 1,250 1,500 1,750 2,000 2,250 2,500 2,750
m/z

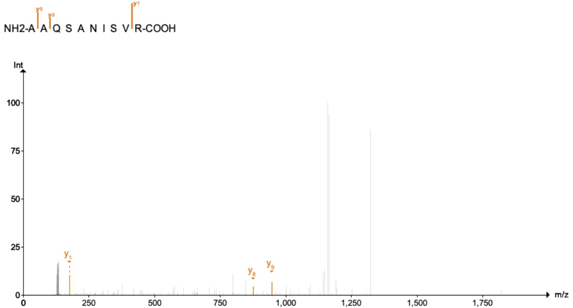
NH2-A A Q S A N I S V R-COOH
y9
y8
y1
Int
100
75
50
25
0
y1
y8
y9
0
250
500
750
1,000
1,250
1,500
1,750
m/z

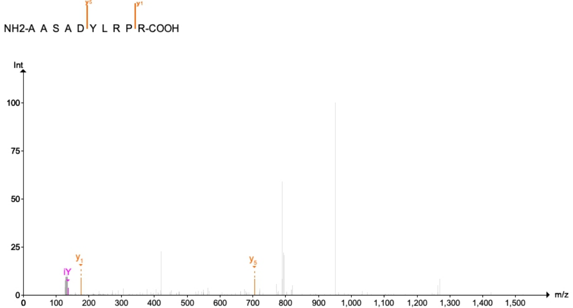
NH2-A A S A D Y L R P R-COOH
y5
y1
Int
100
75
50
25
0
iY
0
100
200
300
400
500
600
700
800
900
1,000
1,100
1,200
1,300
1,400
1,500
m/z

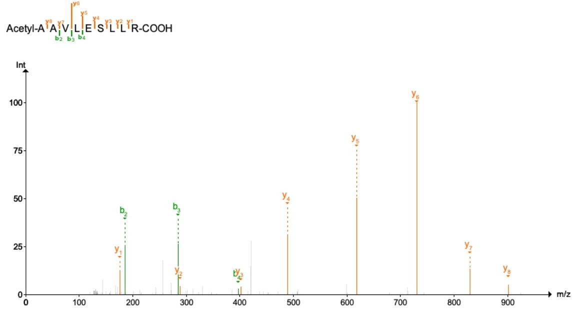

Acetyl-AAVLESLLR-COOH
Int
m/z
y1
y2
y3
y4
y5
y6
y7
y8
b2
b3
b4

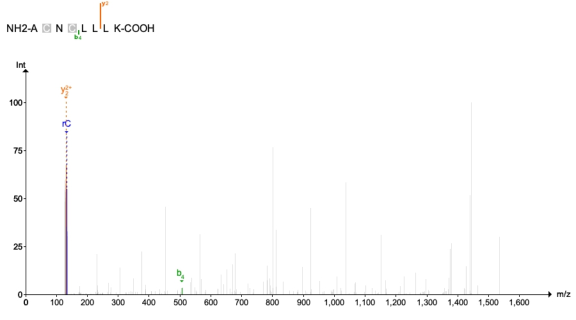

NH2-A C N C L L L K-COOH
y2
b4
Int
m/z
y2 2+
rC
b4
100
75
50
25
0
0
100
200
300
400
500
600
700
800
900
1,000
1,100
1,200
1,300
1,400
1,500
1,600

NH2-A C S V I F P L I Y S E M K-COOH

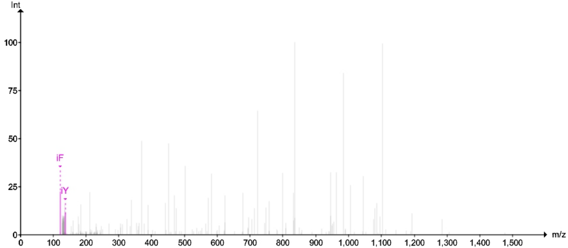

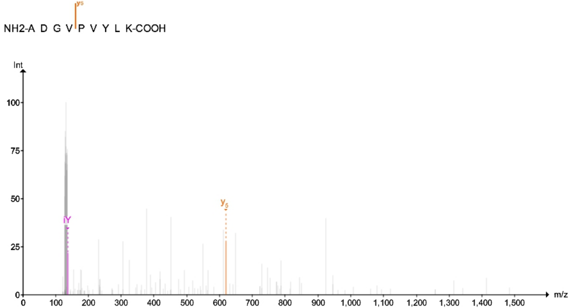

NH2-A D G V P V Y L K-COOH
y5
Int
100
75
50
25
0
iY
y5
m/z
0
100
200
300
400
500
600
700
800
900
1,000
1,100
1,200
1,300
1,400
1,500

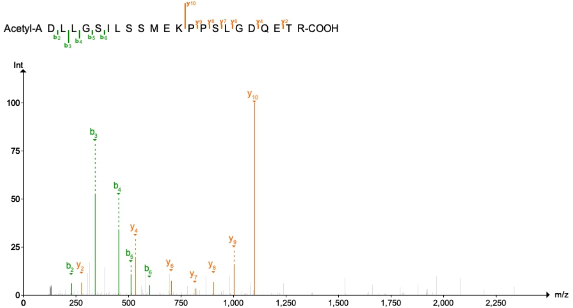

Acetyl-A D L L G S I L S S M E K P P S L G D Q E T R-COOH
b2 b3 b4 b5 b6
y10 y9 y8 y7 y6 y4 y2
Int
100
75
50
25
0
b3
b4
y4
b5
b2
y2
b6
y6
y7
y8
y9
y10
0
250
500
750
1,000
1,250
1,500
1,750
2,000
2,250
m/z

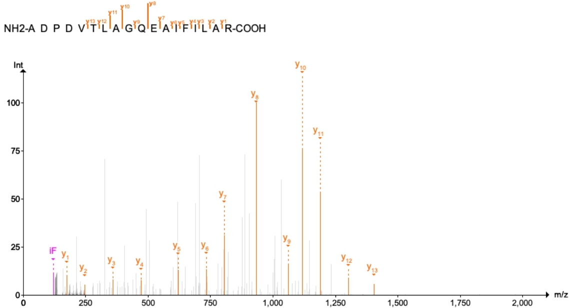
NH2-A D P D V T L A G Q E A I F I L A R-COOH
y13 y12 y11 y10 y9 y8 y7 y6 y5 y4 y3 y2 y1
Int
100
75
50
25
0
iF
y1
y2
y3
y4
y5
y6
y7
y8
y9
y10
y11
y12
y13
0
250
500
750
1,000
1,250
1,500
1,750
2,000
m/z

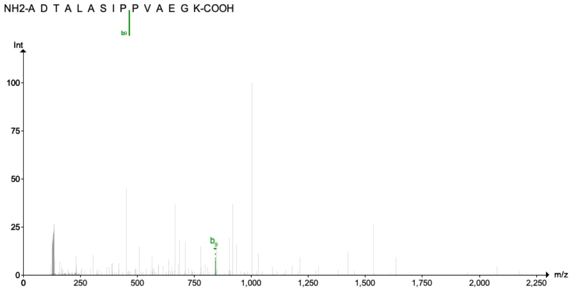
NH2-A D T A L A S I P P V A E G K-COOH
b9
Int
100
75
50
25
0
b9
0
250
500
750
1,000
1,250
1,500
1,750
2,000
2,250
m/z

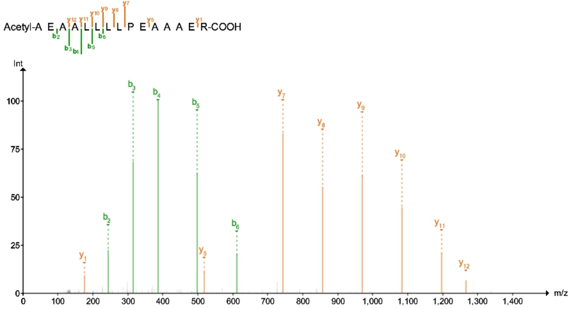

Acetyl-A E A A L L L L P E A A A E R-COOH
b2
b3
b4
b5
b6
y12
y11
y10
y9
y8
y7
y5
y1
Int
m/z

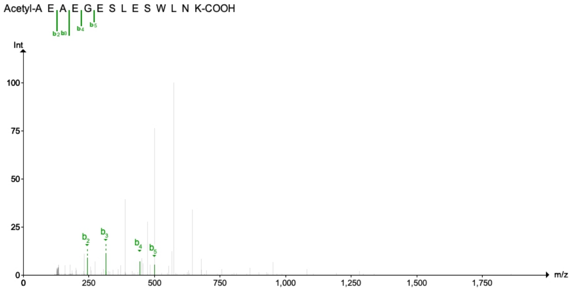

Acetyl-A E A E G E S L E S W L N K-COOH
b2 b3 b4 b5
Int
100
75
50
25
0
b2
b3
b4
b5
0
250
500
750
1,000
1,250
1,500
1,750
m/z

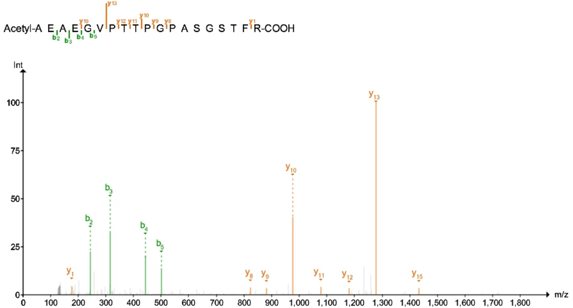

Acetyl-A E A E G V P T T P G P A S G S T F R-COOH
b2
b3
b4
b5
y15
y13
y12
y11
y10
y9
y8
y1
Int
m/z
0
25
50
75
100
0
100
200
300
400
500
600
700
800
900
1,000
1,100
1,200
1,300
1,400
1,500
1,600
1,700
1,800

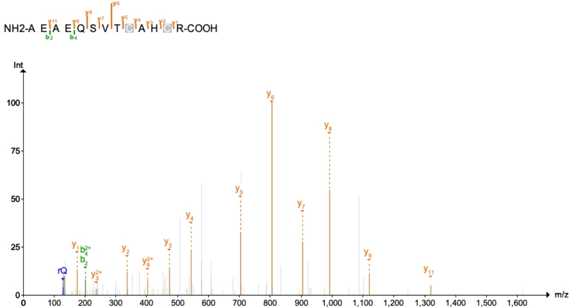
NH2-A E A E Q S V T C A H C R-COOH
Int
m/z
y6
y8
y5
y7
y4
y9
y11
y1
b4 2+
b2
y2
y6 2+
y3
y3 2+
rQ

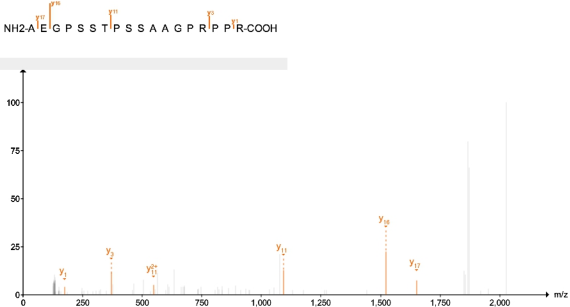
NH2-A E G P S S T P S S A A G P R P P R-COOH
y17
y16
y11
y3
y1
100
75
50
25
0
0
250
500
750
1,000
1,250
1,500
1,750
2,000
m/z
y1
y3
y11 2+
y11
y16
y17

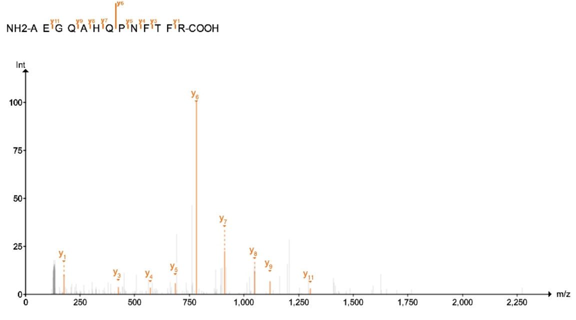

NH2-A E G Q A H Q P N F T F R-COOH
y11
y9
y8
y7
y6
y5
y4
y3
y1
Int
m/z
0
25
50
75
100
250
500
750
1,000
1,250
1,500
1,750
2,000
2,250

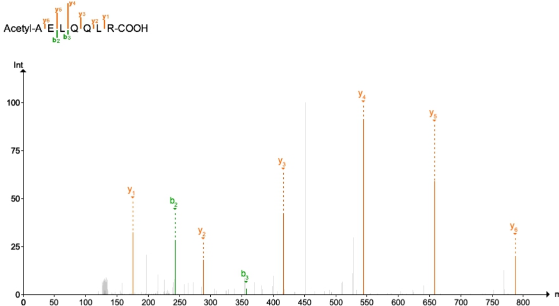
Acetyl-A E L Q Q L R-COOH
y6 y5 y4 y3 y2 y1
b2 b3
Int
m/z
y1
b2
y2
b3
y3
y4
y5
y6
0
25
50
75
100
0 50 100 150 200 250 300 350 400 450 500 550 600 650 700 750 800

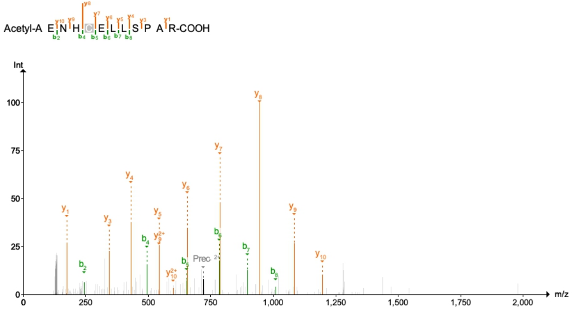

Acetyl-A E N H C E L L S P A R-COOH
Int
m/z

NH2-A E S S F S E E E E E K-COOH

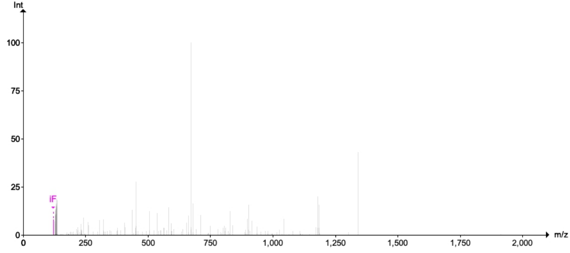

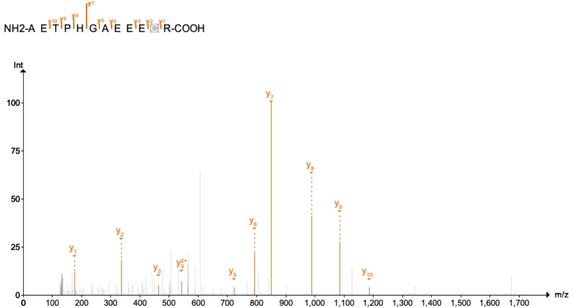
NH2-A E T P H G A E E E C R-COOH
y10 y9 y8 y7 y6 y5 y3 y2 y1
Int
m/z
y1
y2
y3
y9 2+
y5
y6
y7
y8
y9
y10

NH2-A F D Q G A D A I Y D H I N E G K-COOH

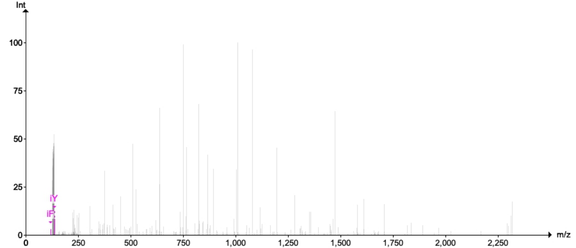

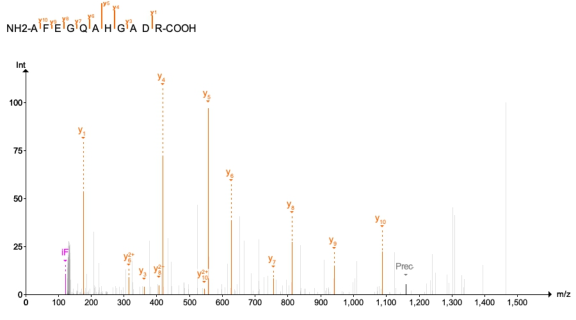
NH2-A F E G Q A H G A D R-COOH
y10 y9 y8 y7 y6 y5 y4 y3 y1
Int
m/z
y4
y5
y1
y6
y8
y10
y9
y7
y6 2+
y3
y8 2+
y10 2+
iF
Prec
0
25
50
75
100
0
100
200
300
400
500
600
700
800
900
1,000
1,100
1,200
1,300
1,400
1,500

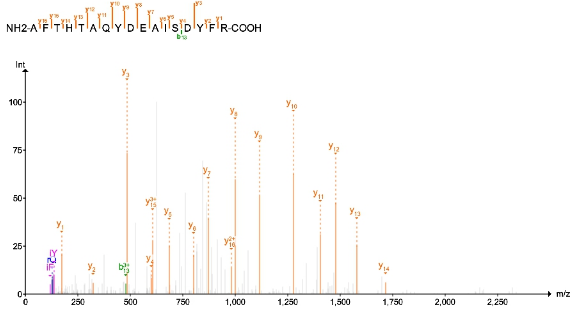

NH2-AFTHTAQYDEAISDYFR-COOH
y16 y15 y14 y13 y12 y11 y10 y9 y8 y7 y6 y5 y4 y3 y2 y1
b13
Int
m/z
y1
y2
b13 3+
y3
y4
y15 3+
y5
y6
y7
y16 2+
y8
y9
y10
y11
y12
y13
y14
iY
iF
100
75
50
25
0
250
500
750
1,000
1,250
1,500
1,750
2,000
2,250

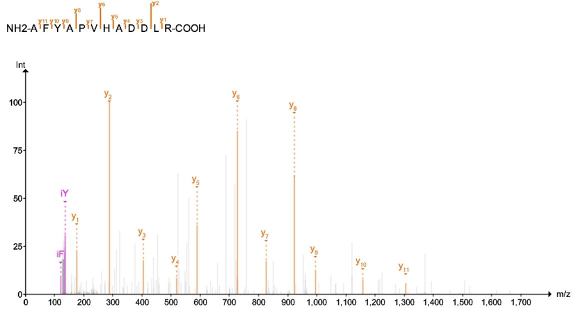

NH2-A F Y A P V H A D D L R-COOH
y11 y10 y9 y8 y7 y6 y5 y4 y3 y2 y1
Int
m/z
y2
y6
y8
y5
iY
y1
y3
y4
y7
y9
y10
y11
iF
0 100 200 300 400 500 600 700 800 900 1,000 1,100 1,200 1,300 1,400 1,500 1,600 1,700
0 25 50 75 100

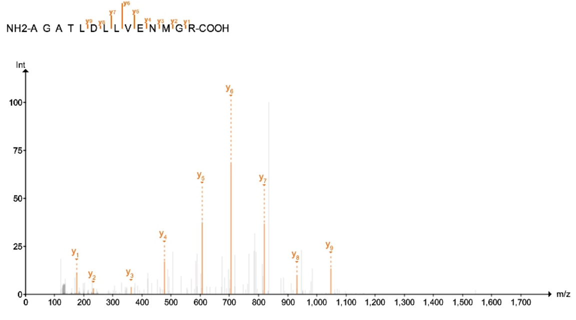

NH2-A G A T L D L L V E N M G R-COOH
y9 y8 y7 y6 y5 y4 y3 y2 y1
Int
100
75
50
25
0
m/z
0 100 200 300 400 500 600 700 800 900 1,000 1,100 1,200 1,300 1,400 1,500 1,600 1,700

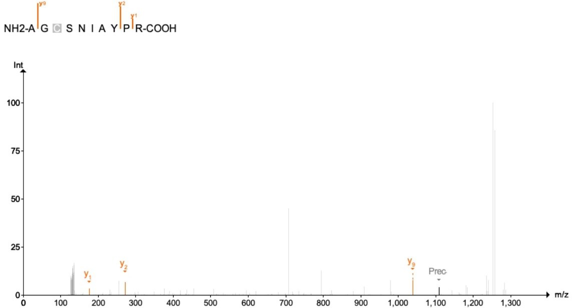

NH2-A G C S N I A Y P R-COOH
y9
y2
y1
Int
100
75
50
25
0
y1
y2
y9
Prec
0
100
200
300
400
500
600
700
800
900
1,000
1,100
1,200
1,300
m/z

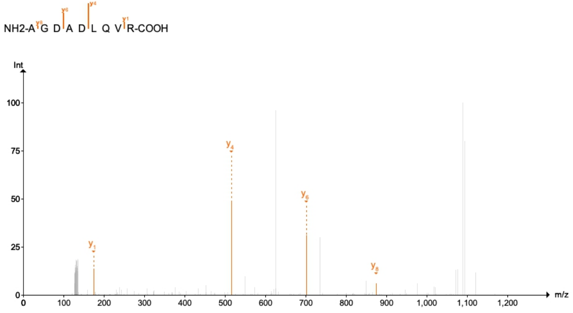
NH2-A G D A D L Q V R-COOH
y8
y6
y4
y1
Int
100
75
50
25
0
y4
y6
y1
y8
0
100
200
300
400
500
600
700
800
900
1,000
1,100
1,200
m/z

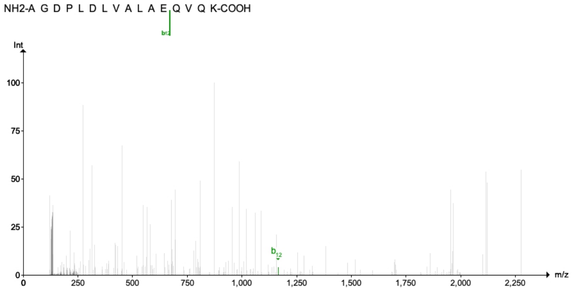
NH2-A G D P L D L V A L A E Q V Q K-COOH
b12
Int
100
75
50
25
0
0
250
500
750
1,000
1,250
1,500
1,750
2,000
2,250
m/z
b12

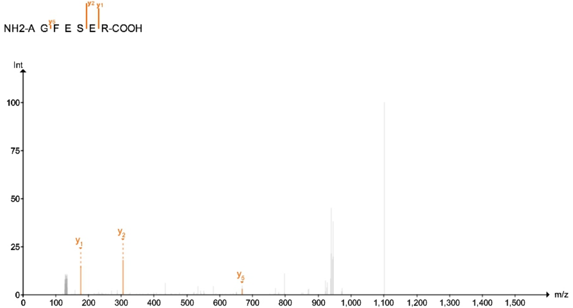

NH2-A G F E S E R-COOH
y5
y2
y1
Int
100
75
50
25
0
y_1
y_2
y_5
0
100
200
300
400
500
600
700
800
900
1,000
1,100
1,200
1,300
1,400
1,500
m/z

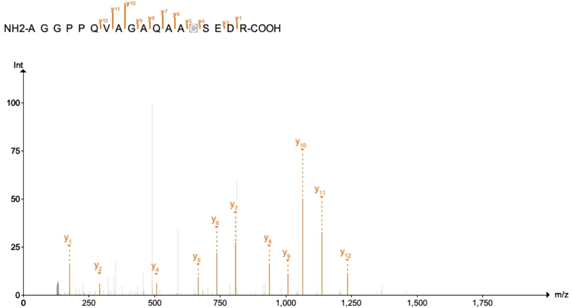

NH2-A G G P P Q V A G A Q A A C S E D R-COOH
y12 y11 y10 y9 y8 y7 y6 y5 y4 y2 y1
Int
100
75
50
25
0
y1
y2
y4
y5
y6
y7
y8
y9
y10
y11
y12
0
250
500
750
1,000
1,250
1,500
1,750
m/z

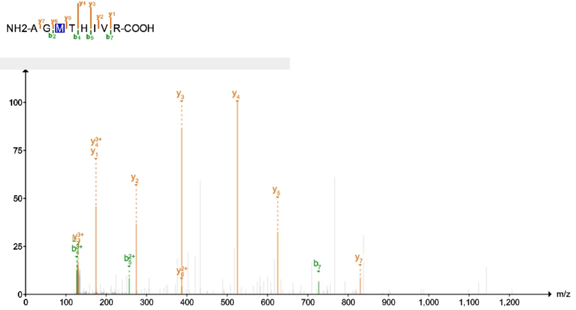
NH2-AGMTHIVR-COOH
y7 y6 y5 y4 y3 y2 y1
b2 b4 b5 b7
y3
y4
y4 3+
y1
y2
y5
y2 3+
b4 2+
b5 2+
y6 2+
b7
y7
0
25
50
75
100
m/z
0 100 200 300 400 500 600 700 800 900 1,000 1,100 1,200

NH2-A G N E Y L I F Q K-COOH

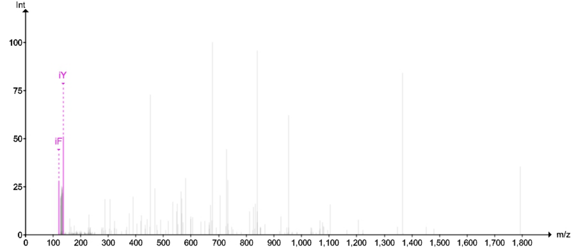

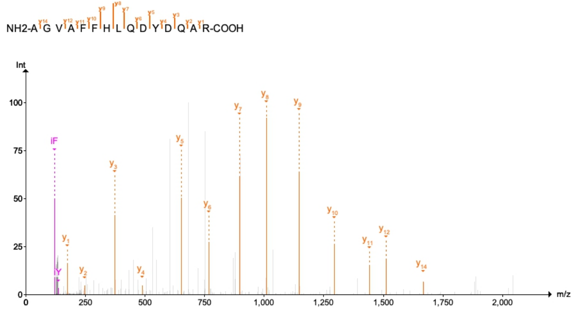

NH2-AGVAFFHLQDYDQAR-COOH
Int
m/z
iF
Y
y1
y2
y3
y4
y5
y6
y7
y8
y9
y10
y11
y12
y14

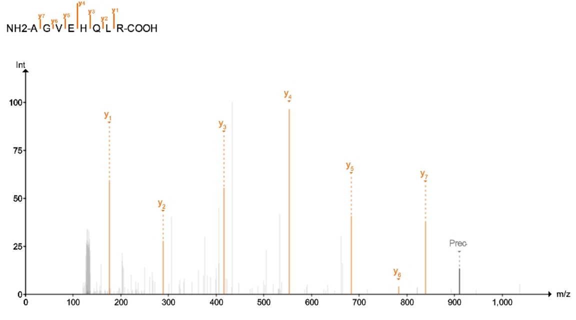
NH2-A G V E H Q L R-COOH
y7 y6 y5 y4 y3 y2 y1
Int
100
75
50
25
0
y1
y2
y3
y4
y5
y6
y7
Prec
0 100 200 300 400 500 600 700 800 900 1,000
m/z

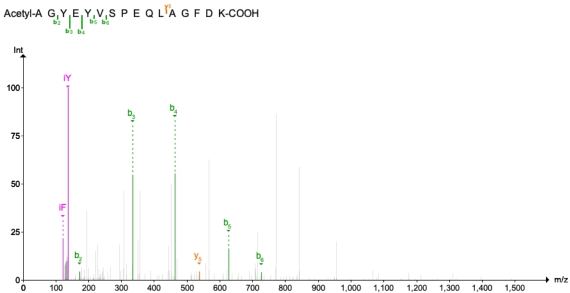
Acetyl-A G Y E Y V S P E Q L A G F D K-COOH
b2
b3
b4
b5
b6
y5
Int
iY
iF
b3
b4
b5
b2
y5
b6
100
75
50
25
0
0
100
200
300
400
500
600
700
800
900
1,000
1,100
1,200
1,300
1,400
1,500
m/z

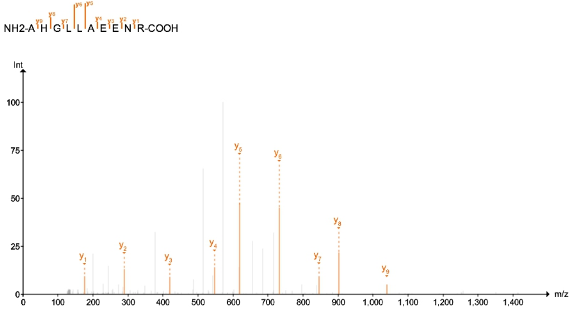

NH2-A H G L L A E E N R-COOH
y9 y8 y7 y6 y5 y4 y3 y2 y1
Int
100
75
50
25
0
y1
y2
y3
y4
y5
y6
y7
y8
y9
0 100 200 300 400 500 600 700 800 900 1,000 1,100 1,200 1,300 1,400
m/z

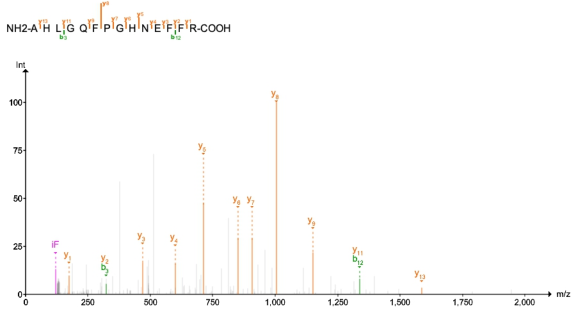

NH2-A H L G Q F P G H N E F F R-COOH
y13 y11 y9 y8 y7 y6 y5 y4 y3 y2 y1
b3 b12
Int
100
75
50
25
0
iF
y1
y2
b3
y3
y4
y5
y6
y7
y8
y9
y11
b12
y13
0
250
500
750
1,000
1,250
1,500
1,750
2,000
m/z

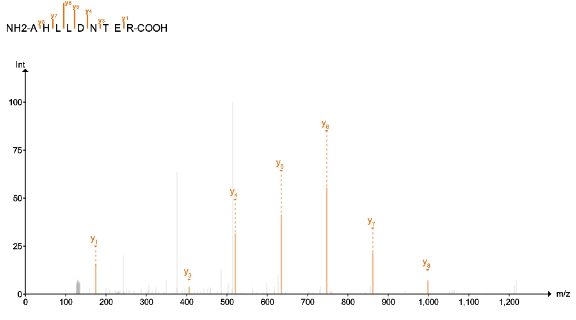
NH2-A H L L D N T E R-COOH
y8 y7 y6 y5 y4 y3 y1
Int
100
75
50
25
0
y1
y3
y4
y5
y6
y7
y8
0 100 200 300 400 500 600 700 800 900 1,000 1,100 1,200
m/z

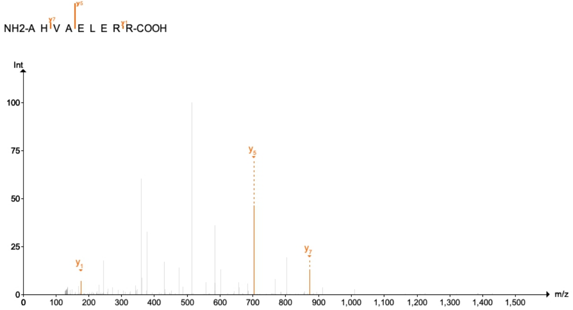

NH2-A H V A E L E R R-COOH
y7
y5
y1
Int
100
75
50
25
0
y1
y5
y7
0
100
200
300
400
500
600
700
800
900
1,000
1,100
1,200
1,300
1,400
1,500
m/z

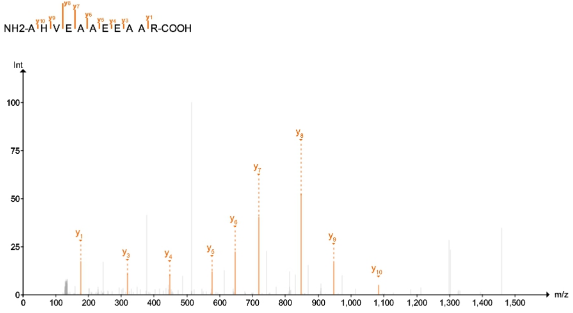

NH2-A H V E A A E E A A R-COOH
y10 y9 y8 y7 y6 y5 y4 y3 y1
Int
100
75
50
25
0
y1
y3
y4
y5
y6
y7
y8
y9
y10
0 100 200 300 400 500 600 700 800 900 1,000 1,100 1,200 1,300 1,400 1,500
m/z

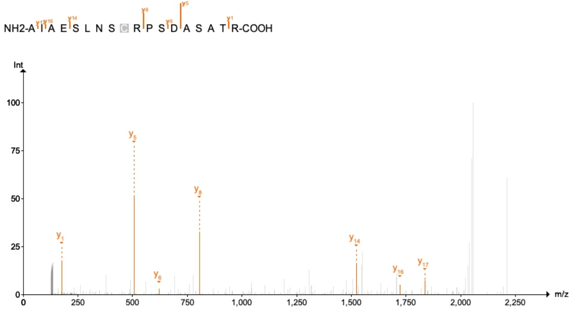

NH2-A I I A E S L N S C R P S D A S A T R-COOH
y17 y16 y14 y8 y6 y5 y1
Int
100
75
50
25
0
y1
y5
y6
y8
y14
y16
y17
0 250 500 750 1,000 1,250 1,500 1,750 2,000 2,250
m/z

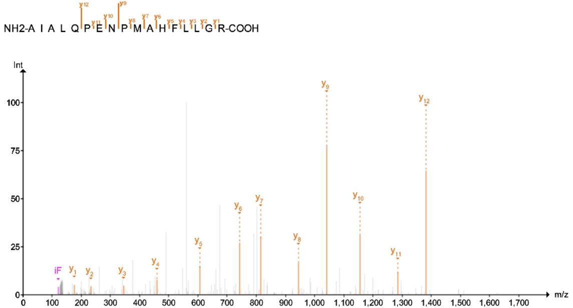

NH2-A I A L Q P E N P M A H F L L G R-COOH
Int
m/z
iF
y1
y2
y3
y4
y5
y6
y7
y8
y9
y10
y11
y12
0
25
50
75
100
0
100
200
300
400
500
600
700
800
900
1,000
1,100
1,200
1,300
1,400
1,500
1,600
1,700

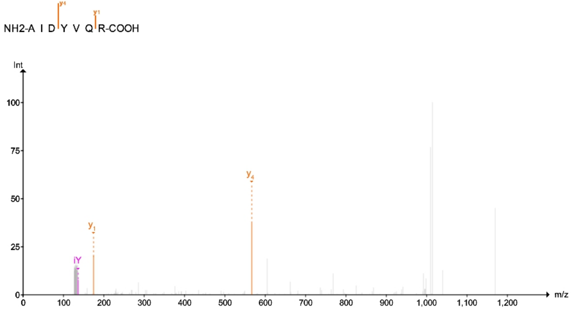

NH2-A I D Y V Q R-COOH
y4
y1
Int
100
75
50
25
0
y4
y1
iY
0
100
200
300
400
500
600
700
800
900
1,000
1,100
1,200
m/z

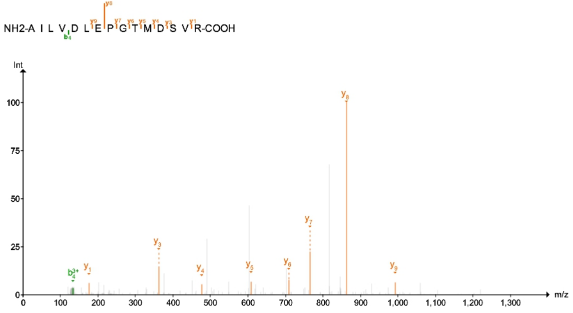

NH2-A I L V D L E P G T M D S V R-COOH
Int
m/z

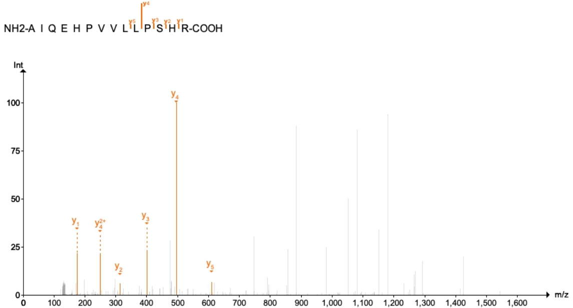

NH2-A I Q E H P V V L L P S H R-COOH
y5
y4
y3
y2
y1
Int
100
75
50
25
0
y1
y4 2+
y2
y3
y4
y5
0
100
200
300
400
500
600
700
800
900
1,000
1,100
1,200
1,300
1,400
1,500
1,600
m/z

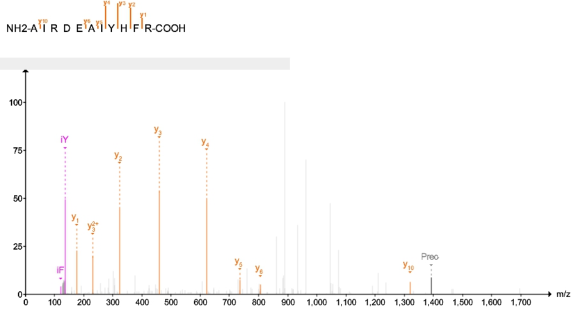

NH2-A I R D E A I Y H F R-COOH
y10
y6
y5
y4
y3
y2
y1
iY
iF
y1
y3 2+
y2
y3
y4
y5
y6
y10
Prec
m/z
0
25
50
75
100
0
100
200
300
400
500
600
700
800
900
1,000
1,100
1,200
1,300
1,400
1,500
1,600
1,700

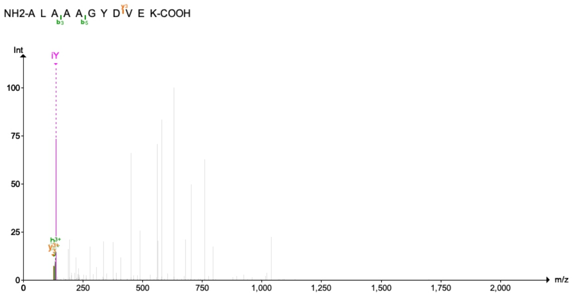
NH2-A L A A A G Y D V E K-COOH
b3
b5
y3
Int
iY
b3+
y3+
m/z
0
25
50
75
100
0
250
500
750
1,000
1,250
1,500
1,750
2,000

NH2-A L A A H P W K-COOH

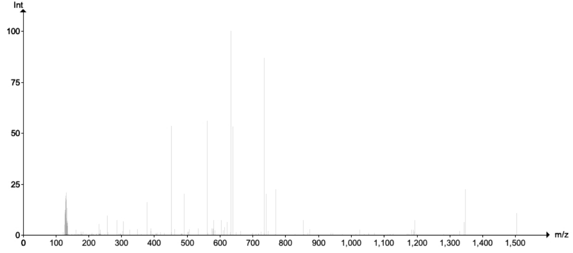

NH2-A L A E Y V V Q Q E G A K-COOH

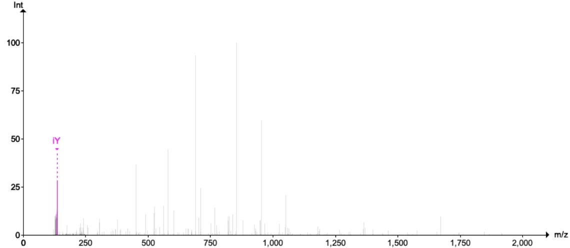

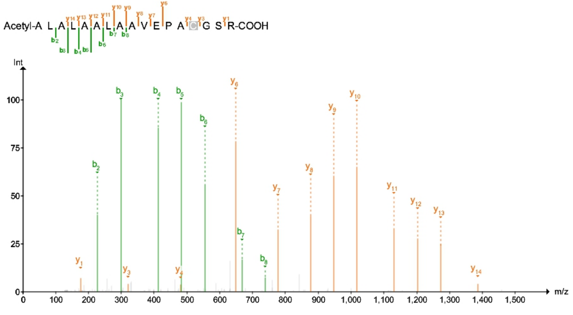

Acetyl-A L A L A A L A A V E P A C G S R-COOH
Int
m/z

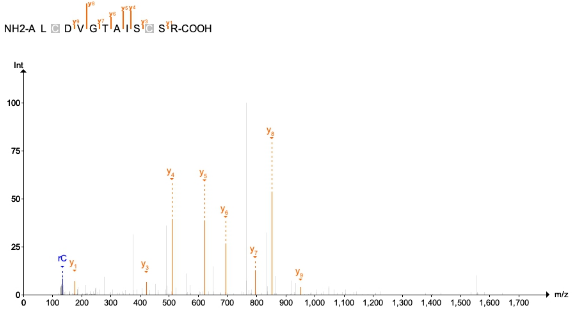
NH2-A L C D V G T A I S C S R-COOH
y9 y8 y7 y6 y5 y4 y3 y1
Int
100
75
50
25
0
rC
y1 y3 y4 y5 y6 y7 y8 y9
0 100 200 300 400 500 600 700 800 900 1,000 1,100 1,200 1,300 1,400 1,500 1,600 1,700
m/z

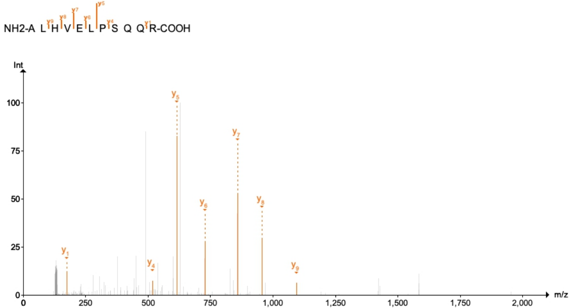

NH2-A L H V E L P S Q Q R-COOH
y9
y8
y7
y6
y5
y4
y1
Int
100
75
50
25
0
y5
y7
y6
y8
y1
y4
y9
250
500
750
1,000
1,250
1,500
1,750
2,000
m/z

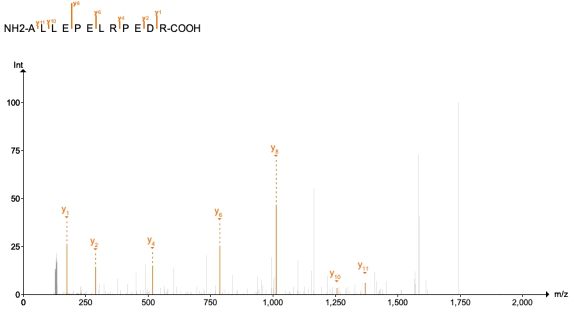

NH2-A L L E P E L R P E D R-COOH
y11 y10 y8 y6 y4 y2 y1
Int
m/z
0
25
50
75
100
250
500
750
1,000
1,250
1,500
1,750
2,000
y1
y2
y4
y6
y8
y10
y11

NH2-A L P E V H Y H L G Q K-COOH

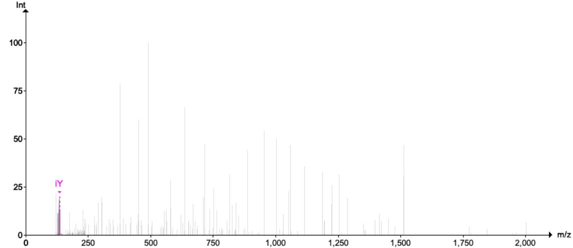

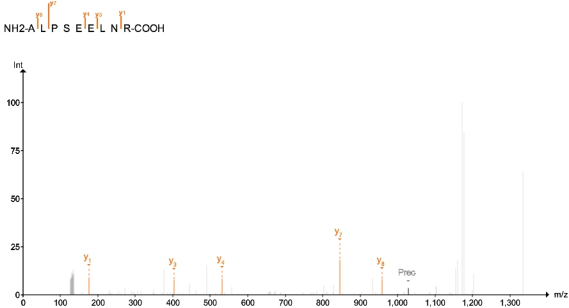

NH2-A L P S E E L N R-COOH
y8
y7
y4
y3
y1
Int
100
75
50
25
0
y1
y3
y4
y7
y8
Prec
0
100
200
300
400
500
600
700
800
900
1,000
1,100
1,200
1,300
m/z

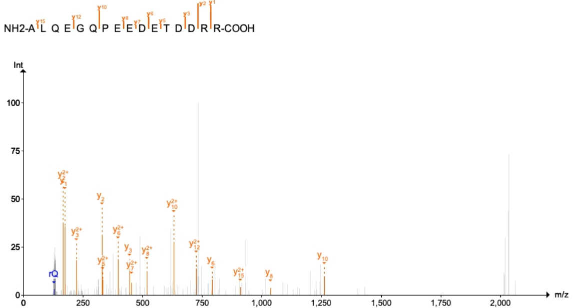

NH2-A L Q E G Q P E E D E T D D R R-COOH
y15
y12
y10
y8
y7
y6
y5
y3
y2
y1
Int
100
75
50
25
0
rQ
m/z
0
250
500
750
1,000
1,250
1,500
1,750
2,000

NH2-A L V D H H D A E V K-COOH

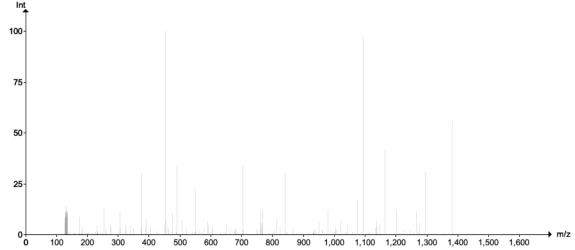

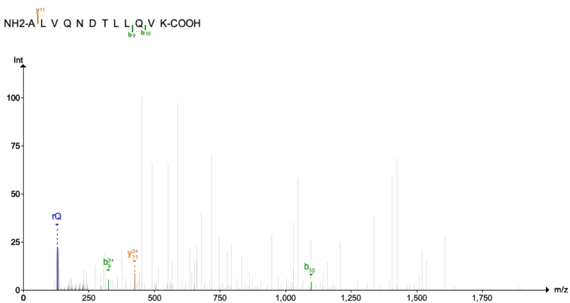

NH2-A L V Q N D T L L Q V K-COOH
y11
b9
b10
Int
100
75
50
25
0
rQ
b_9^{3+}
y_{11}^{3+}
b_{10}
0
250
500
750
1,000
1,250
1,500
1,750
m/z

NH2-A L V Y G H H G D P A K-COOH

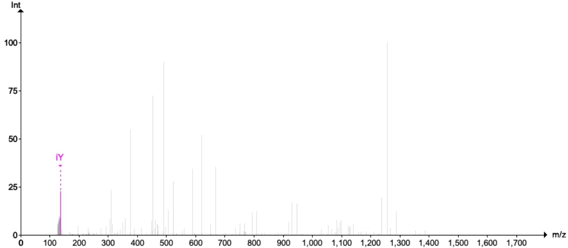

NH2-A L Y E Q N Q S D V N E A K-COOH

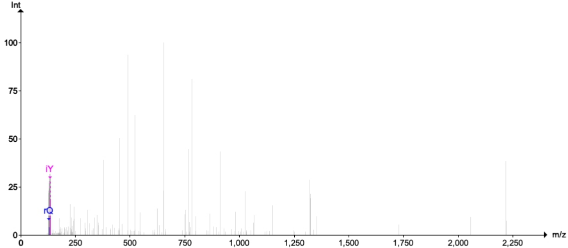

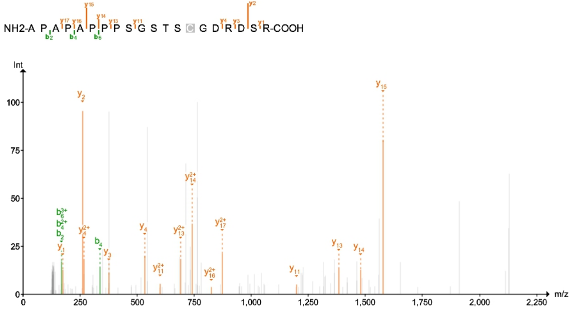

NH2-A P A P A P P S G S T S C G D R D S R-COOH
Int
m/z
0
25
50
75
100
0
250
500
750
1,000
1,250
1,500
1,750
2,000
2,250

NH2-A P D C H G P V L K-COOH

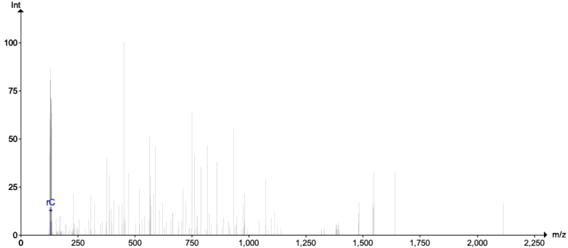

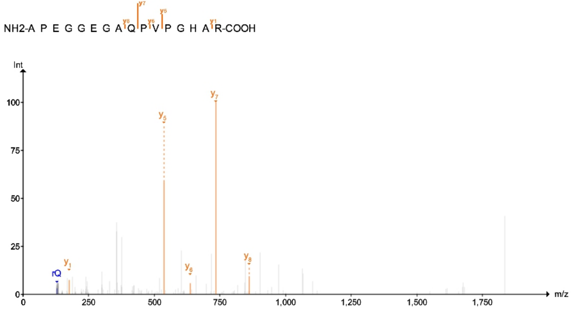

NH2-A P E G G E G A Q P V P G H A R-COOH
y8
y7
y6
y5
y1
Int
m/z
rQ
y1
y5
y6
y7
y8
100
75
50
25
0
0
250
500
750
1,000
1,250
1,500
1,750

NH2-A P H Q V P V Q S E K-COOH

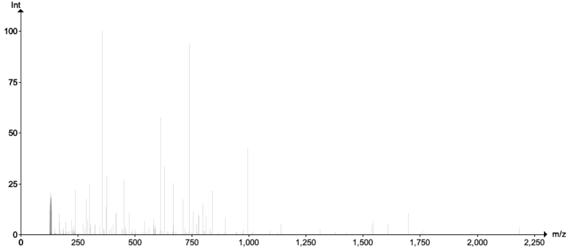

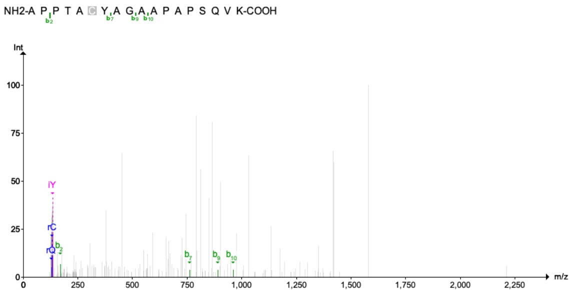

NH2-A P P T A C Y A G A A P A P S Q V K-COOH
b2
b7
b9
b10
Int
100
75
50
25
0
iY
rC
rQ
b2
b7
b9
b10
m/z
0
250
500
750
1,000
1,250
1,500
1,750
2,000
2,250

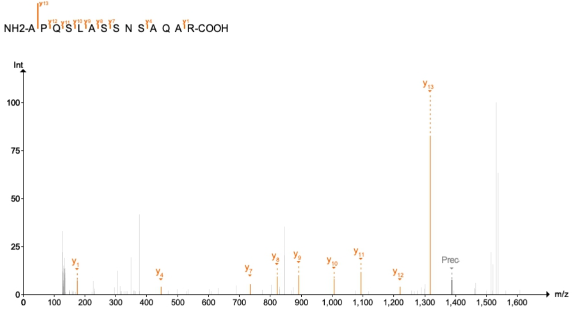

NH2-A P Q S L A S S N S A Q A R-COOH
y13
y12
y11
y10
y9
y8
y7
y4
y1
Int
100
75
50
25
0
y1
y4
y7
y8
y9
y10
y11
y12
y13
Prec
0
100
200
300
400
500
600
700
800
900
1,000
1,100
1,200
1,300
1,400
1,500
1,600
m/z

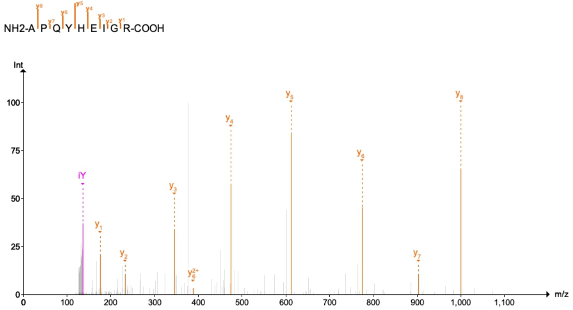
NH2-A P Q Y H E I G R-COOH
y8
y7
y6
y5
y4
y3
y2
y1
Int
100
75
50
25
0
iY
y1
y2
y3
y6 2+
y4
y5
y6
y7
y8
0
100
200
300
400
500
600
700
800
900
1,000
1,100
m/z

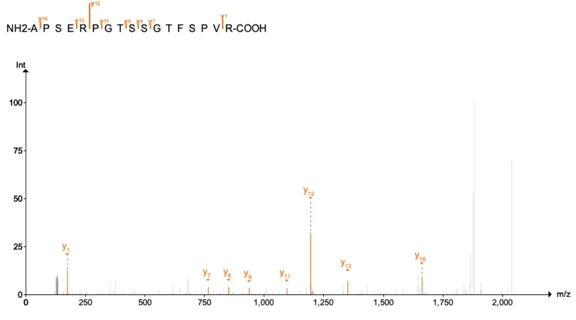

NH2-A P S E R P G T S S G T F S P V R-COOH
y16
y13
y12
y11
y9
y8
y7
y1
Int
100
75
50
25
0
250
500
750
1,000
1,250
1,500
1,750
2,000
m/z

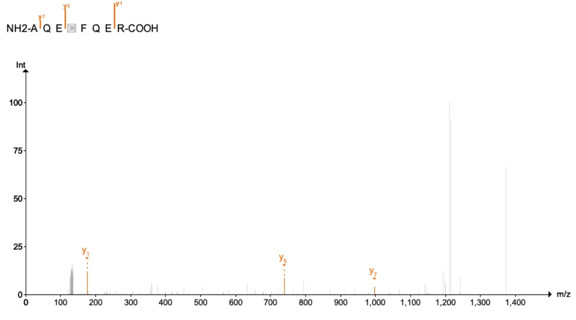
NH2-A Q E C F Q E R-COOH
y7
y5
y1
Int
100
75
50
25
0
y1
y5
y7
0
100
200
300
400
500
600
700
800
900
1,000
1,100
1,200
1,300
1,400
m/z

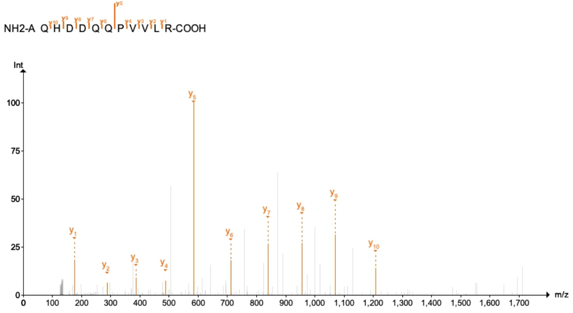

NH2-A Q H D D Q Q P V V L R-COOH
y10 y9 y8 y7 y6 y5 y4 y3 y2 y1
Int
100
75
50
25
0
y1 y2 y3 y4 y5 y6 y7 y8 y9 y10
0 100 200 300 400 500 600 700 800 900 1,000 1,100 1,200 1,300 1,400 1,500 1,600 1,700
m/z

NH2-A Q W D I G I A H A E K-COOH

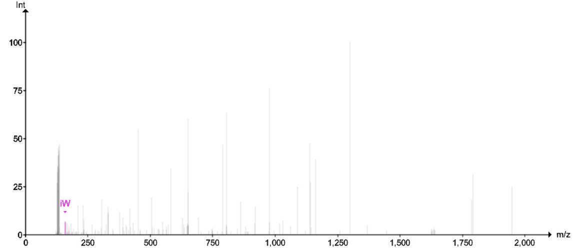

NH2-A S E Y S P A S L D A F G A F L K-COOH

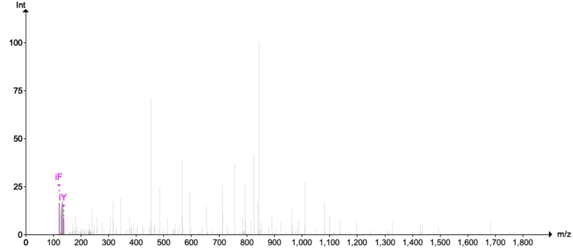

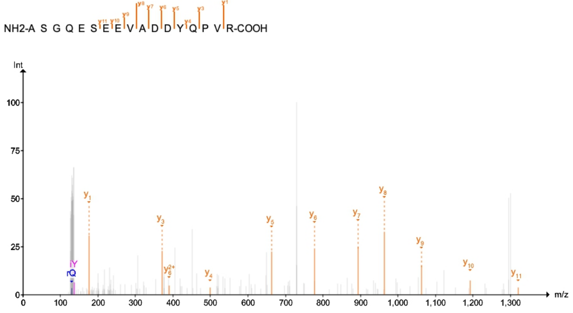
NH2-A S G Q E S E E V A D D Y Q P V R-COOH
y11 y10 y9 y8 y7 y6 y5 y4 y3 y1
Int
100
75
50
25
0
iY
rQ
y1
y3
y6 2+
y4
y5
y6
y7
y8
y9
y10
y11
0 100 200 300 400 500 600 700 800 900 1,000 1,100 1,200 1,300
m/z

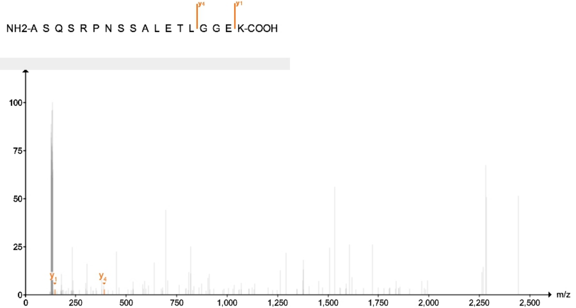

NH2-A S Q S R P N S S A L E T L G G E K-COOH
y4
y1
100
75
50
25
0
0
250
500
750
1,000
1,250
1,500
1,750
2,000
2,250
2,500
m/z

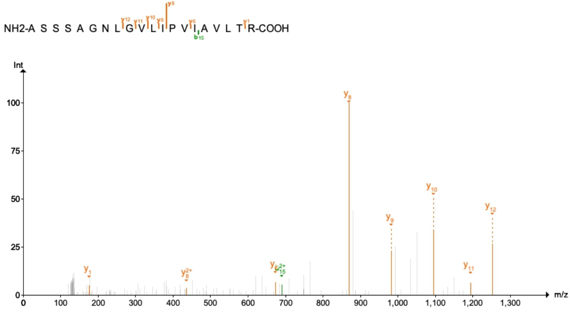
NH2-A S S S A G N L G V L I P V I A V L T R-COOH
y12
y11
y10
y9
y8
y6
y1
b15
Int
100
75
50
25
0
y1
y8 2+
y6
b15 2+
y8
y9
y10
y11
y12
0
100
200
300
400
500
600
700
800
900
1,000
1,100
1,200
1,300
m/z

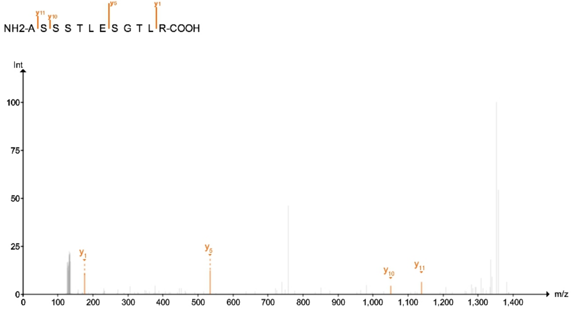

y11
y10
y5
y1
NH2-A S S S T L E S G T L R-COOH
Int
100
75
50
25
0
y1
y5
y10
y11
0
100
200
300
400
500
600
700
800
900
1,000
1,100
1,200
1,300
1,400
m/z

NH2-A T F D A I S K-COOH

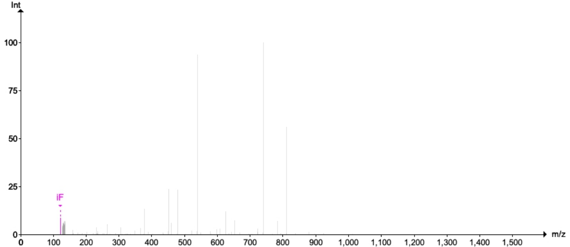

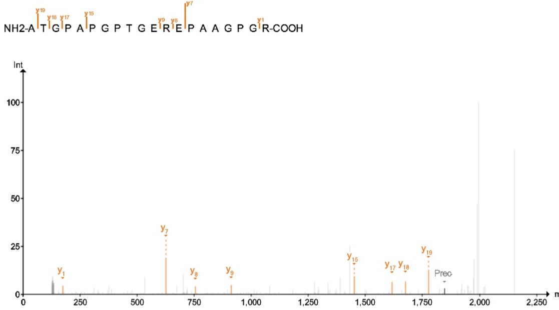

NH2-A T G P A P G P T G E R E P A A G P G R-COOH
y19
y18
y17
y15
y9
y8
y7
y1
Int
100
75
50
25
0
y1
y7
y8
y9
y15
y17
y18
y19
Prec
0
250
500
750
1,000
1,250
1,500
1,750
2,000
2,250
m/z

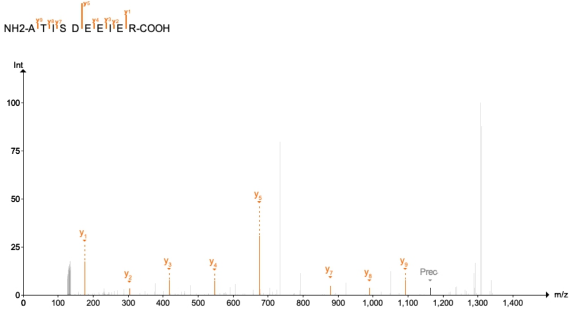
NH2-A T I S D E E I E R-COOH
y9 y8 y7 y5 y4 y3 y2 y1
Int
100
75
50
25
0
y1
y2
y3
y4
y5
y7
y8
y9
Prec
0 100 200 300 400 500 600 700 800 900 1,000 1,100 1,200 1,300 1,400
m/z

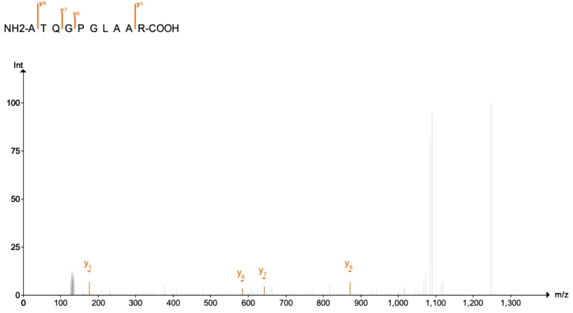

NH2-A T Q G P G L A A R-COOH
y9
y7
y6
y1
Int
100
75
50
25
0
y1
y6
y7
y9
0
100
200
300
400
500
600
700
800
900
1,000
1,100
1,200
1,300
m/z

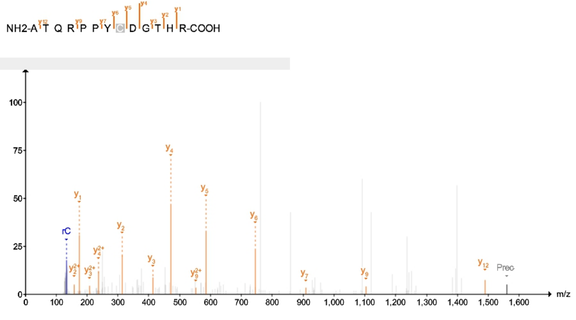

NH2-ATQRPPYCDGTHR-COOH
y12
y9
y7
y6
y5
y4
y3
y2
y1
rC
y2+ 2
y2+ 3
y2+ 4
y2+ 9
Prec
m/z

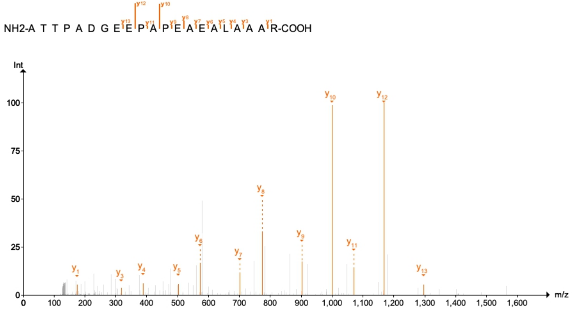

NH2-A T T P A D G E E P A P E A E A L A A A R-COOH
y13 y12 y11 y10 y9 y8 y7 y6 y5 y4 y3 y1
Int
m/z
y1
y3
y4
y5
y6
y7
y8
y9
y10
y11
y12
y13
0
25
50
75
100
0
100
200
300
400
500
600
700
800
900
1,000
1,100
1,200
1,300
1,400
1,500
1,600

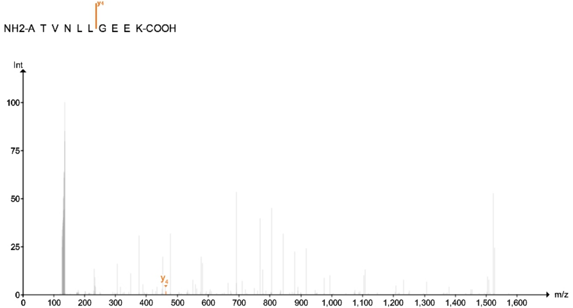

NH2-A T V N L L G E E K-COOH
y4
Int
100
75
50
25
0
0 100 200 300 400 500 600 700 800 900 1,000 1,100 1,200 1,300 1,400 1,500 1,600
m/z

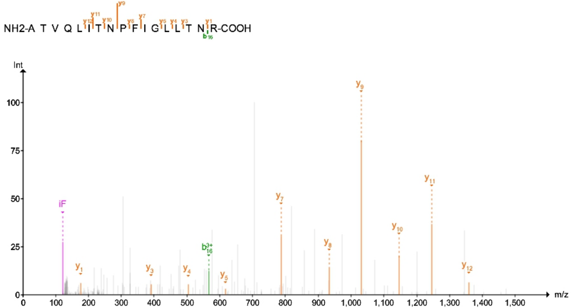
NH2-A T V Q L I T N P F I G L L T N R-COOH
y9
y11
y12
y10
y8
y7
y5
y4
y3
y1
b16
Int
100
75
50
25
0
iF
y1
y3
y4
b16 3+
y5
y7
y8
y9
y10
y11
y12
0
100
200
300
400
500
600
700
800
900
1,000
1,100
1,200
1,300
1,400
1,500
m/z

NH2-A T V Q S V L L D S A G K-COOH

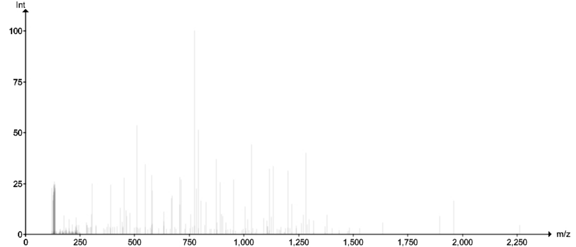

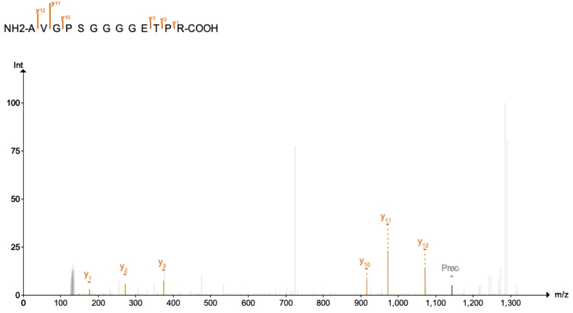

NH2-A V G P S G G G G E T P R-COOH
y12
y11
y10
y3
y2
y1
Int
100
75
50
25
0
y1
y2
y3
y10
y11
y12
Prec
0
100
200
300
400
500
600
700
800
900
1,000
1,100
1,200
1,300
m/z

NH2-A V P E A P G T V E C S K-COOH

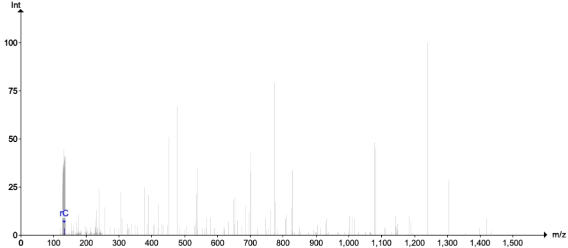

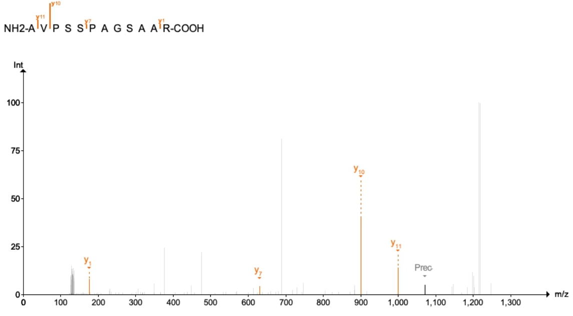

NH2-A V P S S P A G S A A R-COOH
y11
y10
y7
y1
Int
100
75
50
25
0
y1
y7
y10
y11
Prec
m/z
0
100
200
300
400
500
600
700
800
900
1,000
1,100
1,200
1,300

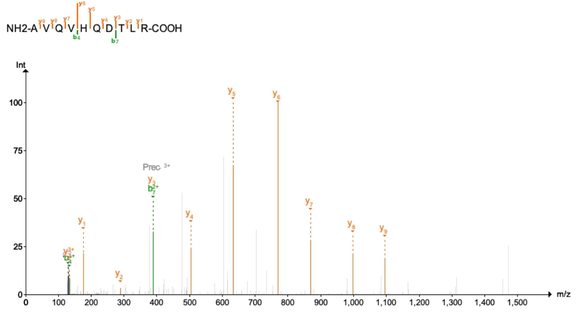
NH2-AVQVHQDTLR-COOH
y9 y8 y7 y6 y5 y4 y3 y2 y1
b4 b7
Int
m/z
Prec 3+
y1
y2
y3
y4
y5
y6
y7
y8
y9
0
25
50
75
100
0
100
200
300
400
500
600
700
800
900
1,000
1,100
1,200
1,300
1,400
1,500

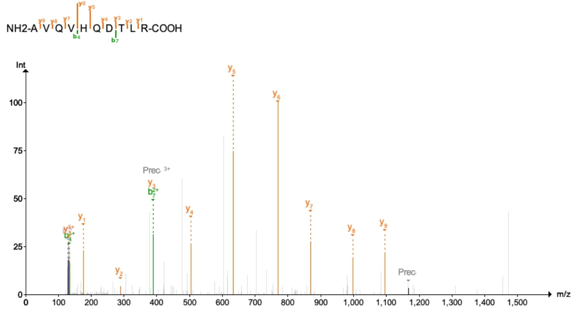
NH2-AVQVHQDTLR-COOH
Int
m/z
Prec 3+
Prec
y1
y2
y3
y4
y5
y6
y7
y8
y9
b4
b7
b7 2+
y3 3+
b4 2+

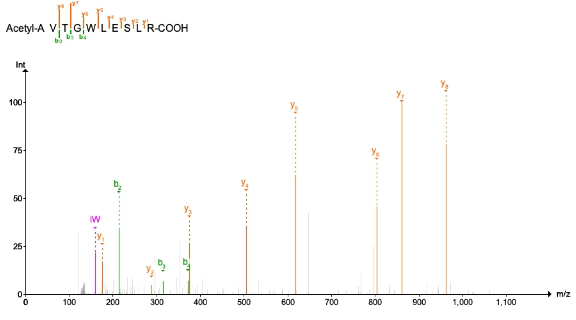

Acetyl-A V T G W L E S L R-COOH
Int
m/z
iW

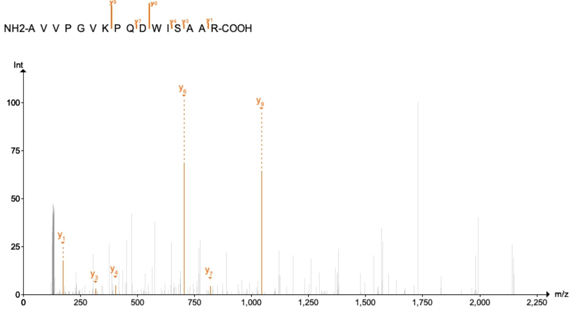

NH2-A V V P G V K P Q D W I S A A R-COOH
y9
y6
y7
y4
y3
y1
Int
100
75
50
25
0
y6
y9
y1
y3
y4
y7
0
250
500
750
1,000
1,250
1,500
1,750
2,000
2,250
m/z

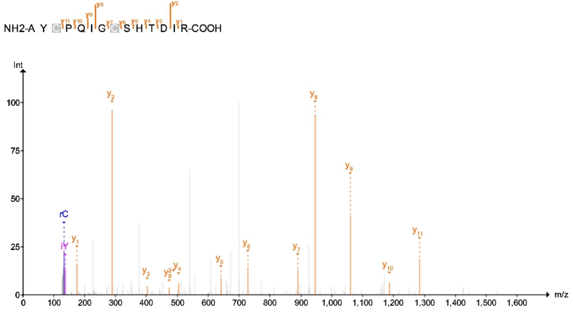

NH2-A Y C P Q I G C S H T D I R-COOH
y11 y10 y9 y8 y7 y6 y5 y4 y3 y2 y1
Int
m/z
rC
iY
y1
y2
y3
y4
y5
y6
y7
y8
y9
y10
y11

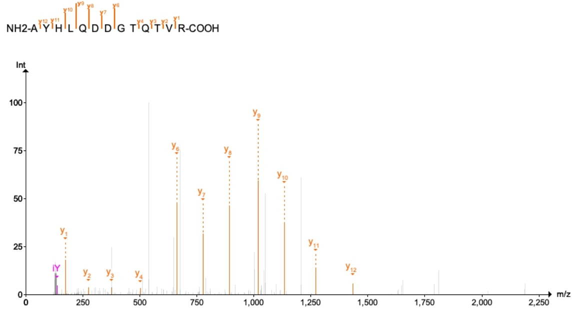

NH2-A Y H L Q D D G T Q T V R-COOH
y12 y11 y10 y9 y8 y7 y6 y4 y3 y2 y1
Int
100
75
50
25
0
y1
y2
y3
y4
y6
y7
y8
y9
y10
y11
y12
IY
m/z
0
250
500
750
1,000
1,250
1,500
1,750
2,000
2,250

NH2-A Y L E P E H T K-COOH

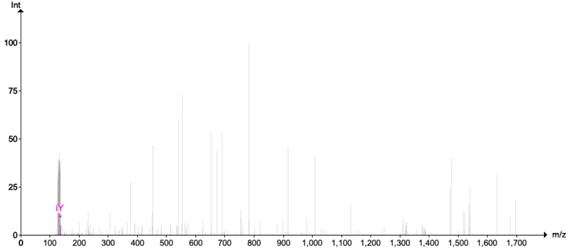

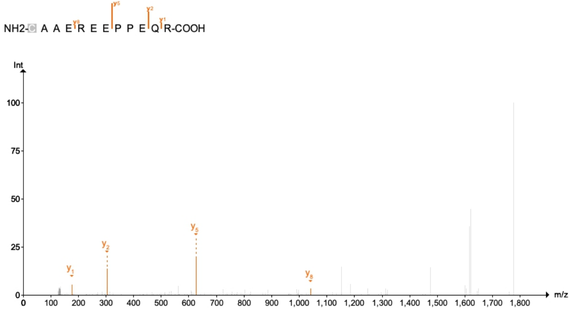
NH2-C A A E R E E P P E Q R-COOH
y8
y5
y2
y1
Int
100
75
50
25
0
y1
y2
y5
y8
0
100
200
300
400
500
600
700
800
900
1,000
1,100
1,200
1,300
1,400
1,500
1,600
1,700
1,800
m/z

NH2-C A N L F E A L V G T L K-COOH

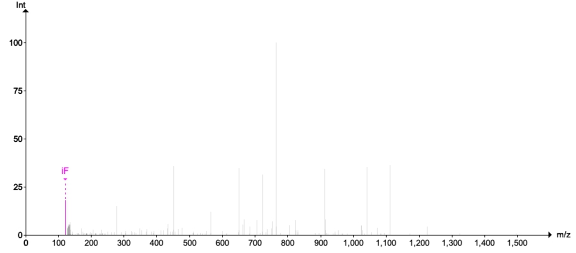

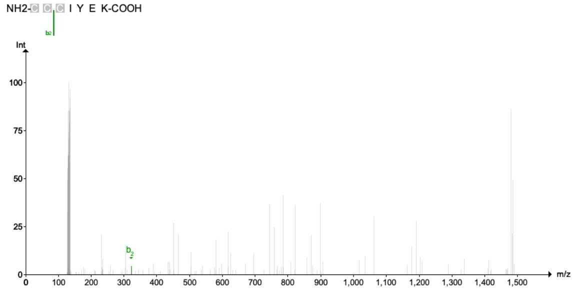

NH2-C C C I Y E K-COOH
b2
Int
100
75
50
25
0
b2
0
100
200
300
400
500
600
700
800
900
1,000
1,100
1,200
1,300
1,400
1,500
m/z

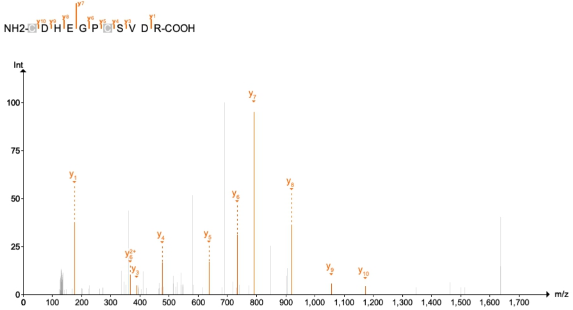

NH2-C D H E G P C S V D R-COOH
y10 y9 y8 y7 y6 y5 y4 y3 y1
Int
100
75
50
25
0
y1
y6 2+
y3
y4
y5
y6
y7
y8
y9
y10
0 100 200 300 400 500 600 700 800 900 1,000 1,100 1,200 1,300 1,400 1,500 1,600 1,700
m/z

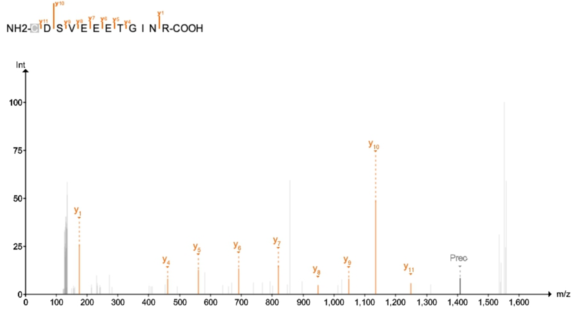

NH2-C D S V E E E T G I N R-COOH
Int
m/z
y1
y4
y5
y6
y7
y8
y9
y10
y11
Prec
0
25
50
75
100
0
100
200
300
400
500
600
700
800
900
1,000
1,100
1,200
1,300
1,400
1,500
1,600

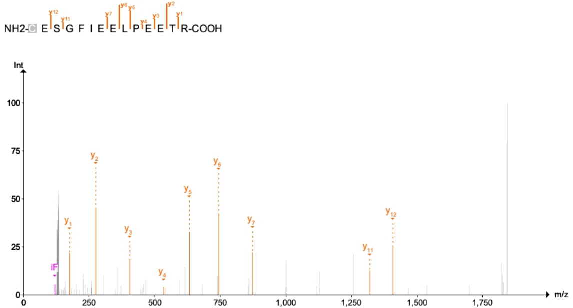
NH2-C E S G F I E E L P E E T R-COOH
y12
y11
y7
y6
y5
y4
y3
y2
y1
Int
100
75
50
25
0
iF
y1
y2
y3
y4
y5
y6
y7
y11
y12
0
250
500
750
1,000
1,250
1,500
1,750
m/z

NH2-C L C A I D I H N K-COOH

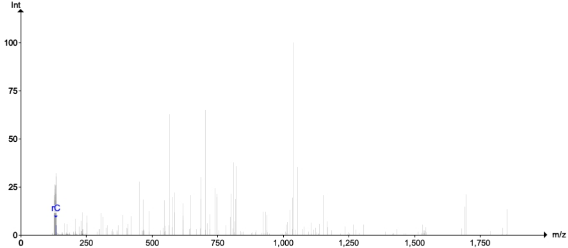

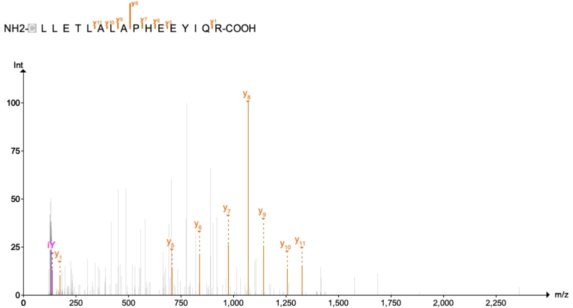

NH2-C L L E T L A L A P H E E Y I Q R-COOH
y11 y10 y9 y8 y7 y6 y5 y1
Int
100
75
50
25
0
iY
y1 y5 y6 y7 y8 y9 y10 y11
0
250
500
750
1,000
1,250
1,500
1,750
2,000
2,250
m/z

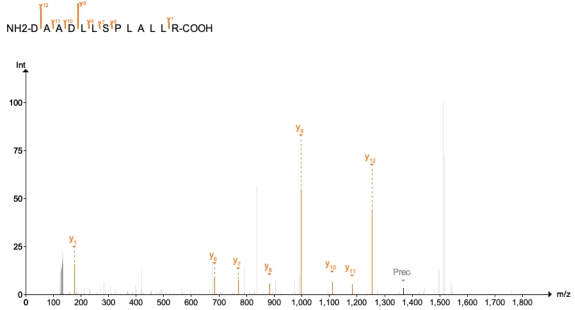
NH2-D A A D L L S P L A L L R-COOH
y12
y11
y10
y9
y8
y7
y6
y1
Int
m/z
Prec
y1
y6
y7
y8
y9
y10
y11
y12
0
25
50
75
100
0 100 200 300 400 500 600 700 800 900 1,000 1,100 1,200 1,300 1,400 1,500 1,600 1,700 1,800

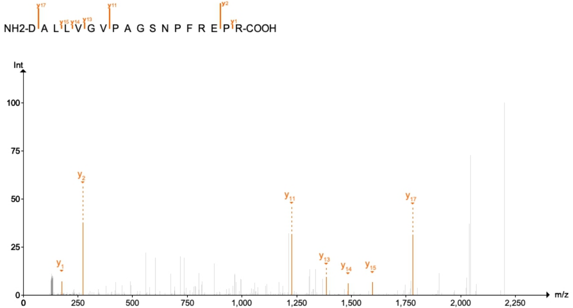
NH2-D A L L V G V P A G S N P F R E P R-COOH
y17
y15
y14
y13
y11
y2
y1
Int
100
75
50
25
0
y2
y11
y17
y1
y13
y14
y15
0
250
500
750
1,000
1,250
1,500
1,750
2,000
2,250
m/z

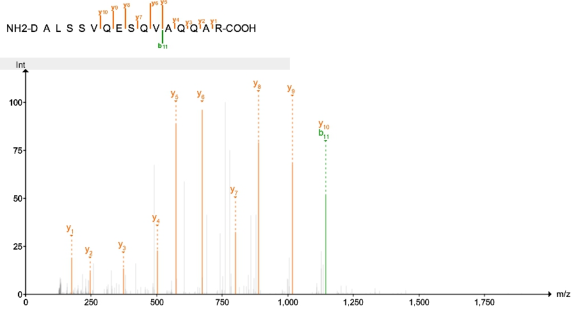

NH2-D A L S S V Q E S Q V A Q Q A R-COOH
Int
m/z

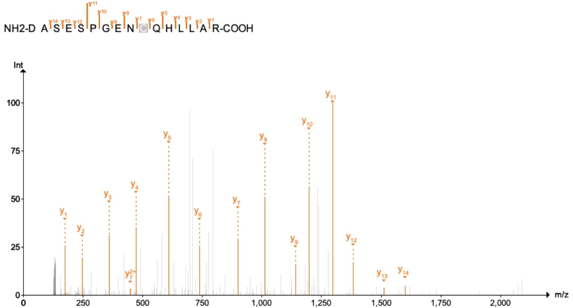
NH2-D A S E S P G E N C Q H L L A R-COOH
y14 y13 y12 y11 y10 y9 y8 y7 y6 y5 y4 y3 y2 y1
Int
100
75
50
25
0
y1
y2
y3
y4
y5
y6
y7
y7 2+
y8
y9
y10
y11
y12
y13
y14
0
250
500
750
1,000
1,250
1,500
1,750
2,000
m/z

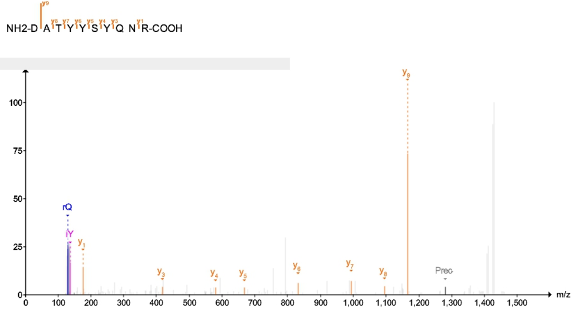
NH2-D A T Y Y S Y Q N R-COOH
y9
y8 y7 y6 y5 y4 y3
y1
rQ
iY
y1
y3
y4
y5
y6
y7
y8
y9
Prec
100
75
50
25
0
0 100 200 300 400 500 600 700 800 900 1,000 1,100 1,200 1,300 1,400 1,500
m/z

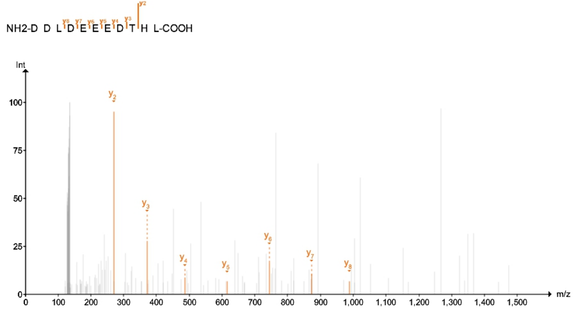
NH2-D D L D E E E D T H L-COOH
y8 y7 y6 y5 y4 y3 y2
Int
100
75
50
25
0
y2
y3
y4
y5
y6
y7
y8
m/z
0 100 200 300 400 500 600 700 800 900 1,000 1,100 1,200 1,300 1,400 1,500

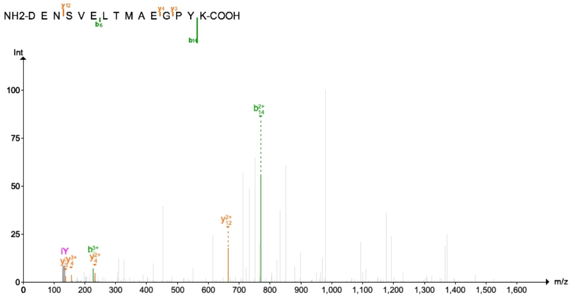

NH2-D E N S V E L T M A E G P Y K-COOH
y12
b6
y4
y3
b14
Int
m/z
b14 2+
y12 2+
iY
b3+
y4 2+
y4 3+

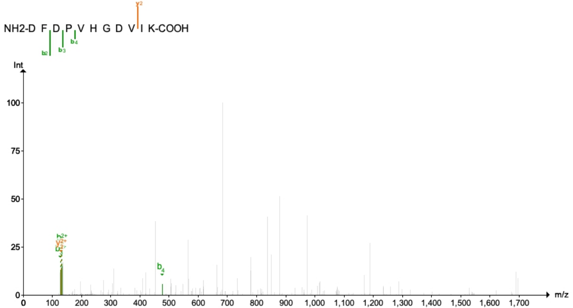
NH2-D F D P V H G D V I K-COOH
y2
b2
b3
b4
Int
100
75
50
25
0
b2+
y2+
b3
b4
0
100
200
300
400
500
600
700
800
900
1,000
1,100
1,200
1,300
1,400
1,500
1,600
1,700
m/z

NH2-D F H L I Q A N K-COOH

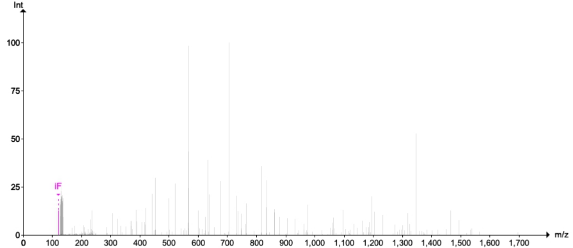

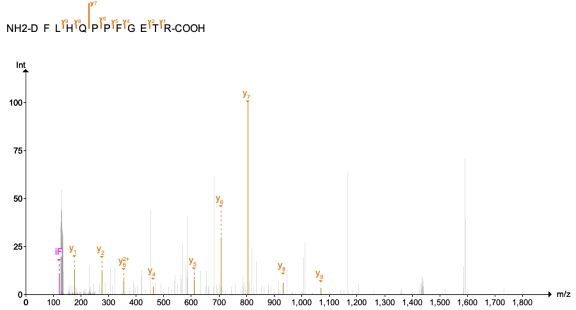

NH2-D F L H Q P P F G E T R-COOH
y9 y8 y7 y6 y5 y4 y2 y1
Int
100
75
50
25
0
iF
y1
y2
y6 2+
y4
y5
y6
y7
y8
y9
m/z
0 100 200 300 400 500 600 700 800 900 1,000 1,100 1,200 1,300 1,400 1,500 1,600 1,700 1,800

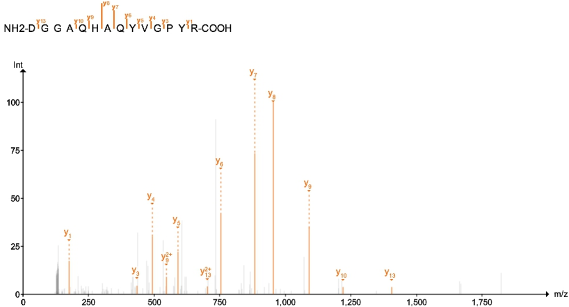

NH2-D G G A Q H A Q Y V G P Y R-COOH
y13 y10 y9 y8 y7 y6 y5 y4 y3 y1
Int
m/z
y1
y3
y4
y5
y6
y7
y8
y9
y10
y13
y9 2+
y13 2+
0
25
50
75
100
250
500
750
1,000
1,250
1,500
1,750

NH2-D G L A L G P G P F V T A L E Y A T D T K-COOH

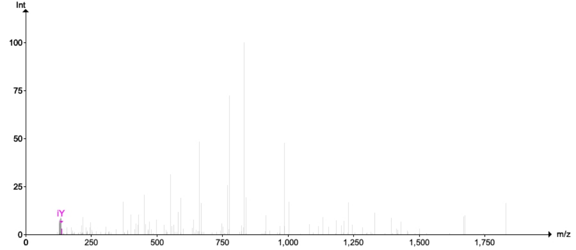

NH2-D G P V R P Q N A E E E K-COOH

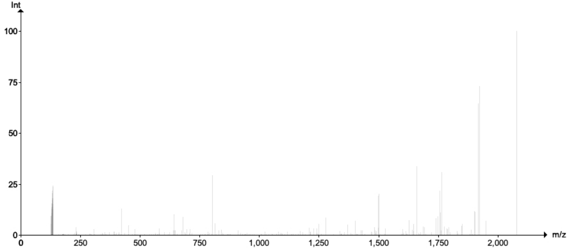

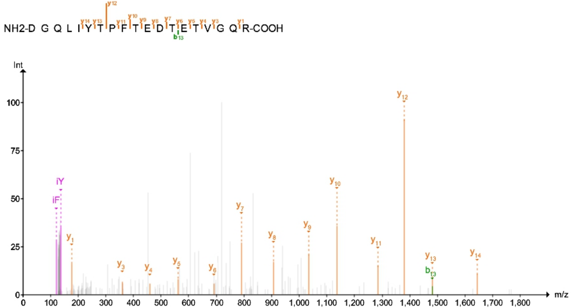
NH2-D G Q L I Y T P F T E D T E T V G Q R-COOH
y12
y14 y13 y11 y10 y9 y8 y7 y6 y5 y4 y3 y1
b13
Int
100
75
50
25
0
iY
iF
y1
y3
y4
y5
y6
y7
y8
y9
y10
y11
y12
y13
b13
y14
0 100 200 300 400 500 600 700 800 900 1,000 1,100 1,200 1,300 1,400 1,500 1,600 1,700 1,800
m/z

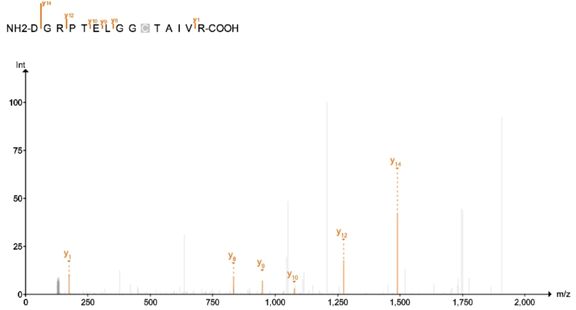

NH2-D G R P T E L G G C T A I V R-COOH
y14
y12
y10
y9
y8
y1
Int
100
75
50
25
0
250
500
750
1,000
1,250
1,500
1,750
2,000
m/z

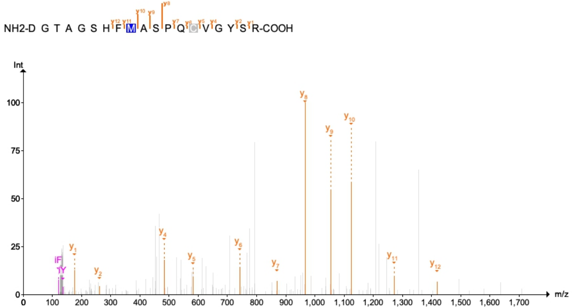

NH2-D G T A G S H F M A S P Q C V G Y S R-COOH
Int
m/z
y1
y2
y4
y5
y6
y7
y8
y9
y10
y11
y12
iF
iY

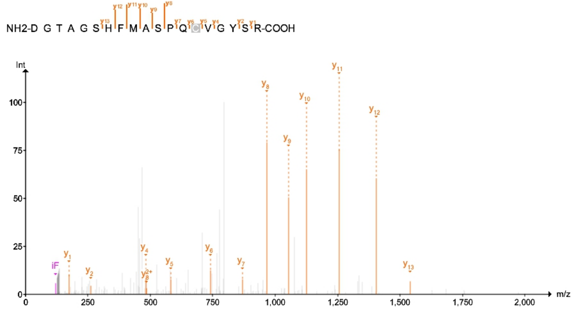
NH2-D G T A G S H F M A S P Q C V G Y S R-COOH
y13 y12 y11 y10 y9 y8 y7 y6 y5 y4 y2 y1
Int
m/z
100
75
50
25
0
0 250 500 750 1,000 1,250 1,500 1,750 2,000
y1 y2 y4 y8 2+ y5 y6 y7 y8 y9 y10 y11 y12 y13 iF

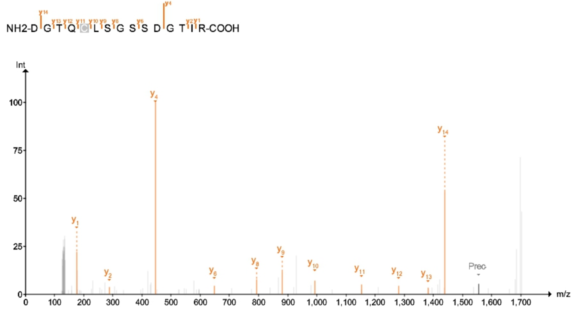

NH2-D G T Q C L S G S S D G T I R-COOH
y14
y13
y12
y11
y10
y9
y8
y6
y4
y2
y1
Int
100
75
50
25
0
y1
y2
y4
y6
y8
y9
y10
y11
y12
y13
y14
Prec
0
100
200
300
400
500
600
700
800
900
1,000
1,100
1,200
1,300
1,400
1,500
1,600
1,700
m/z

NH2-D H A E C P V S A C T C K-COOH

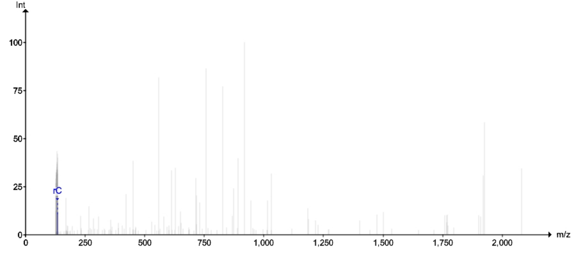

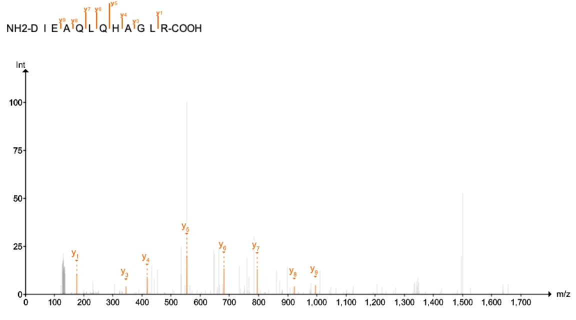
NH2-D I E A Q L Q H A G L R-COOH
y9
y8
y7
y6
y5
y4
y3
y1
Int
100
75
50
25
0
y1
y3
y4
y5
y6
y7
y8
y9
0
100
200
300
400
500
600
700
800
900
1,000
1,100
1,200
1,300
1,400
1,500
1,600
1,700
m/z

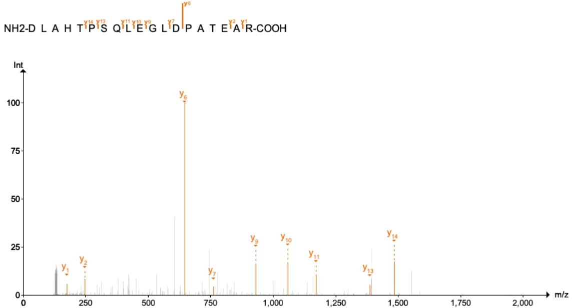
NH2-D L A H T P S Q L E G L D P A T E A R-COOH
Int
m/z
y1
y2
y6
y7
y9
y10
y11
y13
y14

NH2-D L E A L V T D K-COOH

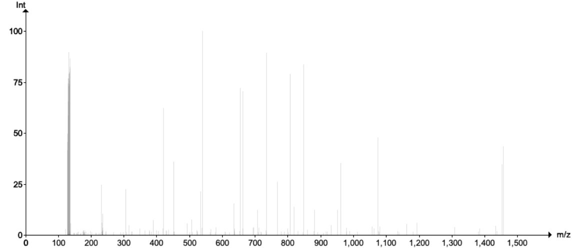

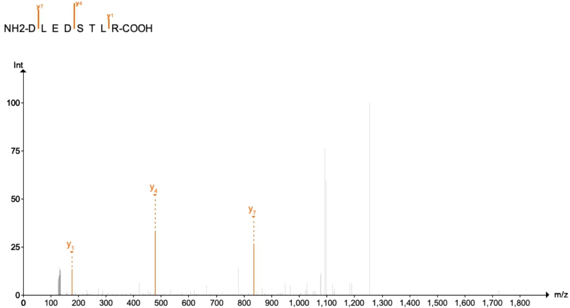
NH2-D L E D S T L R-COOH
y7
y4
y1
Int
100
75
50
25
0
y4
y7
y1
m/z
0
100
200
300
400
500
600
700
800
900
1,000
1,100
1,200
1,300
1,400
1,500
1,600
1,700
1,800

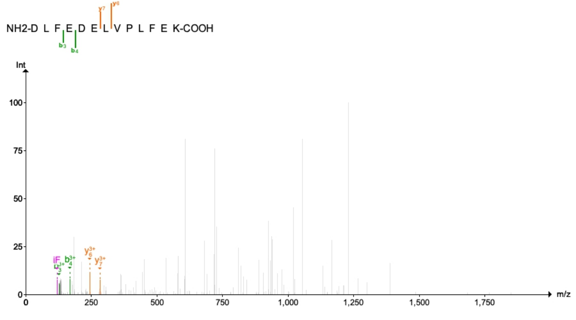

NH2-D L F E D E L V P L F E K-COOH
y7
y6
b3
b4
Int
100
75
50
25
0
iF
b3 3+
b4 3+
y6 3+
y7 3+
0
250
500
750
1,000
1,250
1,500
1,750
m/z

NH2-D L F G S L N V K-COOH

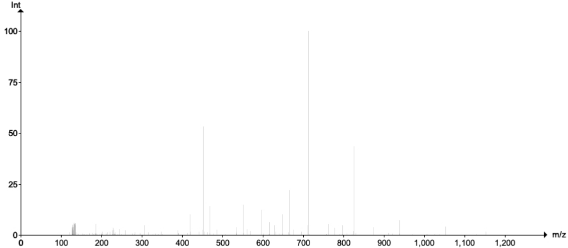

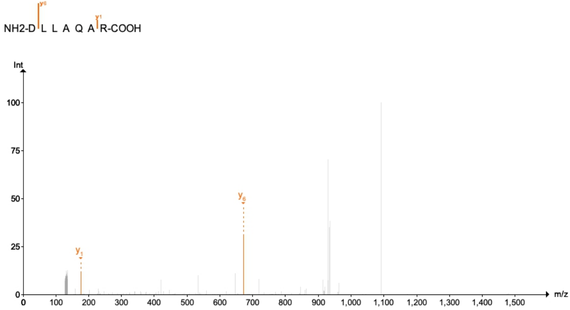

NH2-D L L A Q A R-COOH
y6
y1
Int
100
75
50
25
0
y1
y6
0
100
200
300
400
500
600
700
800
900
1,000
1,100
1,200
1,300
1,400
1,500
m/z

NH2-D L L H C P S K-COOH

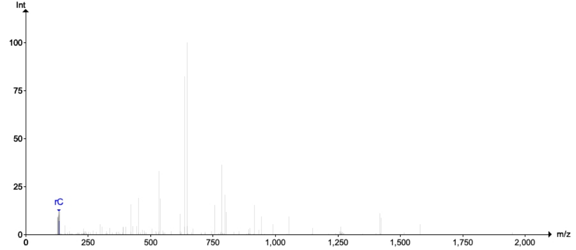

NH2-D L L N A L K-COOH

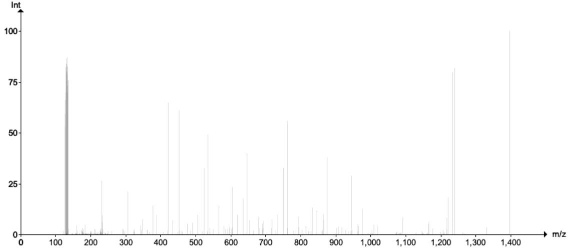

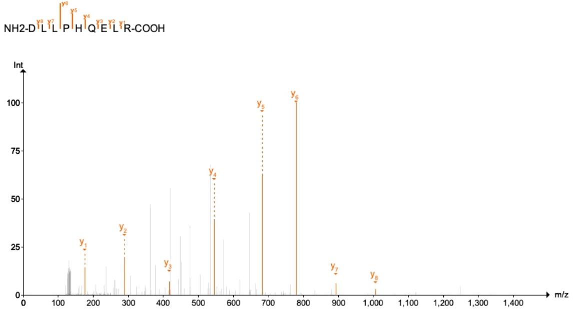

NH2-D L L P H Q E L R-COOH
y8 y7 y6 y5 y4 y3 y2 y1
Int
100
75
50
25
0
y1
y2
y3
y4
y5
y6
y7
y8
0 100 200 300 400 500 600 700 800 900 1,000 1,100 1,200 1,300 1,400
m/z

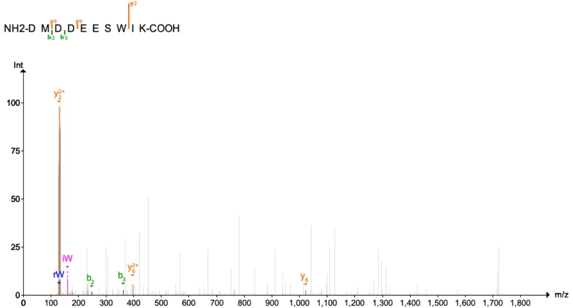

NH2-D M D D E E S W I K-COOH
y8
y6
y2
b2
b3
Int
100
75
50
25
0
y2 2+
iW
rW
b2
b3
y6 2+
y8
m/z
0
100
200
300
400
500
600
700
800
900
1,000
1,100
1,200
1,300
1,400
1,500
1,600
1,700
1,800

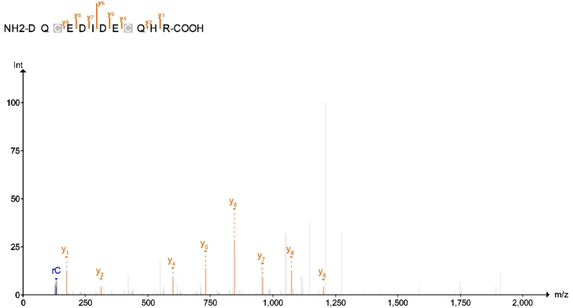
NH2-D Q C E D I D E C Q H R-COOH
Int
m/z
rC
y1
y2
y4
y5
y6
y7
y8
y9

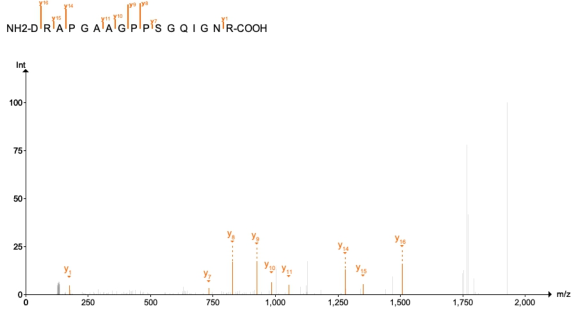
NH2-D R A P G A A G P P S G Q I G N R-COOH
Int
100
75
50
25
0
y1
y7
y8
y9
y10
y11
y14
y15
y16
0
250
500
750
1,000
1,250
1,500
1,750
2,000
m/z

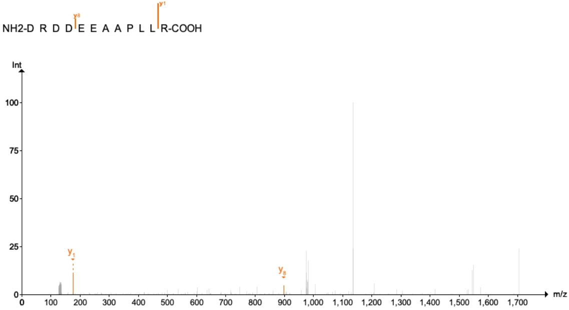

NH2-D R D D E E A A P L L R-COOH
y8
y1
Int
100
75
50
25
0
y1
y8
0
100
200
300
400
500
600
700
800
900
1,000
1,100
1,200
1,300
1,400
1,500
1,600
1,700
m/z

NH2-D R T Q E F L S A C K-COOH

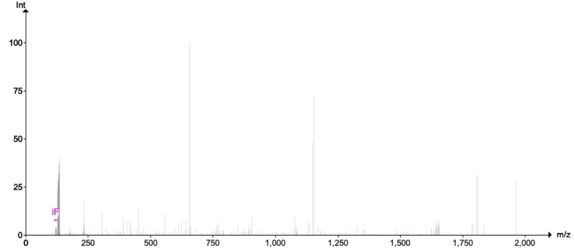

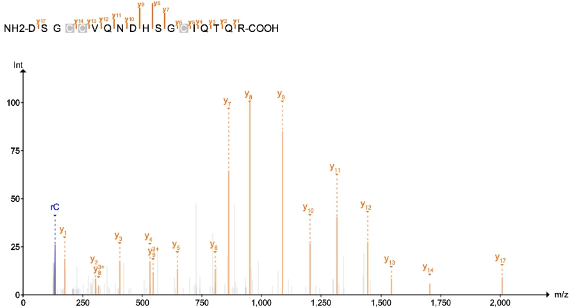

NH2-D S G C C V Q N D H S G C I Q T Q R-COOH
y17 y14 y13 y12 y11 y10 y9 y8 y7 y6 y5 y4 y3 y2 y1
Int
m/z
rC
y1 y2 y8 3+ y3 y4 y9 2+ y5 y6 y7 y8 y9 y10 y11 y12 y13 y14 y17
0 25 50 75 100
0 250 500 750 1,000 1,250 1,500 1,750 2,000

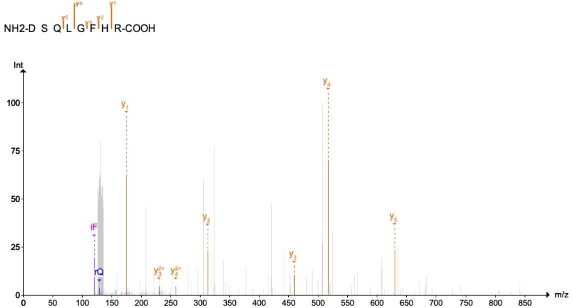

NH2-D S Q L G F H R-COOH
y5 y4 y3 y2 y1
Int
100
75
50
25
0
y1
y2
y3
y4
y5
y3 2+
y4 2+
iF
rQ
0 50 100 150 200 250 300 350 400 450 500 550 600 650 700 750 800 850
m/z

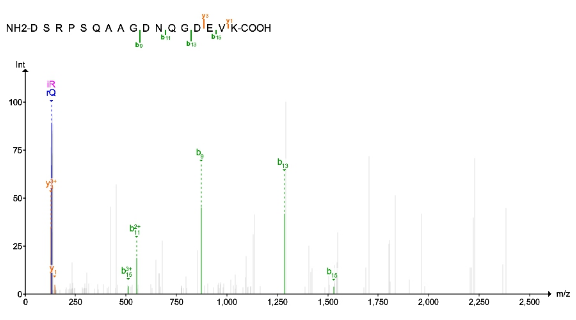

NH2-D S R P S Q A A G D N Q G D E V K-COOH
b9
b11
b13
b15
y3
y1
Int
iR
rQ
100
75
50
25
0
y_3^{3+}
b_9
b_{13}
b_{11}^{2+}
b_{15}^{3+}
b_{15}
y_1
250
500
750
1,000
1,250
1,500
1,750
2,000
2,250
2,500
m/z

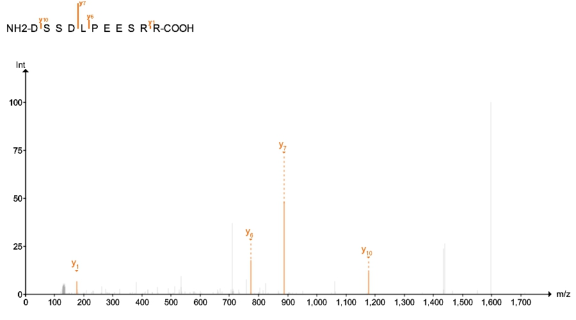
NH2-D S S D L P E E S R R-COOH
y10
y7
y6
y1
Int
100
75
50
25
0
y1
y6
y7
y10
0
100
200
300
400
500
600
700
800
900
1,000
1,100
1,200
1,300
1,400
1,500
1,600
1,700
m/z

NH2-D T A D V V H P F F L A Q K-COOH

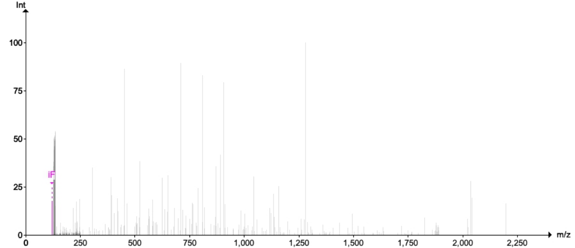

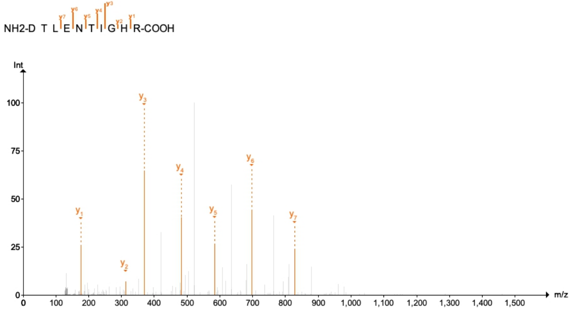
NH2-D T L E N T I G H R-COOH
y7 y6 y5 y4 y3 y2 y1
Int
m/z
y1 y2 y3 y4 y5 y6 y7

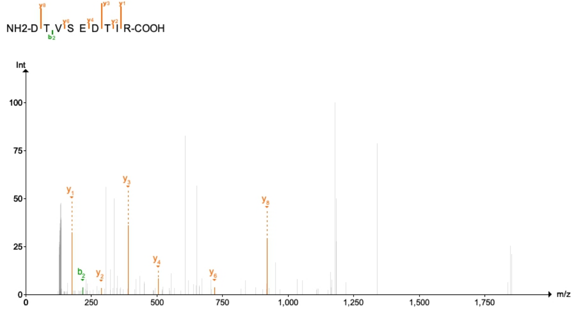

NH2-D T V S E D T I R-COOH
y8
b2
y6
y4
y3
y2
y1
Int
100
75
50
25
0
250
500
750
1,000
1,250
1,500
1,750
m/z
y1
y3
y8
y4
b2
y2
y6

NH2-D V D T Y Y L S N L V K-COOH

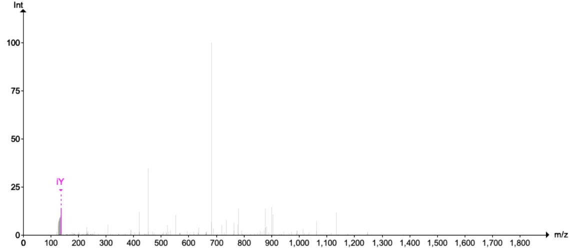

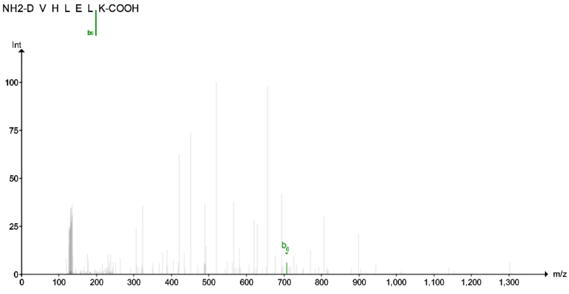

NH2-D V H L E L K-COOH
b6
Int
100
75
50
25
0
b6
0
100
200
300
400
500
600
700
800
900
1,000
1,100
1,200
1,300
m/z

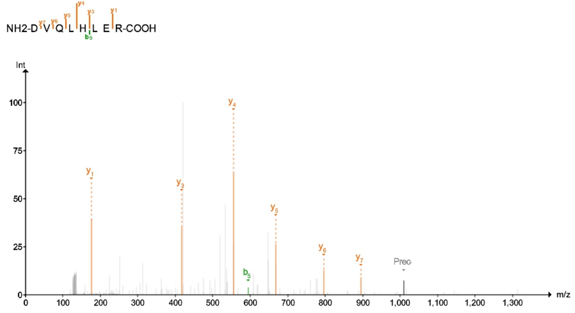
NH2-D V Q L H L E R-COOH
y7 y6 y5 y4 y3 y1
b5
Int
100
75
50
25
0
y1
y3
y4
y5
b5
y6
y7
Prec
0
100
200
300
400
500
600
700
800
900
1,000
1,100
1,200
1,300
m/z

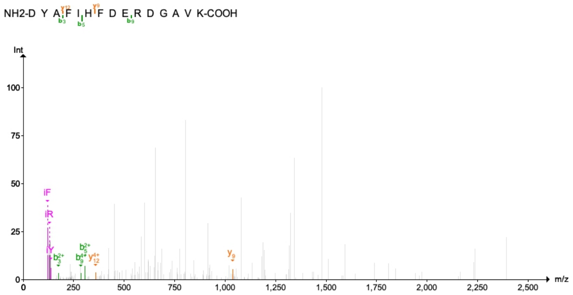

NH2-D Y A F I H F D E R D G A V K-COOH
y12
y9
b3
b5
b9
Int
100
75
50
25
0
iF
iR
iY
b3 2+
b5 2+
b9 4+
y12 4+
y9
0
250
500
750
1,000
1,250
1,500
1,750
2,000
2,250
2,500
m/z

NH2-D Y D S F V L P L L E D K-COOH

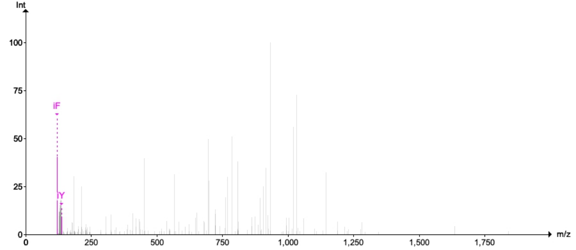

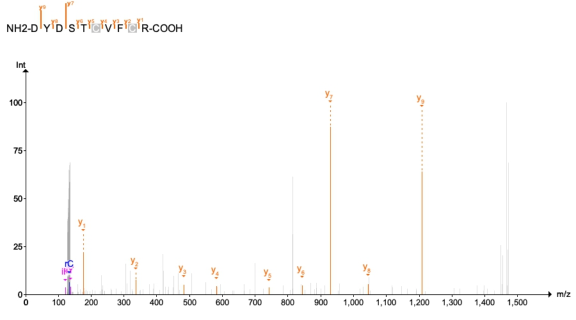
NH2-D Y D S T C V F C R-COOH
Int
m/z
y1
y2
y3
y4
y5
y6
y7
y8
y9
rC

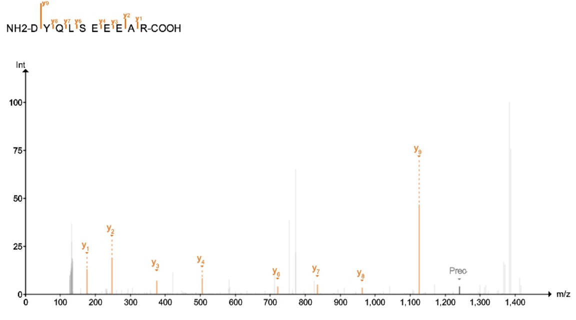

NH2-D Y Q L S E E E A R-COOH
y9
y8
y7
y6
y4
y3
y2
y1
Int
100
75
50
25
0
y1
y2
y3
y4
y6
y7
y8
y9
Prec
0
100
200
300
400
500
600
700
800
900
1,000
1,100
1,200
1,300
1,400
m/z

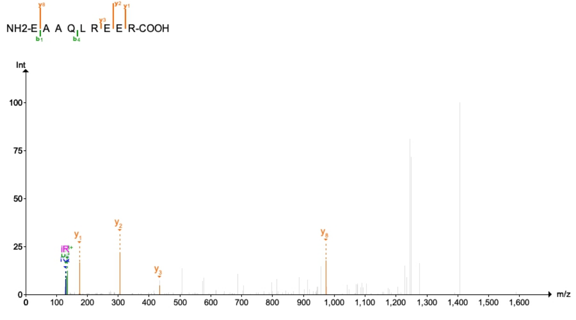
NH2-E A A Q L R E E R-COOH
Int
m/z
y1
y2
y3
y8

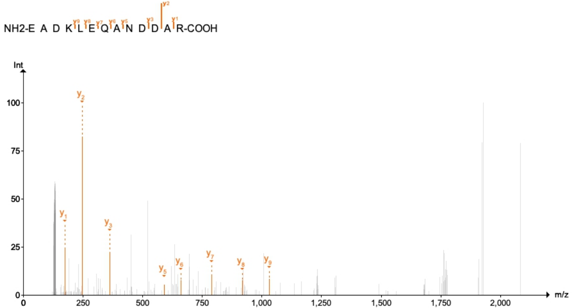
NH2-E A D K L E Q A N D D A R-COOH
Int
m/z
y1
y2
y3
y5
y6
y7
y8
y9
0
25
50
75
100
250
500
750
1,000
1,250
1,500
1,750
2,000

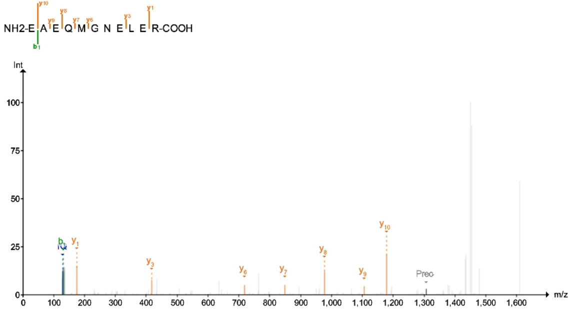

NH2-E A E Q M G N E L E R-COOH
y10
y9
y8
y7
y6
y3
y1
b1
Int
100
75
50
25
0
b1
y1
y3
y6
y7
y8
y9
y10
Prec
0
100
200
300
400
500
600
700
800
900
1,000
1,100
1,200
1,300
1,400
1,500
1,600
m/z

NH2-E A I E S A I G G N A Y Q H S K-COOH

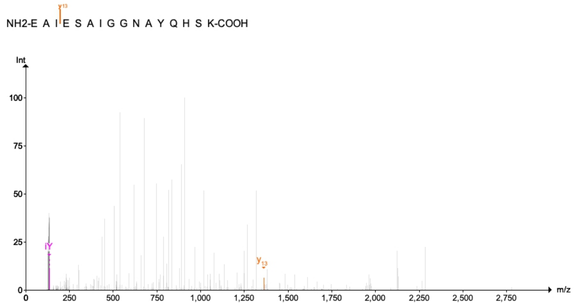

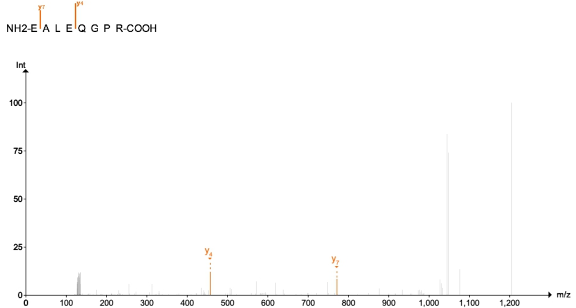
y7
y4
NH2-E A L E Q G P R-COOH
Int
100
75
50
25
0
y4
y7
0
100
200
300
400
500
600
700
800
900
1,000
1,100
1,200
m/z

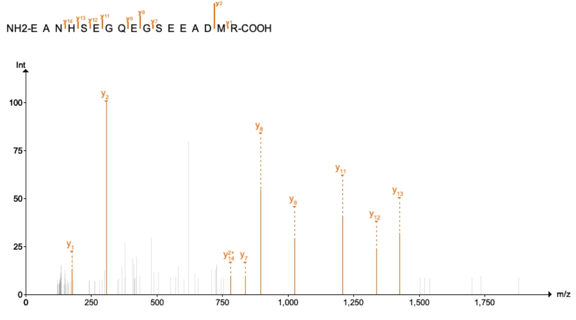

NH2-E A N H S E G Q E G S E E A D M R-COOH
y14 y13 y12 y11 y9 y8 y7 y2 y1
Int
100
75
50
25
0
y1
y2
y2+ 14
y7
y8
y9
y11
y12
y13
0
250
500
750
1,000
1,250
1,500
1,750
m/z

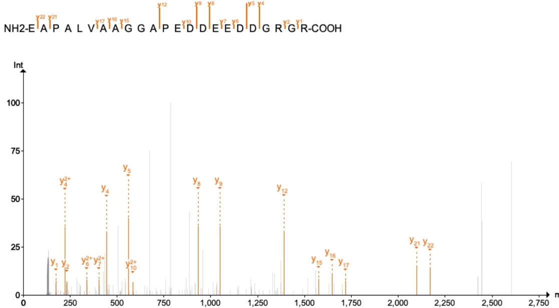
NH2-E A P A L V A A G G A P E D D E E D D G R G R-COOH
Int
m/z
y1
y2
y4
y5
y8
y9
y12
y15
y16
y17
y21
y22
0
25
50
75
100
0
250
500
750
1,000
1,250
1,500
1,750
2,000
2,250
2,500
2,750

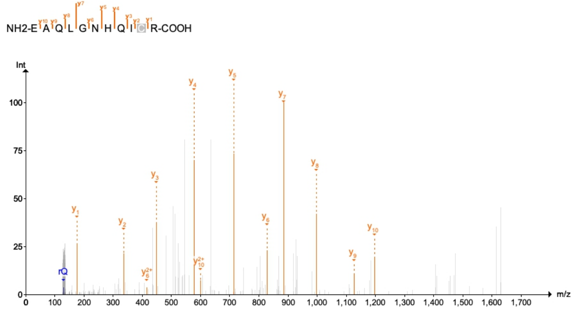

NH2-E A Q L G N H Q I C R-COOH
y10 y9 y8 y7 y6 y5 y4 y3 y2 y1
Int
m/z
y1
y2
y3
y4
y5
y6
y7
y8
y9
y10
y6 2+
y10 2+
rQ
0
25
50
75
100
0
100
200
300
400
500
600
700
800
900
1,000
1,100
1,200
1,300
1,400
1,500
1,600
1,700

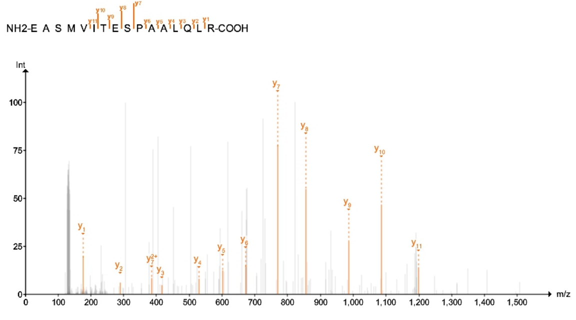
NH2-E A S M V I T E S P A A L Q L R-COOH
y11 y10 y9 y8 y7 y6 y5 y4 y3 y2 y1
Int
m/z
y1 y2 y7 2+ y3 y4 y5 y6 y7 y8 y9 y10 y11
0 25 50 75 100
0 100 200 300 400 500 600 700 800 900 1,000 1,100 1,200 1,300 1,400 1,500

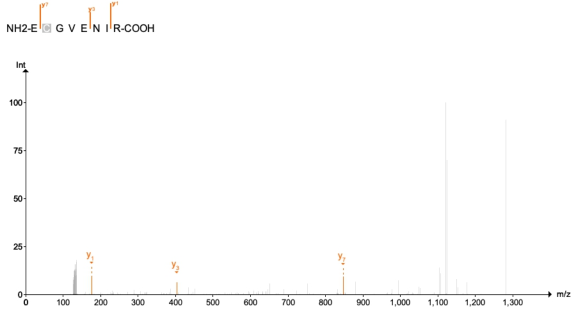

NH2-E C G V E N I R-COOH
y7
y3
y1
Int
100
75
50
25
0
m/z
0
100
200
300
400
500
600
700
800
900
1,000
1,100
1,200
1,300

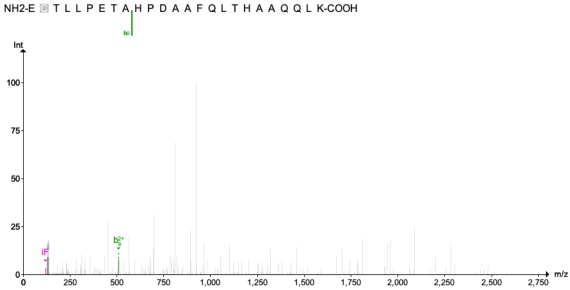
NH2-E C T L L P E T A H P D A A F Q L T H A A Q Q L K-COOH
b9
Int
100
75
50
25
0
iF
b_9^{2+}
0
250
500
750
1,000
1,250
1,500
1,750
2,000
2,250
2,500
2,750
m/z

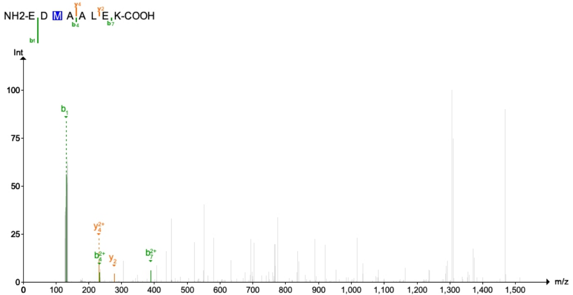

NH2-E D M A A L E K-COOH
b1
b4
b7
y4
y2
Int
100
75
50
25
0
b1
y4 2+
b4 2+
y2
b7 2+
0
100
200
300
400
500
600
700
800
900
1,000
1,100
1,200
1,300
1,400
1,500
m/z

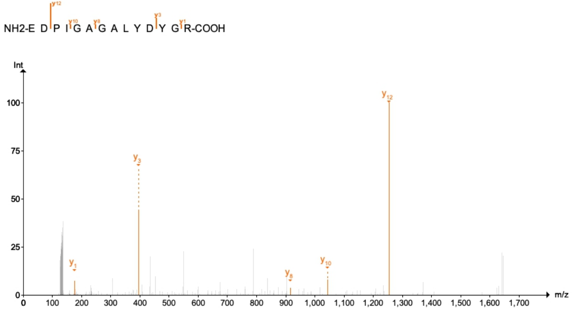

NH2-E D P I G A G A L Y D Y G R-COOH
y12
y10
y8
y3
y1
Int
100
75
50
25
0
m/z
0
100
200
300
400
500
600
700
800
900
1,000
1,100
1,200
1,300
1,400
1,500
1,600
1,700

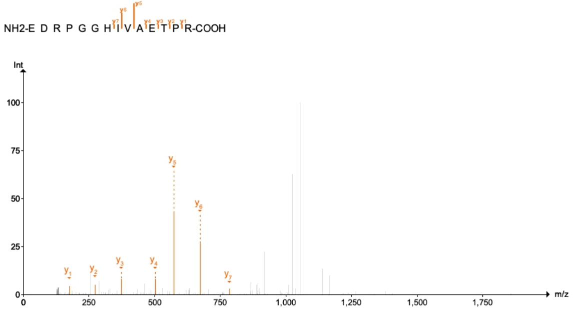

NH2-E D R P G G H I V A E T P R-COOH
y7 y6 y5 y4 y3 y2 y1
Int
100
75
50
25
0
y1
y2
y3
y4
y5
y6
y7
0
250
500
750
1,000
1,250
1,500
1,750
m/z

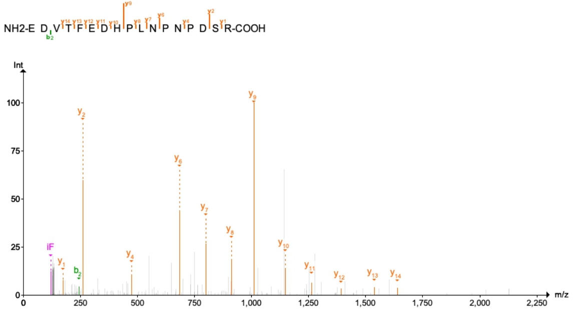

NH2-E D V T F E D H P L N P N P D S R-COOH
b2
y14 y13 y12 y11 y10 y9 y8 y7 y6 y4 y2 y1
Int
100
75
50
25
0
iF
y1
b2
y2
y4
y6
y7
y8
y9
y10
y11
y12
y13
y14
0
250
500
750
1,000
1,250
1,500
1,750
2,000
2,250
m/z

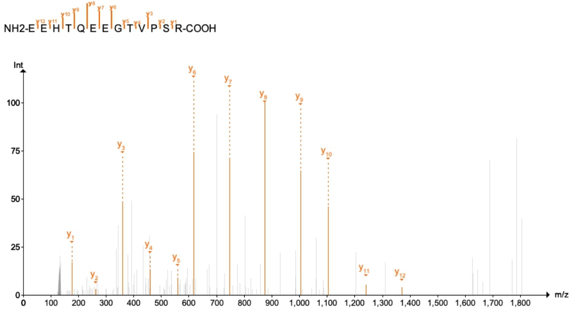
NH2-E E H T Q E E G T V P S R-COOH
Int
m/z
y1
y2
y3
y4
y5
y6
y7
y8
y9
y10
y11
y12

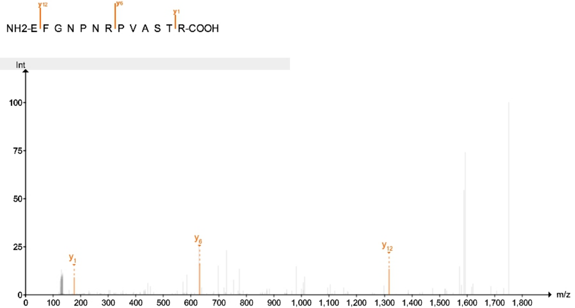

NH2-E F G N P N R P V A S T R-COOH
y12
y6
y1
Int
100
75
50
25
0
0
100
200
300
400
500
600
700
800
900
1,000
1,100
1,200
1,300
1,400
1,500
1,600
1,700
1,800
m/z

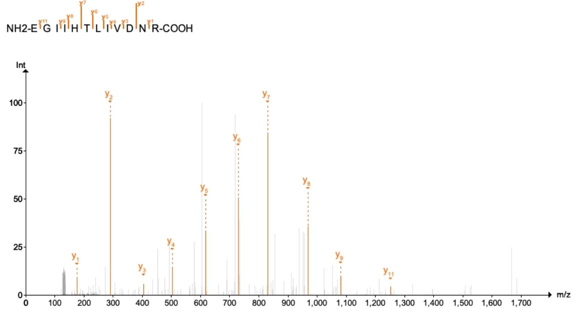

NH2-E G I I H T L I V D N R-COOH
y11 y9 y8 y7 y6 y5 y4 y3 y2 y1
Int
100
75
50
25
0
y1 y2 y3 y4 y5 y6 y7 y8 y9 y11
0 100 200 300 400 500 600 700 800 900 1,000 1,100 1,200 1,300 1,400 1,500 1,600 1,700
m/z

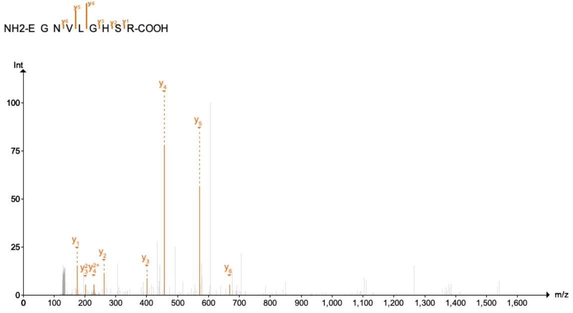

NH2-E G N V L G H S R-COOH
Int
y4
y5
y1
y2
y3
y6
100
75
50
25
0
m/z
0
100
200
300
400
500
600
700
800
900
1,000
1,100
1,200
1,300
1,400
1,500
1,600

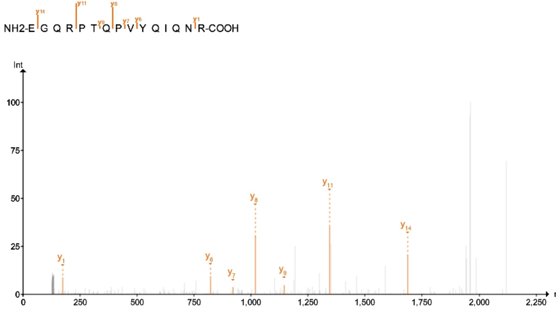

NH2-E G Q R P T Q P V Y Q I Q N R-COOH
y14
y11
y9
y8
y7
y6
y1
Int
100
75
50
25
0
y1
y6
y7
y8
y9
y11
y14
m/z
0
250
500
750
1,000
1,250
1,500
1,750
2,000
2,250

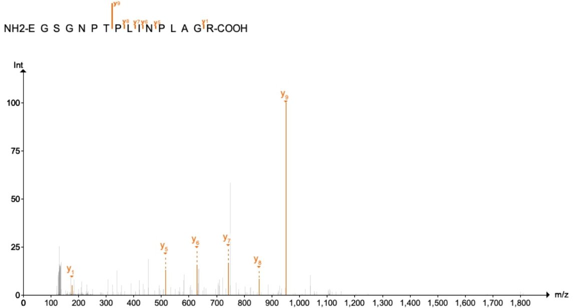
NH2-E G S G N P T P L I N P L A G R-COOH
Int
100
75
50
25
0
y1
y5
y6
y7
y8
y9
m/z
0
100
200
300
400
500
600
700
800
900
1,000
1,100
1,200
1,300
1,400
1,500
1,600
1,700
1,800

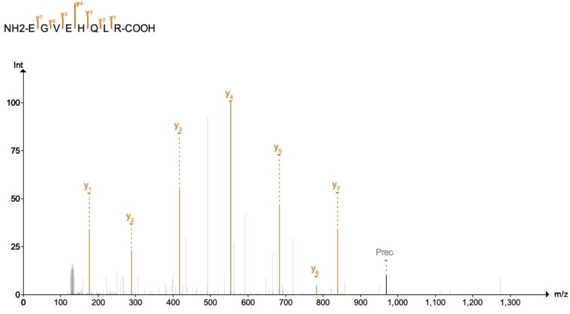

NH2-E G V E H Q L R-COOH
y7
y6
y5
y4
y3
y2
y1
Int
100
75
50
25
0
y1
y2
y3
y4
y5
y6
y7
Prec
0
100
200
300
400
500
600
700
800
900
1,000
1,100
1,200
1,300
m/z

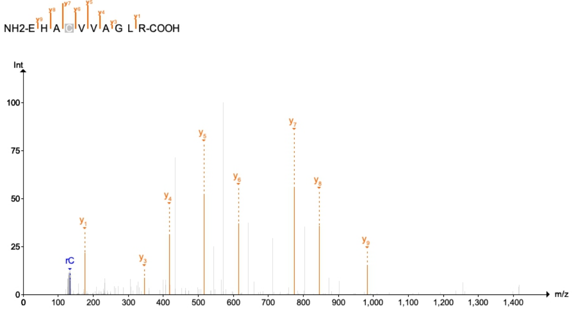
NH2-E H A C V V A G L R-COOH
y9 y8 y7 y6 y5 y4 y3 y1
Int
100
75
50
25
0
rC
y1
y3
y4
y5
y6
y7
y8
y9
0 100 200 300 400 500 600 700 800 900 1,000 1,100 1,200 1,300 1,400
m/z

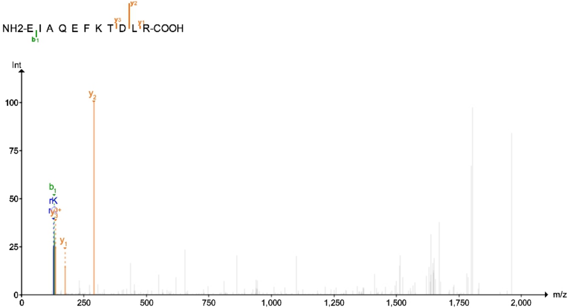
NH2-E I A Q E F K T D L R-COOH
b1
y3
y2
y1
Int
100
75
50
25
0
b1
rK
y2
y1
0
250
500
750
1,000
1,250
1,500
1,750
2,000
m/z

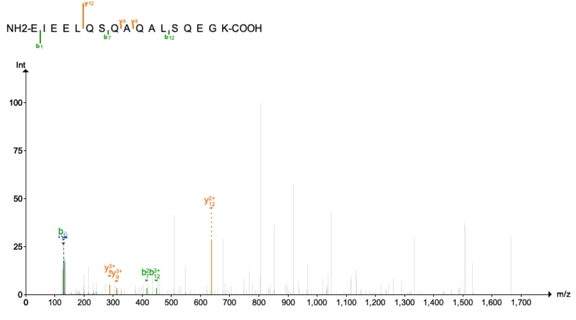

NH2-E I E E L Q S Q A Q A L S Q E G K-COOH
y12
y9
y8
b1
b7
b12
Int
100
75
50
25
0
m/z
0
100
200
300
400
500
600
700
800
900
1,000
1,100
1,200
1,300
1,400
1,500
1,600
1,700

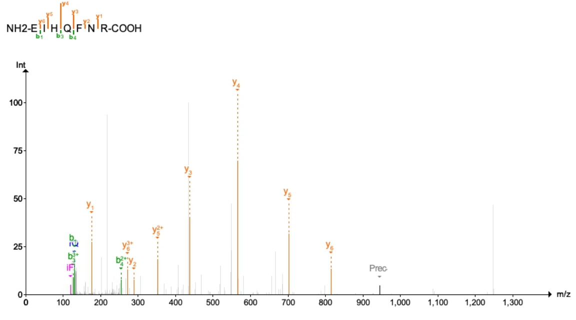

NH2-EIHQFNR-COOH
Int
m/z
Prec
y1
y2
y3
y4
y5
y6
b1
b4
iF

NH2-E I L I N T L H K-COOH

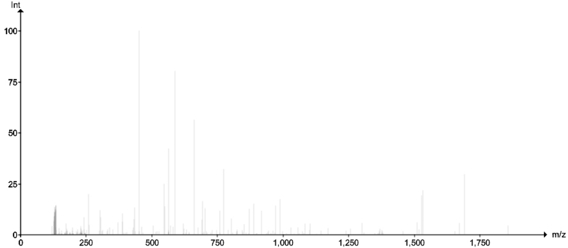

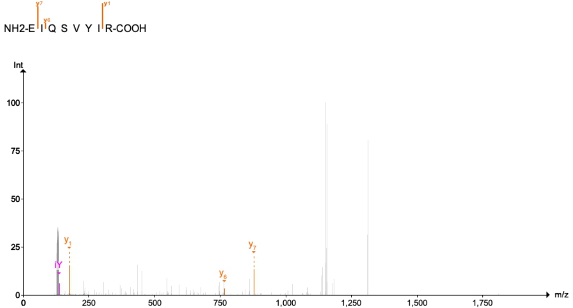
NH2-E I Q S V Y I R-COOH
y7
y6
y1
Int
100
75
50
25
0
y1
iY
y6
y7
0
250
500
750
1,000
1,250
1,500
1,750
m/z

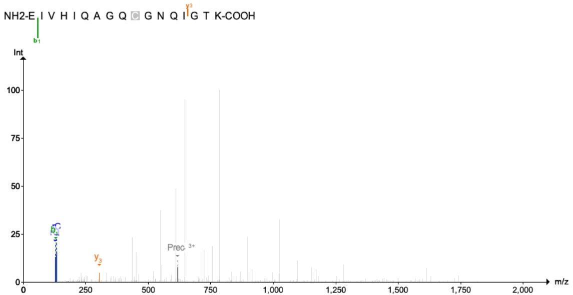

NH2-E I V H I Q A G Q C G N Q I G T K-COOH
b1
y3
Int
100
75
50
25
0
Prec 3+
m/z
0
250
500
750
1,000
1,250
1,500
1,750
2,000

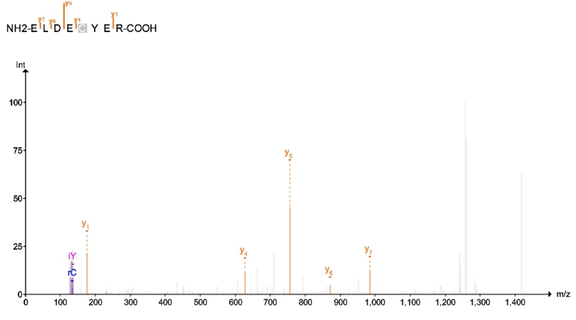
NH2-E L D E C Y E R-COOH
y7
y6
y5
y4
y1
Int
100
75
50
25
0
y5
y1
y4
y6
y7
iY
rC
0
100
200
300
400
500
600
700
800
900
1,000
1,100
1,200
1,300
1,400
m/z

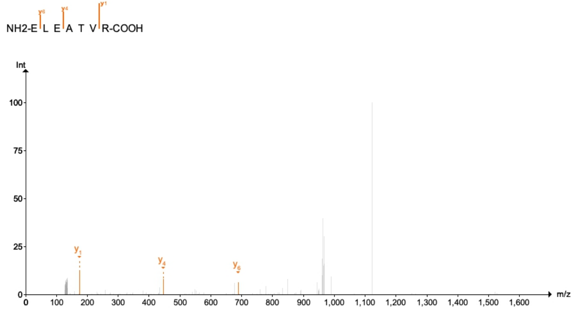

NH2-E L E A T V R-COOH
y6
y4
y1
Int
100
75
50
25
0
y1
y4
y6
m/z
0
100
200
300
400
500
600
700
800
900
1,000
1,100
1,200
1,300
1,400
1,500
1,600

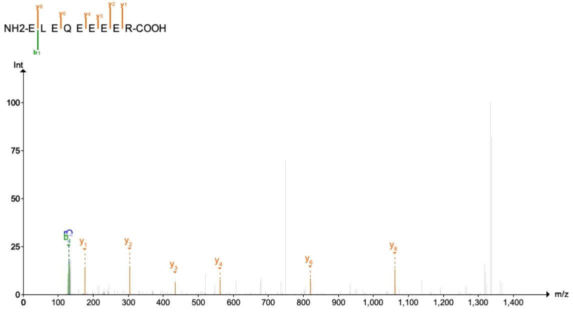

NH2-E L E Q E E E E R-COOH
y8
y6
y4
y3
y2
y1
b1
Int
100
75
50
25
0
b4
y1
y2
y3
y4
y6
y8
0
100
200
300
400
500
600
700
800
900
1,000
1,100
1,200
1,300
1,400
m/z

NH2-E L F S N L Q E F A G P S G K-COOH

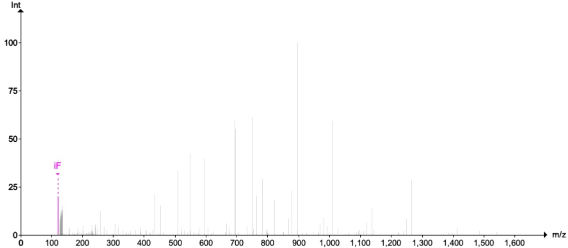

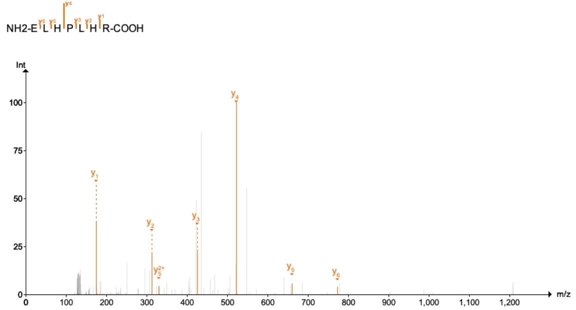

NH2-ELHPLHR-COOH
y6 y5 y4 y3 y2 y1
Int
m/z
y1
y2
y5 2+
y3
y4
y5
y6
0 25 50 75 100
0 100 200 300 400 500 600 700 800 900 1,000 1,100 1,200

NH2-E L I S F L S E P E I L V K-COOH

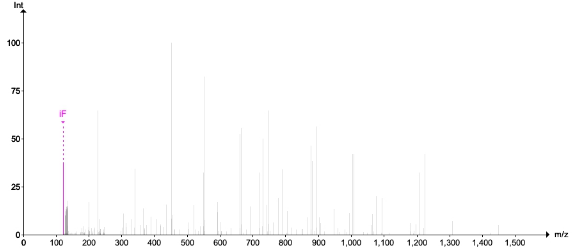

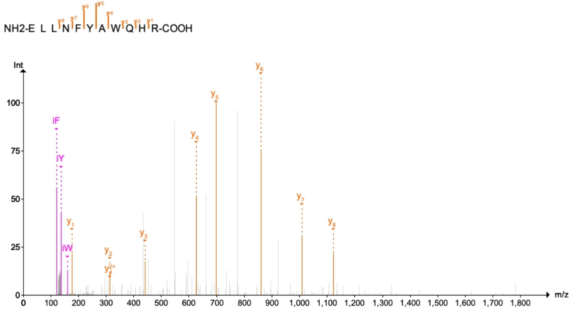

NH2-E L L N F Y A W Q H R-COOH
y8 y7 y6 y5 y4 y3 y2 y1
Int
m/z
iF
iY
iW
y1
y2
y2 2+
y3
y4
y5
y6
y7
y8

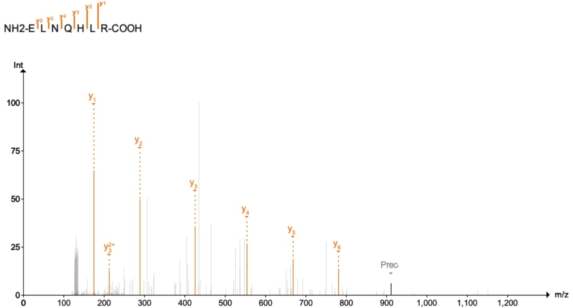

NH2-E L N Q H L R-COOH
y6
y5
y4
y3
y2
y1
Int
m/z
y1
y2
y3
y3 2+
y4
y5
y6
Prec
0
25
50
75
100
0
100
200
300
400
500
600
700
800
900
1,000
1,100
1,200

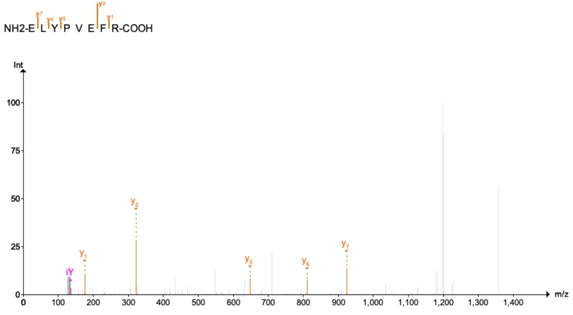
NH2-E L Y P V E F R-COOH
y7
y6
y5
y2
y1
Int
100
75
50
25
0
y2
y1
iY
y5
y6
y7
0
100
200
300
400
500
600
700
800
900
1,000
1,100
1,200
1,300
1,400
m/z

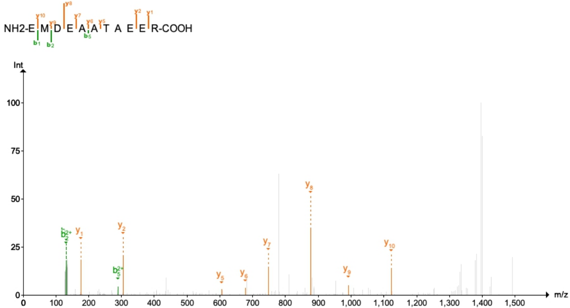

NH2-E M D E A A T A E E R-COOH
y10
y9
y8
y7
y6
y5
y2
y1
b1
b2
b5
Int
100
75
50
25
0
b2 2+
y1
y2
b5 2+
y5
y6
y7
y8
y9
y10
m/z
0
100
200
300
400
500
600
700
800
900
1,000
1,100
1,200
1,300
1,400
1,500

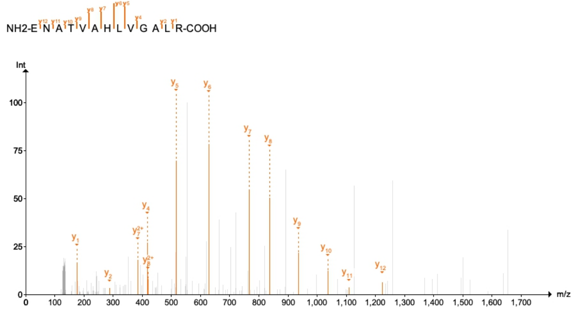
NH2-E N A T V A H L V G A L R-COOH
y12 y11 y10 y9 y8 y7 y6 y5 y4 y2 y1
Int
100
75
50
25
0
y5
y6
y7
y8
y4
y7 2+
y8 2+
y1
y2
y9
y10
y11
y12
m/z
0 100 200 300 400 500 600 700 800 900 1,000 1,100 1,200 1,300 1,400 1,500 1,600 1,700

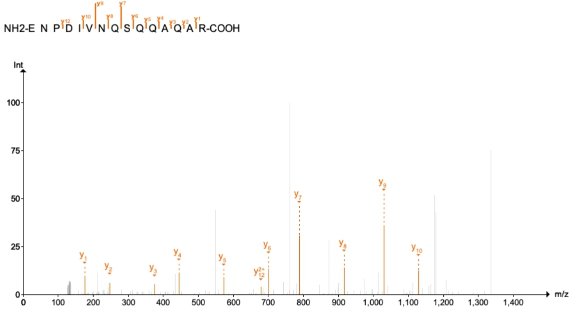
NH2-E N P D I V N Q S Q Q A Q A R-COOH
y12
y10
y9
y8
y7
y6
y5
y4
y3
y2
y1
Int
100
75
50
25
0
y1
y2
y3
y4
y5
y6
y7
y8
y9
y10
y12 2+
m/z
0
100
200
300
400
500
600
700
800
900
1,000
1,100
1,200
1,300
1,400

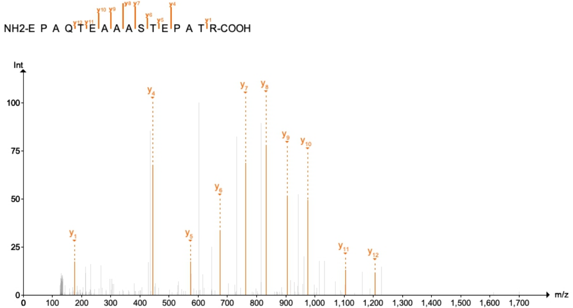

NH2-E P A Q T E A A A S T E P A T R-COOH
y12 y11 y10 y9 y8 y7 y6 y5 y4 y1
Int
y1 y4 y5 y6 y7 y8 y9 y10 y11 y12
0
25
50
75
100
0 100 200 300 400 500 600 700 800 900 1,000 1,100 1,200 1,300 1,400 1,500 1,600 1,700
m/z

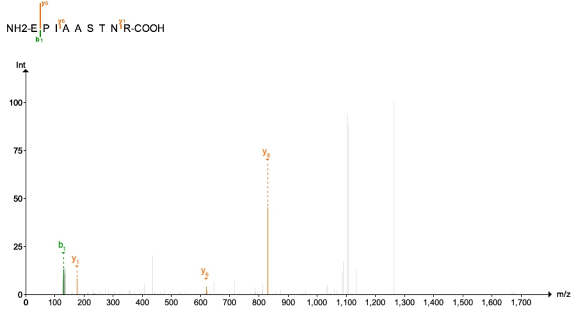

NH2-E P I A A S T N R-COOH
y8
y6
y1
b1
Int
100
75
50
25
0
b1
y1
y6
y8
0 100 200 300 400 500 600 700 800 900 1,000 1,100 1,200 1,300 1,400 1,500 1,600 1,700
m/z

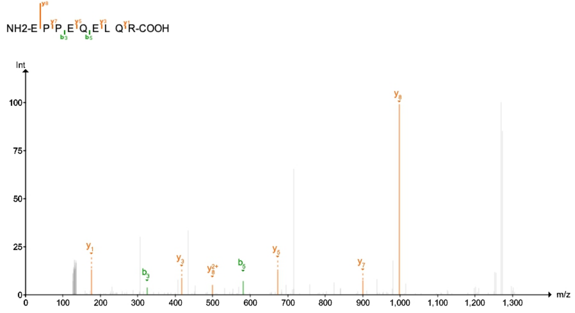
NH2-E P P E Q E L Q R-COOH
y8 y7 y5 y3 y1
b3 b5
Int
100
75
50
25
0
y1 b3 y3 y8 2+ b5 y5 y7 y8
0 100 200 300 400 500 600 700 800 900 1,000 1,100 1,200 1,300
m/z

NH2-E Q C N P E T R P V E K-COOH

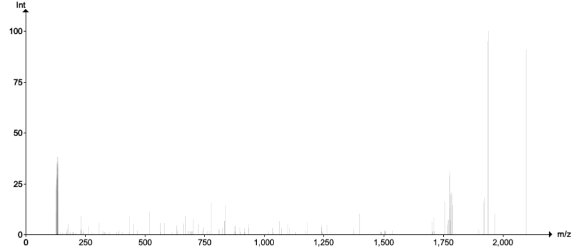

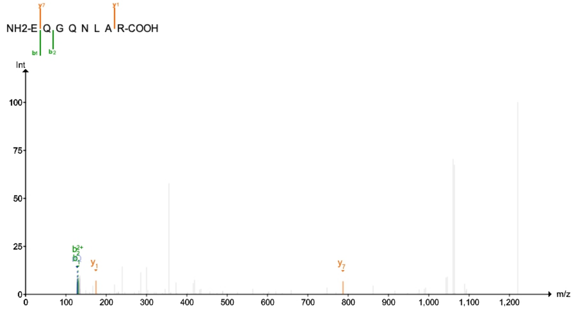

NH2-E Q G Q N L A R-COOH
y7
y1
b1
b2
Int
100
75
50
25
0
b2 2+
b1
y1
y7
0
100
200
300
400
500
600
700
800
900
1,000
1,100
1,200
m/z

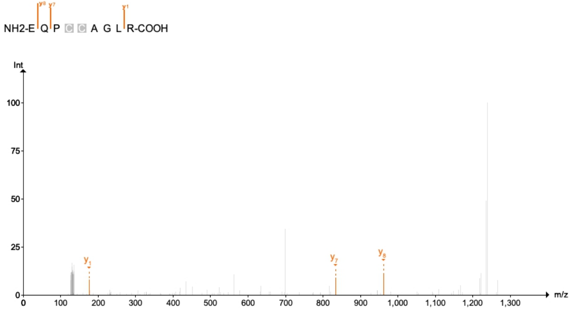

y8
y7
y1
NH2-E Q P C C A G L R-COOH
Int
100
75
50
25
0
y1
y7
y8
0
100
200
300
400
500
600
700
800
900
1,000
1,100
1,200
1,300
m/z

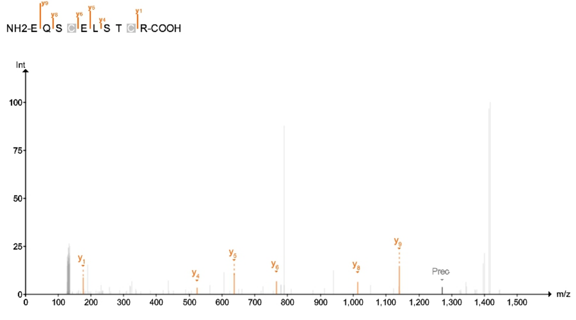

NH2-E Q S C E L S T C R-COOH
y9
y8
y6
y5
y4
y1
Int
100
75
50
25
0
y1
y4
y5
y6
y8
y9
Prec
0
100
200
300
400
500
600
700
800
900
1,000
1,100
1,200
1,300
1,400
1,500
m/z

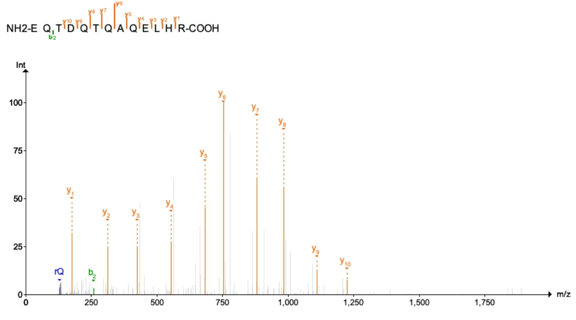

NH2-E Q T D Q T Q A Q E L H R-COOH
b2
y10 y9 y8 y7 y6 y5 y4 y3 y2 y1
Int
m/z
rQ

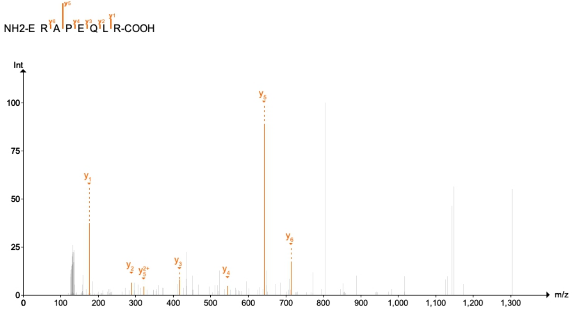
NH2-E R A P E Q L R-COOH
y6
y5
y4
y3
y2
y1
Int
100
75
50
25
0
y1
y2
y5 2+
y3
y4
y5
y6
0
100
200
300
400
500
600
700
800
900
1,000
1,100
1,200
1,300
m/z

NH2-E R E E Y L A Q Q K-COOH

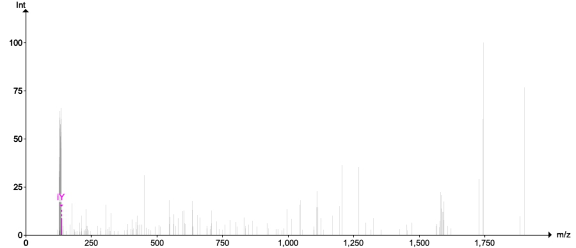

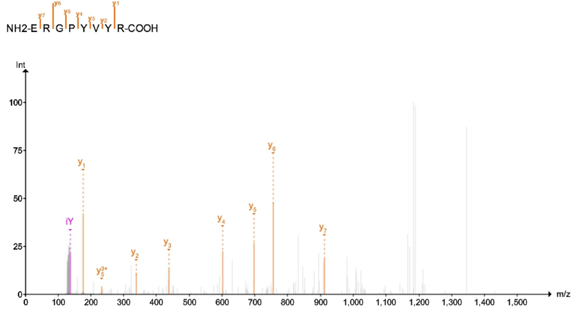
NH2-E R G P Y V Y R-COOH
y7
y6
y5
y4
y3
y2
y1
Int
100
75
50
25
0
iY
y1
y2
y3
y4
y5
y6
y7
y5 3+
m/z
0
100
200
300
400
500
600
700
800
900
1,000
1,100
1,200
1,300
1,400
1,500

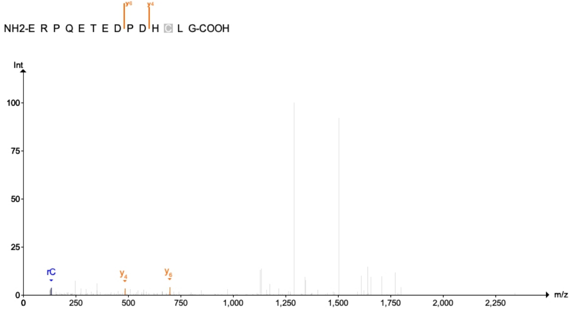
NH2-E R P Q E T E D P D H C L G-COOH
y6
y4
Int
100
75
50
25
0
rC
y4
y6
0
250
500
750
1,000
1,250
1,500
1,750
2,000
2,250
m/z

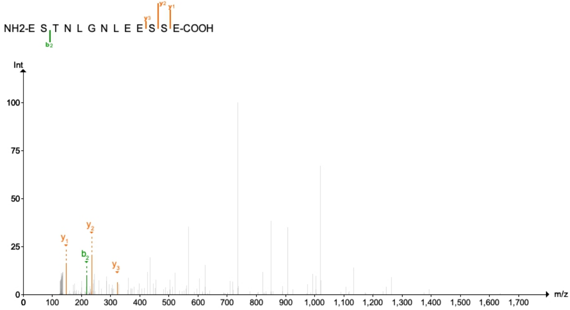
NH2-E S T N L G N L E E S S S E-COOH
b2
y3
y2
y1
Int
m/z
0
25
50
75
100
0
100
200
300
400
500
600
700
800
900
1,000
1,100
1,200
1,300
1,400
1,500
1,600
1,700

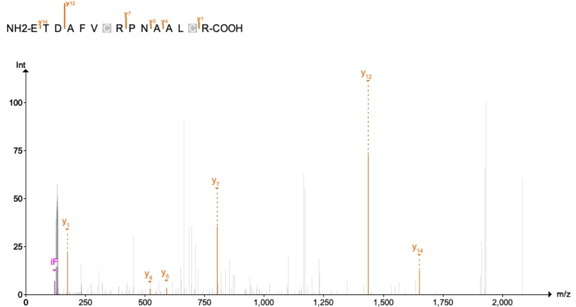

NH2-E T D A F V C R P N A A L C R-COOH
y14
y12
y7
y5
y4
y1
Int
m/z
y1
y4
y5
y7
y12
y14
iF
0
25
50
75
100
0
250
500
750
1,000
1,250
1,500
1,750
2,000

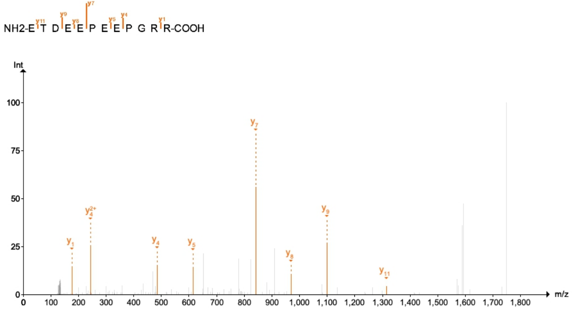

NH2-E T D E E P E E P G R R-COOH
y11
y9
y8
y7
y5
y4
y1
Int
100
75
50
25
0
y7
y4 2+
y9
y1
y4
y5
y8
y11
0
100
200
300
400
500
600
700
800
900
1,000
1,100
1,200
1,300
1,400
1,500
1,600
1,700
1,800
m/z

NH2-E T D G T E G T V E I E T V K-COOH

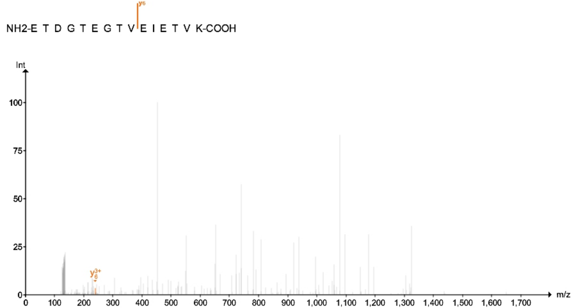

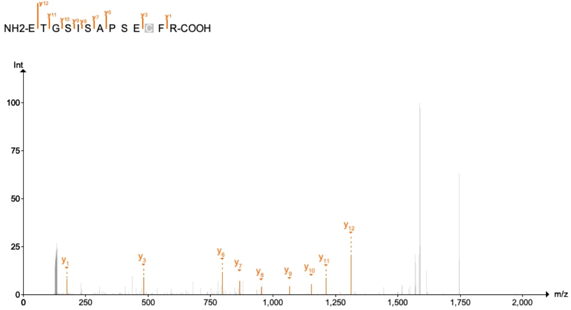
NH2-E T G S I S A P S E C F R-COOH
y12
y11
y10
y9
y8
y7
y6
y3
y1
Int
100
75
50
25
0
250
500
750
1,000
1,250
1,500
1,750
2,000
m/z

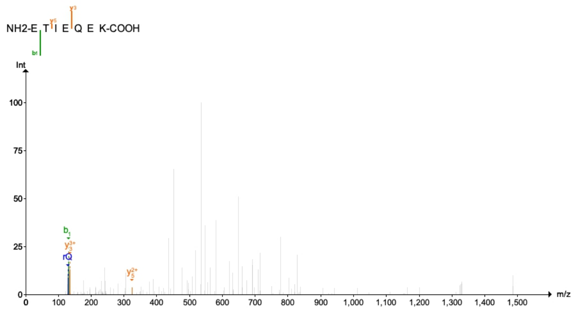

NH2-E T I E Q E K-COOH
b1
y5
y3
Int
100
75
50
25
0
b1
y3 3+
rQ
y5 2+
0
100
200
300
400
500
600
700
800
900
1,000
1,100
1,200
1,300
1,400
1,500
m/z

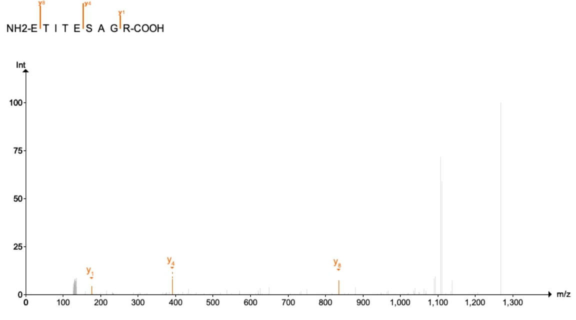

NH2-E T I T E S A G R-COOH
y8
y4
y1
Int
100
75
50
25
0
0
100
200
300
400
500
600
700
800
900
1,000
1,100
1,200
1,300
m/z

NH2-E T L P S I W D S P T K-COOH

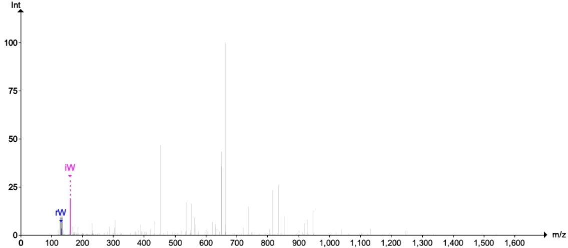

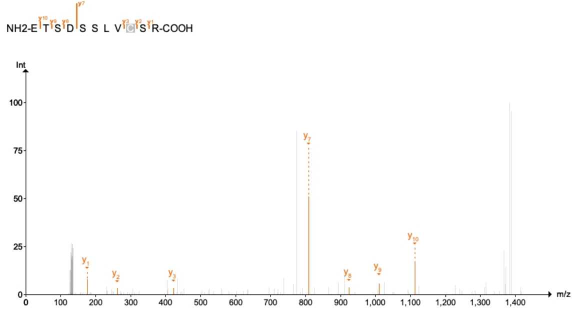

NH2-E T S D S S L V C S R-COOH
y10 y9 y8 y7 y3 y2 y1
Int
100
75
50
25
0
y1
y2
y3
y7
y8
y9
y10
0 100 200 300 400 500 600 700 800 900 1,000 1,100 1,200 1,300 1,400
m/z

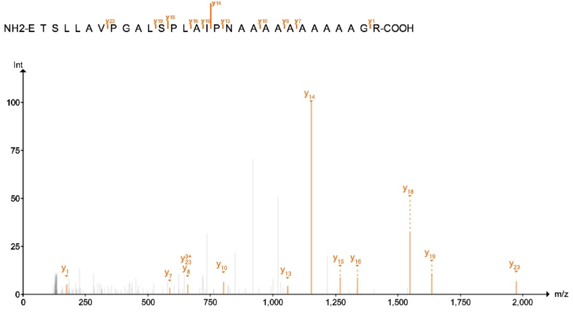
NH2-E T S L L A V P G A L S P L A I P N A A A A A A A A A A G R-COOH
y23 y19 y18 y16 y15 y14 y13 y10 y8 y7 y1
Int
m/z
y14
y18
y1
y7
y8
y23 3+
y10
y13
y15
y16
y19
y23
0
25
50
75
100
0
250
500
750
1,000
1,250
1,500
1,750
2,000

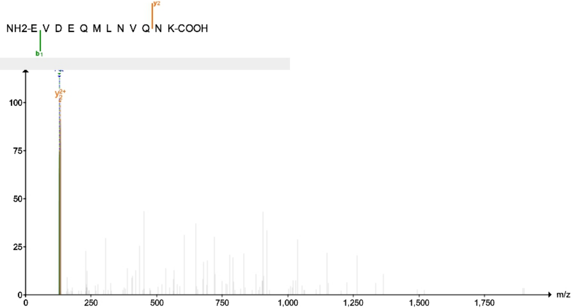

NH2-E V D E Q M L N V Q N K-COOH
y2
b1
y_2^{2+}
m/z

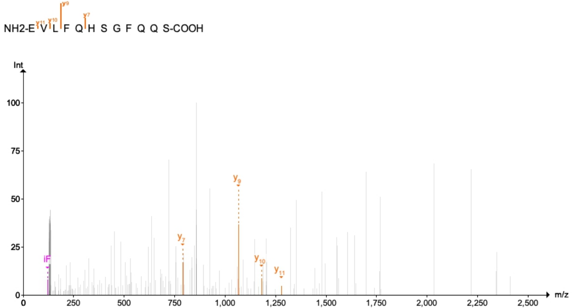
NH2-E V L F Q H S G F Q Q S-COOH
y11
y10
y9
y7
Int
100
75
50
25
0
m/z
250
500
750
1,000
1,250
1,500
1,750
2,000
2,250
2,500
iF
y7
y9
y10
y11

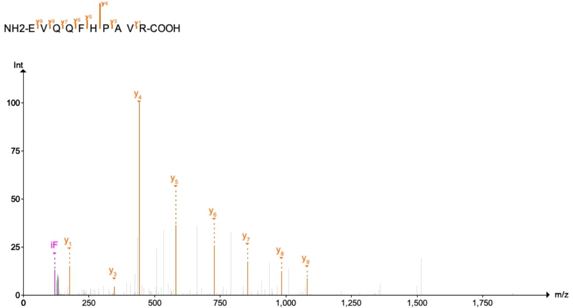

NH2-E V Q Q F H P A V R-COOH
y9 y8 y7 y6 y5 y4 y3 y1
Int
100
75
50
25
0
250
500
750
1,000
1,250
1,500
1,750
m/z
iF
y1
y3
y4
y5
y6
y7
y8
y9

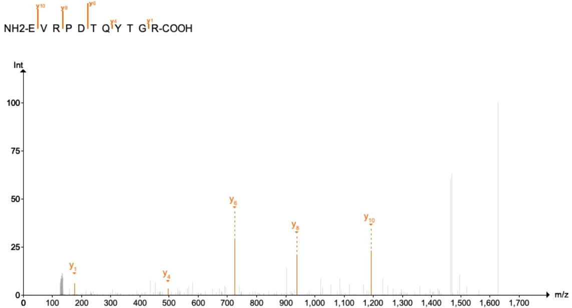

NH2-E V R P D T Q Y T G R-COOH
y10
y8
y6
y4
y1
Int
100
75
50
25
0
m/z
0
100
200
300
400
500
600
700
800
900
1,000
1,100
1,200
1,300
1,400
1,500
1,600
1,700

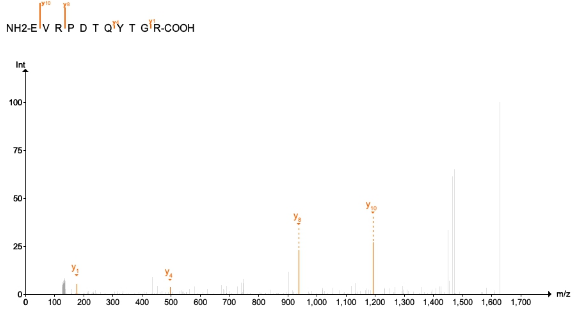

y10
y8
y4
y1
NH2-E V R P D T Q Y T G R-COOH
Int
100
75
50
25
0
y1
y4
y8
y10
0
100
200
300
400
500
600
700
800
900
1,000
1,100
1,200
1,300
1,400
1,500
1,600
1,700
m/z

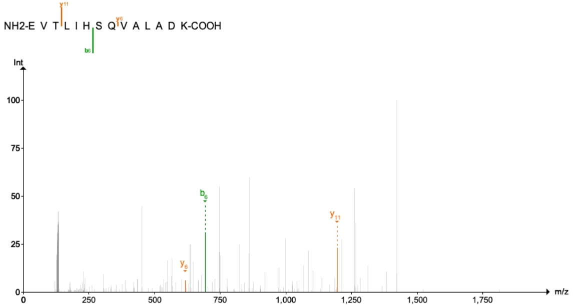

NH2-E V T L I H S Q V A L A D K-COOH
y11
y6
b6
Int
100
75
50
25
0
0
250
500
750
1,000
1,250
1,500
1,750
m/z

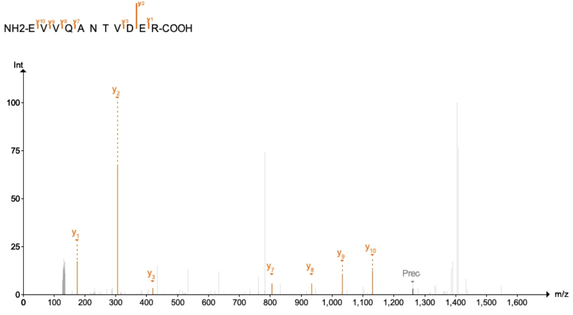

NH2-E V V Q A N T V D E R-COOH
y10 y9 y8 y7 y3 y2 y1
Int
100
75
50
25
0
y2
y1
y3
y7
y8
y9
y10
Prec
0 100 200 300 400 500 600 700 800 900 1,000 1,100 1,200 1,300 1,400 1,500 1,600
m/z

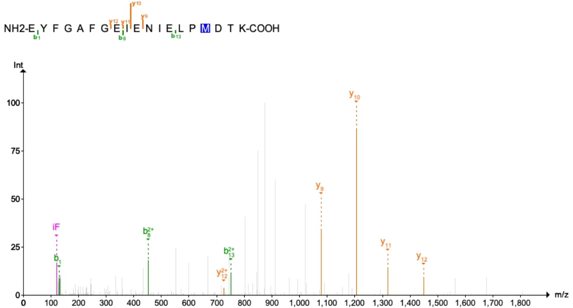
NH2-E Y F G A F G E I E N I E L P M D T K-COOH
b1
b8
b13
y12
y11
y10
y9
Int
100
75
50
25
0
iF
b1
b8 2+
y12 2+
b13 2+
y9
y10
y11
y12
0
100
200
300
400
500
600
700
800
900
1,000
1,100
1,200
1,300
1,400
1,500
1,600
1,700
1,800
m/z

NH2-E Y L T G F H K-COOH

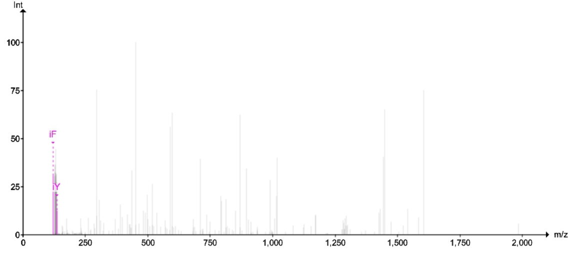

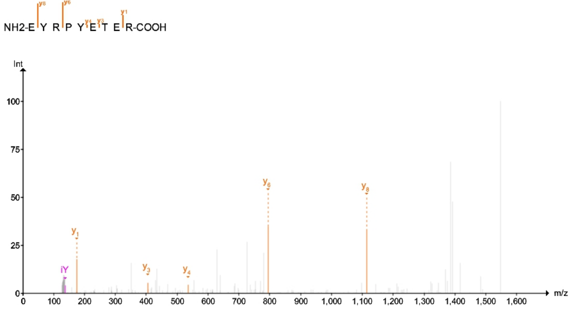

NH2-E Y R P Y E T E R-COOH
y8
y6
y4
y3
y1
Int
100
75
50
25
0
y1
y3
y4
y6
y8
iY
m/z
0
100
200
300
400
500
600
700
800
900
1,000
1,100
1,200
1,300
1,400
1,500
1,600

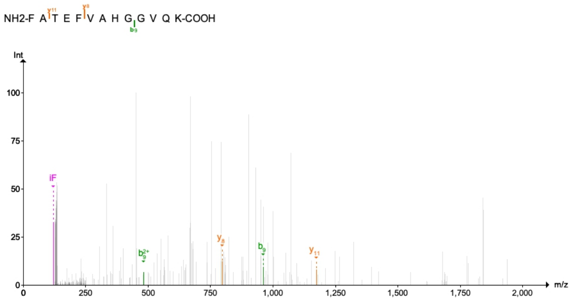

NH2-F A T E F V A H G G V Q K-COOH
y11
y8
b9
Int
100
75
50
25
0
iF
b9 2+
y8
b9
y11
m/z
0
250
500
750
1,000
1,250
1,500
1,750
2,000

NH2-F D A Y F H Q K-COOH

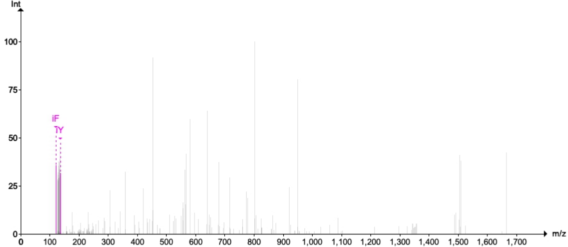

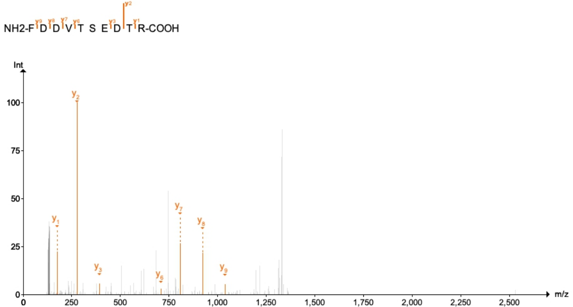

NH2-F D D V T S E D T R-COOH
y9 y8 y7 y6 y3 y2 y1
Int
m/z
y1
y2
y3
y6
y7
y8
y9
0
25
50
75
100
250
500
750
1,000
1,250
1,500
1,750
2,000
2,250
2,500

NH2-F D L L E E L V A K-COOH

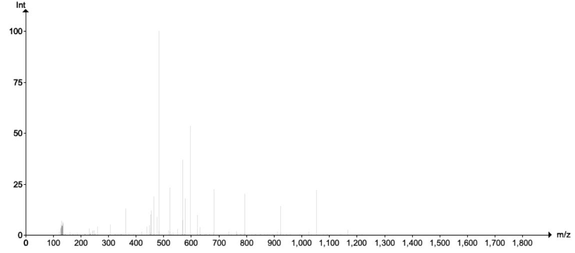

NH2-F F T D D L F Q Y A G E K-COOH

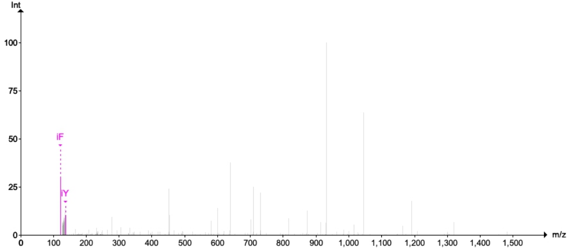

NH2-F G G V Q E L L N Q Q K-COOH

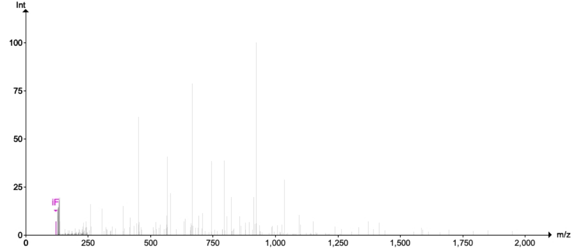

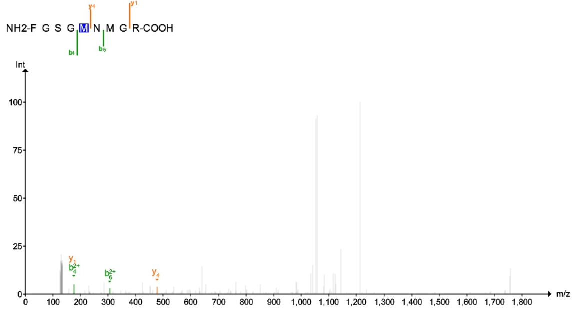

NH2-F G S G M N M G R-COOH
y4
y1
b4
b6
Int
100
75
50
25
0
y1
b_4^{2+}
b_6^{2+}
y4
0 100 200 300 400 500 600 700 800 900 1,000 1,100 1,200 1,300 1,400 1,500 1,600 1,700 1,800
m/z

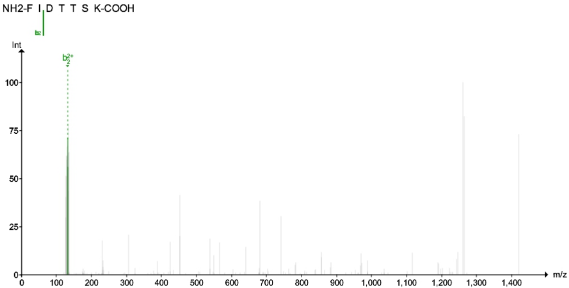

NH2-F I D T T S K-COOH
b2
Int
b$_2^{2+}$
100
75
50
25
0
0
100
200
300
400
500
600
700
800
900
1,000
1,100
1,200
1,300
1,400
m/z

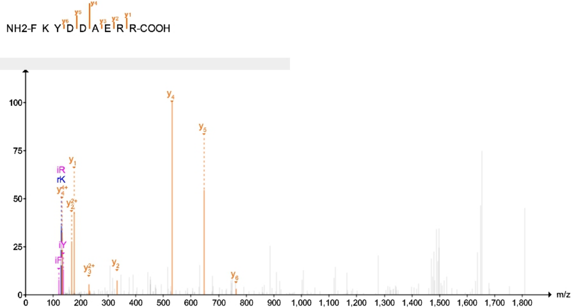

NH2-F K Y D D A E R R-COOH
y6
y5
y4
y3
y2
y1
100
75
50
25
0
y4
y5
y1
iR
rK
y4 4+
y2 2+
iY
iF
y3 2+
y2
y6
m/z
0
100
200
300
400
500
600
700
800
900
1,000
1,100
1,200
1,300
1,400
1,500
1,600
1,700
1,800

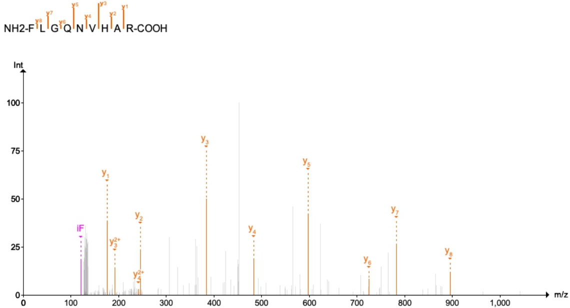

NH2-F L G Q N V H A R-COOH
y8 y7 y6 y5 y4 y3 y2 y1
Int
m/z
y1
y2
y3
y4
y5
y6
y7
y8
y3 2+
y4 2+
iF
0
25
50
75
100
100
200
300
400
500
600
700
800
900
1,000

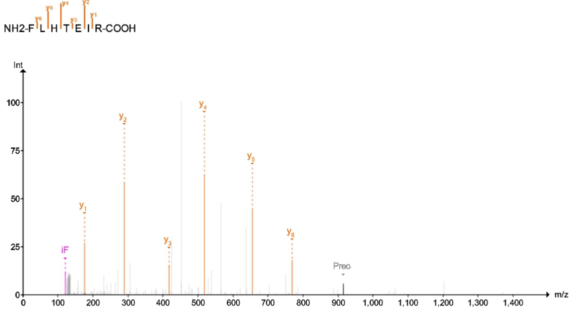

NH2-F L H T E I R-COOH
y6 y5 y4 y3 y2 y1
Int
100
75
50
25
0
y2
y4
y5
y1
y3
y6
iF
Prec
0 100 200 300 400 500 600 700 800 900 1,000 1,100 1,200 1,300 1,400
m/z

NH2-F N A H H G D T K-COOH

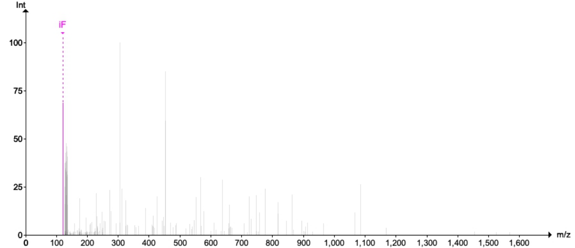

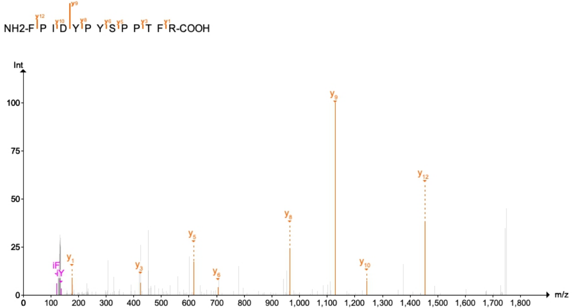

NH2-F P I D Y P Y S P P T F R-COOH
y12
y10
y9
y8
y6
y5
y3
y1
Int
100
75
50
25
0
y9
y12
y8
y5
y10
y6
y3
y1
iF
-Y
m/z
0
100
200
300
400
500
600
700
800
900
1,000
1,100
1,200
1,300
1,400
1,500
1,600
1,700
1,800

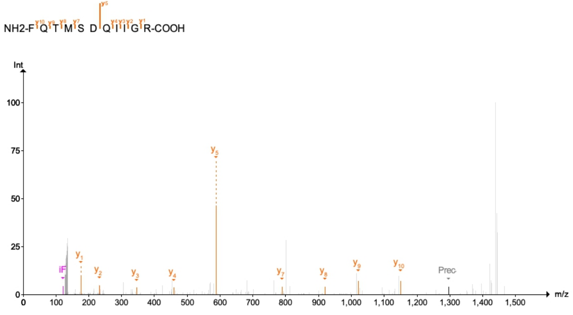

NH2-FQTMS DQIIGR-COOH
y10 y9 y8 y7 y5 y4 y3 y2 y1
Int
100
75
50
25
0
iF
y1
y2
y3
y4
y5
y7
y8
y9
y10
Prec
0 100 200 300 400 500 600 700 800 900 1,000 1,100 1,200 1,300 1,400 1,500
m/z

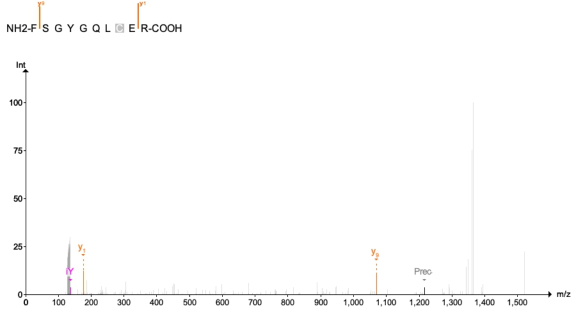
NH2-F S G Y G Q L C E R-COOH
y9
y1
Int
m/z
Prec
IY
0
25
50
75
100
0
100
200
300
400
500
600
700
800
900
1,000
1,100
1,200
1,300
1,400
1,500

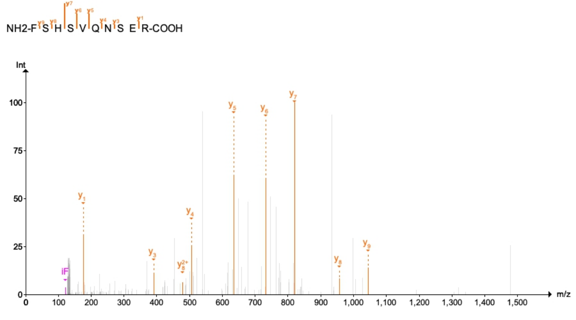

NH2-F S H S V Q N S E R-COOH
y9 y8 y7 y6 y5 y4 y3 y1
Int
m/z
y1
y3
y8 2+
y4
y5
y6
y7
y8
y9
iF
0
25
50
75
100
0
100
200
300
400
500
600
700
800
900
1,000
1,100
1,200
1,300
1,400
1,500

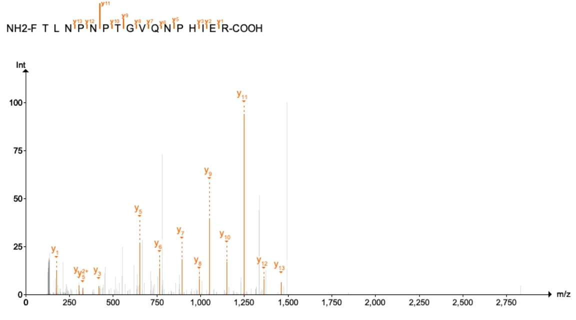
NH2-F T L N P N P T G V Q N P H I E R-COOH
y13 y12 y11 y10 y9 y8 y7 y6 y5 y3 y2 y1
Int
100
75
50
25
0
y1
y_5^{2+}
y3
y5
y6
y7
y8
y9
y10
y11
y12
y13
0
250
500
750
1,000
1,250
1,500
1,750
2,000
2,250
2,500
2,750
m/z

NH2-F V G I K D E D Q L E A F L K-COOH

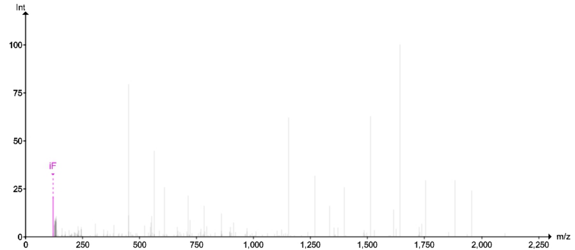

NH2-F Y P W T I D N K-COOH

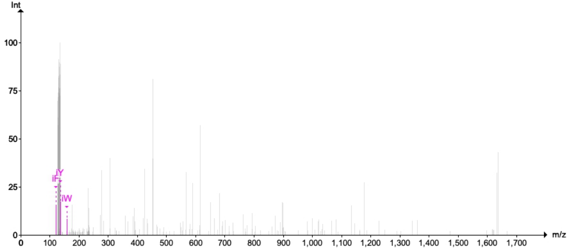

NH2-G A H Y C V P C Y E N K-COOH

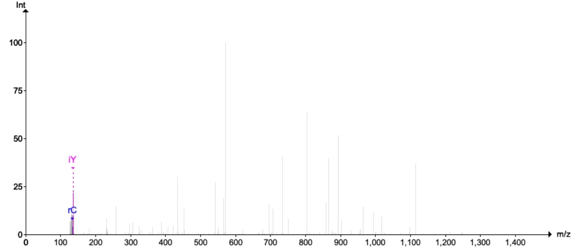

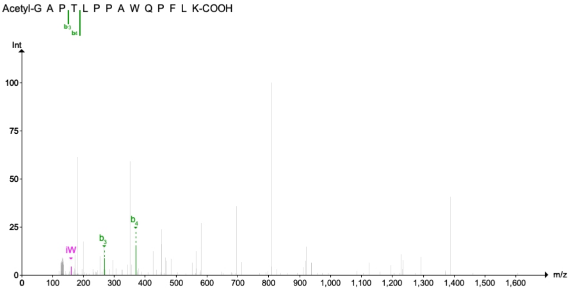
Acetyl-G A P T L P P A W Q P F L K-COOH
b3
b4
Int
100
75
50
25
0
iW
b3
b4
0
100
200
300
400
500
600
700
800
900
1,000
1,100
1,200
1,300
1,400
1,500
1,600
m/z

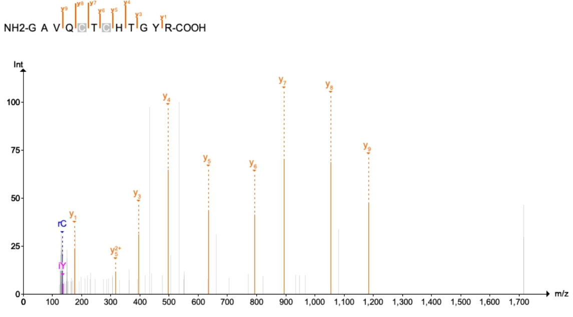
NH2-G A V Q C T C H T G Y R-COOH
y9 y8 y7 y6 y5 y4 y3 y1
Int
m/z
rC
iY
y1
y3
y4
y5
y6
y7
y8
y9
y5 2+
0
25
50
75
100
0
100
200
300
400
500
600
700
800
900
1,000
1,100
1,200
1,300
1,400
1,500
1,600
1,700

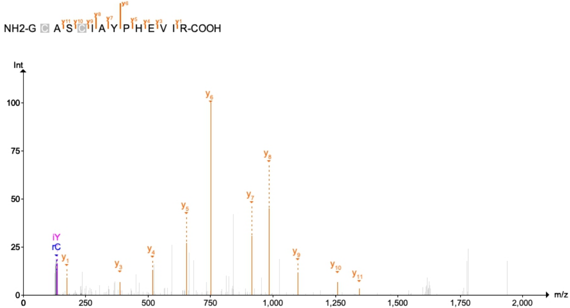

NH2-G C A S C I A Y P H E V I R-COOH
y11 y10 y9 y8 y7 y6 y5 y4 y3 y1
Int
100
75
50
25
0
iY
rC
y1
y3
y4
y5
y6
y7
y8
y9
y10
y11
0
250
500
750
1,000
1,250
1,500
1,750
2,000
m/z

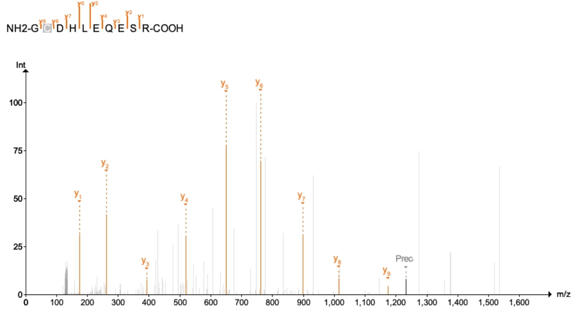

NH2-G C D H L E Q E S R-COOH
y9 y8 y7 y6 y5 y4 y3 y2 y1
Int
m/z
y1
y2
y3
y4
y5
y6
y7
y8
y9
Prec
0
25
50
75
100
0
100
200
300
400
500
600
700
800
900
1,000
1,100
1,200
1,300
1,400
1,500
1,600

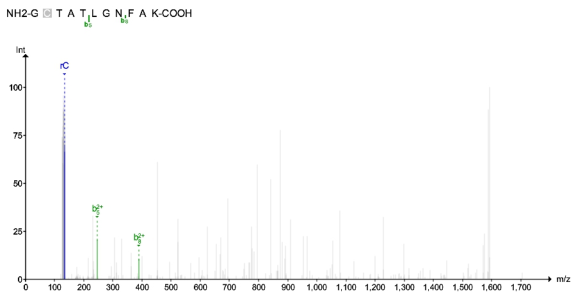
NH2-G C T A T L G N F A K-COOH
b5
b8
Int
rC
b5 2+
b8 2+
m/z

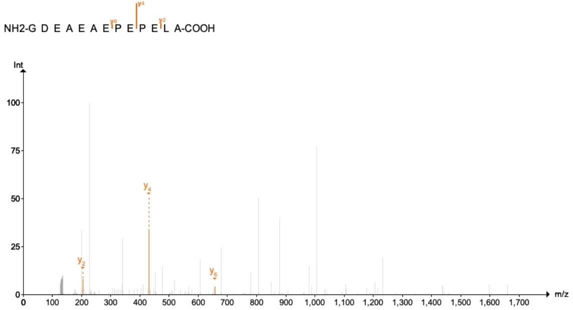

NH2-G D E A E A E P E P E L A-COOH
y6
y4
y2
Int
100
75
50
25
0
y4
y2
y6
0
100
200
300
400
500
600
700
800
900
1,000
1,100
1,200
1,300
1,400
1,500
1,600
1,700
m/z

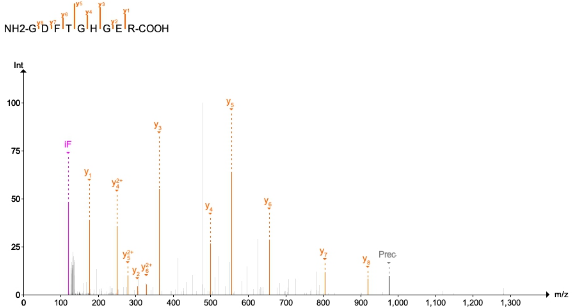
NH2-G D F T G H G E R-COOH
y8 y7 y6 y5 y4 y3 y2 y1
Int
100
75
50
25
0
iF
y1
y4 2+
y3
y5
y4
y6
y5 2+
y2
y6 2+
y7
y8
Prec
0
100
200
300
400
500
600
700
800
900
1,000
1,100
1,200
1,300
m/z

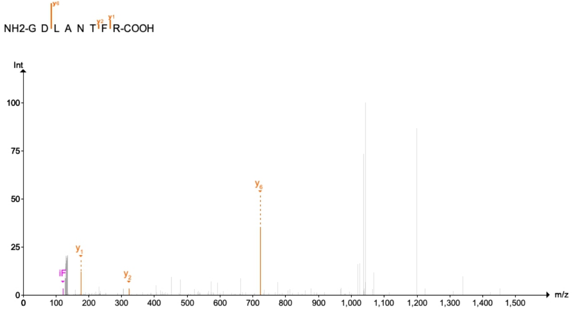
NH2-G D L A N T F R-COOH
y6
y2
y1
Int
100
75
50
25
0
y6
y1
iF
y2
0
100
200
300
400
500
600
700
800
900
1,000
1,100
1,200
1,300
1,400
1,500
m/z

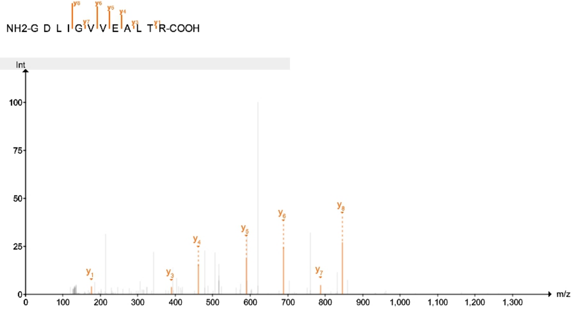

NH2-G D L I G V V E A L T R-COOH
y8
y7
y6
y5
y4
y3
y1
Int
m/z

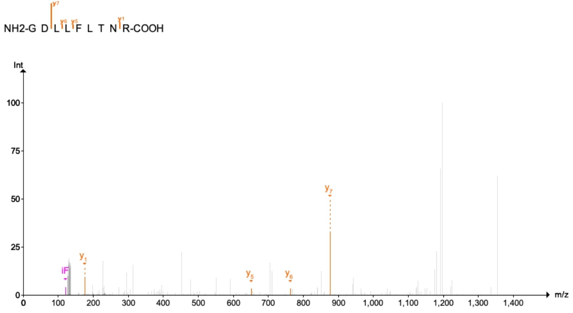

NH2-G D L L F L T N R-COOH
y7
y6
y5
y1
Int
100
75
50
25
0
iF
y1
y5
y6
y7
0
100
200
300
400
500
600
700
800
900
1,000
1,100
1,200
1,300
1,400
m/z

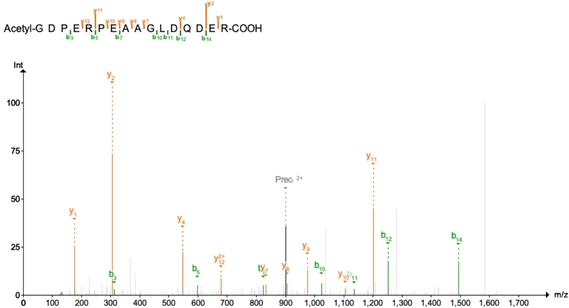

Acetyl-G D P E R P E A A G L D Q D E R-COOH
Int
m/z
Prec 2+
y1
y2
y4
y7
y8
y9
y10
y11
y12 2+
b3
b5
b10
b11
b12
b14
0
25
50
75
100
0, 100, 200, 300, 400, 500, 600, 700, 800, 900, 1,000, 1,100, 1,200, 1,300, 1,400, 1,500, 1,600, 1,700

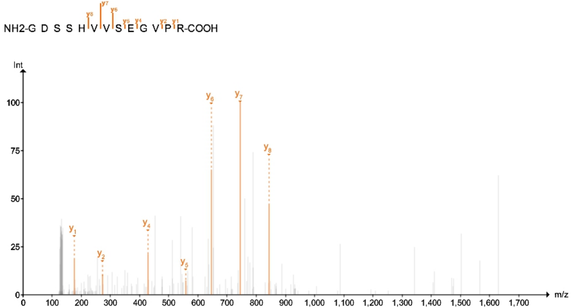

NH2-G D S S H V V S E G V P R-COOH
y8
y7
y6
y5
y4
y2
y1
Int
100
75
50
25
0
y1
y2
y4
y5
y6
y7
y8
0
100
200
300
400
500
600
700
800
900
1,000
1,100
1,200
1,300
1,400
1,500
1,600
1,700
m/z

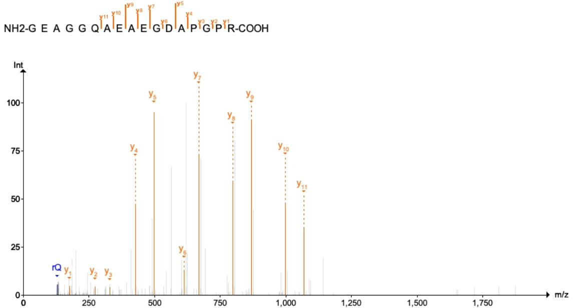

NH2-G E A G G Q A E A E G D A P G P R-COOH
Int
m/z
rQ
y1
y2
y3
y4
y5
y6
y7
y8
y9
y10
y11
0
25
50
75
100
0
250
500
750
1,000
1,250
1,500
1,750

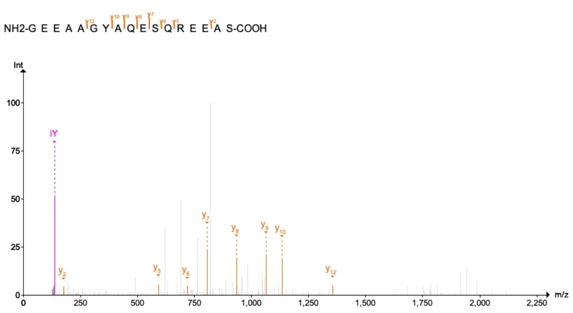
NH2-G E E A A G Y A Q E S Q R E E A S-COOH
y12
y10
y9
y8
y7
y6
y5
y2
Int
iY
y2
y5
y6
y7
y8
y9
y10
y12
m/z
0
25
50
75
100
0
250
500
750
1,000
1,250
1,500
1,750
2,000
2,250

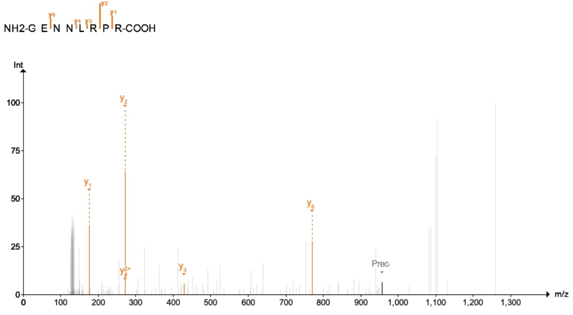
NH2-G E N N L R P R-COOH
y6
y4
y3
y2
y1
Int
100
75
50
25
0
y1
y2
y3
y4 2+
y6
Prec
m/z
100
200
300
400
500
600
700
800
900
1,000
1,100
1,200
1,300

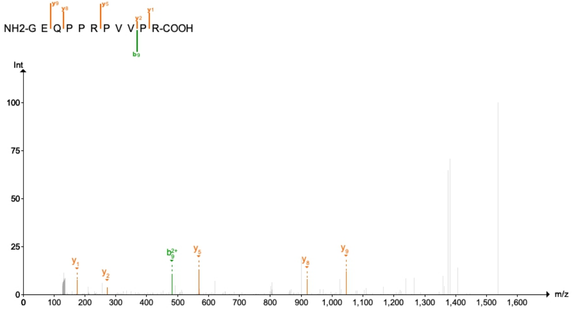

NH2-G E Q P P R P V V P R-COOH
y9
y8
y5
y2
y1
b9
Int
100
75
50
25
0
y1
y2
b9 2+
y5
y8
y9
m/z
0
100
200
300
400
500
600
700
800
900
1,000
1,100
1,200
1,300
1,400
1,500
1,600

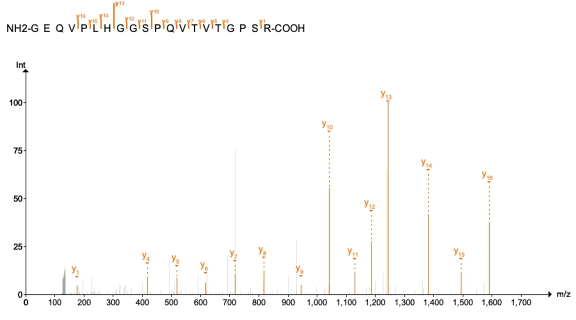

NH2-G E Q V P L H G G S P Q V T V T G P S R-COOH
y16 y15 y14 y13 y12 y11 y10 y9 y8 y7 y6 y5 y4 y1
Int
100
75
50
25
0
y1 y4 y5 y6 y7 y8 y9 y10 y11 y12 y13 y14 y15 y16
0 100 200 300 400 500 600 700 800 900 1,000 1,100 1,200 1,300 1,400 1,500 1,600 1,700
m/z

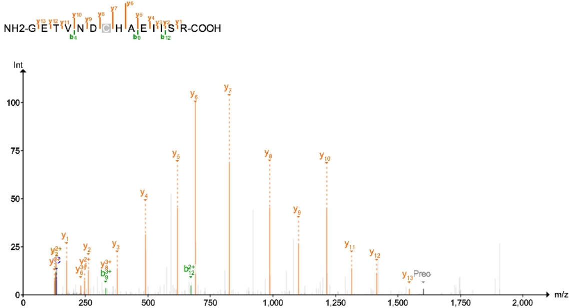
NH2-G E T V N D C H A E I I S R-COOH
Int
m/z
Prec

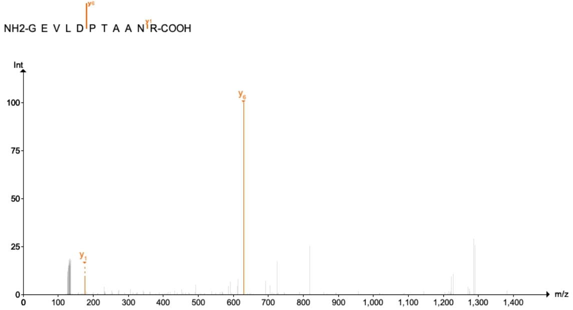

NH2-G E V L D P T A A N R-COOH
y6
y1
Int
y6
y1
m/z

NH2-G F G L L G S I F G K-COOH

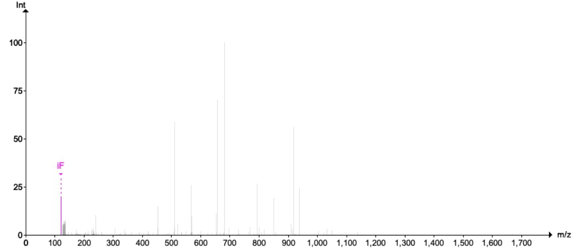

NH2-G G L G L I L K-COOH

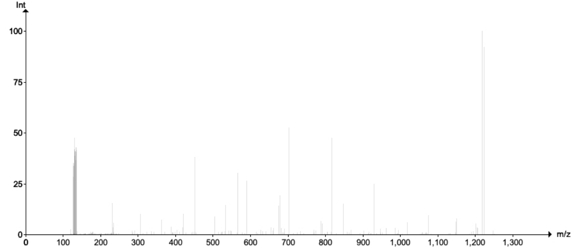

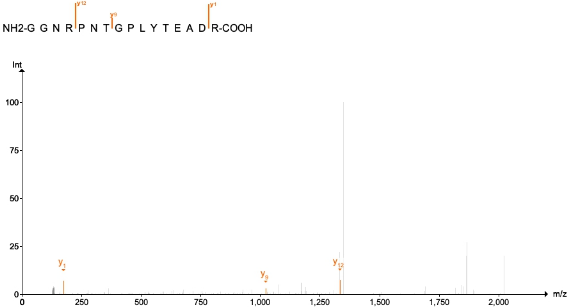

NH2-G G N R P N T G P L Y T E A D R-COOH
y12
y9
y1
Int
100
75
50
25
0
0
250
500
750
1,000
1,250
1,500
1,750
2,000
m/z

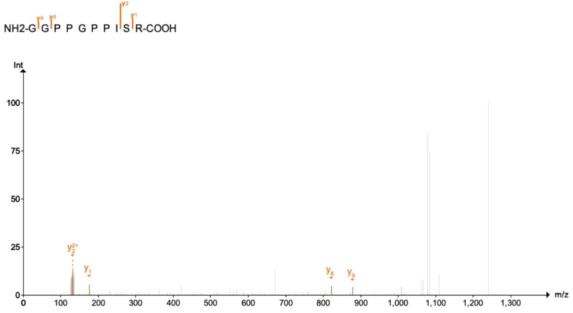

NH2-G G P P G P P I S R-COOH
y9
y8
y2
y1
Int
100
75
50
25
0
y_2^{2+}
y_1
y_8
y_9
0
100
200
300
400
500
600
700
800
900
1,000
1,100
1,200
1,300
m/z

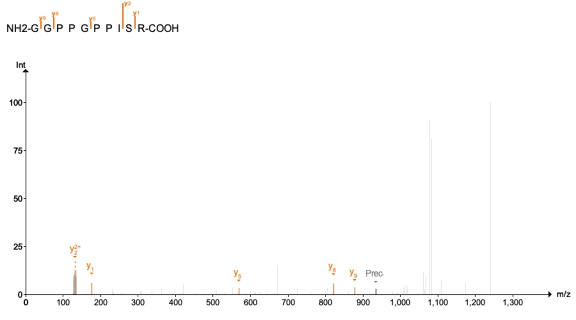
NH2-G G P P G P P I S R-COOH
y9
y8
y5
y2
y1
Int
100
75
50
25
0
y_2^{2+}
y_1
y_5
y_8
y_9
Prec
0
100
200
300
400
500
600
700
800
900
1,000
1,100
1,200
1,300
m/z

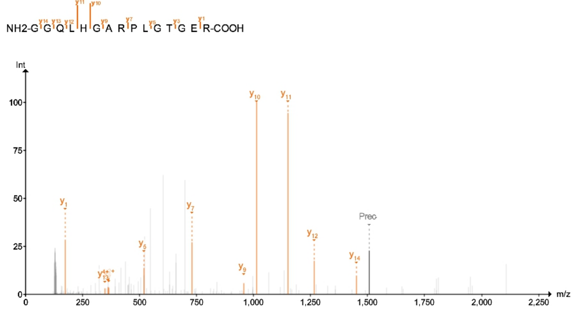

NH2-G G Q L H G A R P L G T G E R-COOH
y14 y13 y12 y11 y10 y9 y7 y5 y3 y1
Int
100
75
50
25
0
y10
y11
y1
y7
Prec
y12
y5
y14
y9
y13 4+
m/z
0
250
500
750
1,000
1,250
1,500
1,750
2,000
2,250

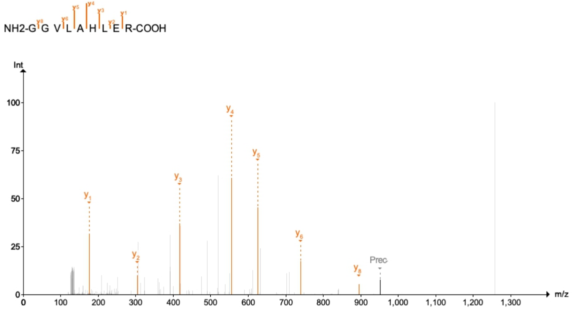
NH2-G G V L A H L E R-COOH
y8
y6
y5
y4
y3
y2
y1
Int
100
75
50
25
0
y1
y2
y3
y4
y5
y6
y8
Prec
0
100
200
300
400
500
600
700
800
900
1,000
1,100
1,200
1,300
m/z

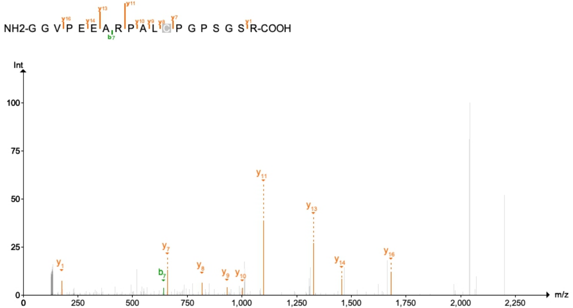

NH2-G G V P E E A R P A L C P G P S G S R-COOH
y16
y14
y13
b7
y11
y10
y9
y8
y7
y1
Int
100
75
50
25
0
m/z
0
250
500
750
1,000
1,250
1,500
1,750
2,000
2,250
y1
y7
b7
y8
y9
y10
y11
y13
y14
y16

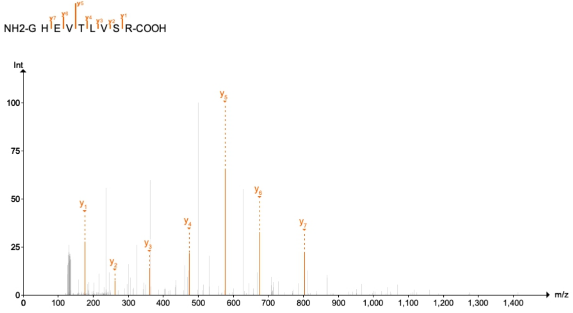
NH2-G H E V T L V S R-COOH
y7
y6
y5
y4
y3
y2
y1
Int
100
75
50
25
0
y1
y2
y3
y4
y5
y6
y7
m/z
0
100
200
300
400
500
600
700
800
900
1,000
1,100
1,200
1,300
1,400

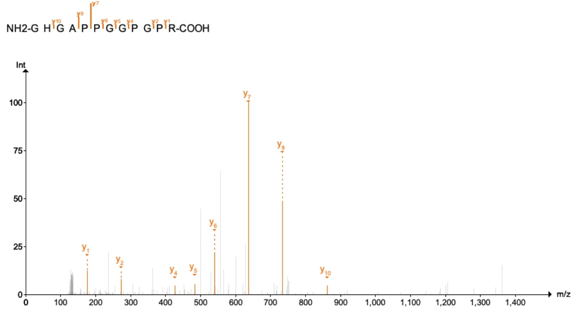

NH2-G H G A P P G G P G P R-COOH
Int
m/z
y1
y2
y4
y5
y6
y7
y8
y10

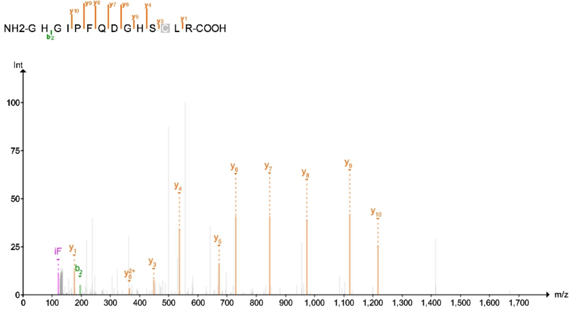

NH2-G H G I P F Q D G H S C L R-COOH
y10 y9 y8 y7 y6 y5 y4 y3 y1
b2
Int
100
75
50
25
0
iF
y1
b2
y6 2+
y3
y4
y5
y6
y7
y8
y9
y10
m/z
0 100 200 300 400 500 600 700 800 900 1,000 1,100 1,200 1,300 1,400 1,500 1,600 1,700

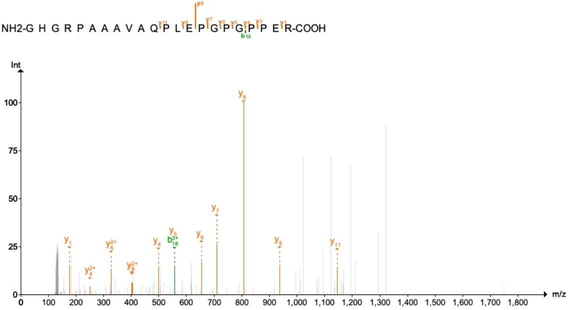

NH2-G H G R P A A A V A Q P L E P G P G P P E R-COOH
Int
m/z
y1
y4 2+
y6 2+
y8 2+
y4
y5
b18 3+
y6
y7
y8
y9
y11
b18

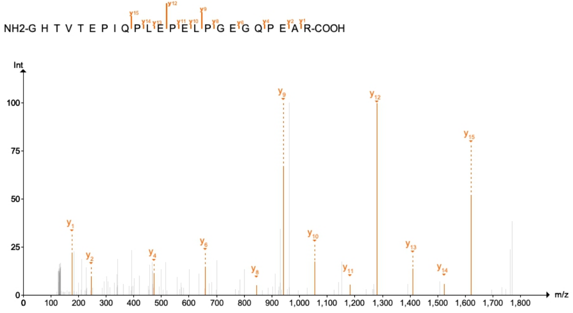
NH2-G H T V T E P I Q P L E P E L P G E G Q P E A R-COOH
Int
m/z
y1
y2
y4
y6
y8
y9
y10
y11
y12
y13
y14
y15
0
25
50
75
100
0
100
200
300
400
500
600
700
800
900
1,000
1,100
1,200
1,300
1,400
1,500
1,600
1,700
1,800

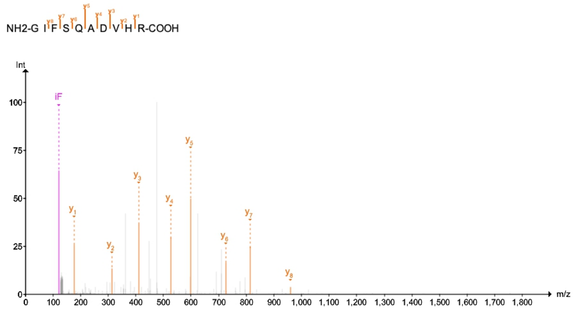
NH2-G I F S Q A D V H R-COOH
Int
iF
y1
y2
y3
y4
y5
y6
y7
y8
m/z

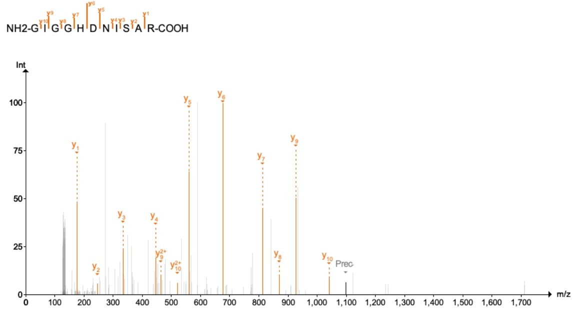

NH2-G I G G H D N I S A R-COOH
Int
m/z
y1
y2
y3
y4
y5
y6
y7
y8
y9
y10
y9 2+
y10 2+
Prec

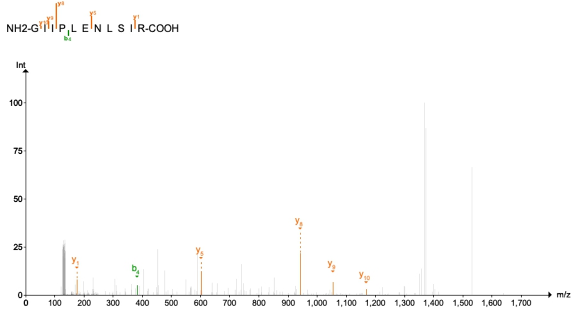
NH2-G I I P L E N L S I R-COOH
y8
y10
y9
b4
y5
y1
Int
100
75
50
25
0
m/z
0
100
200
300
400
500
600
700
800
900
1,000
1,100
1,200
1,300
1,400
1,500
1,600
1,700
y1
b4
y5
y8
y9
y10

NH2-G K Y D C G H V Q K-COOH

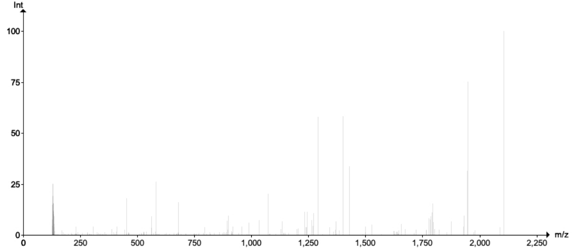

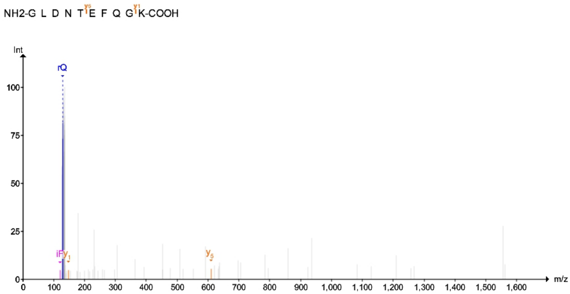
NH2-G L D N T E F Q G K-COOH
y5
y1
Int
rQ
iF
y1
y5
m/z
0
25
50
75
100
0
100
200
300
400
500
600
700
800
900
1,000
1,100
1,200
1,300
1,400
1,500
1,600

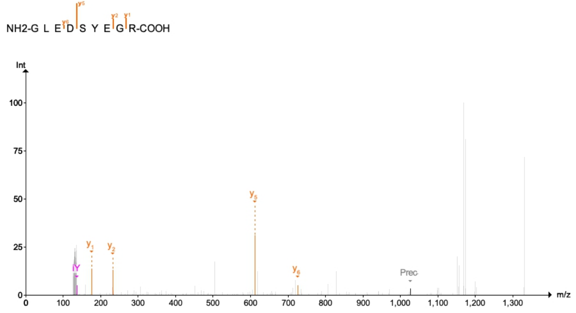

NH2-G L E D S Y E G R-COOH
y6
y5
y2
y1
Int
100
75
50
25
0
y5
y1
y2
y6
IY
Prec
0
100
200
300
400
500
600
700
800
900
1,000
1,100
1,200
1,300
m/z

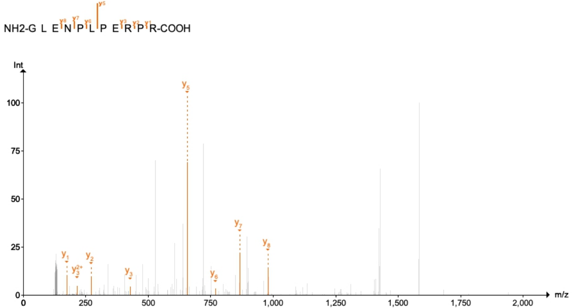

NH2-G L E N P L P E R P R-COOH
y8
y7
y6
y5
y3
y2
y1
Int
100
75
50
25
0
y5
y7
y8
y1
y2
y3 2+
y3
y6
0
250
500
750
1,000
1,250
1,500
1,750
2,000
m/z

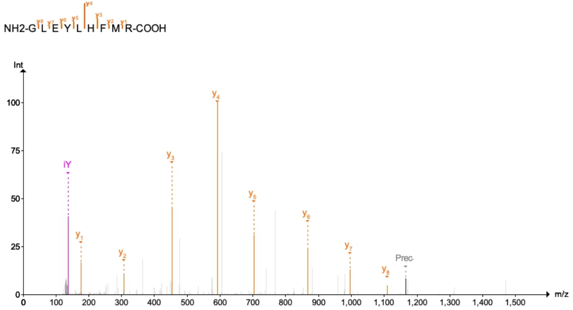

NH2-G L E Y L H F M R-COOH
y8 y7 y6 y5 y4 y3 y2 y1
Int
iY
y1
y2
y3
y4
y5
y6
y7
y8
Prec
m/z
0
25
50
75
100
0 100 200 300 400 500 600 700 800 900 1,000 1,100 1,200 1,300 1,400 1,500

NH2-G L H T V L S K-COOH

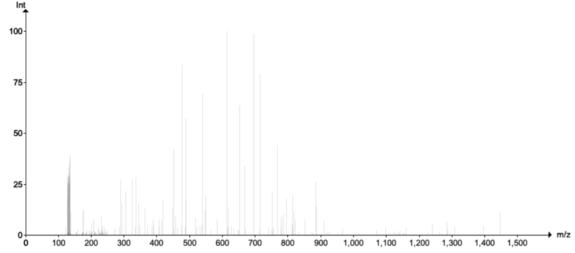

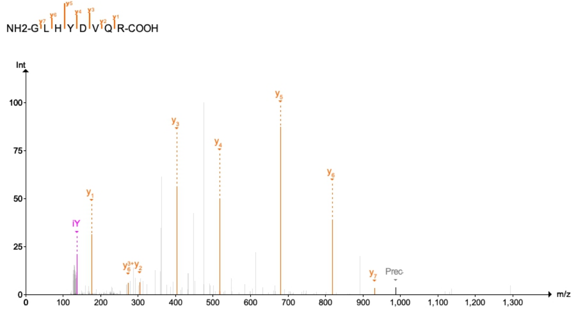

NH2-GLHYDVQR-COOH
y7
y6
y5
y4
y3
y2
y1
Int
100
75
50
25
0
y1
iY
y3
y4
y5
y6
y6 3+ y2
y7
Prec
0
100
200
300
400
500
600
700
800
900
1,000
1,100
1,200
1,300
m/z

NH2-G L L L Y G P P G T G K-COOH

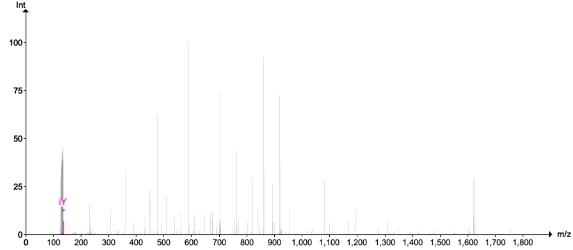

NH2-G L L N D A F Q K-COOH

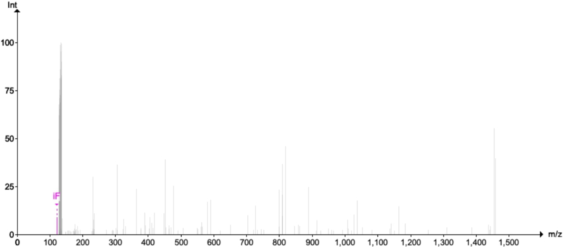

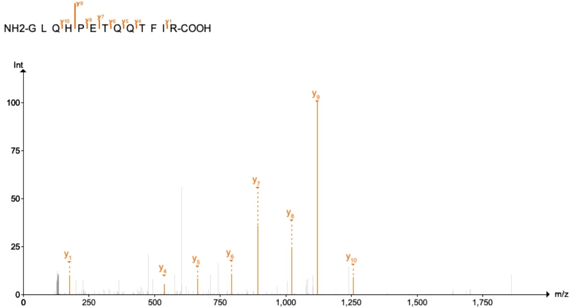

NH2-G L Q H P E T Q Q T F I R-COOH
y9
y10
y8
y7
y6
y5
y4
y1
Int
100
75
50
25
0
250
500
750
1,000
1,250
1,500
1,750
m/z
y1
y4
y5
y6
y7
y8
y9
y10

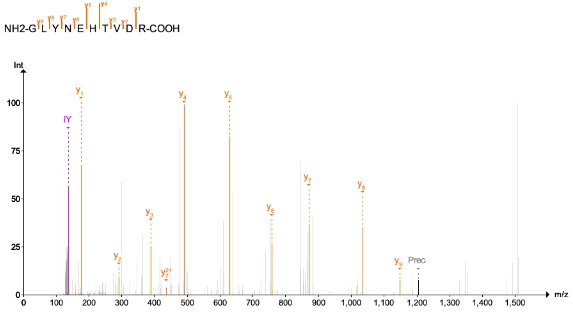
NH2-G L Y N E H T V D R-COOH
y9 y8 y7 y6 y5 y4 y3 y2 y1
Int
100
75
50
25
0
iY
y1
y2
y3
y4
y5
y6
y7
y8
y9
y_7^{2+}
Prec
m/z
0 100 200 300 400 500 600 700 800 900 1,000 1,100 1,200 1,300 1,400 1,500

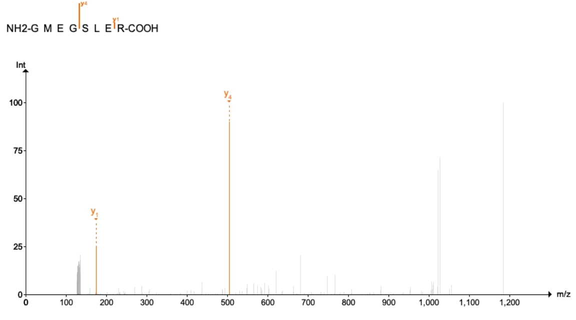
NH2-G M E G S L E R-COOH
y4
y1
Int
y_4
y_1
m/z
0
25
50
75
100
0
100
200
300
400
500
600
700
800
900
1,000
1,100
1,200

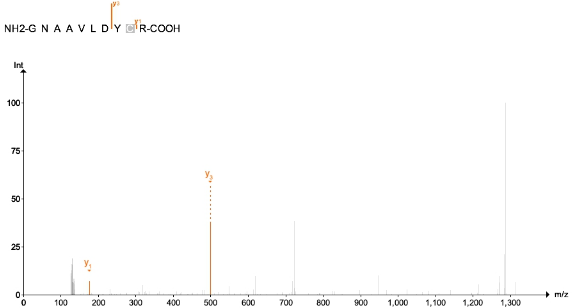

NH2-G N A A V L D Y C R-COOH
y3
y1
Int
100
75
50
25
0
y3
y1
0
100
200
300
400
500
600
700
800
900
1,000
1,100
1,200
1,300
m/z

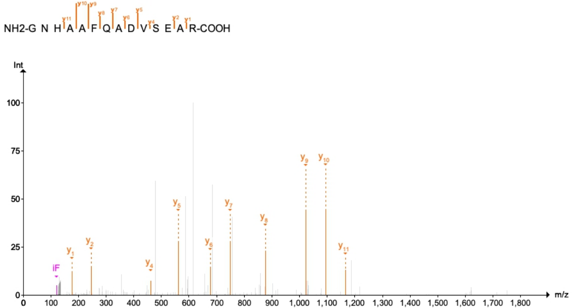
NH2-G N H A A F Q A D V S E A R-COOH
y11 y10 y9 y8 y7 y6 y5 y4 y2 y1
Int
100
75
50
25
0
iF
y1 y2 y4 y5 y6 y7 y8 y9 y10 y11
0 100 200 300 400 500 600 700 800 900 1,000 1,100 1,200 1,300 1,400 1,500 1,600 1,700 1,800
m/z

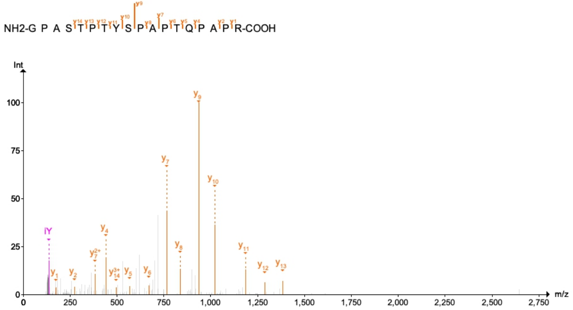

NH2-G P A S T P T Y S P A P T Q P A P R-COOH
y14 y13 y12 y11 y10 y9 y8 y7 y6 y5 y4 y2 y1
Int
100
75
50
25
0
iY
y1
y2
y7 2+
y4
y14 3+
y5
y6
y7
y8
y9
y10
y11
y12
y13
m/z
0
250
500
750
1,000
1,250
1,500
1,750
2,000
2,250
2,500
2,750

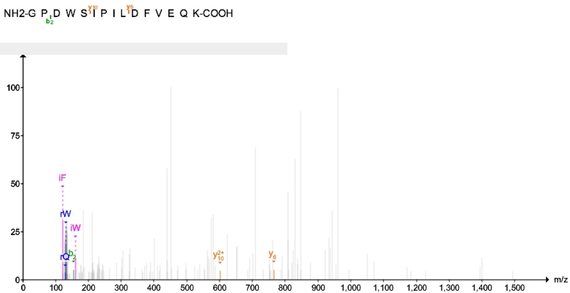

NH2-G P D W S I P I L D F V E Q K-COOH
b2
y10
y6
iF
rW
iW
rQ
b2
y10 2+
y6
m/z
0
25
50
75
100
0
100
200
300
400
500
600
700
800
900
1,000
1,100
1,200
1,300
1,400
1,500

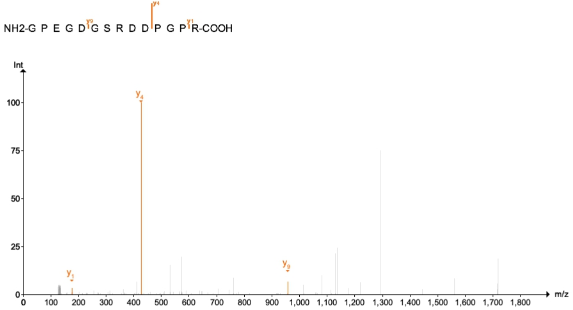
NH2-G P E G D G S R D D P G P R-COOH
y9
y4
y1
Int
y4
y1
y9
m/z
0
25
50
75
100
0
100
200
300
400
500
600
700
800
900
1,000
1,100
1,200
1,300
1,400
1,500
1,600
1,700
1,800

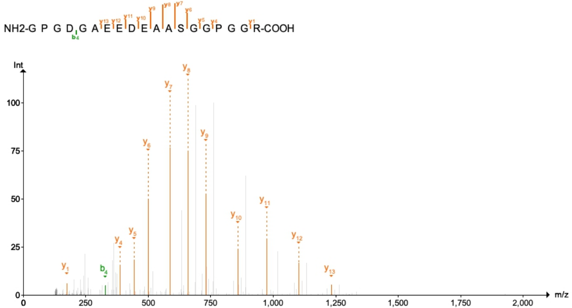

NH2-G P G D G A E E D E A A S G G P G G R-COOH
Int
m/z
y1
b4
y4
y5
y6
y7
y8
y9
y10
y11
y12
y13
0
25
50
75
100
250
500
750
1,000
1,250
1,500
1,750
2,000

NH2-G P L A T G G I K-COOH

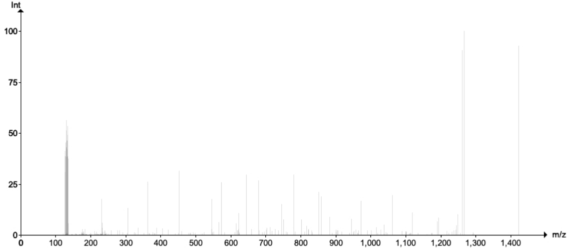

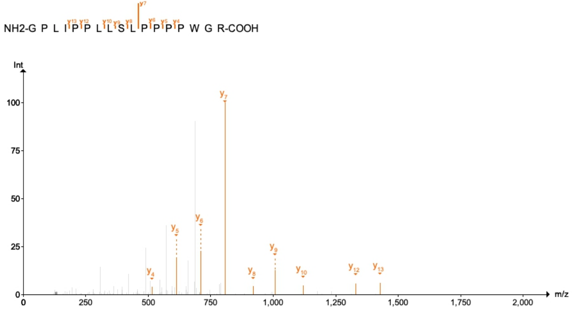
NH2-G P L I P P L L S L P P P P W G R-COOH
y13 y12 y10 y9 y8 y7 y6 y5 y4
Int
100
75
50
25
0
y4 y5 y6 y7 y8 y9 y10 y12 y13
0 250 500 750 1,000 1,250 1,500 1,750 2,000
m/z

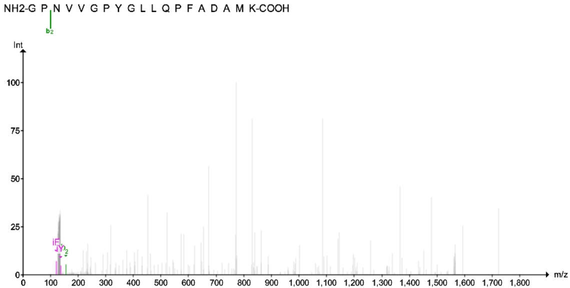

NH2-G P N V V G P Y G L L Q P F A D A M K-COOH
b2
Int
100
75
50
25
0
iF
Y2
0
100
200
300
400
500
600
700
800
900
1,000
1,100
1,200
1,300
1,400
1,500
1,600
1,700
1,800
m/z

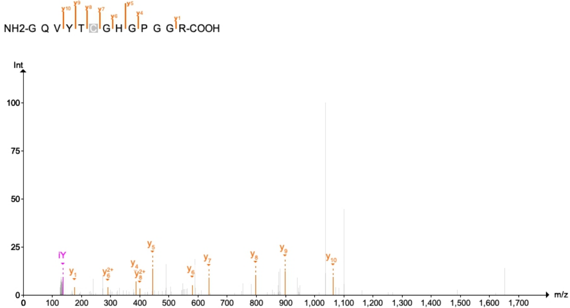

NH2-G Q V Y T C G H G P G G R-COOH
y10
y9
y8
y7
y6
y5
y4
y1
Int
100
75
50
25
0
iY
y1
y6 2+
y4
y8 2+
y5
y6
y7
y8
y9
y10
m/z
0
100
200
300
400
500
600
700
800
900
1,000
1,100
1,200
1,300
1,400
1,500
1,600
1,700

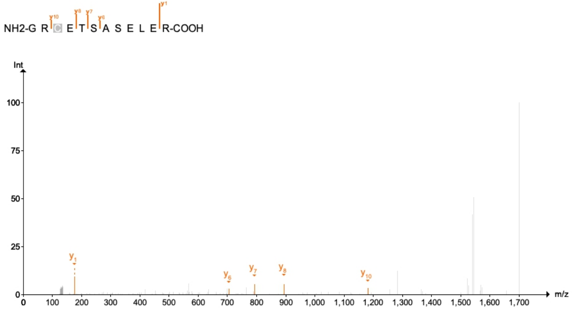
NH2-G R C E T S A S E L E R-COOH
y10
y8
y7
y6
y1
Int
100
75
50
25
0
m/z
0
100
200
300
400
500
600
700
800
900
1,000
1,100
1,200
1,300
1,400
1,500
1,600
1,700
y1
y6
y7
y8
y10

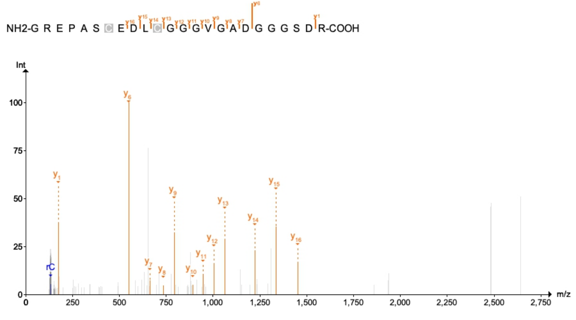

NH2-G R E P A S C E D L C G G V G A D G G G S D R-COOH
Int
m/z
y1
y6
y7
y8
y9
y10
y11
y12
y13
y14
y15
y16
rC

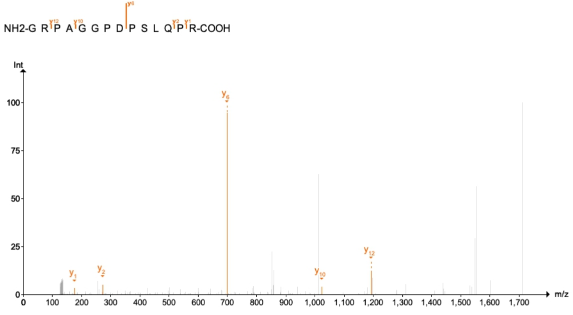

NH2-G R P A G G P D P S L Q P R-COOH
y12
y10
y6
y2
y1
Int
100
75
50
25
0
0
100
200
300
400
500
600
700
800
900
1,000
1,100
1,200
1,300
1,400
1,500
1,600
1,700
m/z
y6
y1
y2
y10
y12

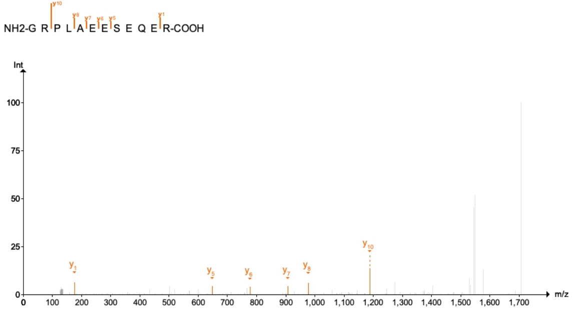

NH2-G R P L A E E S E Q E R-COOH
y10
y8
y7
y6
y5
y1
Int
m/z
y1
y5
y6
y7
y8
y10

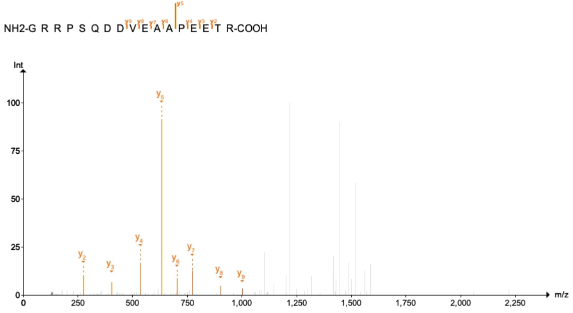

NH2-G R R P S Q D D V E A A P E E T R-COOH
y9 y8 y7 y6 y5 y4 y3 y2
Int
m/z
y2
y3
y4
y5
y6
y7
y8
y9
0
25
50
75
100
250
500
750
1,000
1,250
1,500
1,750
2,000
2,250

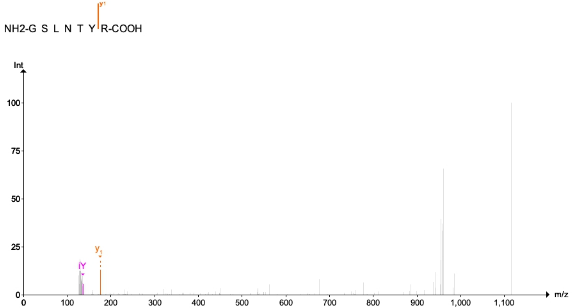

y1
NH2-G S L N T Y R-COOH
Int
100
75
50
25
0
IY
y1
0
100
200
300
400
500
600
700
800
900
1,000
1,100
m/z

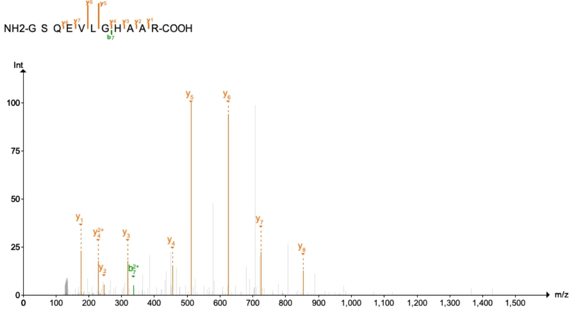

NH2-G S Q E V L G H A A R-COOH
Int
m/z
y1
y2
y3
y4
y5
y6
y7
y8
b7
0
25
50
75
100
0
100
200
300
400
500
600
700
800
900
1,000
1,100
1,200
1,300
1,400
1,500

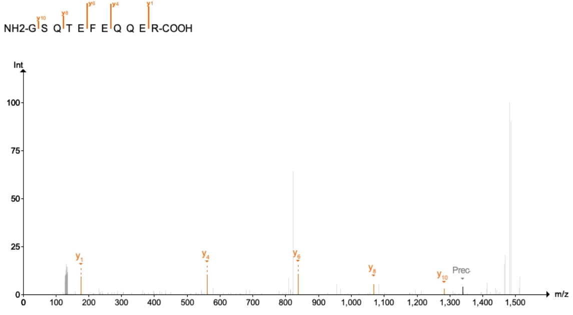

NH2-G S Q T E F E Q Q E R-COOH
y10
y8
y6
y4
y1
Int
100
75
50
25
0
m/z
0
100
200
300
400
500
600
700
800
900
1,000
1,100
1,200
1,300
1,400
1,500
y1
y4
y6
y8
y10
Prec

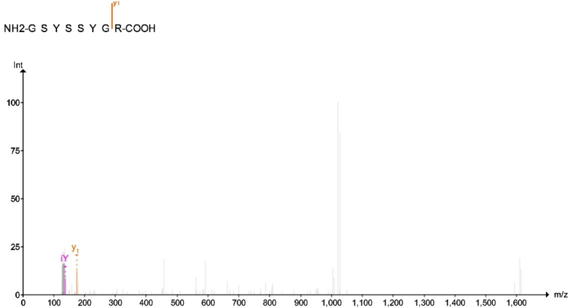

NH2-G S Y S S Y G R-COOH
y1
Int
100
75
50
25
0
iY
y1
0
100
200
300
400
500
600
700
800
900
1,000
1,100
1,200
1,300
1,400
1,500
1,600
m/z

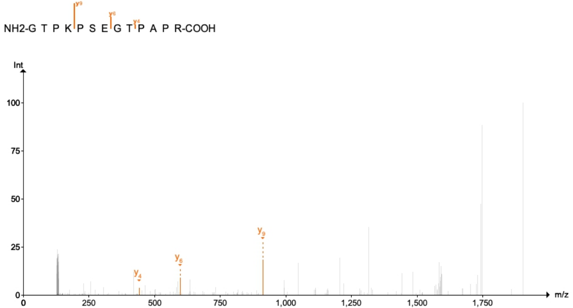
NH2-G T P K P S E G T P A P R-COOH
y9
y6
y4
Int
100
75
50
25
0
0
250
500
750
1,000
1,250
1,500
1,750
m/z
y9
y6
y4

NH2-G T S S F G K-COOH

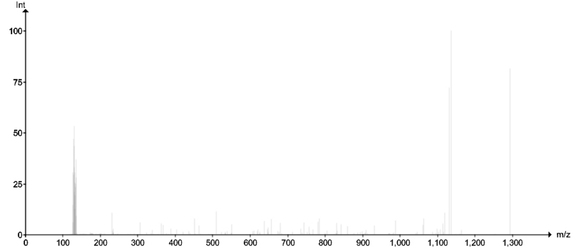

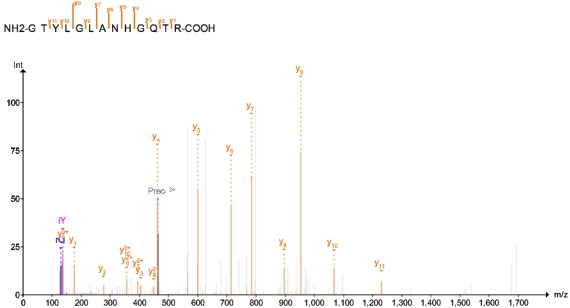

NH2-G T Y L G L A N H G Q T R-COOH
Int
m/z
Prec 3+
iY
y1
y2
y3
y4
y5
y6
y7
y8
y9
y10
y11
0
25
50
75
100
0
100
200
300
400
500
600
700
800
900
1,000
1,100
1,200
1,300
1,400
1,500
1,600
1,700

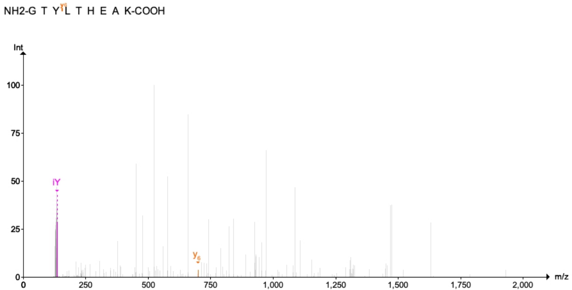
NH2-G T Y L T H E A K-COOH
y6
Int
100
75
50
25
0
iY
y6
0
250
500
750
1,000
1,250
1,500
1,750
2,000
m/z

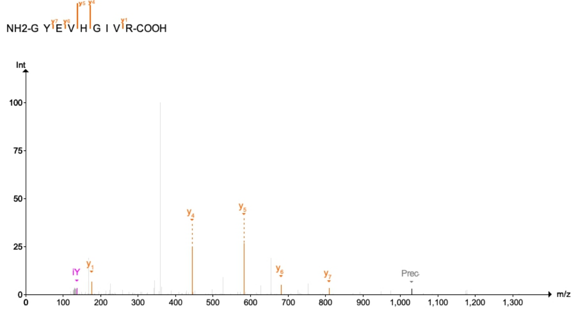

NH2-G Y E V H G I V R-COOH
y7
y6
y5
y4
y1
Int
100
75
50
25
0
y4
y5
iY
y1
y6
y7
Prec
0
100
200
300
400
500
600
700
800
900
1,000
1,100
1,200
1,300
m/z

NH2-G Y L L Y G P P G C G K-COOH

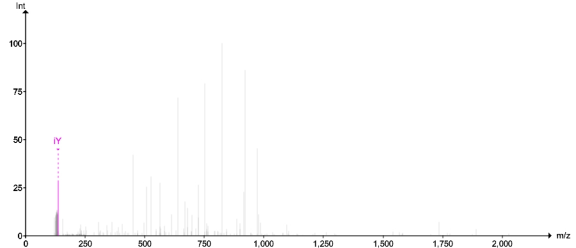

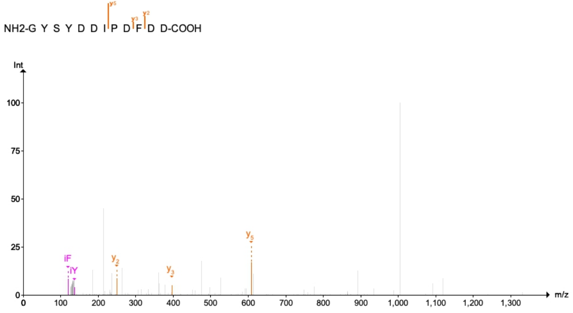

NH2-G Y S Y D D I P D F D D-COOH
y5
y3
y2
Int
100
75
50
25
0
iF
iY
y2
y3
y5
0
100
200
300
400
500
600
700
800
900
1,000
1,100
1,200
1,300
m/z

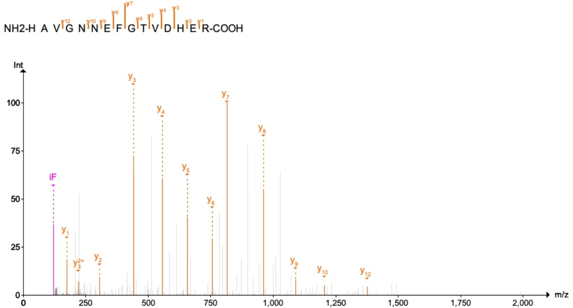

NH2-H A V G N N E F G T V D H E R-COOH
y12 y10 y9 y8 y7 y6 y5 y4 y3 y2 y1
Int
100
75
50
25
0
iF
y1
y3 2+
y2
y3
y4
y5
y6
y7
y8
y9
y10
y12
0
250
500
750
1,000
1,250
1,500
1,750
2,000
m/z

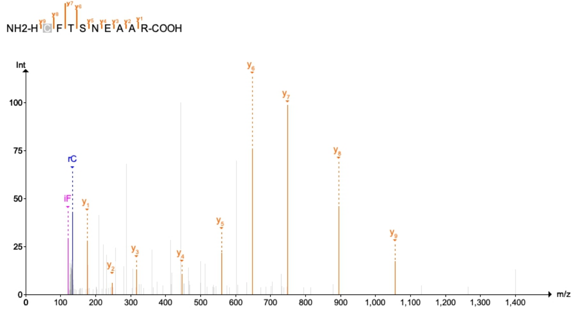

NH2-H C F T S N E A A R-COOH
y9 y8 y7 y6 y5 y4 y3 y2 y1
Int
m/z
rC
iF
y1
y2
y3
y4
y5
y6
y7
y8
y9
0
25
50
75
100
0
100
200
300
400
500
600
700
800
900
1,000
1,100
1,200
1,300
1,400

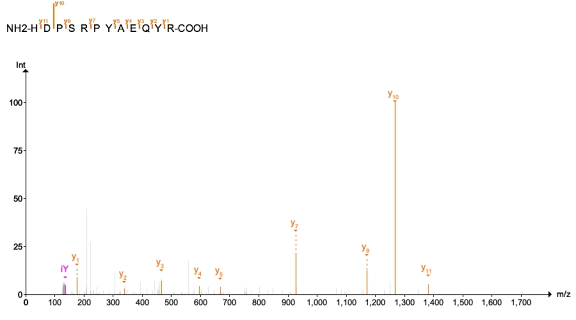

NH2-H D P S R P Y A E Q Y R-COOH
y10
y11
y9
y7
y5
y4
y3
y2
y1
Int
100
75
50
25
0
y10
y7
y9
y11
iY
y1
y2
y3
y4
y5
0
100
200
300
400
500
600
700
800
900
1,000
1,100
1,200
1,300
1,400
1,500
1,600
1,700
m/z

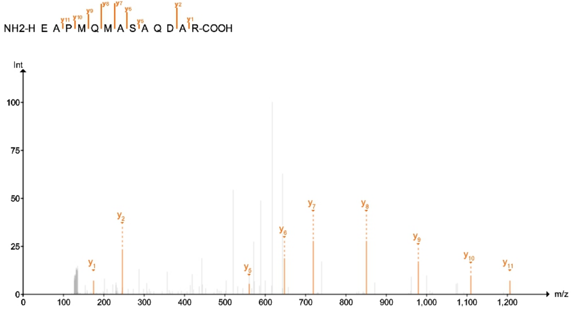
NH2-H E A P M Q M A S A Q D A R-COOH
y11 y10 y9 y8 y7 y6 y5 y2 y1
Int
100
75
50
25
0
y1 y2 y5 y6 y7 y8 y9 y10 y11
0 100 200 300 400 500 600 700 800 900 1,000 1,100 1,200
m/z

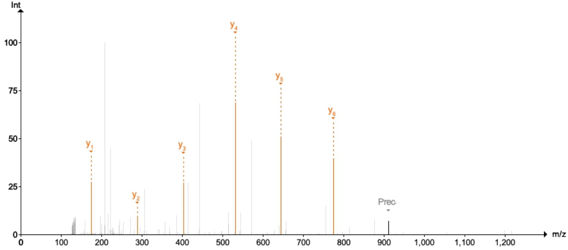

Int
100
75
50
25
0
y1
y2
y3
y4
y5
y6
Prec
0
100
200
300
400
500
600
700
800
900
1,000
1,100
1,200
m/z

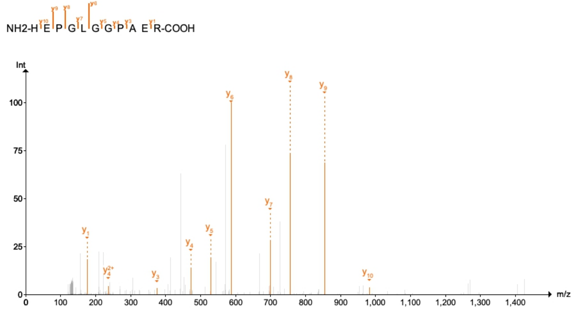

NH2-H E P G L G G P A E R-COOH
y10 y9 y8 y7 y6 y5 y4 y3 y1
Int
m/z
y1
y2+4
y3
y4
y5
y6
y7
y8
y9
y10

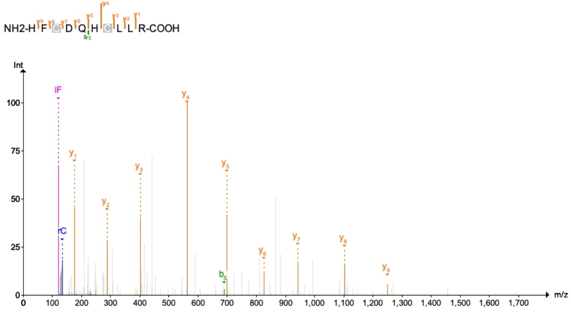

NH2-H F C D Q H C L L R-COOH
y9 y8 y7 y6 y5 y4 y3 y2 y1
b5
Int
iF
rC
y1 y2 y3 y4 y5 y6 y7 y8 y9
b5
m/z
0
25
50
75
100
0 100 200 300 400 500 600 700 800 900 1,000 1,100 1,200 1,300 1,400 1,500 1,600 1,700

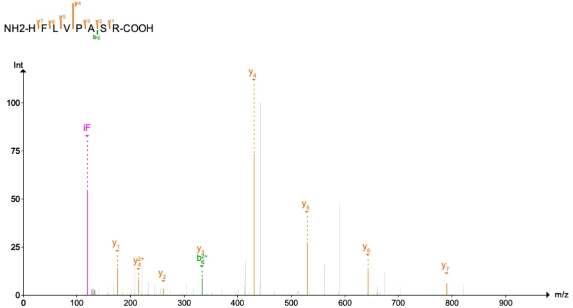
NH2-HFLVPASR-COOH
y7 y6 y5 y4 y3 y2 y1
b6
Int
m/z
iF
y4
y5
y6
y7
y1
y4 2+
y2
y3
b6 2+
0
25
50
75
100
0
100
200
300
400
500
600
700
800
900

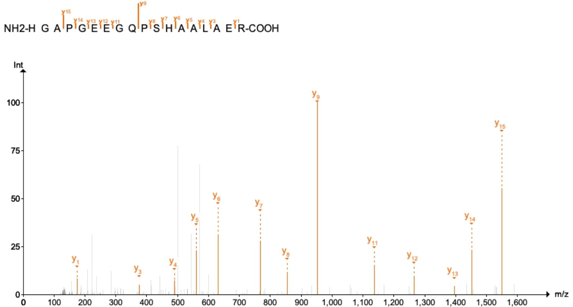

NH2-H G A P G E E G Q P S H A A L A E R-COOH
y15
y14
y13
y12
y11
y9
y8
y7
y6
y5
y4
y3
y1
Int
100
75
50
25
0
y1
y3
y4
y5
y6
y7
y8
y9
y11
y12
y13
y14
y15
0
100
200
300
400
500
600
700
800
900
1,000
1,100
1,200
1,300
1,400
1,500
1,600
m/z

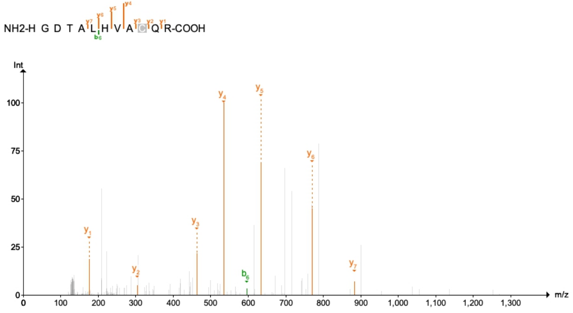

NH2-H G D T A L H V A C Q R-COOH
y7 y6 y5 y4 y3 y2 y1
b6
Int
100
75
50
25
0
y1 y2 y3 y4 y5 y6 y7 b6
0 100 200 300 400 500 600 700 800 900 1,000 1,100 1,200 1,300
m/z

NH2-H G I E Y Q S K-COOH

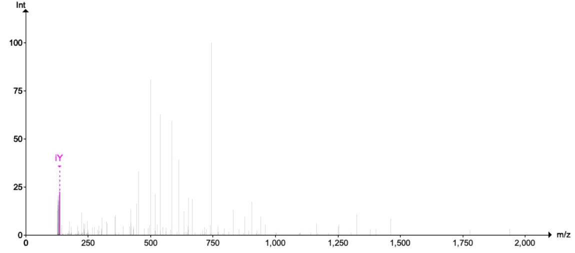

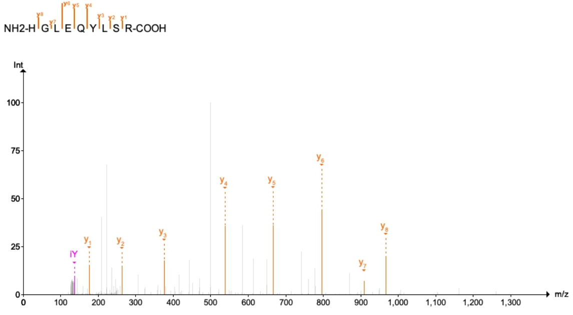
NH2-H G L E Q Y L S R-COOH
y8 y7 y6 y5 y4 y3 y2 y1
Int
m/z
IY
y1
y2
y3
y4
y5
y6
y7
y8

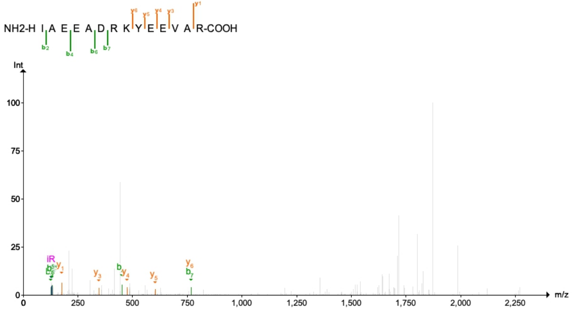

NH2-H I A E E A D R K Y E E V A R-COOH
y6 y5 y4 y3 y1
b2 b4 b6 b7
Int
100
75
50
25
0
iR
y1
y3
y4
y5
y6
b7
0 250 500 750 1,000 1,250 1,500 1,750 2,000 2,250
m/z

NH2-H I L G D P N L T K-COOH

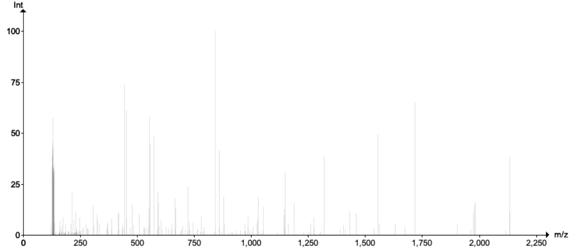

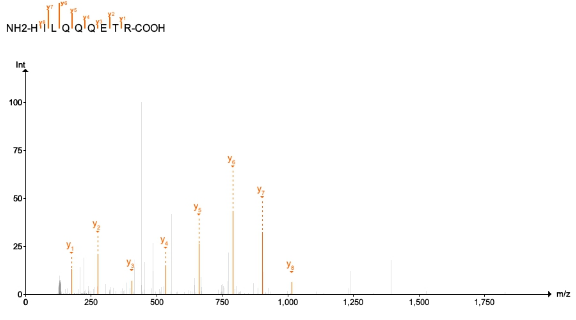

NH2-H I L Q Q Q E T R-COOH
y7 y8 y6 y5 y4 y3 y2 y1
Int
100
75
50
25
0
y1
y2
y3
y4
y5
y6
y7
y8
0
250
500
750
1,000
1,250
1,500
1,750
m/z

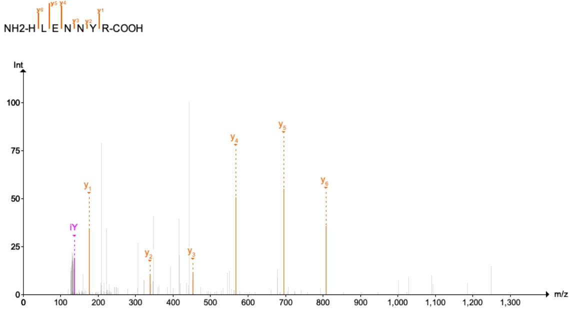

NH2-H L E N N Y R-COOH
y6
y5
y4
y3
y2
y1
Int
100
75
50
25
0
IY
y1
y2
y3
y4
y5
y6
0
100
200
300
400
500
600
700
800
900
1,000
1,100
1,200
1,300
m/z

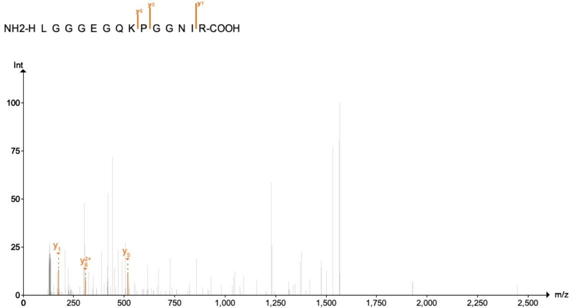
NH2-H L G G G E G Q K P G G N I R-COOH
y6
y5
y1
Int
100
75
50
25
0
y1
y6 2+
y5
0
250
500
750
1,000
1,250
1,500
1,750
2,000
2,250
2,500
m/z

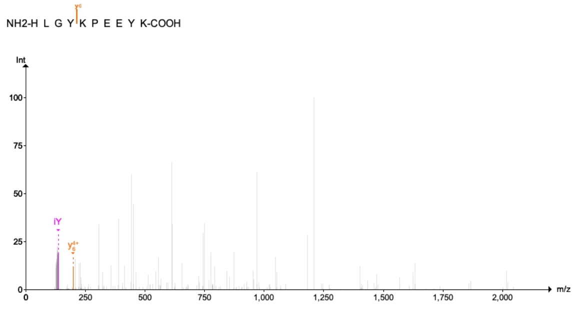

NH2-H L G Y K P E E Y K-COOH
y6
Int
100
75
50
25
0
iY
y6 4+
0
250
500
750
1,000
1,250
1,500
1,750
2,000
m/z

NH2-H L L E N A K-COOH

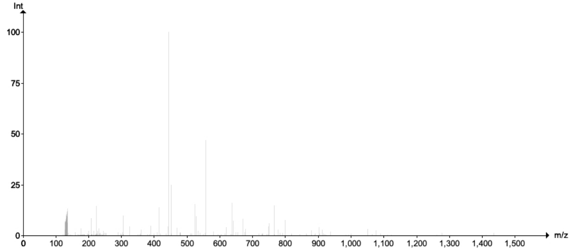

NH2-H L L Q P P P R P A Q P T S G K-COOH

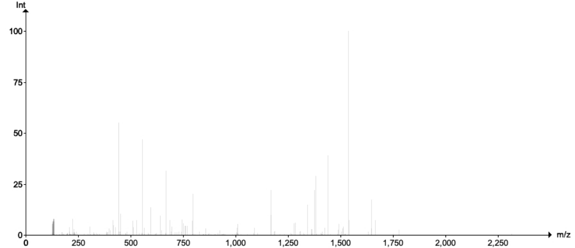

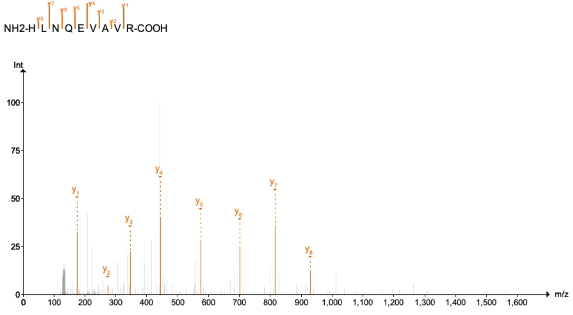

NH2-H L N Q E V A V R-COOH
y8 y7 y6 y5 y4 y3 y2 y1
Int
m/z
y1 y2 y3 y4 y5 y6 y7 y8
0 25 50 75 100
0 100 200 300 400 500 600 700 800 900 1,000 1,100 1,200 1,300 1,400 1,500 1,600

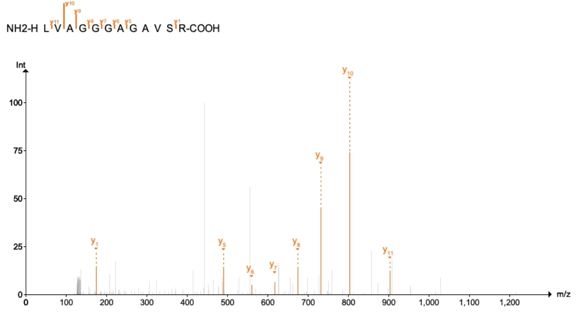
NH2-H L V A G G G A G A V S R-COOH
y11
y10
y9
y8
y7
y6
y5
y1
Int
m/z
y1
y5
y6
y7
y8
y9
y10
y11
0
25
50
75
100
0
100
200
300
400
500
600
700
800
900
1,000
1,100
1,200

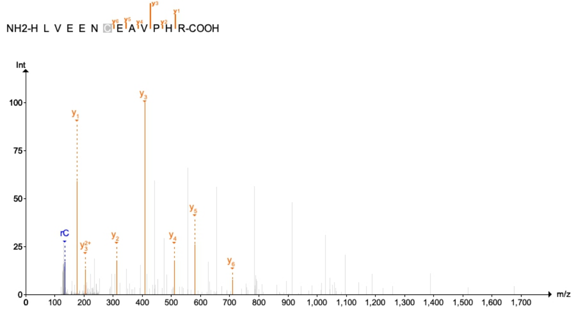
NH2-H L V E E N C E A V P H R-COOH
y6 y5 y4 y3 y2 y1
Int
m/z
rC
y1
y2
y3
y3 2+
y4
y5
y6
0
25
50
75
100
0 100 200 300 400 500 600 700 800 900 1,000 1,100 1,200 1,300 1,400 1,500 1,600 1,700

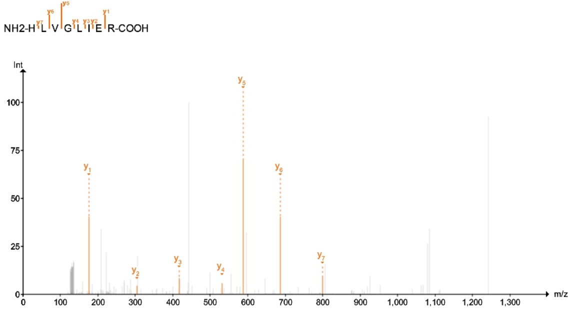
NH2-H L V G L I E R-COOH
y7
y6
y5
y4
y3
y2
y1
Int
m/z
100
75
50
25
0
0
100
200
300
400
500
600
700
800
900
1,000
1,100
1,200
1,300

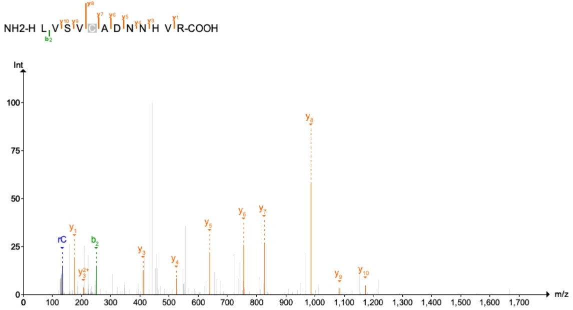

NH2-H L V S V C A D N N H V R-COOH
Int
m/z
rC
y1
b2
y3
y4
y5
y6
y7
y8
y9
y10

NH2-H N D V V P T M A Q G V L E Y K-COOH

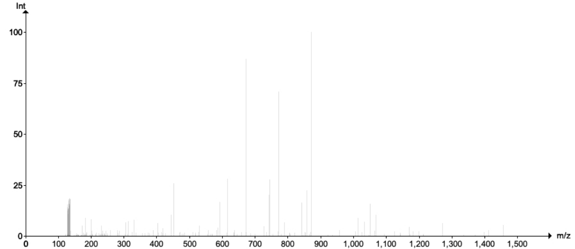

NH2-H N E E T G D N V G P L I I K-COOH

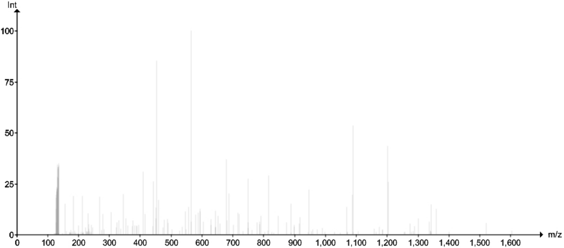

NH2-H N L Q P F H P K-COOH

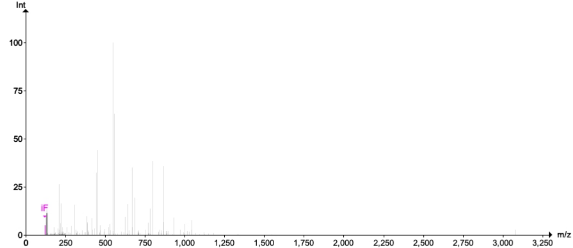

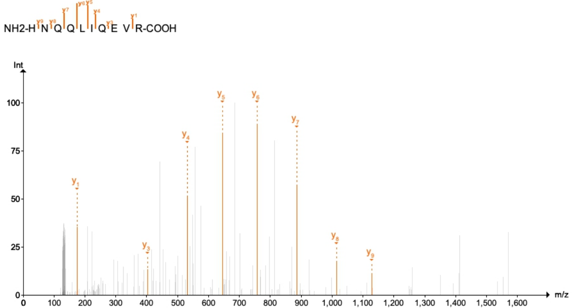
NH2-H N Q Q L I Q E V R-COOH
y9 y8 y7 y6 y5 y4 y3 y1
Int
100
75
50
25
0
y1 y3 y4 y5 y6 y7 y8 y9
0 100 200 300 400 500 600 700 800 900 1,000 1,100 1,200 1,300 1,400 1,500 1,600
m/z

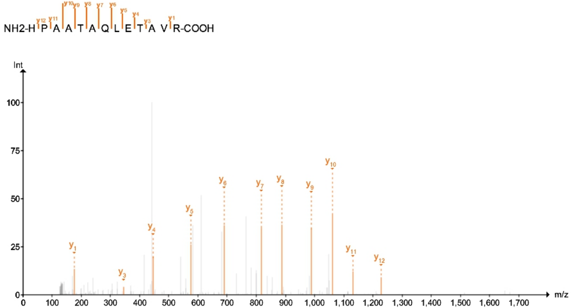

NH2-HPAATAQLETAVR-COOH
y12 y11 y10 y9 y8 y7 y6 y5 y4 y3 y1
Int
100
75
50
25
0
y1 y3 y4 y5 y6 y7 y8 y9 y10 y11 y12
0 100 200 300 400 500 600 700 800 900 1,000 1,100 1,200 1,300 1,400 1,500 1,600 1,700
m/z

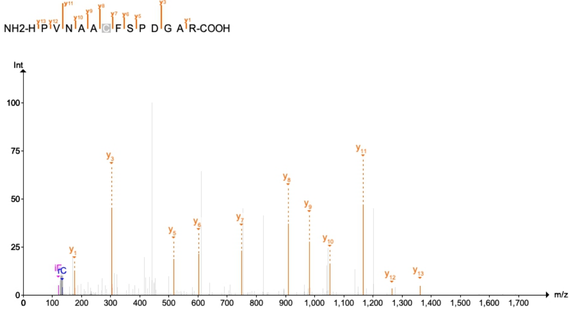
NH2-H P V N A A C F S P D G A R-COOH
y13 y12 y11 y10 y9 y8 y7 y6 y5 y3 y1
Int
100
75
50
25
0
y1 y3 y5 y6 y7 y8 y9 y10 y11 y12 y13
IF
rC
m/z
0 100 200 300 400 500 600 700 800 900 1,000 1,100 1,200 1,300 1,400 1,500 1,600 1,700

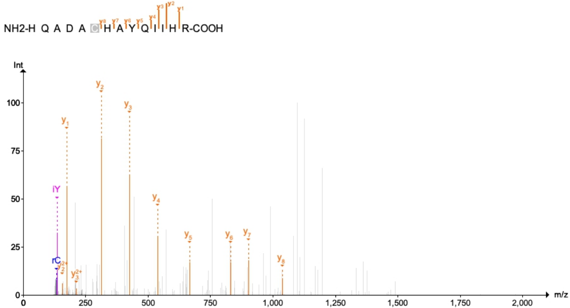
NH2-H Q A D A C H A Y Q I I H R-COOH
Int
100
75
50
25
0
y1
y2
y3
y4
y5
y6
y7
y8
iY
rC
m/z
0
250
500
750
1,000
1,250
1,500
1,750
2,000

NH2-H Q F G N E I K-COOH

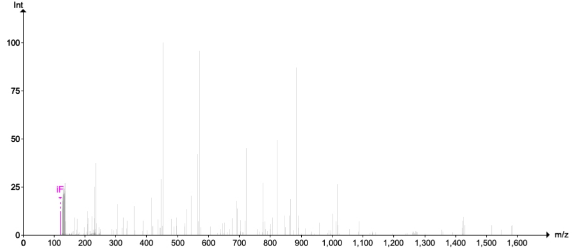

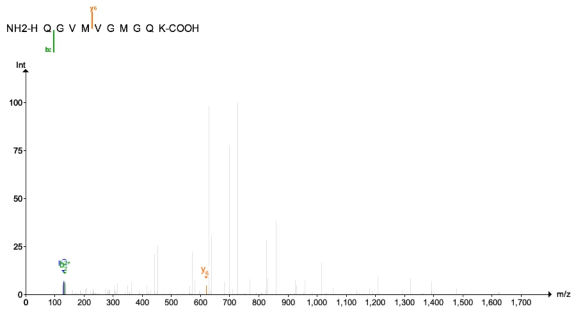
NH2-H Q G V M V G M G Q K-COOH
y6
b2
Int
100
75
50
25
0
y6
b2 2+
0
100
200
300
400
500
600
700
800
900
1,000
1,100
1,200
1,300
1,400
1,500
1,600
1,700
m/z

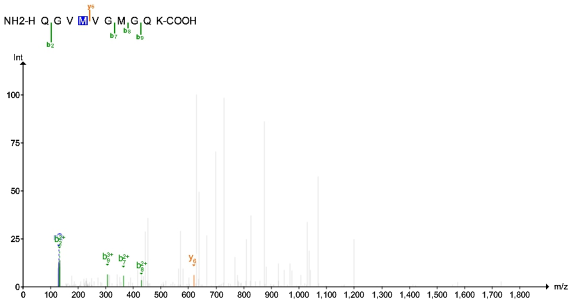

NH2-H Q G V M V G M G Q K-COOH
y6
b2
b7
b8
b9
Int
100
75
50
25
0
b2 2+
b9 3+
b7 2+
b8 2+
y6
0
100
200
300
400
500
600
700
800
900
1,000
1,100
1,200
1,300
1,400
1,500
1,600
1,700
1,800
m/z

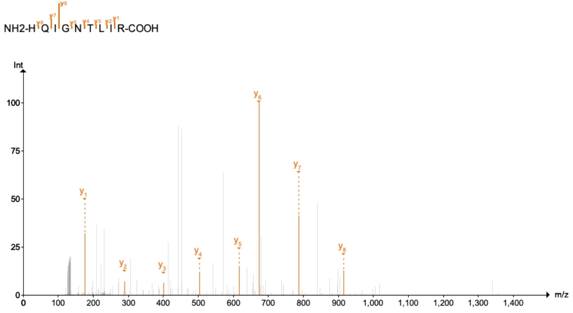
NH2-H Q I G N T L I R-COOH
y8
y7
y6
y5
y4
y3
y2
y1
Int
100
75
50
25
0
m/z
0
100
200
300
400
500
600
700
800
900
1,000
1,100
1,200
1,300
1,400

NH2-H Q Q Q L L A S P G S S T V D N K-COOH

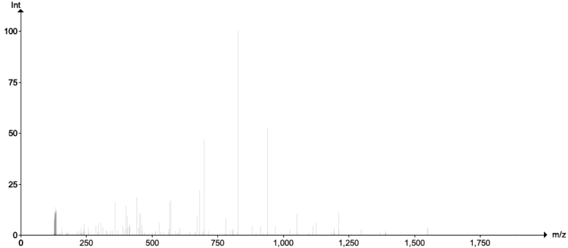

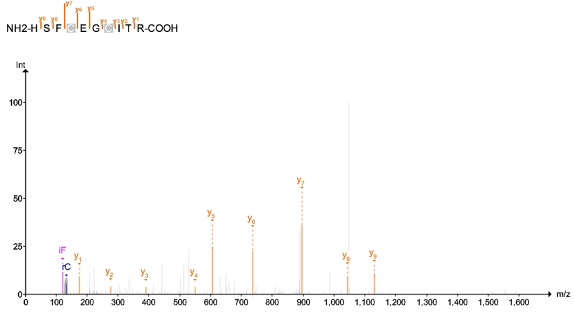
NH2-H S F C E G C I T R-COOH
y9 y8 y7 y6 y5 y4 y3 y2 y1
Int
m/z
iF
rC
y1
y2
y3
y4
y5
y6
y7
y8
y9
0
25
50
75
100
0 100 200 300 400 500 600 700 800 900 1,000 1,100 1,200 1,300 1,400 1,500 1,600

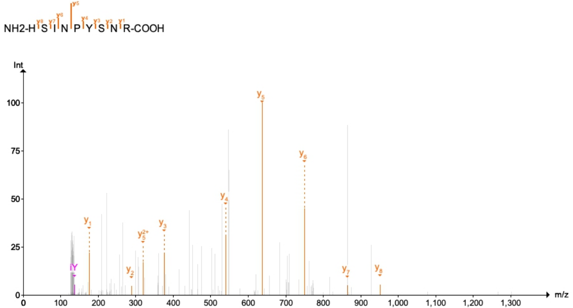
NH2-HSINPYSNR-COOH
Int
100
75
50
25
0
y1
y2
y5 2+
y3
y4
y5
y6
y7
y8
IY
0
100
200
300
400
500
600
700
800
900
1,000
1,100
1,200
1,300
m/z

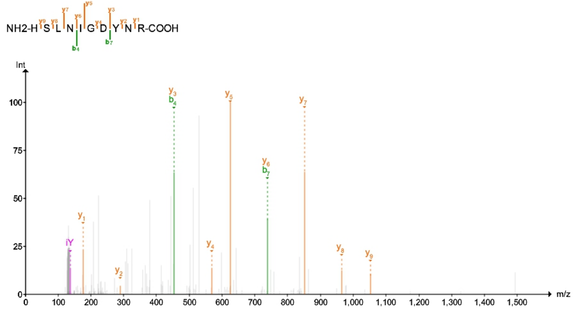

NH2-H S L N I G D Y N R-COOH
y9 y8 y7 y6 y5 y4 y3 y2 y1
b4 b7
Int
m/z
y3
b4
y5
y7
y6
b7
y1
iY
y2
y4
y8
y9

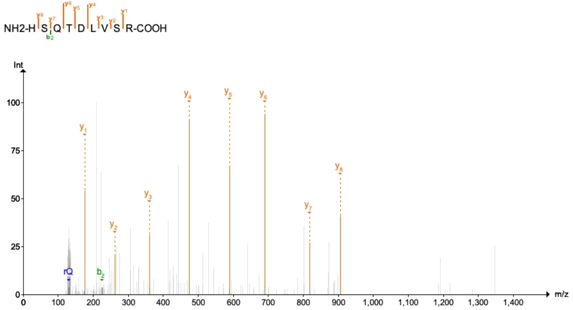

NH2-H S Q T D L V S R-COOH
y8
y7
y6
y5
y4
y3
y2
y1
b2
Int
100
75
50
25
0
y1
y2
y3
y4
y5
y6
y7
y8
rQ
b2
0
100
200
300
400
500
600
700
800
900
1,000
1,100
1,200
1,300
1,400
m/z

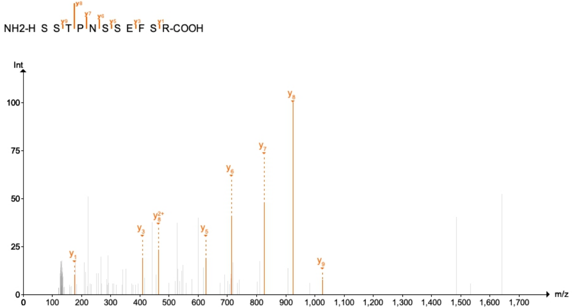
NH2-H S S T P N S S E F S R-COOH
y9
y8
y7
y6
y5
y3
y1
Int
100
75
50
25
0
y1
y3
y8 2+
y5
y6
y7
y8
y9
m/z
0
100
200
300
400
500
600
700
800
900
1,000
1,100
1,200
1,300
1,400
1,500
1,600
1,700

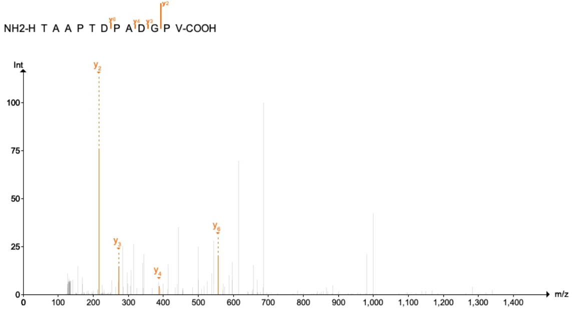

NH2-H T A A P T D P A D G P V-COOH
y6
y4
y3
y2
Int
100
75
50
25
0
y2
y3
y4
y6
0
100
200
300
400
500
600
700
800
900
1,000
1,100
1,200
1,300
1,400
m/z

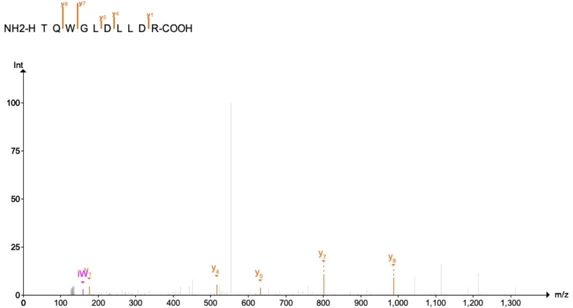

NH2-H T Q W G L D L L D R-COOH
y8
y7
y5
y4
y1
Int
100
75
50
25
0
m/z
0
100
200
300
400
500
600
700
800
900
1,000
1,100
1,200
1,300
IW
y1
y4
y5
y7
y8

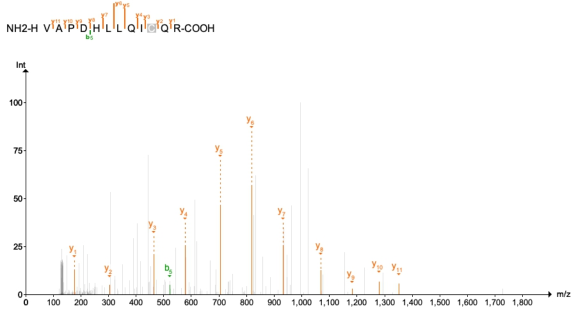

NH2-H V A P D H L L Q I C Q R-COOH
Int
m/z
y1
y2
y3
y4
y5
y6
y7
y8
y9
y10
y11
b5

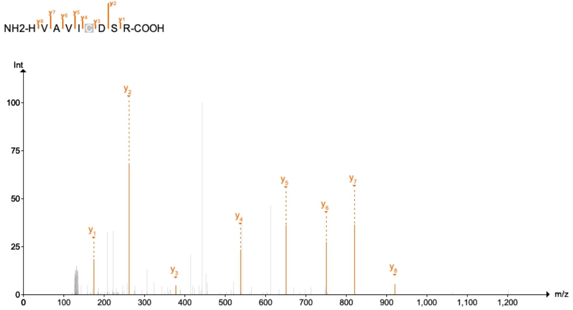
NH2-H V A V I C D S R-COOH
y8 y7 y6 y5 y4 y3 y2 y1
Int
m/z
y1
y2
y3
y4
y5
y6
y7
y8
0
25
50
75
100
0
100
200
300
400
500
600
700
800
900
1,000
1,100
1,200

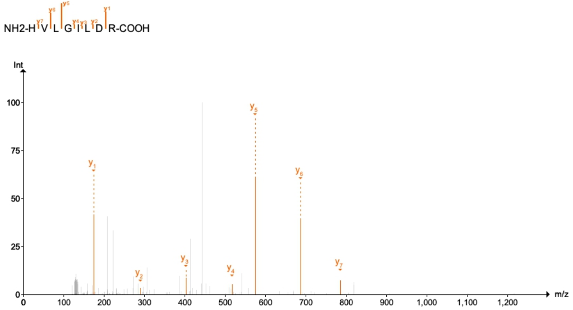
NH2-H V L G I L D R-COOH
Int
m/z
y1
y2
y3
y4
y5
y6
y7
0
25
50
75
100
0
100
200
300
400
500
600
700
800
900
1,000
1,100
1,200

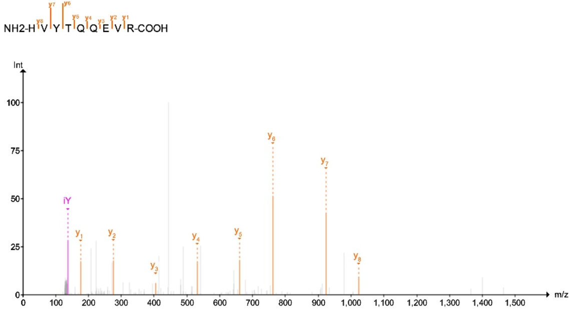
NH2-H V Y T Q Q E V R-COOH
y8 y7 y6 y5 y4 y3 y2 y1
Int
100
75
50
25
0
iY
y1
y2
y3
y4
y5
y6
y7
y8
0 100 200 300 400 500 600 700 800 900 1,000 1,100 1,200 1,300 1,400 1,500
m/z

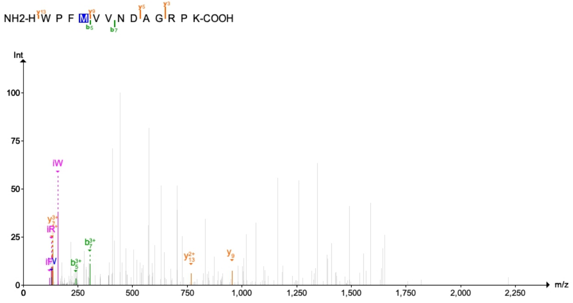

NH2-H W P F M V V N D A G R P K-COOH
Int
m/z
iW
iR
iF
V
b7
b5
y13
y9
y5
y3

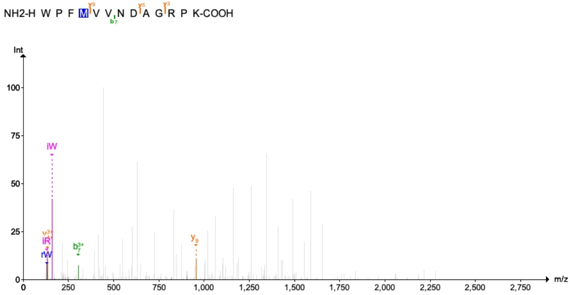

NH2-H W P F M V V N D A G R P K-COOH
y9
b7
y5
y3
Int
100
75
50
25
0
iW
y3+
iR
rW
b7 3+
y9
0
250
500
750
1,000
1,250
1,500
1,750
2,000
2,250
2,500
2,750
m/z

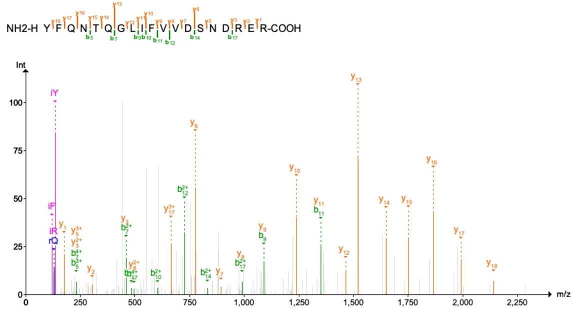

NH2-H Y F Q N T Q G L I F V V D S N D R E R-COOH
Int
m/z
iY
iF
iR
rQ
y6
y10
y11
b11
y13
y14
y15
y16
y17
y18
y12
y9
b9
y8
y7
y3
y2
y1

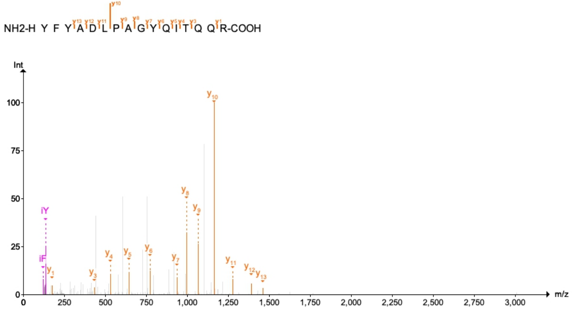

NH2-H Y F Y A D L P A G Y Q I T Q Q R-COOH
y13 y12 y11 y10 y9 y8 y7 y6 y5 y4 y3 y1
Int
m/z
iY
iF
y1
y3
y4
y5
y6
y7
y8
y9
y10
y11
y12
y13

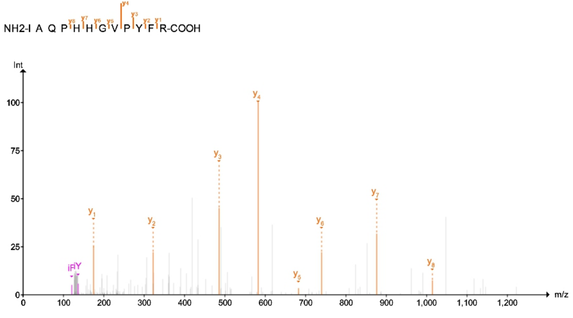

NH2-I A Q P H H G V P Y F R-COOH
y8
y7
y6
y5
y4
y3
y2
y1
Int
m/z
y1
y2
y3
y4
y5
y6
y7
y8
iF
iY

NH2-I A S H P A E Q Y V S K-COOH

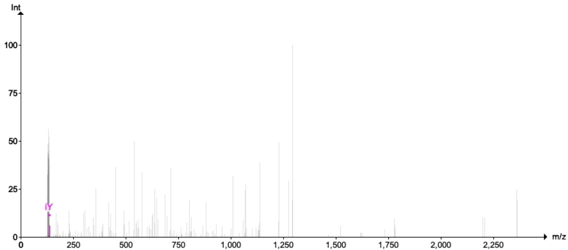

NH2-I C H T L T E K-COOH

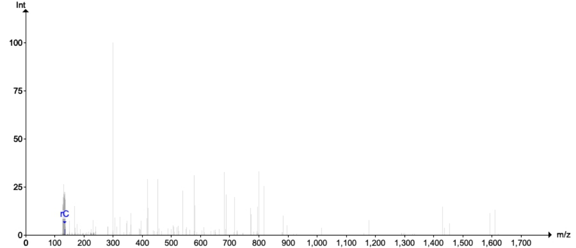

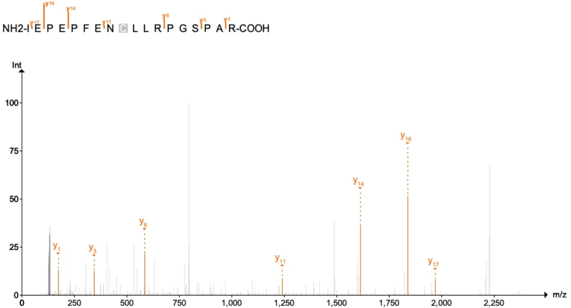
NH2-I E P E P F E N C L L R P G S P A R-COOH
y17
y16
y14
y11
y6
y3
y1
Int
100
75
50
25
0
250
500
750
1,000
1,250
1,500
1,750
2,000
2,250
m/z
y1
y3
y6
y11
y14
y16
y17

NH2-I G G G G V Q L G K-COOH

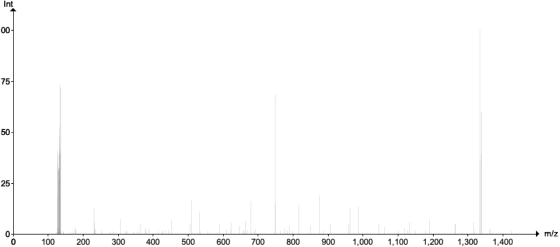

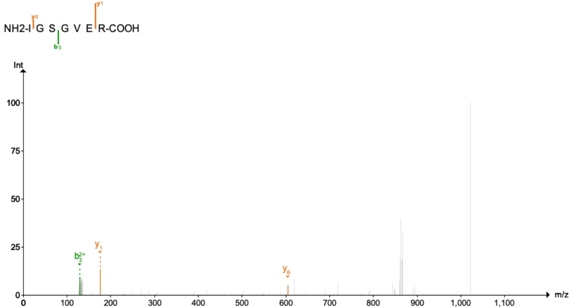

NH2-I G S G V E R-COOH
y6
y1
b3
Int
100
75
50
25
0
b_3^{2+}
y_1
y_6
0
100
200
300
400
500
600
700
800
900
1,000
1,100
m/z

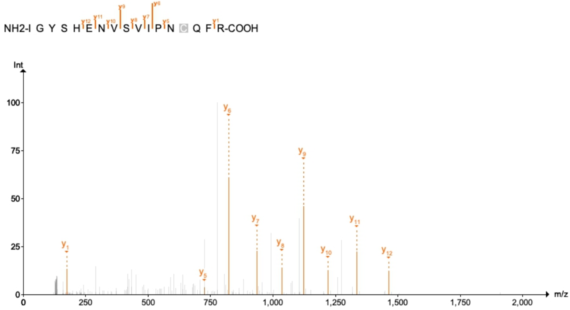

NH2-I G Y S H E N V S V I P N C Q F R-COOH
y12 y11 y10 y9 y8 y7 y6 y5 y1
Int
100
75
50
25
0
y1 y5 y6 y7 y8 y9 y10 y11 y12
0 250 500 750 1,000 1,250 1,500 1,750 2,000
m/z

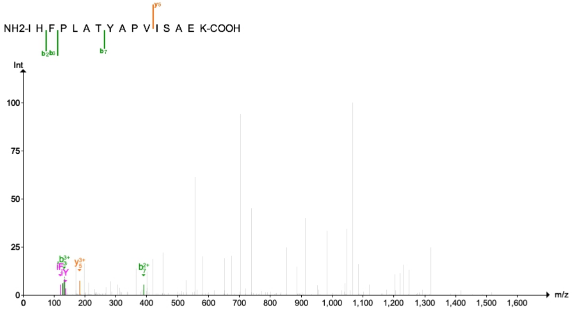

NH2-I H F P L A T Y A P V I S A E K-COOH
Int
m/z

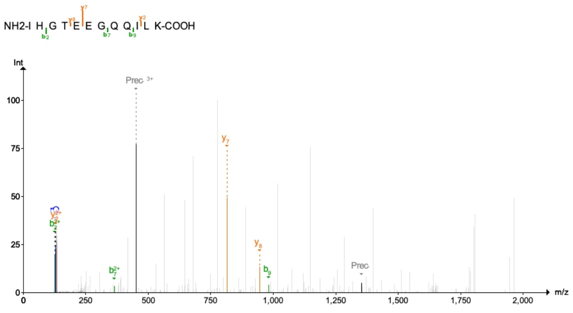

NH2-I H G T E E G Q Q I L K-COOH
Int
Prec 3+
y7
y8
b9
Prec
m/z

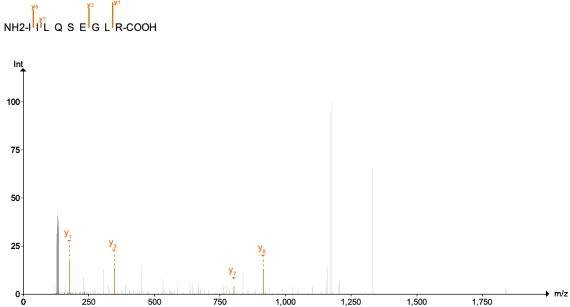

NH2-I I L Q S E G L R-COOH
y8
y7
y3
y1
Int
100
75
50
25
0
y1
y3
y7
y8
0
250
500
750
1,000
1,250
1,500
1,750
m/z

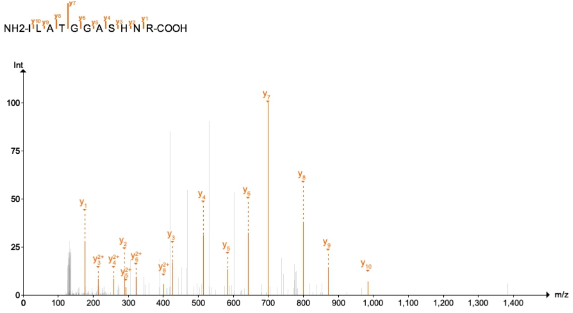
NH2-I L A T G G A S H N R-COOH
y10 y9 y8 y7 y6 y5 y4 y3 y2 y1
Int
100
75
50
25
0
y1
y2
y3
y4
y5
y6
y7
y8
y9
y10
y3 2+
y4 2+
y5 2+
y6 2+
y8 2+
m/z
0 100 200 300 400 500 600 700 800 900 1,000 1,100 1,200 1,300 1,400

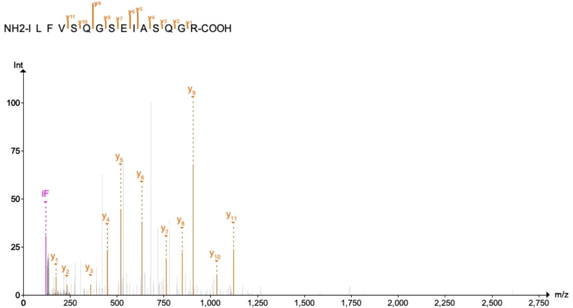

NH2-I L F V S Q G S E I A S Q G R-COOH
y11 y10 y9 y8 y7 y6 y5 y4 y3 y2 y1
Int
100
75
50
25
0
iF
y1
y2
y3
y4
y5
y6
y7
y8
y9
y10
y11
0
250
500
750
1,000
1,250
1,500
1,750
2,000
2,250
2,500
2,750
m/z

NH2-I L I D S I Y K-COOH

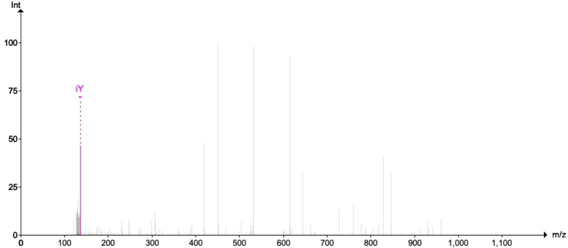

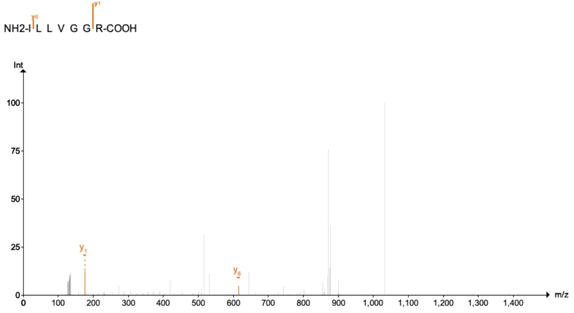

NH2-I L L V G G R-COOH
y6
y1
Int
100
75
50
25
0
m/z
0
100
200
300
400
500
600
700
800
900
1,000
1,100
1,200
1,300
1,400

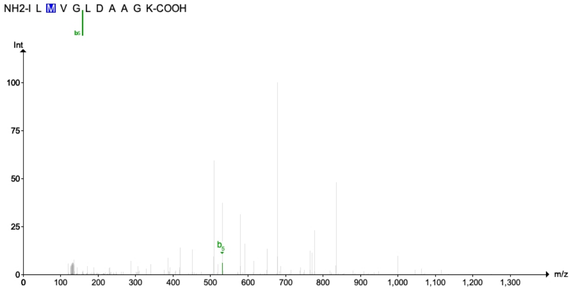

NH2-I L M V G L D A A G K-COOH
b5
Int
100
75
50
25
0
b5
0
100
200
300
400
500
600
700
800
900
1,000
1,100
1,200
1,300
m/z

NH2-I N S Q L V A Q Q V A Q Q Y A T P P P P K K-COOH

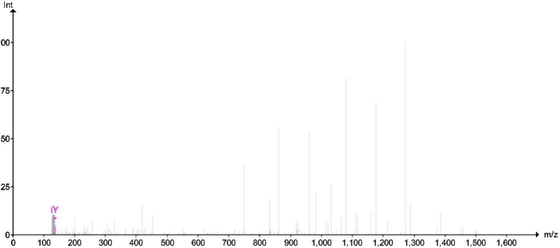

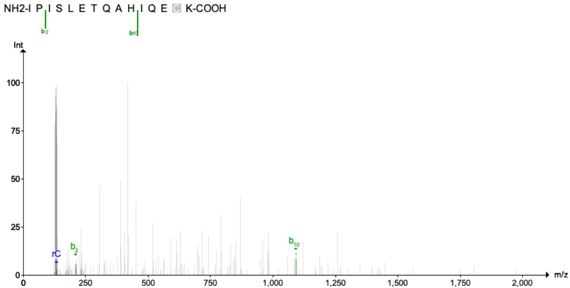

NH2-I P I S L E T Q A H I Q E C K-COOH
b2
b10
Int
100
75
50
25
0
rC
b2
b10
0
250
500
750
1,000
1,250
1,500
1,750
2,000
m/z

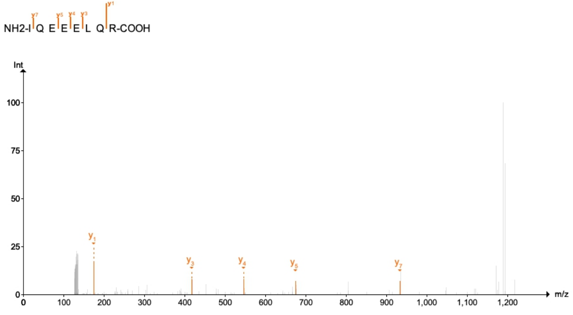

NH2-I Q E E E L Q R-COOH
y7
y5
y4
y3
y1
Int
100
75
50
25
0
y1
y3
y4
y5
y7
0
100
200
300
400
500
600
700
800
900
1,000
1,100
1,200
m/z

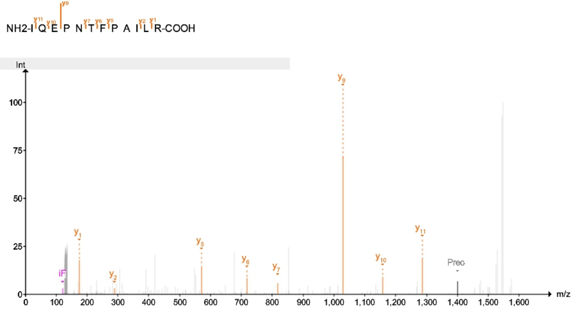

NH2-I Q E P N T F P A I L R-COOH
y11 y10 y9 y7 y6 y5 y2 y1
Int
100
75
50
25
0
y9
y1
y2
y5
y6
y7
y10
y11
iF
Prec
0 100 200 300 400 500 600 700 800 900 1,000 1,100 1,200 1,300 1,400 1,500 1,600
m/z

NH2-I S L P I E D Y F N K-COOH

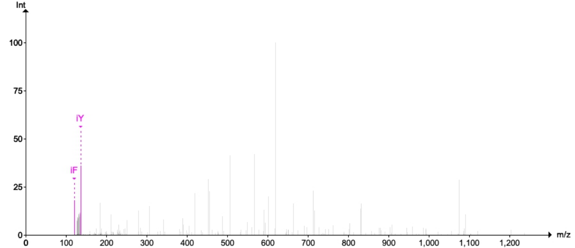

NH2-I V T H D I P I K-COOH

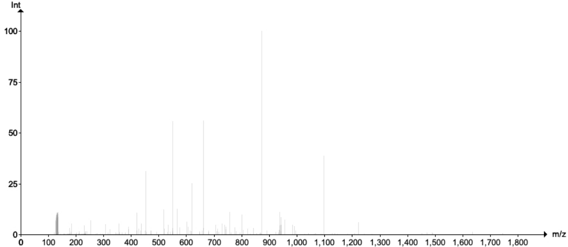

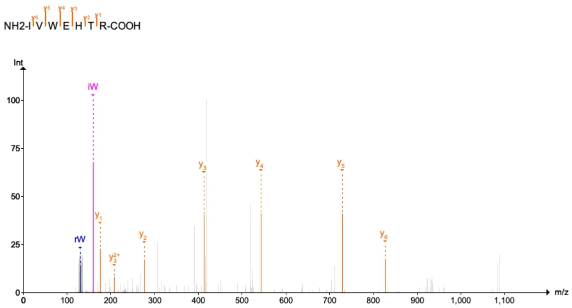

NH2-I V W E H T R-COOH
y6
y5
y4
y3
y2
y1
Int
100
75
50
25
0
iW
rW
y1
y2
y3
y3 2+
y4
y5
y6
0
100
200
300
400
500
600
700
800
900
1,000
1,100
m/z

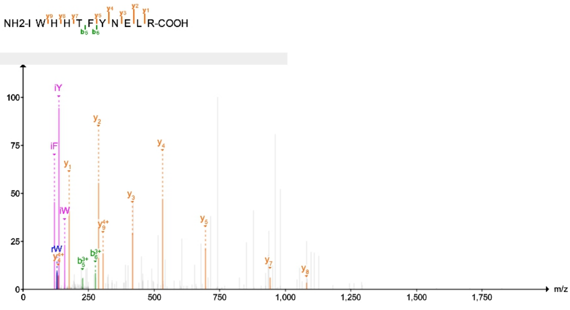
NH2-I W H H T F Y N E L R-COOH
iY
iF
iW
rW
y1
y2
y3
y4
y5
y7
y8
m/z

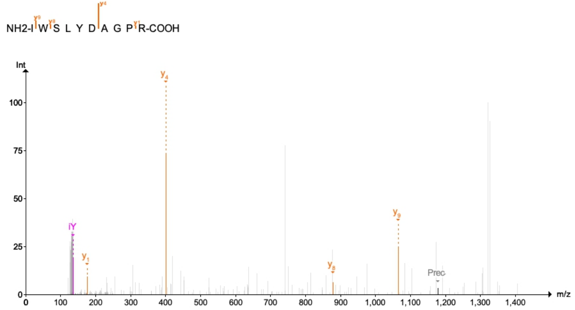

NH2-I W S L Y D A G P R-COOH
y9
y8
y4
y1
Int
y4
y9
y8
y1
iY
Prec
m/z
0
25
50
75
100
0
100
200
300
400
500
600
700
800
900
1,000
1,100
1,200
1,300
1,400

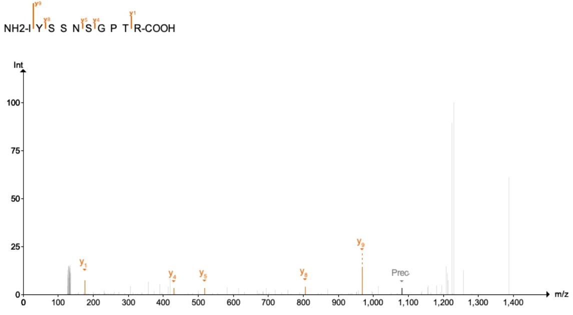
NH2-I Y S S N S G P T R-COOH
y9
y8
y5
y4
y1
Int
100
75
50
25
0
y1
y4
y5
y8
y9
Prec
0
100
200
300
400
500
600
700
800
900
1,000
1,100
1,200
1,300
1,400
m/z

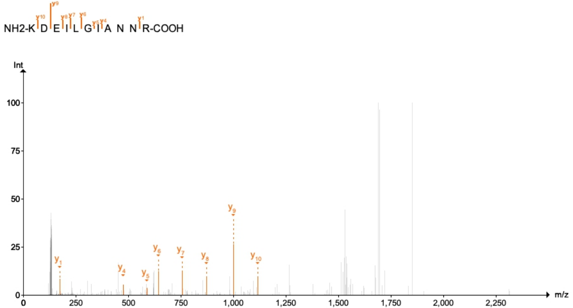

NH2-K D E I L G I A N N R-COOH
y10
y9
y8
y7
y6
y5
y4
y1
Int
100
75
50
25
0
y1
y4
y5
y6
y7
y8
y9
y10
0
250
500
750
1,000
1,250
1,500
1,750
2,000
2,250
m/z

NH2-K F E E I P I A H I K-COOH

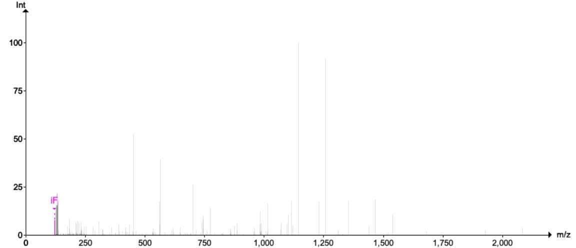

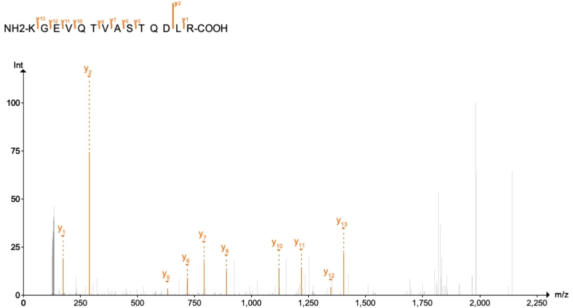

NH2-K G E V Q T V A S T Q D L R-COOH
Int
m/z
y1
y2
y5
y6
y7
y8
y10
y11
y12
y13
0
25
50
75
100
250
500
750
1,000
1,250
1,500
1,750
2,000
2,250

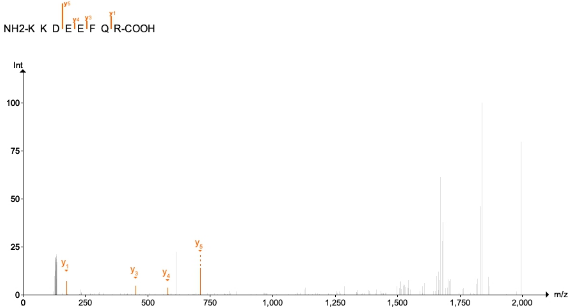

NH2-K K D E E F Q R-COOH
y5
y4
y3
y1
Int
100
75
50
25
0
250
500
750
1,000
1,250
1,500
1,750
2,000
m/z

NH2-K L E N C N Y A V E L G K-COOH

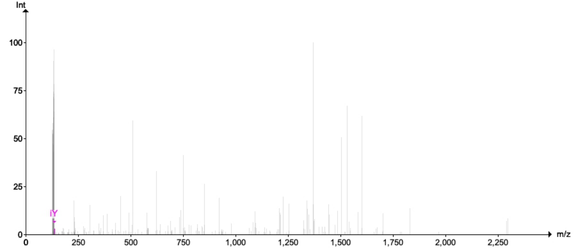

NH2-K L G T V I T P D T W K-COOH

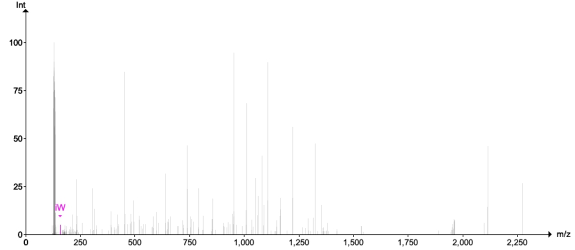

NH2-K P A P V P A E P F D N T T Y K-COOH

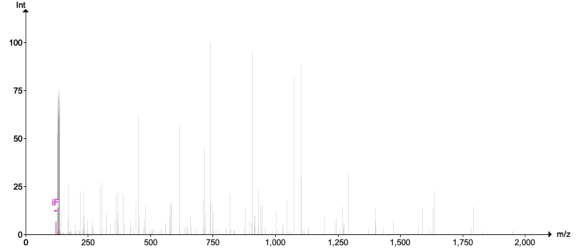

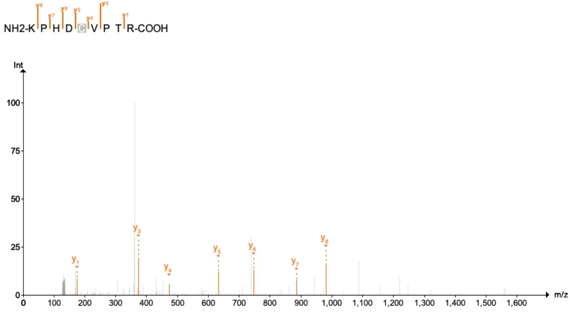

NH2-K P H D C V P T R-COOH
y8
y7
y6
y5
y4
y3
y1
Int
m/z
y1
y3
y4
y5
y6
y7
y8
0
25
50
75
100
0
100
200
300
400
500
600
700
800
900
1,000
1,100
1,200
1,300
1,400
1,500
1,600

NH2-K P L P G N A E E Q P S L Y E K-COOH

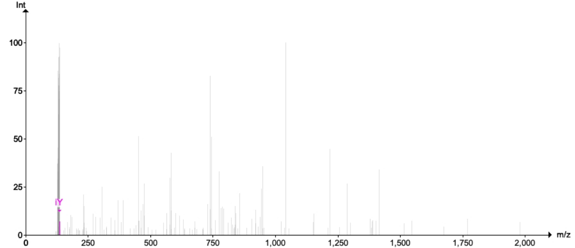

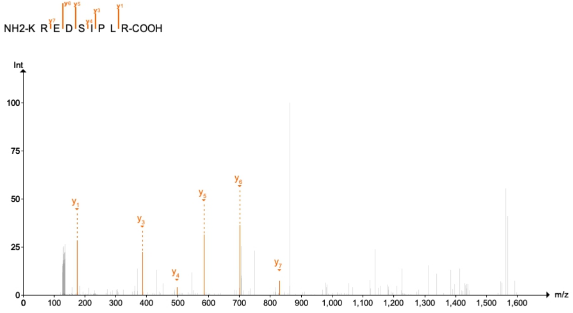
NH2-K R E D S I P L R-COOH
y8
y7
y6
y5
y4
y3
y1
Int
100
75
50
25
0
y1
y3
y4
y5
y6
y7
0
100
200
300
400
500
600
700
800
900
1,000
1,100
1,200
1,300
1,400
1,500
1,600
m/z

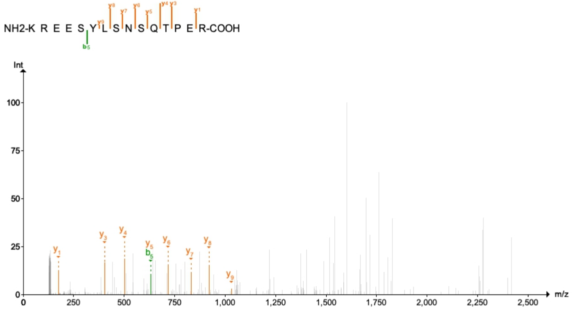

NH2-K R E E S Y L S N S Q T P E R-COOH
y8 y9 y7 y6 y5 y4 y3 y1
b5
Int
100
75
50
25
0
y1 y3 y4 y5 b5 y6 y7 y8 y9
0 250 500 750 1,000 1,250 1,500 1,750 2,000 2,250 2,500
m/z

NH2-K V E Q D L W N H A F K-COOH

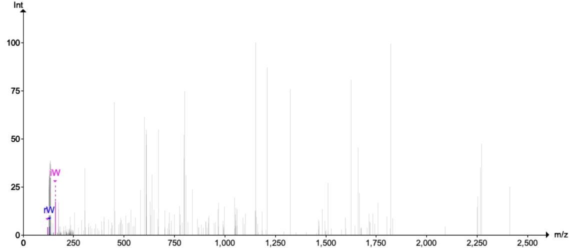

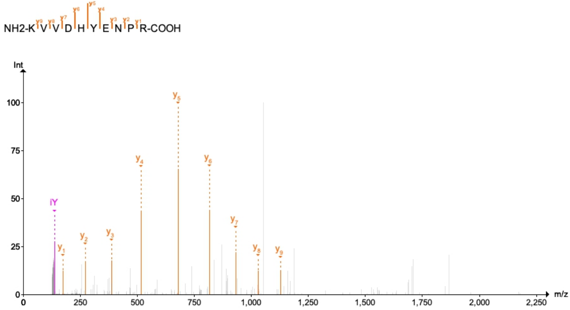

NH2-KVVDHYENPR-COOH
y9 y8 y7 y6 y5 y4 y3 y2 y1
Int
100
75
50
25
0
iY
y1
y2
y3
y4
y5
y6
y7
y8
y9
0
250
500
750
1,000
1,250
1,500
1,750
2,000
2,250
m/z

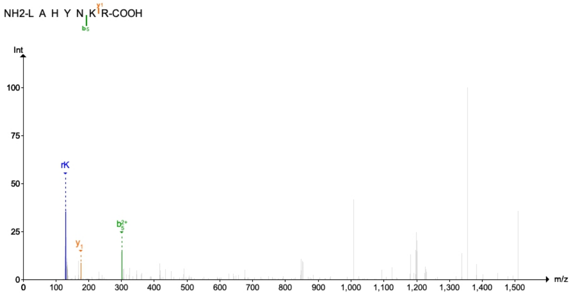
NH2-L A H Y N K R-COOH
y1
b5
Int
100
75
50
25
0
rK
y1
b5 2+
0
100
200
300
400
500
600
700
800
900
1,000
1,100
1,200
1,300
1,400
1,500
m/z

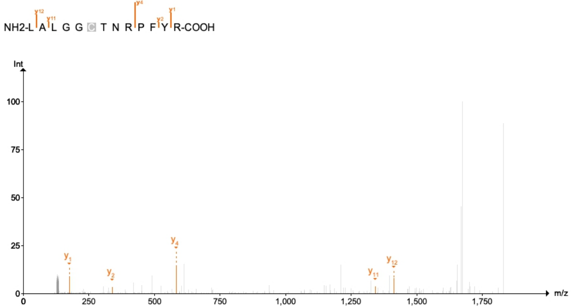
NH2-L A L G G C T N R P F Y R-COOH
y12
y11
y4
y2
y1
Int
100
75
50
25
0
250
500
750
1,000
1,250
1,500
1,750
m/z
y1
y2
y4
y11
y12

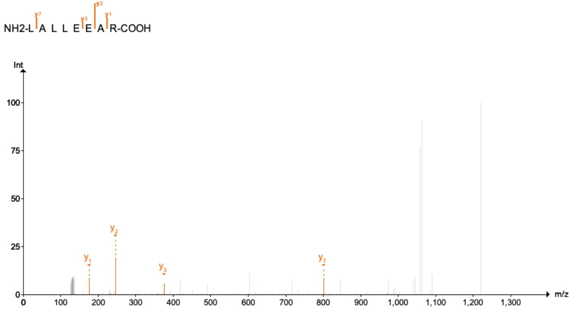
NH2-L A L L E E A R-COOH
y7
y3
y2
y1
Int
100
75
50
25
0
y1
y2
y3
y7
0
100
200
300
400
500
600
700
800
900
1,000
1,100
1,200
1,300
m/z

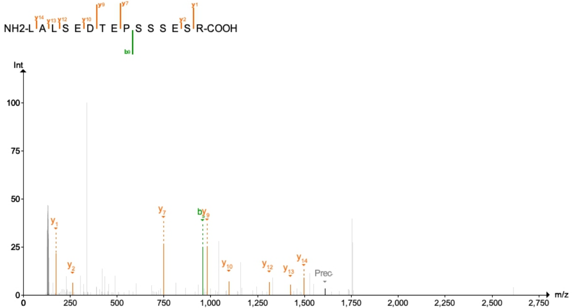

NH2-L A L S E D T E P S S S E S R-COOH
y14 y13 y12 y10 y9 y7 y2 y1
b9
Int
100
75
50
25
0
y1
y2
y7
b9
y9
y10
y12
y13
y14
Prec
m/z
0
250
500
750
1,000
1,250
1,500
1,750
2,000
2,250
2,500
2,750

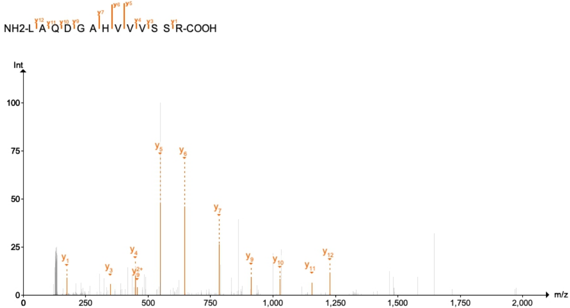

NH2-L A Q D G A H V V V S S R-COOH
y12
y11
y10
y9
y7
y6
y5
y4
y3
y1
Int
100
75
50
25
0
y5
y6
y7
y1
y3
y4
y9 2+
y9
y10
y11
y12
0
250
500
750
1,000
1,250
1,500
1,750
2,000
m/z

NH2-L A Q G P E L A E D D A N L L H K-COOH

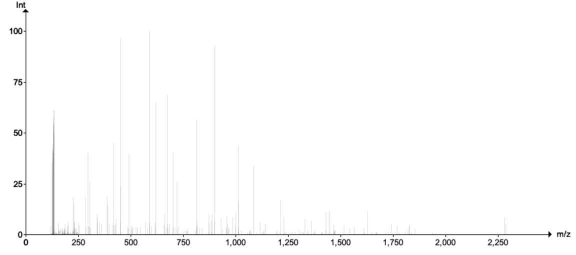

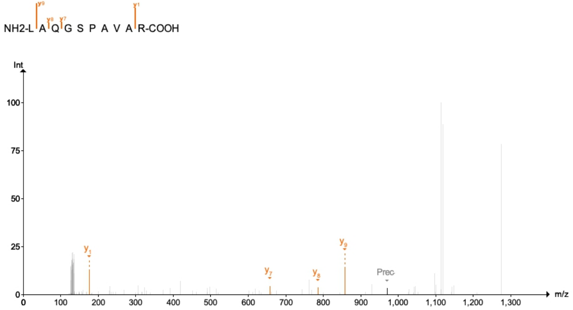

NH2-L A Q G S P A V A R-COOH
y9
y8
y7
y1
Int
100
75
50
25
0
m/z
100
200
300
400
500
600
700
800
900
1,000
1,100
1,200
1,300
y1
y7
y8
y9
Prec

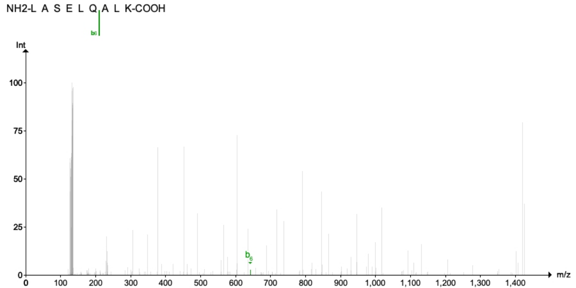
NH2-L A S E L Q A L K-COOH
b6
Int
100
75
50
25
0
0
100
200
300
400
500
600
700
800
900
1,000
1,100
1,200
1,300
1,400
m/z
b6

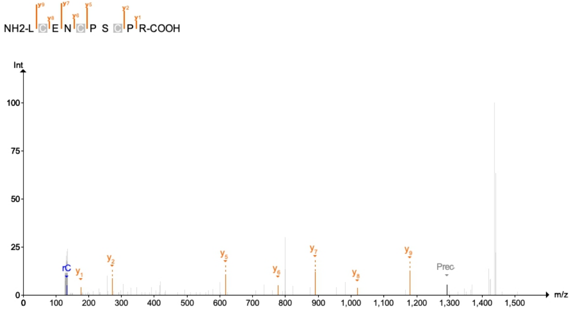

NH2-L C E N C P S C P R-COOH
y9
y8
y7
y6
y5
y2
y1
Int
100
75
50
25
0
rC
y1
y2
y5
y6
y7
y8
y9
Prec
0
100
200
300
400
500
600
700
800
900
1,000
1,100
1,200
1,300
1,400
1,500
m/z

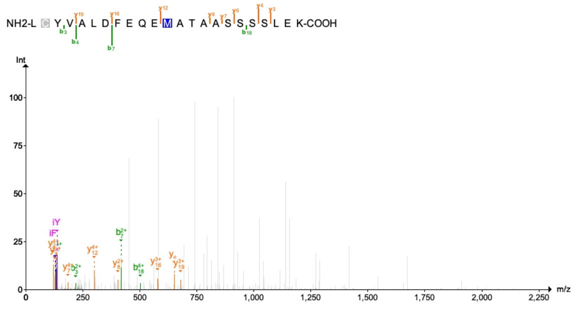

NH2-L C Y V A L D F E Q E M A T A A S S S S L E K-COOH
Int
m/z
100
75
50
25
0
250
500
750
1,000
1,250
1,500
1,750
2,000
2,250
iY
iF

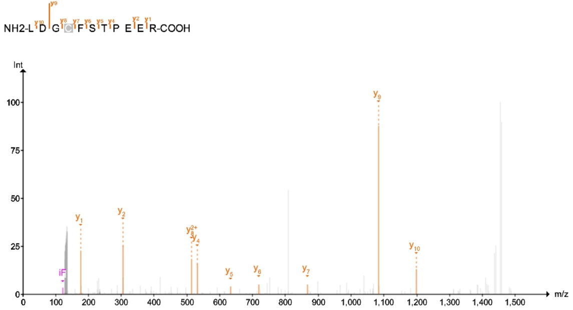

NH2-L D G C F S T P E E R-COOH
y10
y9
y8
y7
y6
y5
y4
y2
y1
Int
100
75
50
25
0
y1
y2
y8 2+
y4
y5
y6
y7
y9
y10
iF
m/z
0
100
200
300
400
500
600
700
800
900
1,000
1,100
1,200
1,300
1,400
1,500

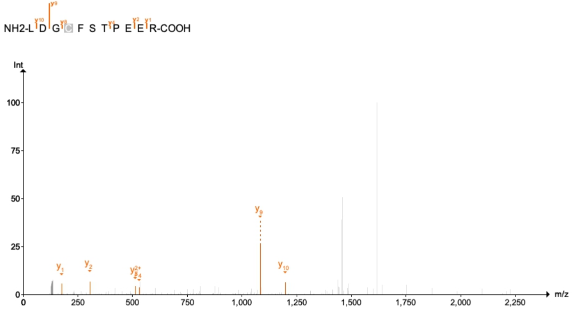
NH2-L D G C F S T P E E R-COOH
y10
y9
y8
y4
y2
y1
Int
100
75
50
25
0
y9
y1
y2
y8 2+
y4
y10
0
250
500
750
1,000
1,250
1,500
1,750
2,000
2,250
m/z

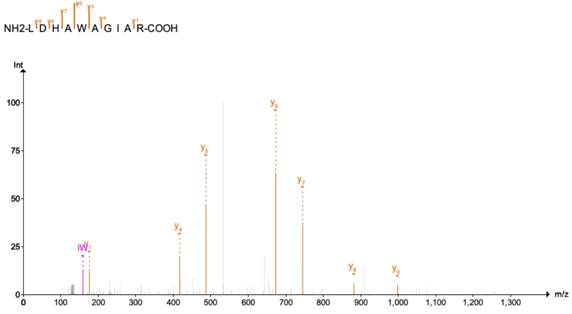

NH2-L D H A W A G I A R-COOH
y9 y8 y7 y6 y5 y4 y1
Int
100
75
50
25
0
y6
y5
y7
y4
IW
y1
y8
y9
0 100 200 300 400 500 600 700 800 900 1,000 1,100 1,200 1,300
m/z

NH2-L D I T T L T G V P E E H I K-COOH

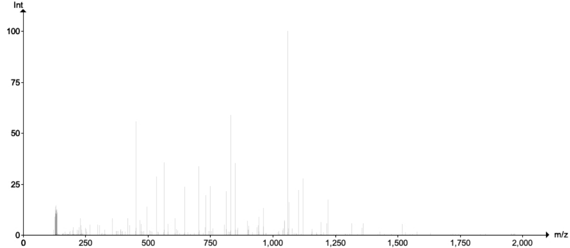

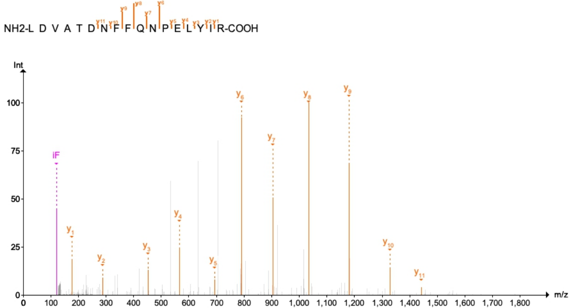

NH2-L D V A T D N F F Q N P E L Y I R-COOH
y11 y10 y9 y8 y7 y6 y5 y4 y3 y2 y1
Int
100
75
50
25
0
iF
y1
y2
y3
y4
y5
y6
y7
y8
y9
y10
y11
0 100 200 300 400 500 600 700 800 900 1,000 1,100 1,200 1,300 1,400 1,500 1,600 1,700 1,800
m/z

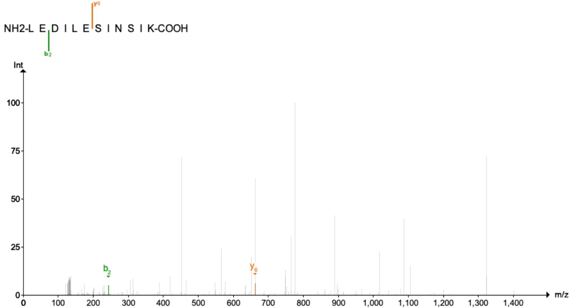
NH2-L E D I L E S I N S I K-COOH
y6
b2
Int
100
75
50
25
0
m/z
0
100
200
300
400
500
600
700
800
900
1,000
1,100
1,200
1,300
1,400

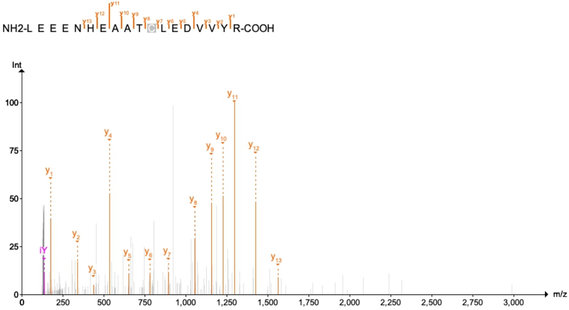

NH2-L E E E N H E A A T C L E D V V Y R-COOH
y13 y12 y11 y10 y9 y8 y7 y6 y5 y4 y3 y2 y1
Int
100
75
50
25
0
y1
y2
y3
y4
y5
y6
y7
y8
y9
y10
y11
y12
y13
iY
0 250 500 750 1,000 1,250 1,500 1,750 2,000 2,250 2,500 2,750 3,000
m/z

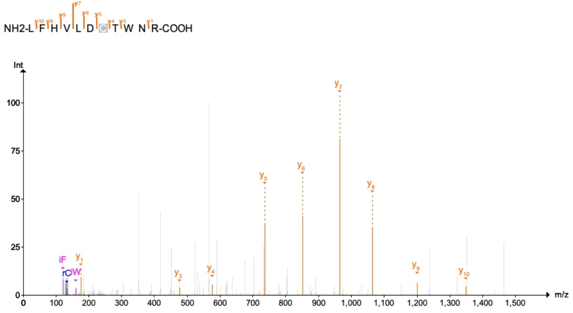

NH2-L F H V L D C T W N R-COOH
y10 y9 y8 y7 y6 y5 y4 y3 y1
Int
100
75
50
25
0
y7
y5
y6
y8
y1
iF
rC
iW
y3
y4
y9
y10
m/z
0 100 200 300 400 500 600 700 800 900 1,000 1,100 1,200 1,300 1,400 1,500

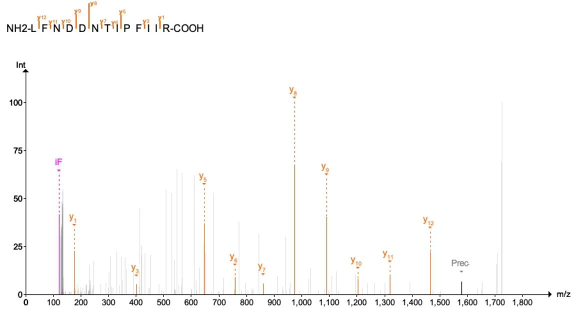

NH2-L F N D D N T I P F I I R-COOH
Int
m/z
iF
y1
y3
y5
y6
y7
y8
y9
y10
y11
y12
Prec

NH2-L F T E V E G T C T G K-COOH

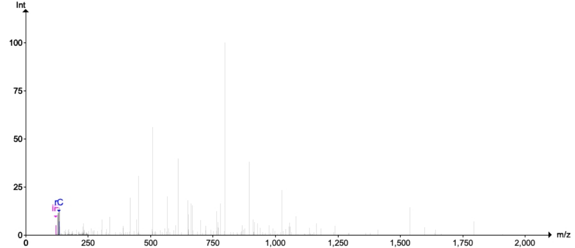

NH2-L G F G S F V E K-COOH

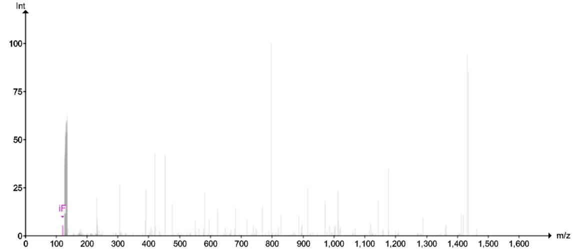

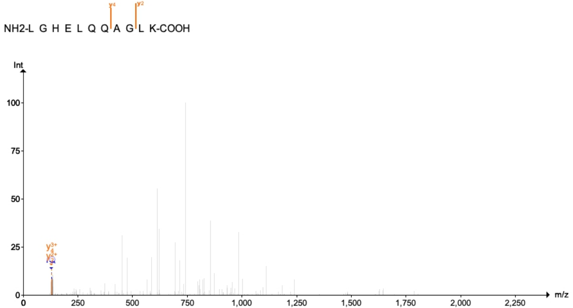

y4
y2
NH2-L G H E L Q Q A G L K-COOH
Int
100
75
50
25
0
y_4^{3+}
y_2^{2+}
0
250
500
750
1,000
1,250
1,500
1,750
2,000
2,250
m/z

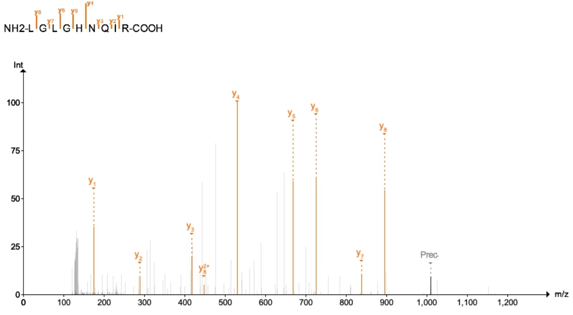

NH2-L G L G H N Q I R-COOH
y8
y7
y6
y5
y4
y3
y2
y1
Int
100
75
50
25
0
y4
y5
y6
y8
y1
y3
y2
y8 2+
y7
Prec
0
100
200
300
400
500
600
700
800
900
1,000
1,100
1,200
m/z

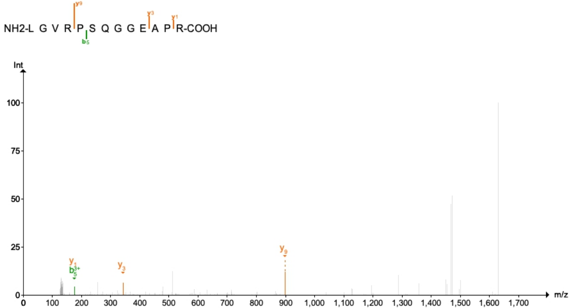

NH2-L G V R P S Q G G E A P R-COOH
y9
b5
y3
y1
Int
100
75
50
25
0
y1
b5 3+
y3
y9
0
100
200
300
400
500
600
700
800
900
1,000
1,100
1,200
1,300
1,400
1,500
1,600
1,700
m/z

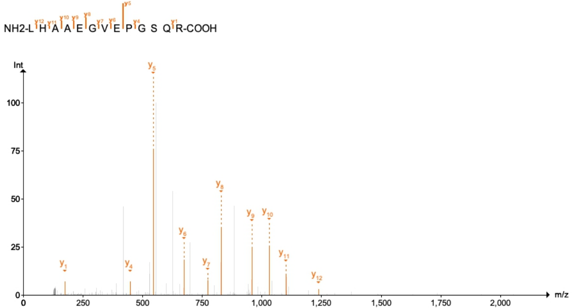

NH2-L H A A E G V E P G S Q R-COOH
y12 y11 y10 y9 y8 y7 y6 y5 y4 y1
Int
m/z
y5
y8
y6
y9
y10
y11
y7
y12
y1
y4
0
25
50
75
100
250
500
750
1,000
1,250
1,500
1,750
2,000

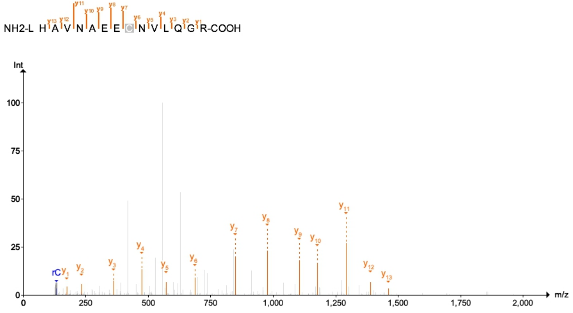

NH2-L H A V N A E E C N V L Q G R-COOH
y13 y12 y11 y10 y9 y8 y7 y6 y5 y4 y3 y2 y1
Int
100
75
50
25
0
rC
y1
y2
y3
y4
y5
y6
y7
y8
y9
y10
y11
y12
y13
0
250
500
750
1,000
1,250
1,500
1,750
2,000
m/z

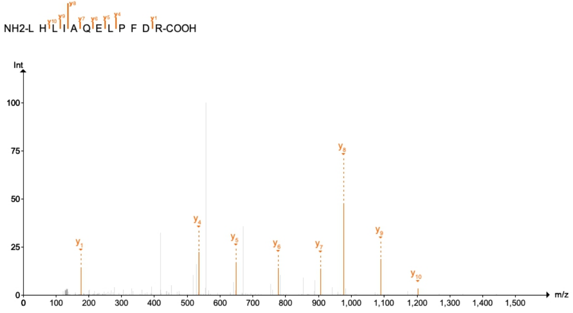
NH2-L H L I A Q E L P F D R-COOH
y10 y9 y8 y7 y6 y5 y4 y1
Int
100
75
50
25
0
y1
y4
y5
y6
y7
y8
y9
y10
0 100 200 300 400 500 600 700 800 900 1,000 1,100 1,200 1,300 1,400 1,500
m/z

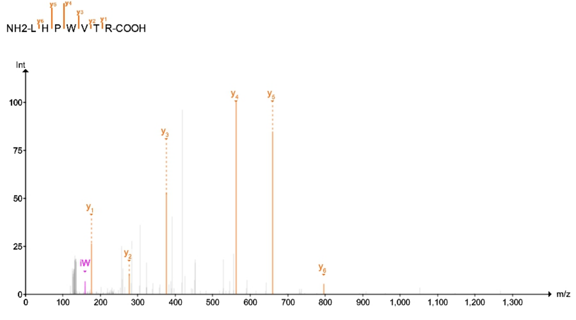

NH2-L H P W V T R-COOH
y6 y5 y4 y3 y2 y1
Int
100
75
50
25
0
y4
y5
y3
y1
y2
iW
y6
0 100 200 300 400 500 600 700 800 900 1,000 1,100 1,200 1,300
m/z

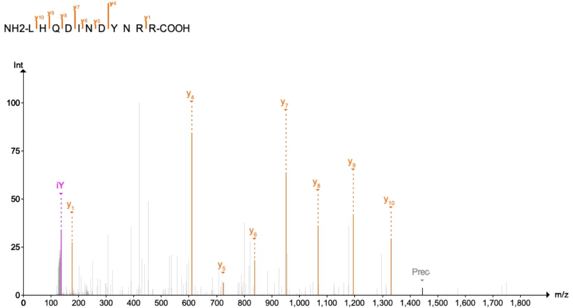
NH2-L H Q D I N D Y N R R-COOH
y10
y9
y8
y7
y6
y5
y4
y1
Int
100
75
50
25
0
IY
y1
y4
y5
y6
y7
y8
y9
y10
Prec
m/z
0
100
200
300
400
500
600
700
800
900
1,000
1,100
1,200
1,300
1,400
1,500
1,600
1,700
1,800

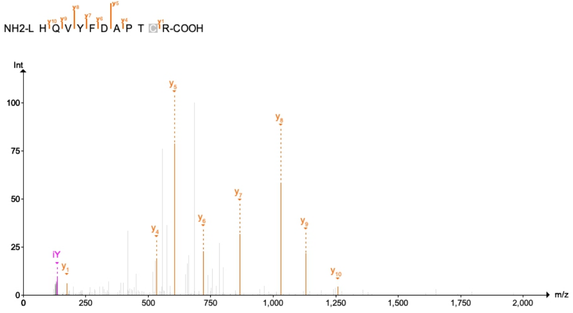
NH2-L H Q V Y F D A P T C R-COOH
y10 y9 y8 y7 y6 y5 y4 y1
Int
100
75
50
25
0
iY
y1
y4
y5
y6
y7
y8
y9
y10
0 250 500 750 1,000 1,250 1,500 1,750 2,000
m/z

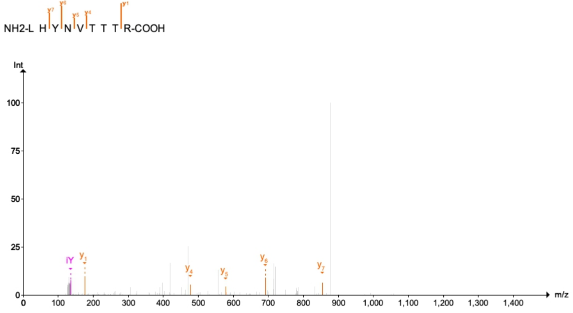
NH2-L H Y N V T T T R-COOH
y7
y6
y5
y4
y1
Int
100
75
50
25
0
iY
y1
y4
y5
y6
y7
0
100
200
300
400
500
600
700
800
900
1,000
1,100
1,200
1,300
1,400
m/z

NH2-L I D F G I A H K-COOH

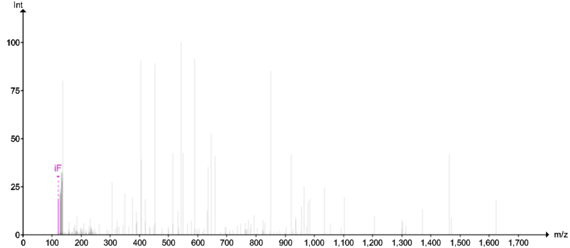

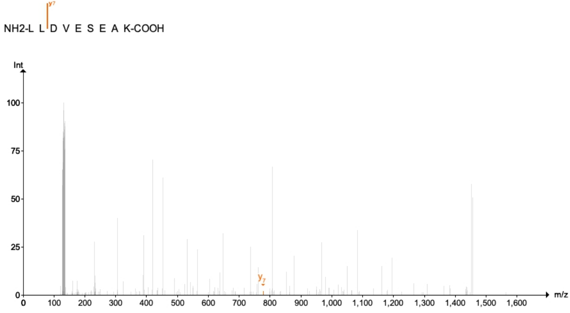
y7
NH2-L L D V E S E A K-COOH
Int
100
75
50
25
0
y7
0
100
200
300
400
500
600
700
800
900
1,000
1,100
1,200
1,300
1,400
1,500
1,600
m/z

NH2-L L E H S F I K-COOH

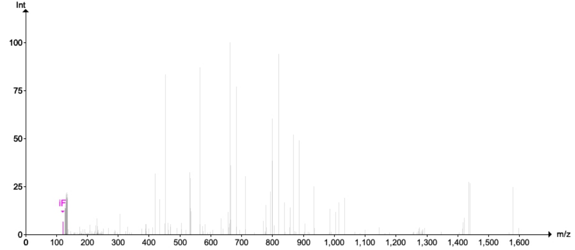

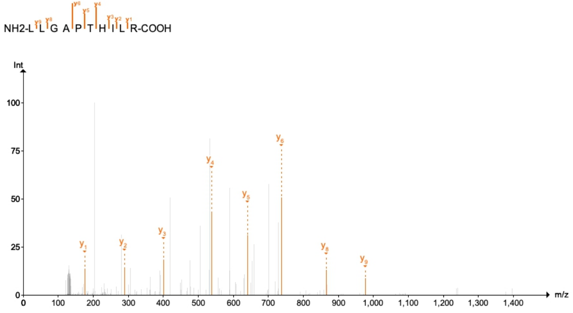

NH2-L L G A P T H I L R-COOH
y9 y8 y6 y5 y4 y3 y2 y1
Int
100
75
50
25
0
y1
y2
y3
y4
y5
y6
y8
y9
0 100 200 300 400 500 600 700 800 900 1,000 1,100 1,200 1,300 1,400
m/z

NH2-L L L Q Q N K-COOH

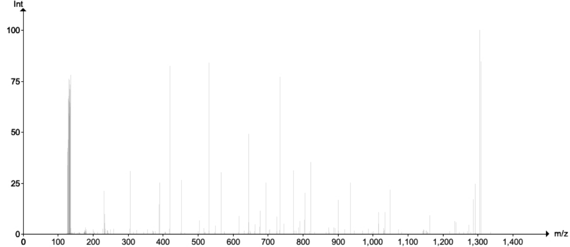

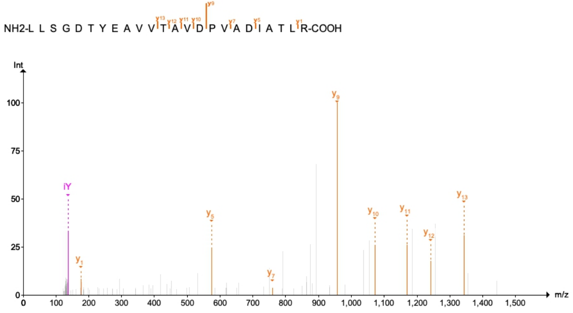
NH2-L L S G D T Y E A V V T A V D P V A D I A T L R-COOH
y13 y12 y11 y10 y9 y7 y5 y1
Int
100
75
50
25
0
iY
y1
y5
y7
y9
y10
y11
y12
y13
0 100 200 300 400 500 600 700 800 900 1,000 1,100 1,200 1,300 1,400 1,500
m/z

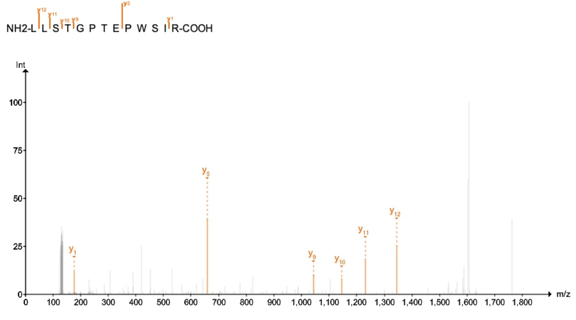
NH2-L L S T G P T E P W S I R-COOH
y12
y11
y10
y9
y5
y1
Int
100
75
50
25
0
m/z
0
100
200
300
400
500
600
700
800
900
1,000
1,100
1,200
1,300
1,400
1,500
1,600
1,700
1,800

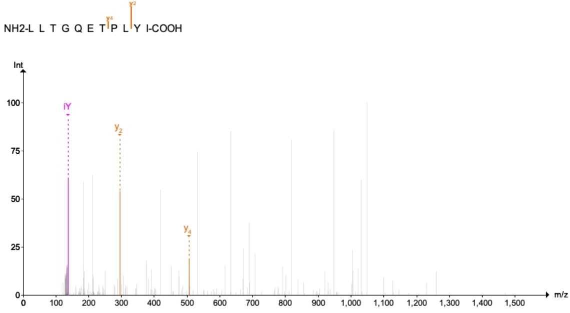
NH2-L L T G Q E T P L Y I-COOH
y4
y2
Int
IY
y2
y4
100
75
50
25
0
0
100
200
300
400
500
600
700
800
900
1,000
1,100
1,200
1,300
1,400
1,500
m/z

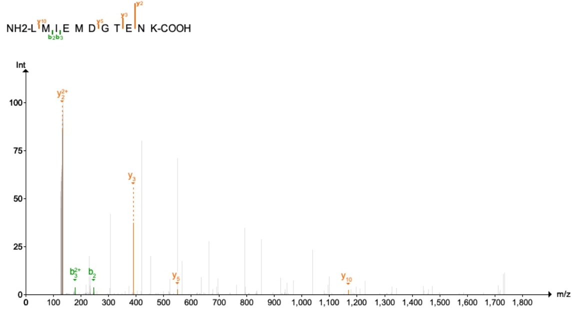

NH2-L M I E M D G T E N K-COOH
y10
b2
b3
y5
y3
y2
Int
m/z
y2 2+
y3
y5
y10
b3 2+
b2
0
25
50
75
100
0
100
200
300
400
500
600
700
800
900
1,000
1,100
1,200
1,300
1,400
1,500
1,600
1,700
1,800

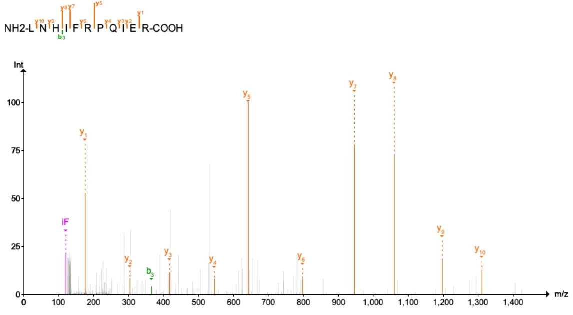
NH2-L N H I F R P Q I E R-COOH
Int
m/z
y1
y2
y3
y4
y5
y6
y7
y8
y9
y10
b3
iF
0
25
50
75
100
0
100
200
300
400
500
600
700
800
900
1,000
1,100
1,200
1,300
1,400

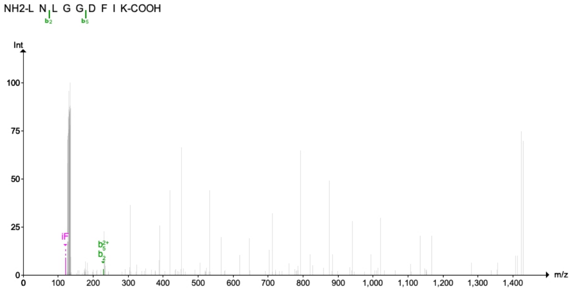
NH2-L N L G G D F I K-COOH
b2
b5
Int
100
75
50
25
0
iF
b_5^{2+}
b_2
0
100
200
300
400
500
600
700
800
900
1,000
1,100
1,200
1,300
1,400
m/z

NH2-L N N A P V A G Y E G D V G S K-COOH

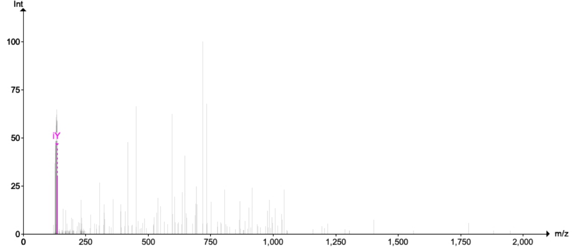

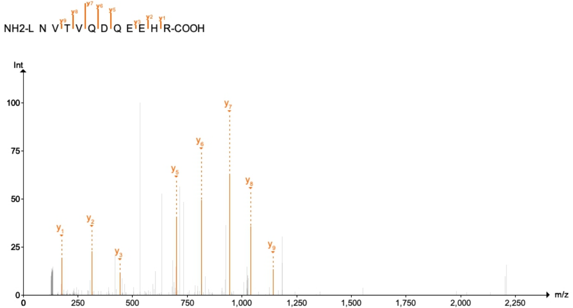
NH2-L N V T V Q D Q E E H R-COOH
y9 y8 y7 y6 y5 y3 y2 y1
Int
100
75
50
25
0
y1
y2
y3
y5
y6
y7
y8
y9
0
250
500
750
1,000
1,250
1,500
1,750
2,000
2,250
m/z

NH2-L P S R P P P H Y P G I K-COOH

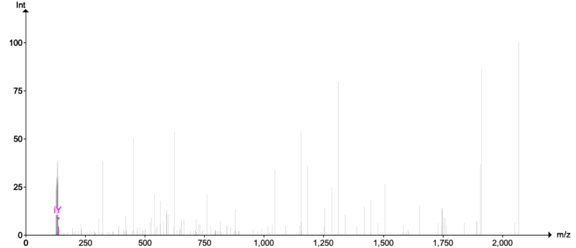

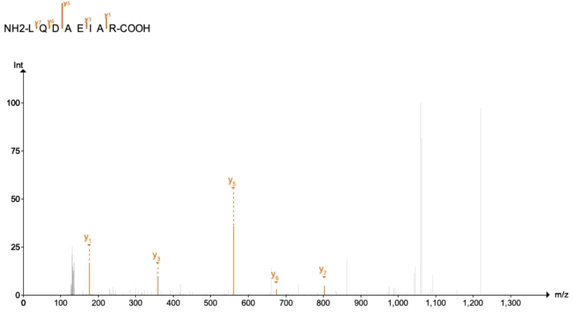
NH2-L Q D A E I A R-COOH
y7 y6 y5 y3 y1
Int
100
75
50
25
0
y1
y3
y5
y6
y7
0 100 200 300 400 500 600 700 800 900 1,000 1,100 1,200 1,300
m/z

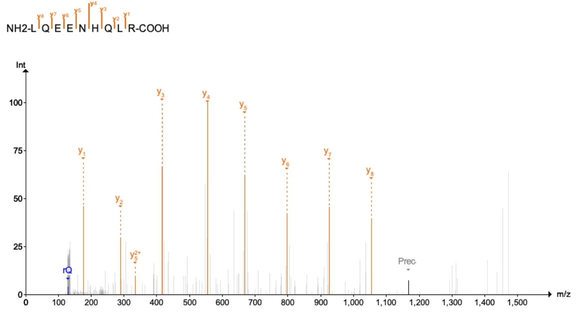

NH2-L Q E E N H Q L R-COOH
y8 y7 y6 y5 y4 y3 y2 y1
Int
100
75
50
25
0
y1
y2
y$_5^{2+}$
y3
y4
y5
y6
y7
y8
rQ
Prec
0 100 200 300 400 500 600 700 800 900 1,000 1,100 1,200 1,300 1,400 1,500
m/z

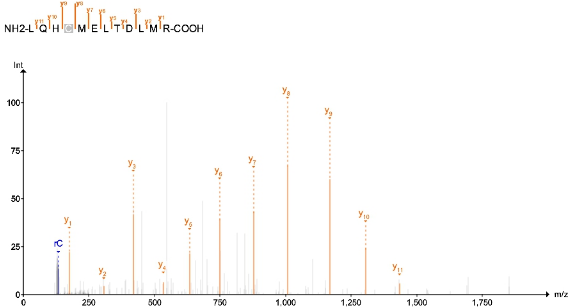

NH2-L Q H C M E L T D L M R-COOH
y11 y10 y9 y8 y7 y6 y5 y4 y3 y2 y1
Int
m/z
rC
y1
y2
y3
y4
y5
y6
y7
y8
y9
y10
y11
0
25
50
75
100
250
500
750
1,000
1,250
1,500
1,750

NH2-L Q L V G T A A M L L A S K-COOH

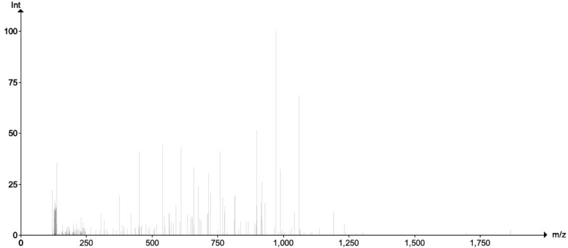

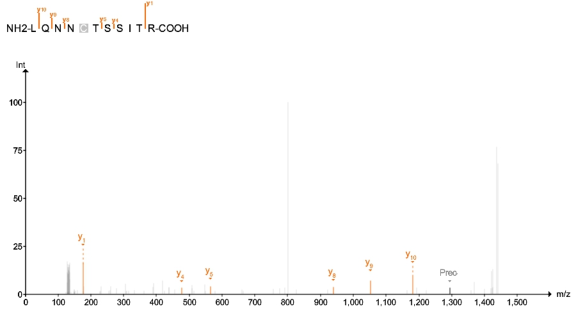
NH2-L Q N N C T S S I T R-COOH
y10
y9
y8
y5
y4
y1
Int
100
75
50
25
0
y1
y4
y5
y8
y9
y10
Prec
0
100
200
300
400
500
600
700
800
900
1,000
1,100
1,200
1,300
1,400
1,500
m/z

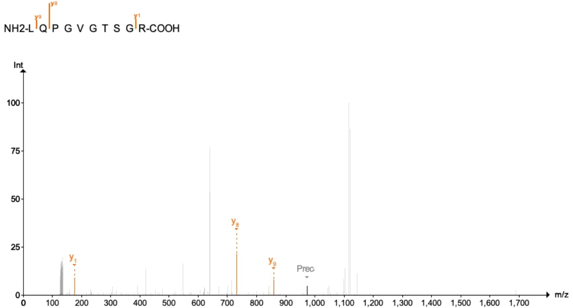
NH2-L Q P G V G T S G R-COOH
y9
y8
y1
Int
100
75
50
25
0
y1
y8
y9
Prec
0
100
200
300
400
500
600
700
800
900
1,000
1,100
1,200
1,300
1,400
1,500
1,600
1,700
m/z

NH2-L Q V L P E L L F Q S T P K-COOH

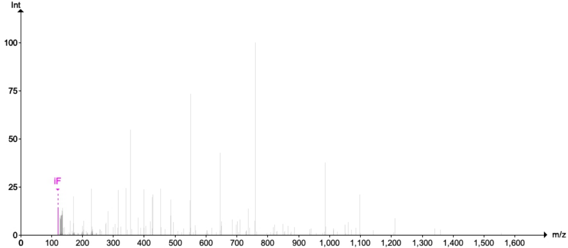

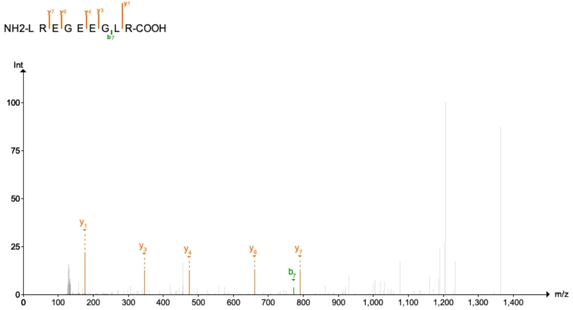
NH2-L R E G E E G L R-COOH
y7
y6
y4
y3
y1
b7
Int
100
75
50
25
0
y1
y3
y4
y6
y7
b7
0
100
200
300
400
500
600
700
800
900
1,000
1,100
1,200
1,300
1,400
m/z

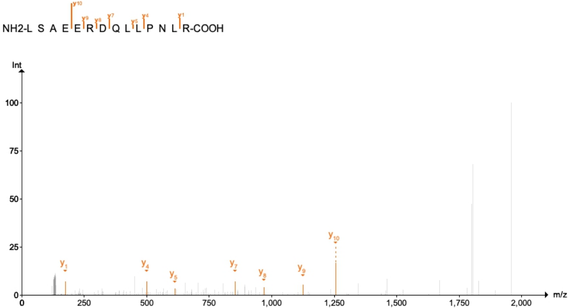

NH2-L S A E E R D Q L L P N L R-COOH
y10
y9
y8
y7
y5
y4
y1
Int
100
75
50
25
0
250
500
750
1,000
1,250
1,500
1,750
2,000
m/z
y1
y4
y5
y7
y8
y9
y10

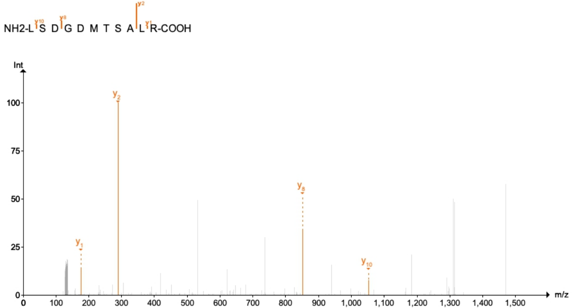
NH2-L S D G D M T S A L R-COOH
y10
y8
y2
y1
Int
m/z
y1
y2
y8
y10
0
25
50
75
100
0
100
200
300
400
500
600
700
800
900
1,000
1,100
1,200
1,300
1,400
1,500

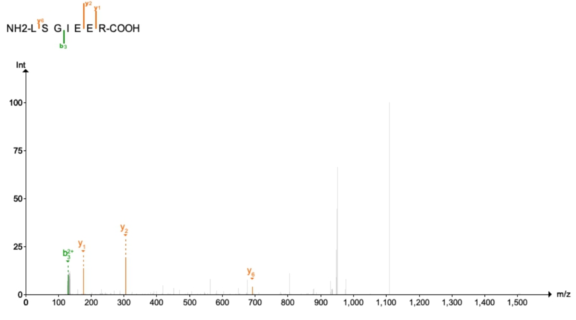

NH2-L S G I E E R-COOH
y6
y2
y1
b3
Int
m/z
b_3^{2+}
y_1
y_2
y_6

NH2-L S P A Q G P P Q P Q S S A K-COOH

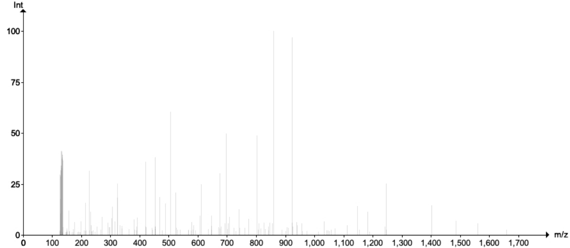

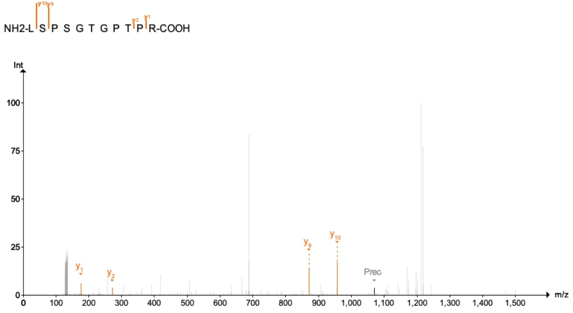

NH2-L S P S G T G P T P R-COOH
y10
y9
y2
y1
Int
100
75
50
25
0
y1
y2
y9
y10
Prec
0
100
200
300
400
500
600
700
800
900
1,000
1,100
1,200
1,300
1,400
1,500
m/z

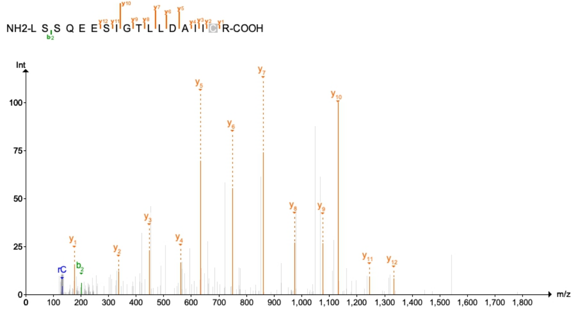
NH2-L S S Q E E S I G T L L D A I I C R-COOH
Int
m/z
rC
b_2
y_1
y_2
y_3
y_4
y_5
y_6
y_7
y_8
y_9
y_{10}
y_{11}
y_{12}

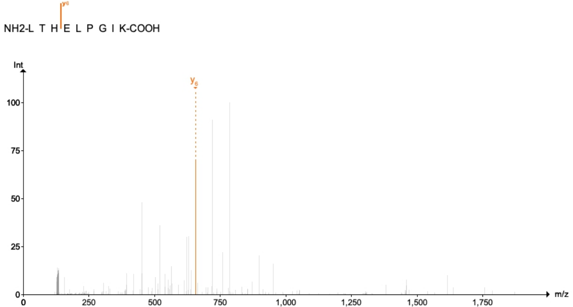

NH2-L T H E L P G I K-COOH
y_6
Int
y_6
100
75
50
25
0
0
250
500
750
1,000
1,250
1,500
1,750
m/z

NH2-L T L L H Y D P V V K-COOH

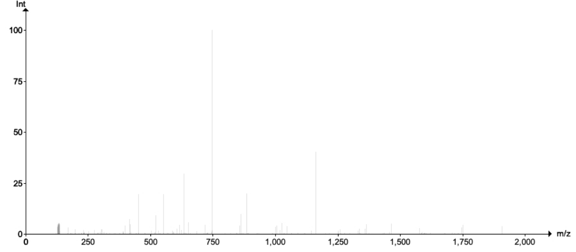

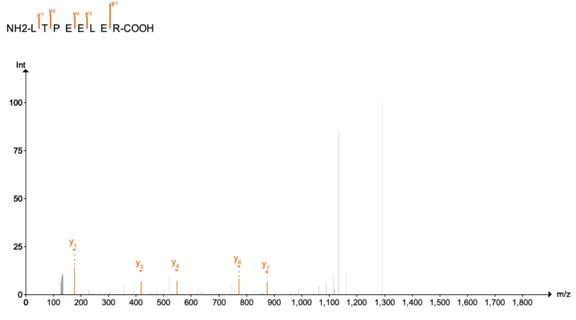

NH2-L T P E E L E R-COOH
y7
y6
y4
y3
y1
Int
100
75
50
25
0
y1
y3
y4
y6
y7
0
100
200
300
400
500
600
700
800
900
1,000
1,100
1,200
1,300
1,400
1,500
1,600
1,700
1,800
m/z

NH2-L T P Q H D Q I Q T Q P L G K-COOH

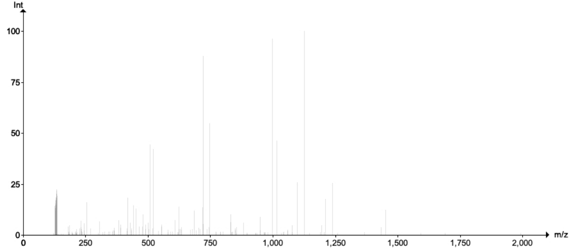

NH2-L T V D E L K-COOH

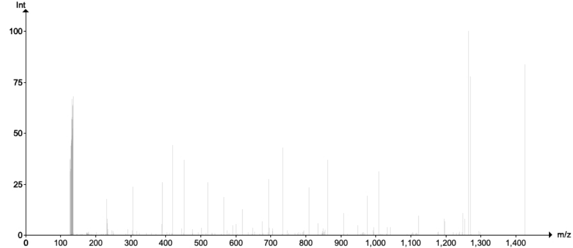

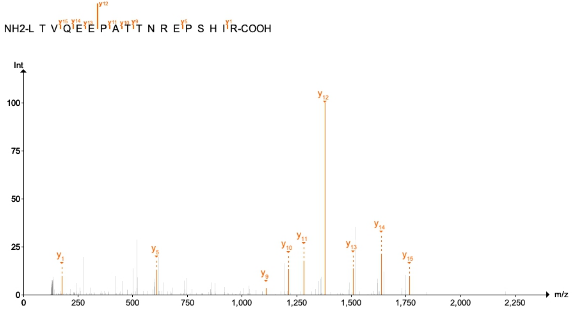
NH2-L T V Q E E P A T T N R E P S H I R-COOH
y15 y14 y13 y12 y11 y10 y9 y5 y1
Int
100
75
50
25
0
250
500
750
1,000
1,250
1,500
1,750
2,000
2,250
m/z
y1
y5
y9
y10
y11
y12
y13
y14
y15

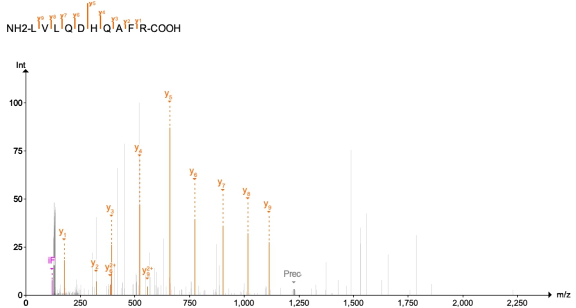
NH2-L V L Q D H Q A F R-COOH
y9 y8 y7 y6 y5 y4 y3 y2 y1
Int
100
75
50
25
0
y5
y4
y6
y7
y8
y9
y3
y1
y2
y5 2+
y9 2+
iF
Prec
250
500
750
1,000
1,250
1,500
1,750
2,000
2,250
m/z

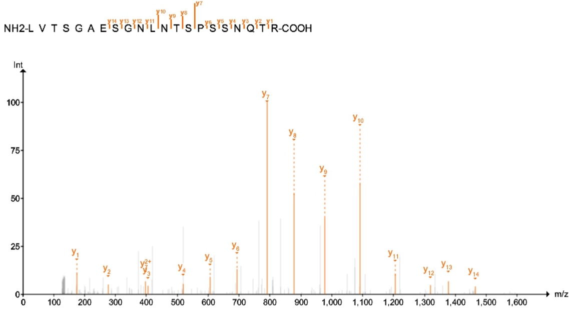

NH2-L V T S G A E S G N L N T S P S S N Q T R-COOH
y14 y13 y12 y11 y10 y9 y8 y7 y6 y5 y4 y3 y2 y1
Int
100
75
50
25
0
y1
y2
y7 2+
y3
y4
y5
y6
y7
y8
y9
y10
y11
y12
y13
y14
0 100 200 300 400 500 600 700 800 900 1,000 1,100 1,200 1,300 1,400 1,500 1,600
m/z

NH2-L Y H E A T T D T Y K-COOH

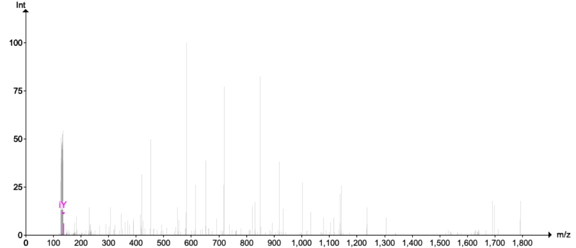

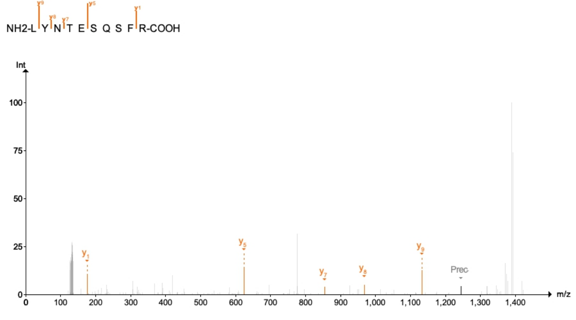

NH2-L Y N T E S Q S F R-COOH
y9
y8
y7
y5
y1
Int
100
75
50
25
0
y1
y5
y7
y8
y9
Prec
0
100
200
300
400
500
600
700
800
900
1,000
1,100
1,200
1,300
1,400
m/z

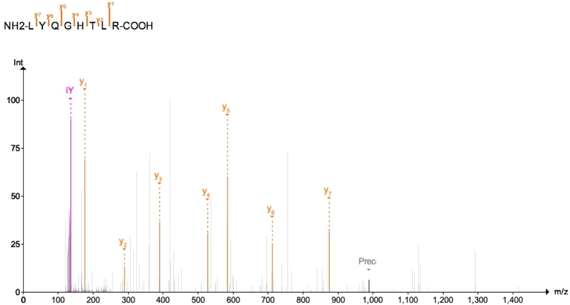

NH2-L Y Q G H T L R-COOH
y7 y6 y5 y4 y3 y2 y1
Int
m/z
IY
y1
y2
y3
y4
y5
y6
y7
Prec
0
25
50
75
100
0 100 200 300 400 500 600 700 800 900 1,000 1,100 1,200 1,300 1,400

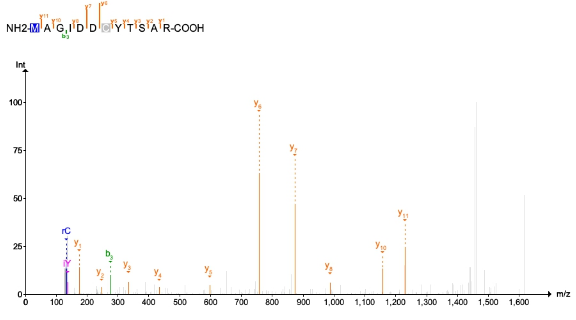
NH2-M A G I D D C Y T S A R-COOH
Int
m/z
y1
y2
y3
y4
y5
y6
y7
y8
y10
y11
b3
rC
iY
0
25
50
75
100
0
100
200
300
400
500
600
700
800
900
1,000
1,100
1,200
1,300
1,400
1,500
1,600

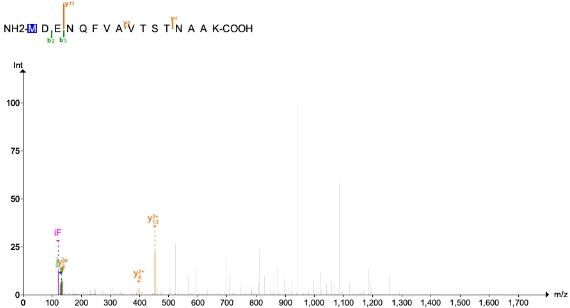
NH2-M D E N Q F V A V T S T N A A K-COOH
y13
y8
y4
b2
b3
Int
100
75
50
25
0
iF
y13 3+
y8 2+
y4 3+
0
100
200
300
400
500
600
700
800
900
1,000
1,100
1,200
1,300
1,400
1,500
1,600
1,700
m/z

NH2-M D E V L Y S I A E K-COOH

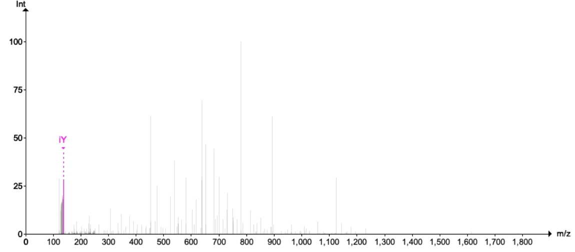

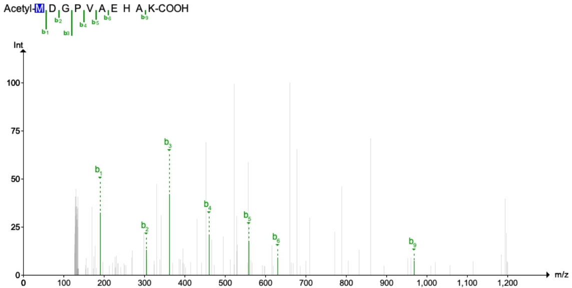
Acetyl-M D G P V A E H A K-COOH
b1
b2
b3
b4
b5
b6
b9
Int
100
75
50
25
0
0
100
200
300
400
500
600
700
800
900
1,000
1,100
1,200
m/z

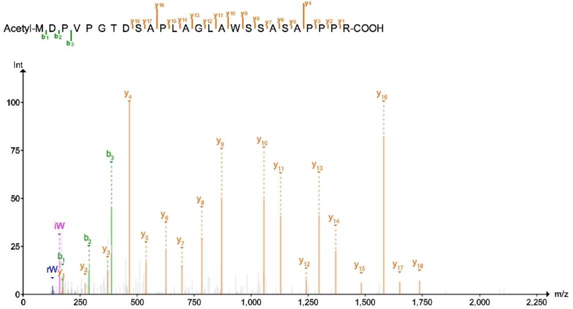

Acetyl-M D P V P G T D S A P L A G L A W S S A S A P P P R-COOH
Int
m/z

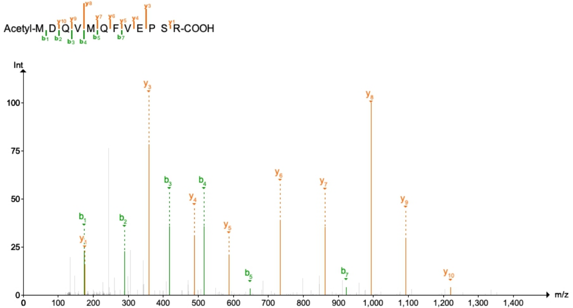

Acetyl-M D Q V M Q F V E P S R-COOH
Int
m/z

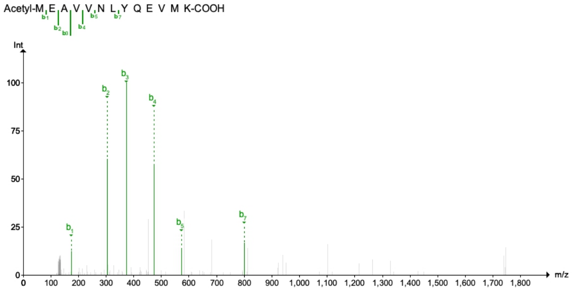

Acetyl-M E A V V N L Y Q E V M K-COOH
b1
b2
b3
b4
b5
b7
Int
100
75
50
25
0
b1
b2
b3
b4
b5
b7
0
100
200
300
400
500
600
700
800
900
1,000
1,100
1,200
1,300
1,400
1,500
1,600
1,700
1,800
m/z

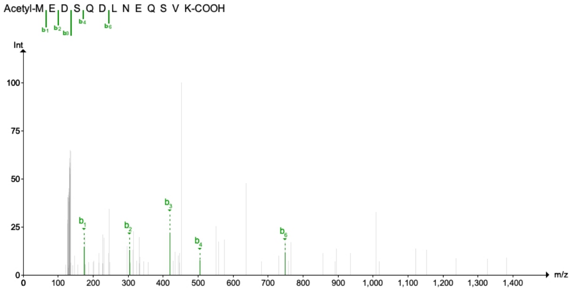
Acetyl-M E D S Q D L N E Q S V K-COOH
b1
b2
b3
b4
b6
Int
100
75
50
25
0
b1
b2
b3
b4
b6
0
100
200
300
400
500
600
700
800
900
1,000
1,100
1,200
1,300
1,400
m/z

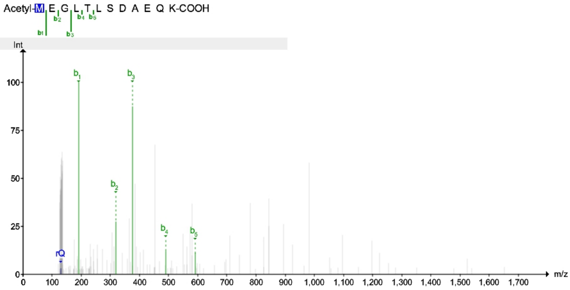
Acetyl-M E G L T L S D A E Q K-COOH
b1
b2
b3
b4
b5
Int
100
75
50
25
0
rQ
m/z
0
100
200
300
400
500
600
700
800
900
1,000
1,100
1,200
1,300
1,400
1,500
1,600
1,700

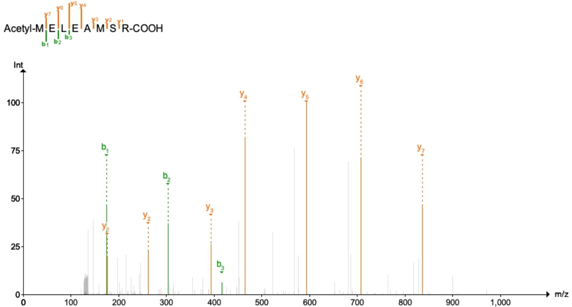

Acetyl-M E L E A M S R-COOH
y7 y6 y5 y4 y3 y2 y1
b1 b2 b3
Int
m/z
0
25
50
75
100
0
100
200
300
400
500
600
700
800
900
1,000
b1
b2
b3
y1
y2
y3
y4
y5
y6
y7

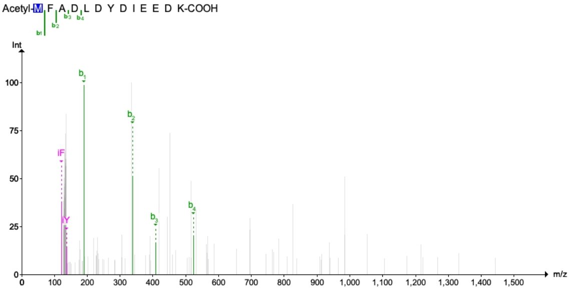

Acetyl-M F A D L D Y D I E E D K-COOH
b1
b2
b3
b4
Int
iF
iY
m/z

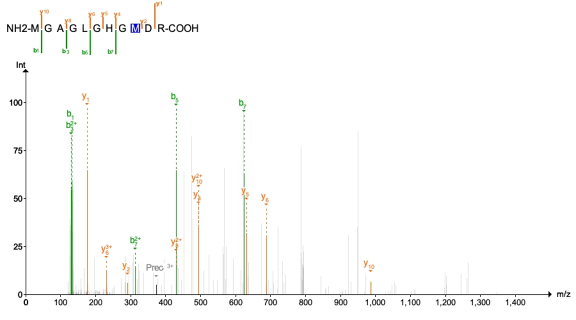

NH2-M G A G L G H G M D R-COOH
Int
m/z
b1
b2+3
y1
b5
b7
y2+10
y4
y5
y6
y3+6
b2+7
y2+3
y2
Prec 3+
y10

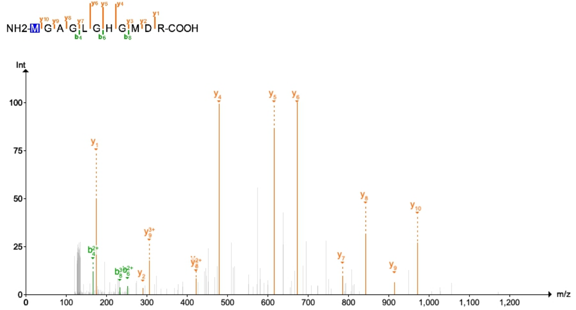

NH2-M G A G L G H G M D R-COOH
Int
m/z
y1
y2
y4
y5
y6
y7
y8
y9
y10
y_9^{3+}
y_8^{2+}
b_4^{2+}
b_6^{2+}
0
25
50
75
100
0
100
200
300
400
500
600
700
800
900
1,000
1,100
1,200

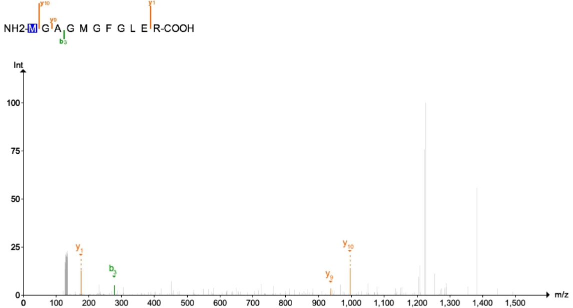

NH2-M G A G M G F G L E R-COOH
y10
y9
b3
y1
Int
100
75
50
25
0
y1
b3
y9
y10
m/z
0
100
200
300
400
500
600
700
800
900
1,000
1,100
1,200
1,300
1,400
1,500

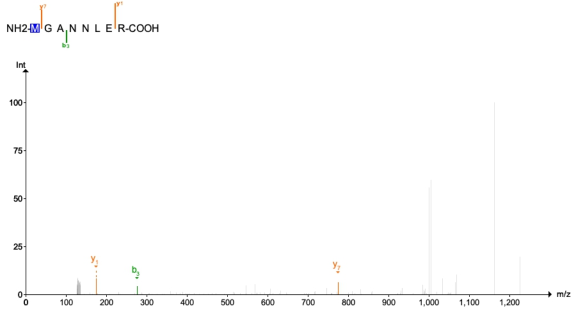
NH2-M G A N N L E R-COOH
y7
y1
b3
Int
100
75
50
25
0
m/z
0
100
200
300
400
500
600
700
800
900
1,000
1,100
1,200

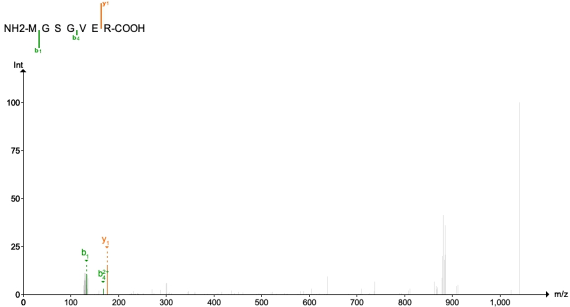

NH2-M G S G V E R-COOH
b1
b4
y1
Int
100
75
50
25
0
0
100
200
300
400
500
600
700
800
900
1,000
m/z
b1
y1
b4 2+

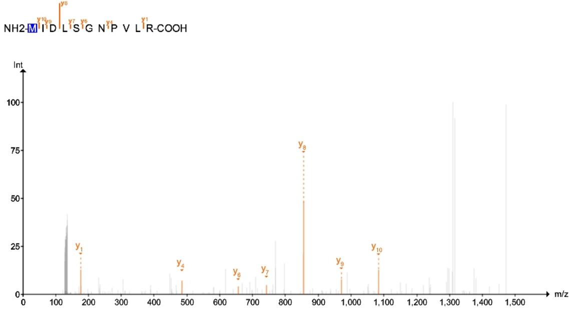
NH2-M I D L S G N P V L R-COOH
Int
m/z
y1
y4
y6
y7
y8
y9
y10

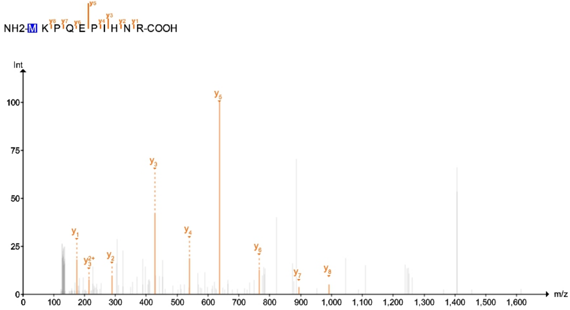

NH2-M K P Q E P I H N R-COOH
y8 y7 y6 y5 y4 y3 y2 y1
Int
m/z
y1
y3 2+
y2
y3
y4
y5
y6
y7
y8
0
25
50
75
100
0 100 200 300 400 500 600 700 800 900 1,000 1,100 1,200 1,300 1,400 1,500 1,600

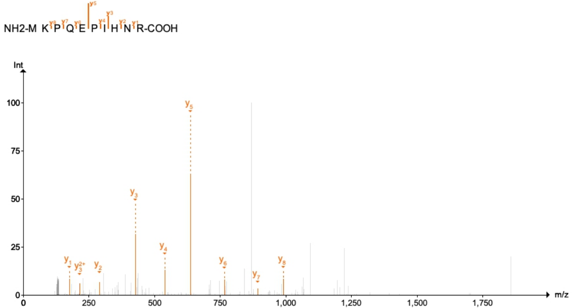

NH2-M K P Q E P I H N R-COOH
y8 y7 y6 y5 y4 y3 y2 y1
Int
m/z
100
75
50
25
0
250
500
750
1,000
1,250
1,500
1,750
y1
y_3^{2+}
y2
y3
y4
y5
y6
y7
y8

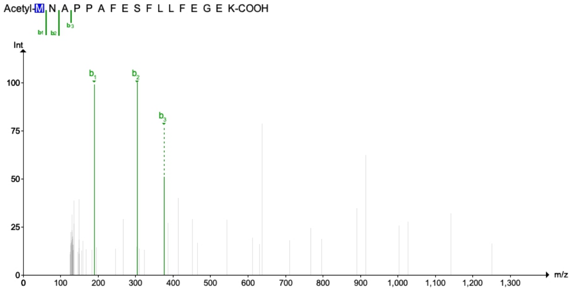

Acetyl-M N A P P A F E S F L L F E G E K-COOH
b1
b2
b3
Int
100
75
50
25
0
m/z
0
100
200
300
400
500
600
700
800
900
1,000
1,100
1,200
1,300

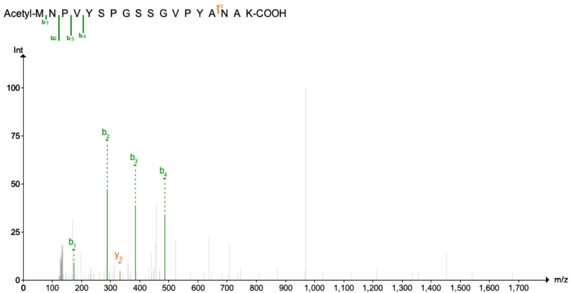

Acetyl-M N P V Y S P G S S G V P Y A N A K-COOH
b1
b2
b3
b4
y3
Int
100
75
50
25
0
b2
b3
b4
b1
y3
0
100
200
300
400
500
600
700
800
900
1,000
1,100
1,200
1,300
1,400
1,500
1,600
1,700
m/z

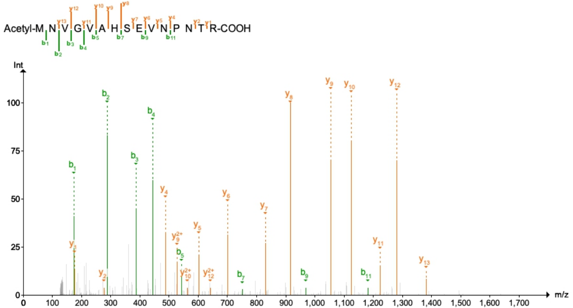
Acetyl-M N V G V A H S E V N P N T R-COOH
Int
m/z
b1
b2
b3
b4
b5
b7
b9
b11
y1
y2
y4
y5
y6
y7
y8
y9
y10
y11
y12
y13
y9 2+
y10 2+
y12 2+
0
25
50
75
100
0
100
200
300
400
500
600
700
800
900
1,000
1,100
1,200
1,300
1,400
1,500
1,600
1,700

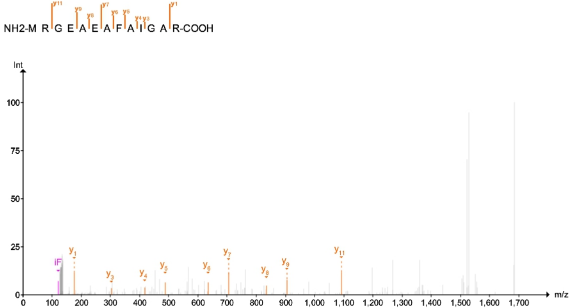
NH2-M R G E A E A F A I G A R-COOH
y11 y9 y8 y7 y6 y5 y4 y3 y1
Int
100
75
50
25
0
iF
y1 y3 y4 y5 y6 y7 y8 y9 y11
0 100 200 300 400 500 600 700 800 900 1,000 1,100 1,200 1,300 1,400 1,500 1,600 1,700
m/z

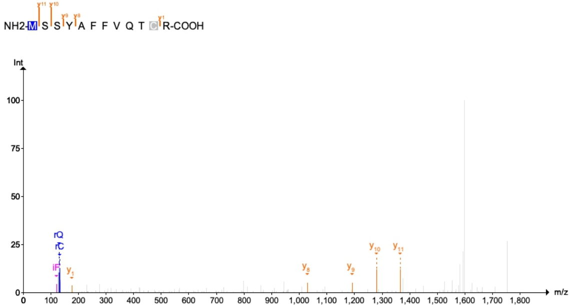

NH2-M S S Y A F F V Q T C R-COOH
y11
y10
y9
y8
y1
Int
100
75
50
25
0
rQ
rC
iF
y1
y8
y9
y10
y11
m/z
0
100
200
300
400
500
600
700
800
900
1,000
1,100
1,200
1,300
1,400
1,500
1,600
1,700
1,800

NH2-M T G T L E T Q F T C P F C N H E K-COOH

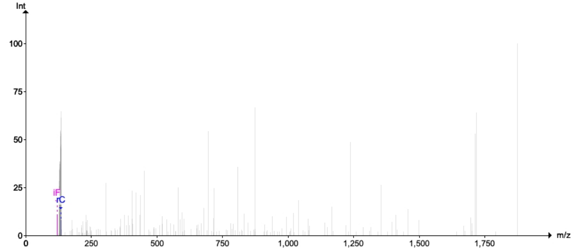

NH2-M T L D E W K-COOH

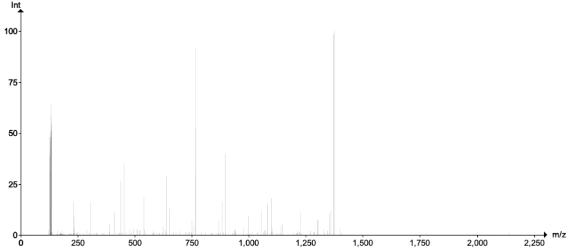

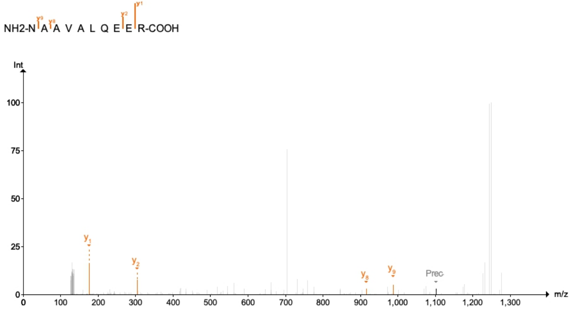

NH2-N A A V A L Q E E R-COOH
y9
y8
y2
y1
Int
100
75
50
25
0
y1
y2
y8
y9
Prec
0
100
200
300
400
500
600
700
800
900
1,000
1,100
1,200
1,300
m/z

NH2-N A L Q E L Q Q I I I T P I K-COOH

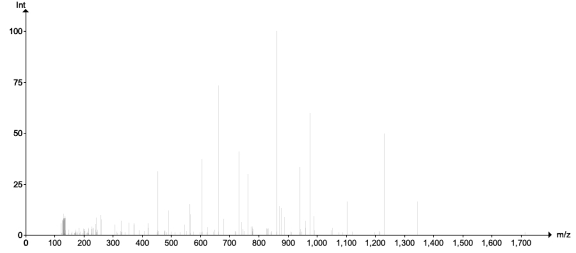

NH2-N A T H E Q A A A A L K-COOH

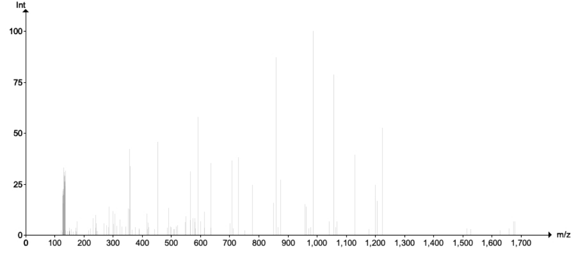

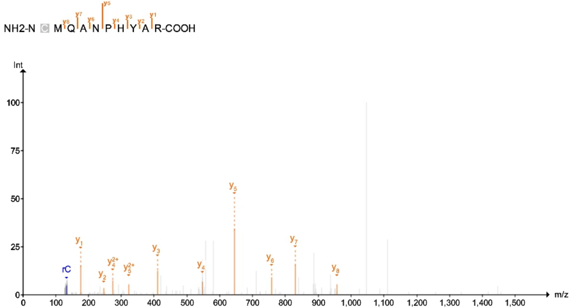
NH2-N C M Q A N P H Y A R-COOH
y8 y7 y6 y5 y4 y3 y2 y1
Int
100
75
50
25
0
rC
y1
y2
y4 2+
y5 2+
y3
y4
y5
y6
y7
y8
m/z
0 100 200 300 400 500 600 700 800 900 1,000 1,100 1,200 1,300 1,400 1,500

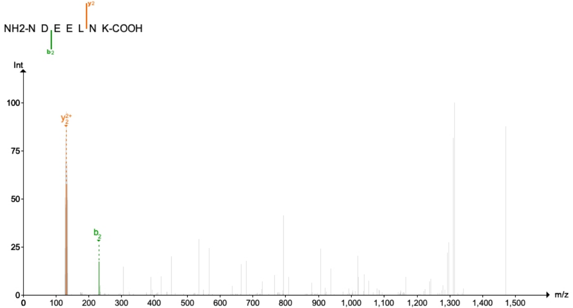

NH2-N D E E L N K-COOH
y2
b2
Int
100
75
50
25
0
y_2^{2+}
b_2
0
100
200
300
400
500
600
700
800
900
1,000
1,100
1,200
1,300
1,400
1,500
m/z

NH2-N E L L P S H P L E L S E K-COOH

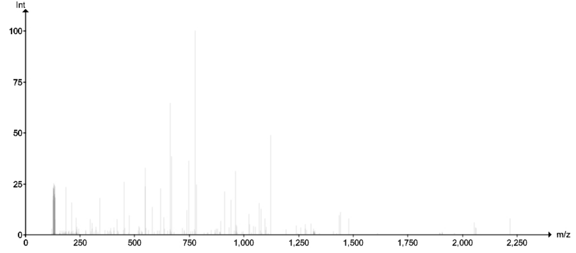

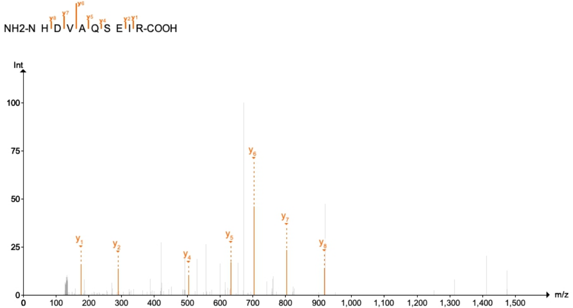
NH2-N H D V A Q S E I R-COOH
y8 y7 y6 y5 y4 y2 y1
Int
100
75
50
25
0
y1
y2
y4
y5
y6
y7
y8
m/z
0 100 200 300 400 500 600 700 800 900 1,000 1,100 1,200 1,300 1,400 1,500

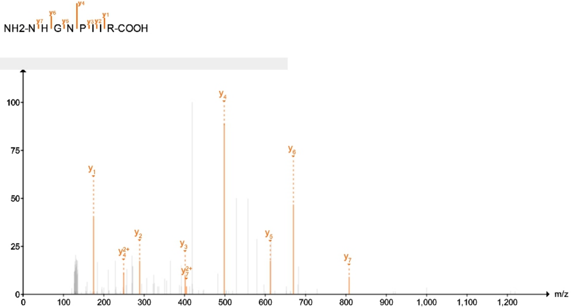
NH2-N H G N P I I R-COOH
y7
y6
y5
y4
y3
y2
y1
100
75
50
25
0
0
100
200
300
400
500
600
700
800
900
1,000
1,100
1,200
m/z
y4
y6
y1
y2
y4 2+
y3
y7 2+
y5
y7

NH2-N H S I I L S A P N P E G K-COOH

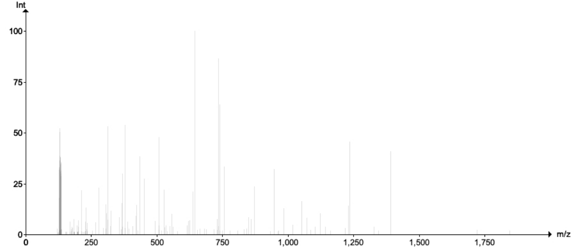

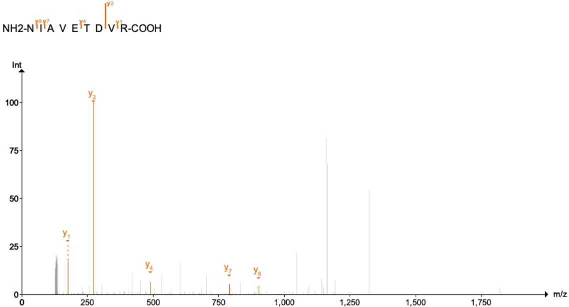
NH2-N I A V E T D V R-COOH
y8 y7 y4 y2 y1
Int
m/z
y1
y2
y4
y7
y8
100
75
50
25
0
0
250
500
750
1,000
1,250
1,500
1,750

NH2-N I C V L A H V D H G K-COOH

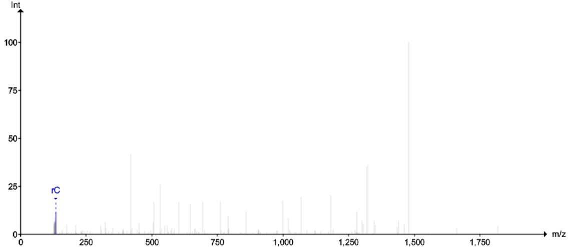

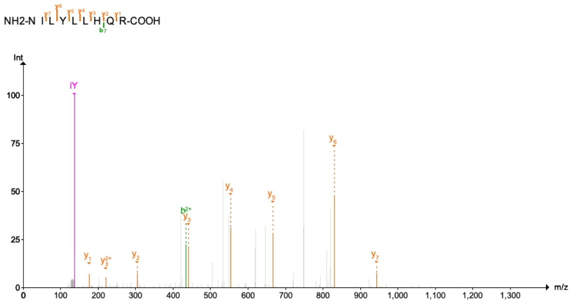
NH2-N I L Y L L H Q R-COOH
y7 y6 y5 y4 y3 y2 y1
b7
Int
m/z
IY
b_7^{2+}
y_1
y_2
y_3
y_3^{2+}
y_4
y_5
y_6
y_7
0
25
50
75
100
0
100
200
300
400
500
600
700
800
900
1,000
1,100
1,200
1,300

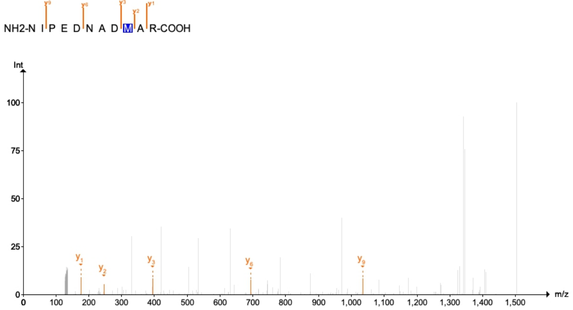
NH2-N I P E D N A D M A R-COOH
y9
y6
y3
y2
y1
Int
100
75
50
25
0
0
100
200
300
400
500
600
700
800
900
1,000
1,100
1,200
1,300
1,400
1,500
m/z
y1
y2
y3
y6
y9

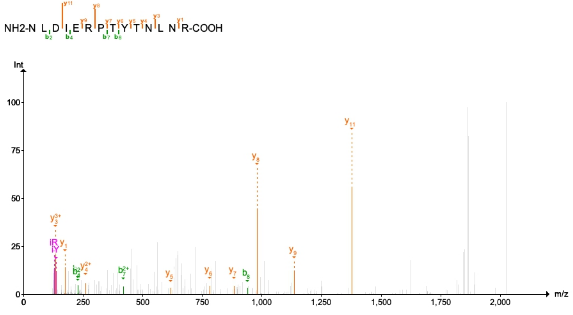
NH2-N L D I E R P T Y T N L N R-COOH
Int
100
75
50
25
0
0
250
500
750
1,000
1,250
1,500
1,750
2,000
m/z
y11
y8
y9
y1
y7
y6
y5
y4
y3
iR
iY
b2
b4
b7
b8

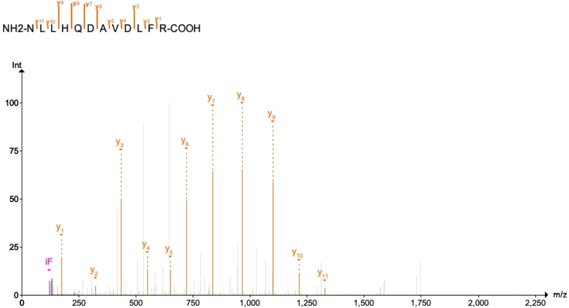
NH2-N L L H Q D A V D L F R-COOH
Int
m/z
y1
y2
y3
y4
y5
y6
y7
y8
y9
y10
y11
iF
0
25
50
75
100
0
250
500
750
1,000
1,250
1,500
1,750
2,000
2,250

NH2-N L P E G V A M E V T K-COOH

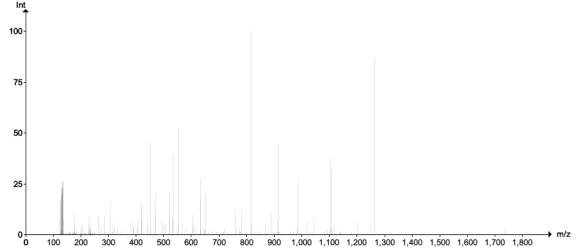

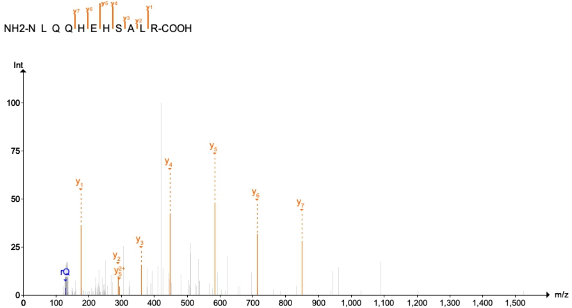

NH2-N L Q Q H E H S A L R-COOH
y7 y6 y5 y4 y3 y2 y1
Int
100
75
50
25
0
y1
y2
y5 2+
rQ
y3
y4
y5
y6
y7
0 100 200 300 400 500 600 700 800 900 1,000 1,100 1,200 1,300 1,400 1,500
m/z

NH2-N L T N L A S N V K-COOH

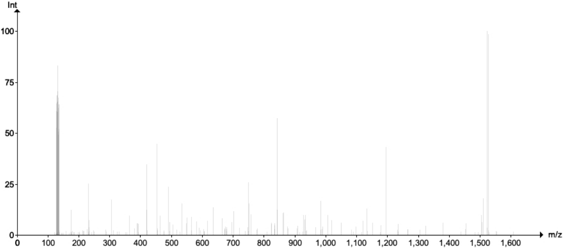

NH2-N L V H S G A T K-COOH

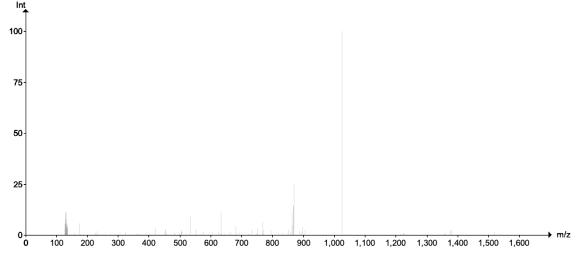

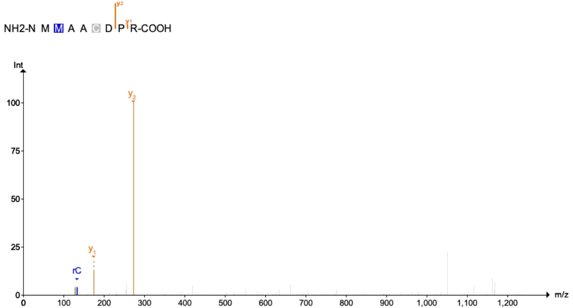
NH2-N M M A A C D P R-COOH
y2
y1
Int
100
75
50
25
0
y2
y1
rC
0
100
200
300
400
500
600
700
800
900
1,000
1,100
1,200
m/z

NH2-N N I D V C P E C G H L K-COOH

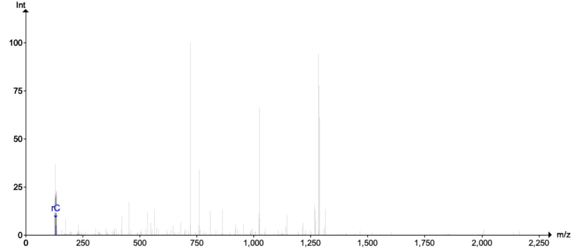

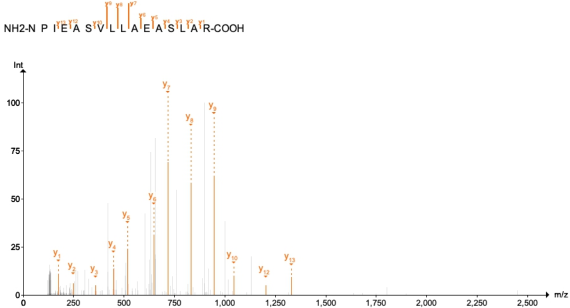

NH2-N P I E A S V L L A E A S L A R-COOH
y13 y12 y10 y9 y8 y7 y6 y5 y4 y3 y2 y1
Int
100
75
50
25
0
y1 y2 y3 y4 y5 y6 y7 y8 y9 y10 y12 y13
0 250 500 750 1,000 1,250 1,500 1,750 2,000 2,250 2,500
m/z

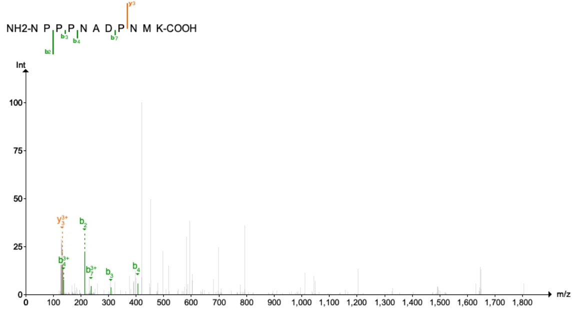
NH2-N P P P N A D P N M K-COOH
b2
b3
b4
b7
y3
Int
m/z
y3 3+
b2
b4 3+
b7 3+
b3
b4
0
25
50
75
100
0
100
200
300
400
500
600
700
800
900
1,000
1,100
1,200
1,300
1,400
1,500
1,600
1,700
1,800

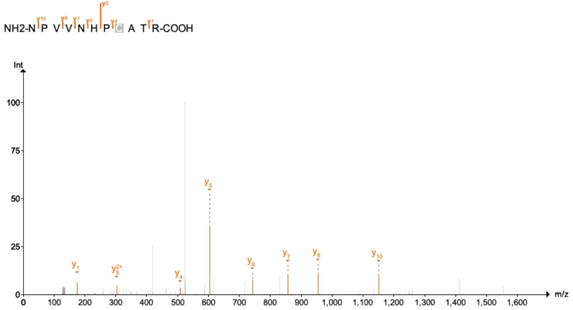
NH2-N P V V N H P C A T R-COOH
y10
y8
y7
y6
y5
y4
y1
Int
100
75
50
25
0
m/z
y1
y5 2+
y4
y5
y6
y7
y8
y10
0
100
200
300
400
500
600
700
800
900
1,000
1,100
1,200
1,300
1,400
1,500
1,600

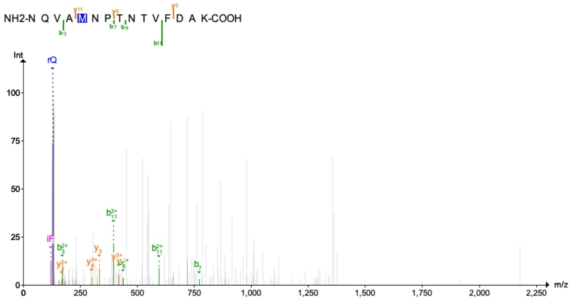
NH2-N Q V A M N P T N T V F D A K-COOH
b3
y11
y8
b7
b8
y3
b11
Int
rQ
iF
b11 3+
b3 2+
y3
y3 2+
y8 3+
y11 3+
y8 2+
b11 2+
b7
m/z
0
25
50
75
100
250
500
750
1,000
1,250
1,500
1,750
2,000
2,250

NH2-N R E P L V K-COOH

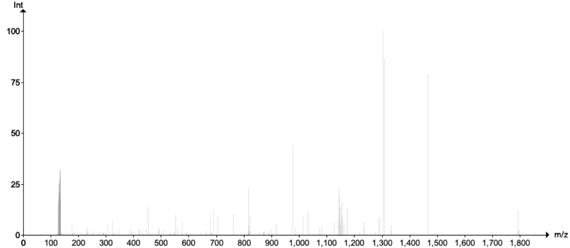

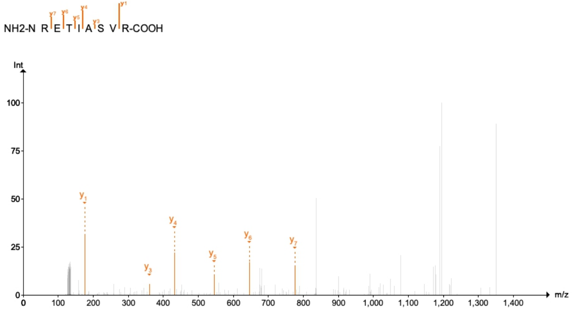
NH2-N R E T I A S V R-COOH
y7
y6
y5
y4
y3
y1
Int
100
75
50
25
0
y1
y3
y4
y5
y6
y7
0
100
200
300
400
500
600
700
800
900
1,000
1,100
1,200
1,300
1,400
m/z

NH2-N R P E A F P T A E D I F A K-COOH

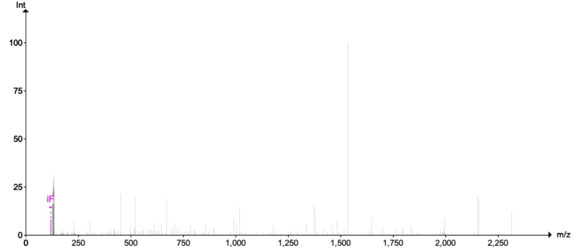

NH2-N R P P L P A G T N S K-COOH

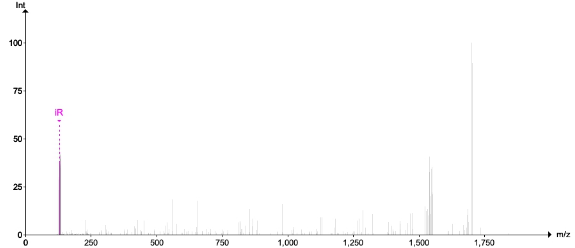

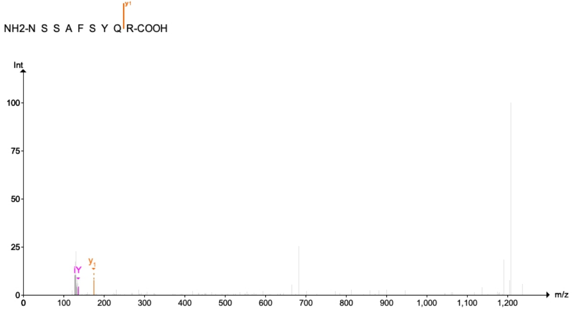

y1
NH2-N S S A F S Y Q R-COOH
Int
100
75
50
25
0
iY
y1
0
100
200
300
400
500
600
700
800
900
1,000
1,100
1,200
m/z

NH2-N S Y N N S Q A P S P G L G S K-COOH

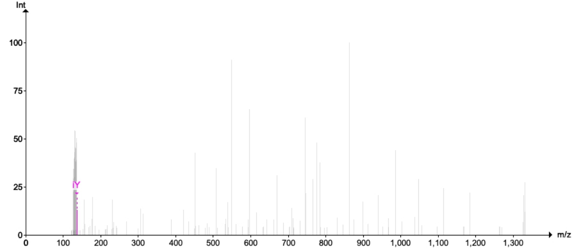

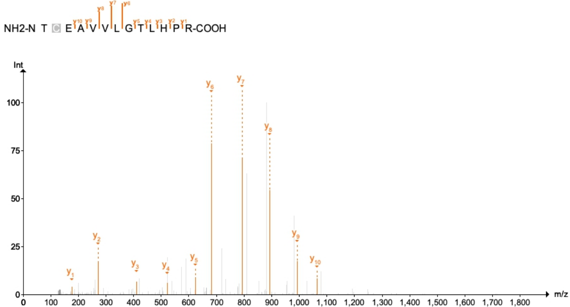
NH2-N T C E A V V L G T L H P R-COOH
Int
m/z
y1
y2
y3
y4
y5
y6
y7
y8
y9
y10
0
25
50
75
100
0
100
200
300
400
500
600
700
800
900
1,000
1,100
1,200
1,300
1,400
1,500
1,600
1,700
1,800

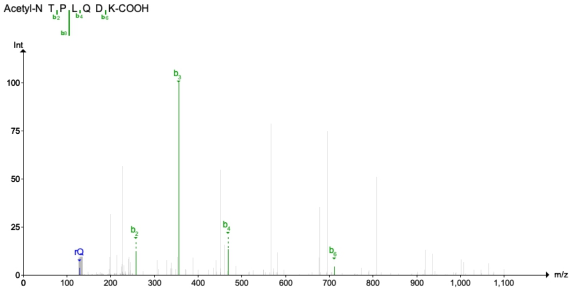
Acetyl-N T P L Q D K-COOH
b2
b3
b4
b6
Int
100
75
50
25
0
b3
b2
b4
b6
rQ
0
100
200
300
400
500
600
700
800
900
1,000
1,100
m/z

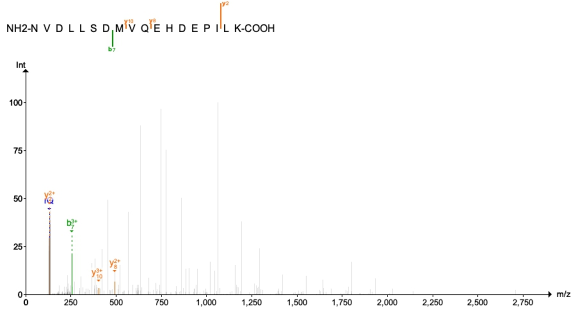

NH2-N V D L L S D M V Q E H D E P I L K-COOH
y2
y10
y8
b7
Int
m/z
0
25
50
75
100
250
500
750
1,000
1,250
1,500
1,750
2,000
2,250
2,500
2,750
y2 2+
b7 3+
y10 3+
y8 2+

NH2-N V I Q E P R P C G G P H T A V G T K-COOH

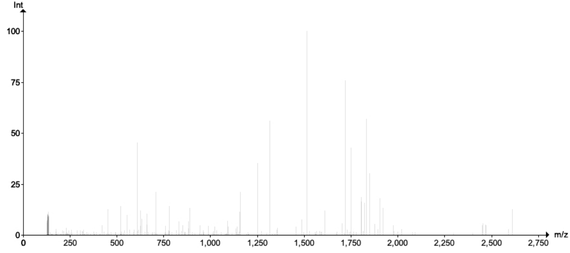

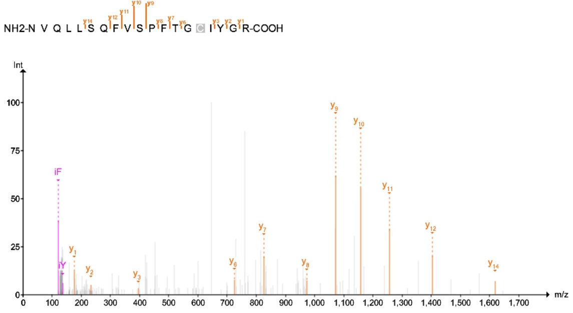
NH2-N V Q L L S Q F V S P F T G C I Y G R-COOH
y14
y12
y11
y10
y9
y8
y7
y6
y3
y2
y1
Int
m/z
iF
iY
y1
y2
y3
y6
y7
y8
y9
y10
y11
y12
y14
0
25
50
75
100
0
100
200
300
400
500
600
700
800
900
1,000
1,100
1,200
1,300
1,400
1,500
1,600
1,700

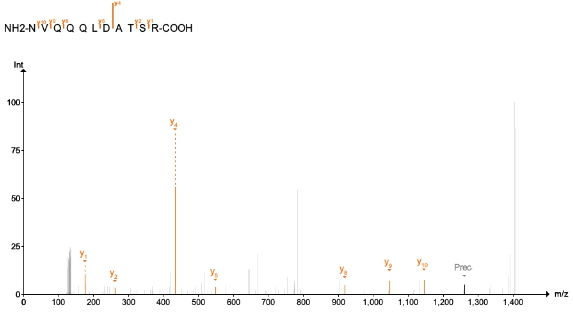

NH2-N V Q Q Q L D A T S R-COOH
y10 y9 y8 y5 y4 y2 y1
Int
100
75
50
25
0
y4
y1
y2
y5
y8
y9
y10
Prec
0 100 200 300 400 500 600 700 800 900 1,000 1,100 1,200 1,300 1,400
m/z

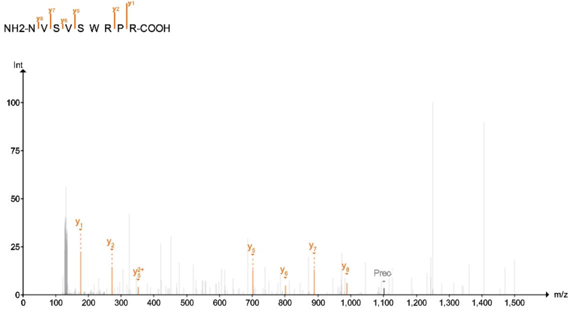

NH2-N V S V S W R P R-COOH
y8
y7
y6
y5
y2
y1
Int
100
75
50
25
0
m/z
100
200
300
400
500
600
700
800
900
1,000
1,100
1,200
1,300
1,400
1,500
Prec

NH2-N Y N K P W E P K-COOH

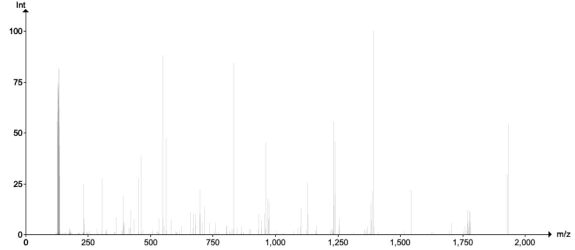

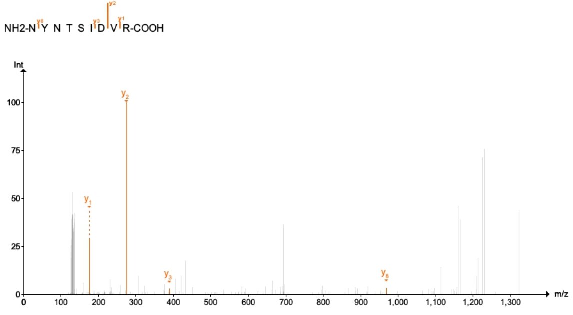
NH2-N Y N T S I D V R-COOH
y8
y3
y2
y1
Int
m/z
y1
y2
y3
y8
0
25
50
75
100
0
100
200
300
400
500
600
700
800
900
1,000
1,100
1,200
1,300

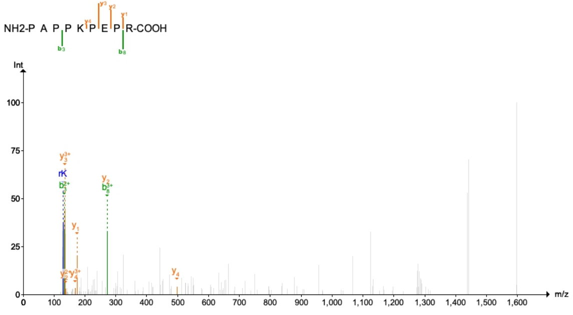

NH2-P A P P K P E P R-COOH
y3
y2
y1
y4
b3
b8
Int
m/z
100
75
50
25
0
0
100
200
300
400
500
600
700
800
900
1,000
1,100
1,200
1,300
1,400
1,500
1,600
rK
y1
y2
y4

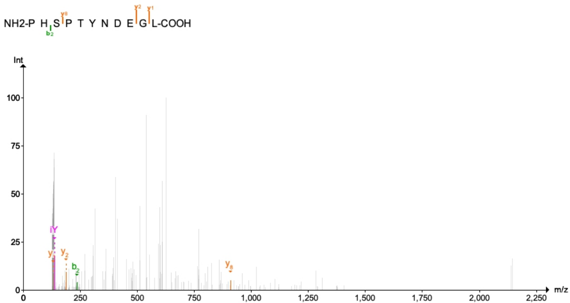

NH2-P H S P T Y N D E G L-COOH
Int
m/z
0
25
50
75
100
0
250
500
750
1,000
1,250
1,500
1,750
2,000
2,250

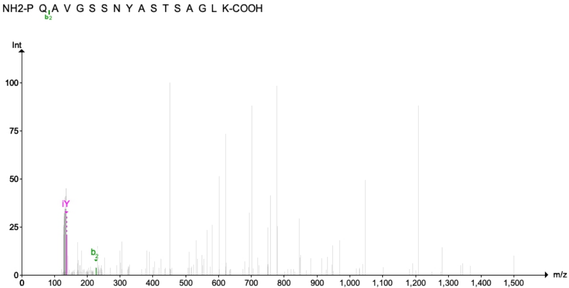
NH2-P Q A V G S S N Y A S T S A G L K-COOH
b2
Int
100
75
50
25
0
IY
b2
0
100
200
300
400
500
600
700
800
900
1,000
1,100
1,200
1,300
1,400
1,500
m/z

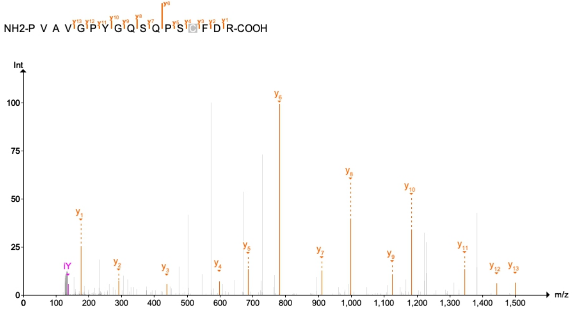

NH2-P V A V G P Y G Q S Q P S C F D R-COOH
y13 y12 y11 y10 y9 y8 y7 y6 y5 y4 y3 y2 y1
Int
100
75
50
25
0
iY
y1
y2
y3
y4
y5
y6
y7
y8
y9
y10
y11
y12
y13
0 100 200 300 400 500 600 700 800 900 1,000 1,100 1,200 1,300 1,400 1,500
m/z

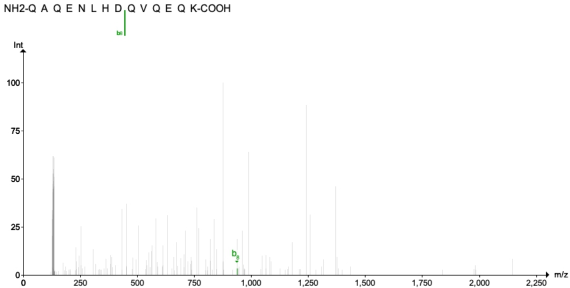
NH2-Q A Q E N L H D Q V Q E Q K-COOH
b8
Int
100
75
50
25
0
b8
0
250
500
750
1,000
1,250
1,500
1,750
2,000
2,250
m/z

NH2-Q C F H E E V E Q G V K-COOH

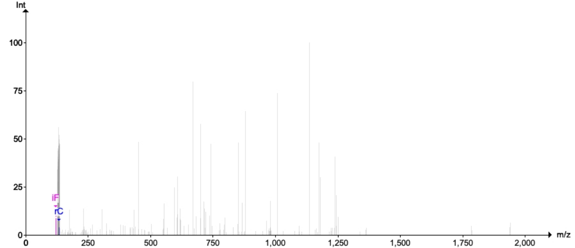

NH2-Q C N H Q A F Q K-COOH

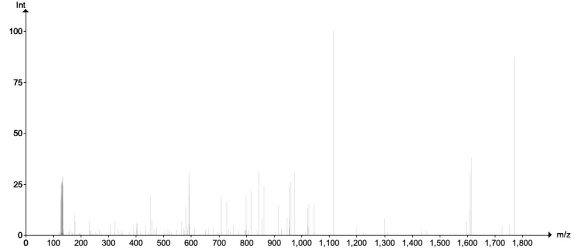

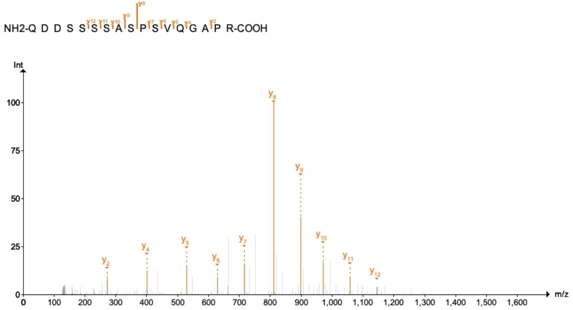

NH2-Q D D S S S S A S P S V Q G A P R-COOH
y12 y11 y10 y9 y8 y7 y6 y5 y4 y2
Int
100
75
50
25
0
y2 y4 y5 y6 y7 y8 y9 y10 y11 y12
0 100 200 300 400 500 600 700 800 900 1,000 1,100 1,200 1,300 1,400 1,500 1,600
m/z

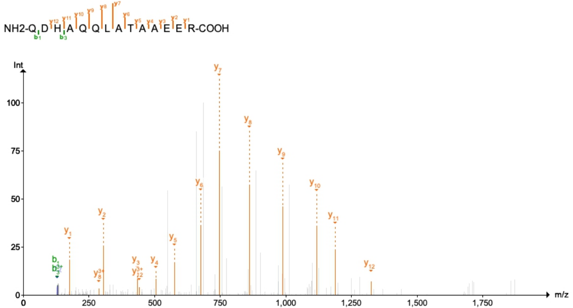
NH2-Q D H A Q Q L A T A A E E R-COOH
Int
m/z
y1
y2
y3
y4
y5
y6
y7
y8
y9
y10
y11
y12
b1
b3
0
25
50
75
100
250
500
750
1,000
1,250
1,500
1,750

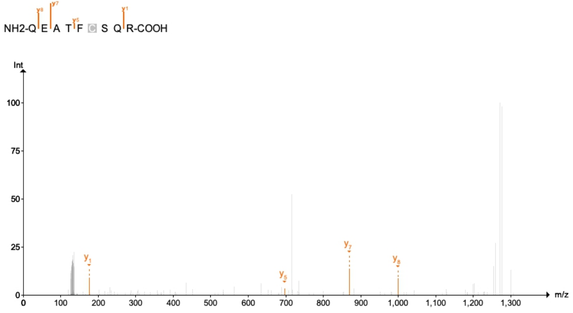

NH2-Q E A T F C S Q R-COOH
y8
y7
y5
y1
Int
100
75
50
25
0
y1
y5
y7
y8
0
100
200
300
400
500
600
700
800
900
1,000
1,100
1,200
1,300
m/z

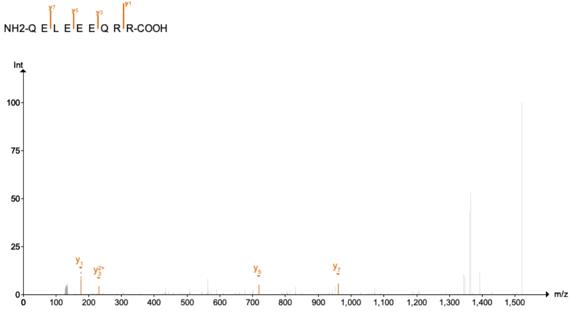

NH2-Q E L E E E Q R R-COOH
y7
y5
y3
y1
Int
100
75
50
25
0
0
100
200
300
400
500
600
700
800
900
1,000
1,100
1,200
1,300
1,400
1,500
m/z
y1
y3 2+
y5
y7

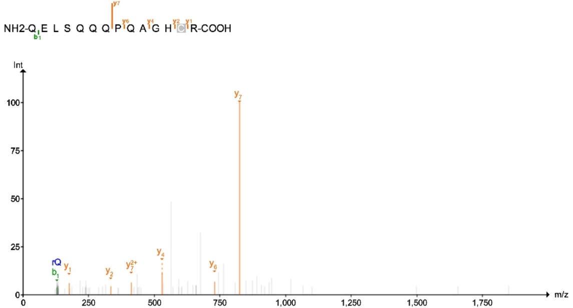

NH2-Q E L S Q Q Q P Q A G H C R-COOH
y7
y6
y4
y2
y1
b1
Int
100
75
50
25
0
rQ
b1
y1
y2
y7 2+
y4
y6
y7
0
250
500
750
1,000
1,250
1,500
1,750
m/z

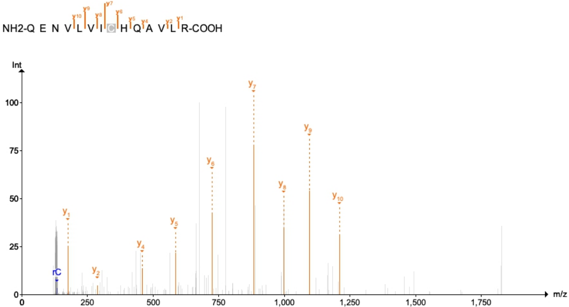
NH2-Q E N V L V I C H Q A V L R-COOH
y10 y9 y8 y7 y6 y5 y4 y2 y1
Int
100
75
50
25
0
y7
y9
y6
y8
y10
y1
y5
y4
y2
rC
0
250
500
750
1,000
1,250
1,500
1,750
m/z

NH2-Q H N E D I L E Y K-COOH

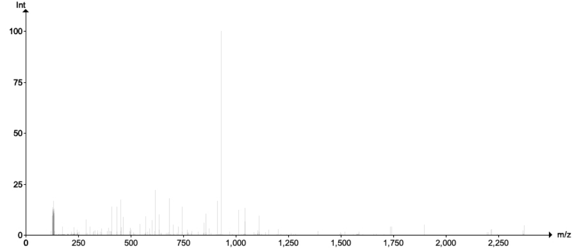

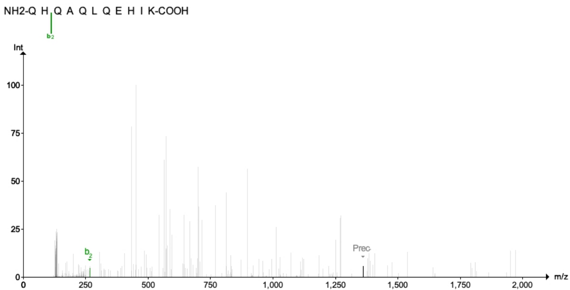
NH2-Q H Q A Q L Q E H I K-COOH
b2
Int
100
75
50
25
0
b2
Prec
0
250
500
750
1,000
1,250
1,500
1,750
2,000
m/z

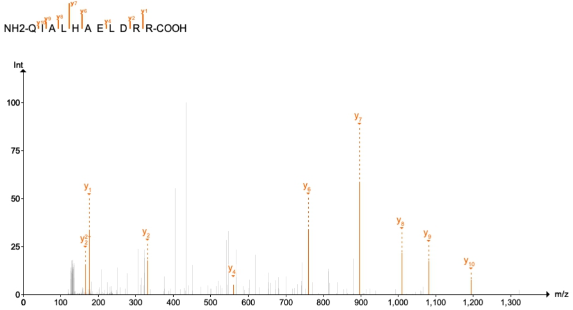
NH2-Q I A L H A E L D R R-COOH
y10 y9 y8 y7 y6 y4 y2 y1
Int
m/z
y1
y2 2+
y2
y4
y6
y7
y8
y9
y10
0
25
50
75
100
0
100
200
300
400
500
600
700
800
900
1,000
1,100
1,200
1,300

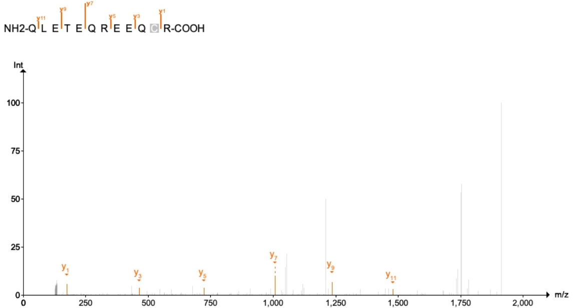

NH2-Q L E T E Q R E E Q C R-COOH
y11
y9
y7
y5
y3
y1
Int
100
75
50
25
0
0
250
500
750
1,000
1,250
1,500
1,750
2,000
m/z

NH2-Q L L D D H K-COOH

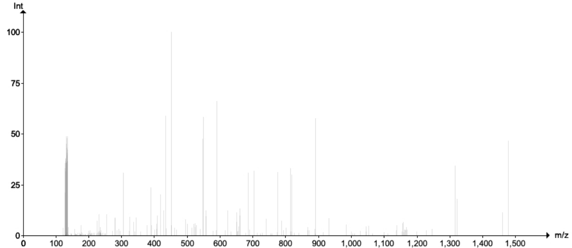

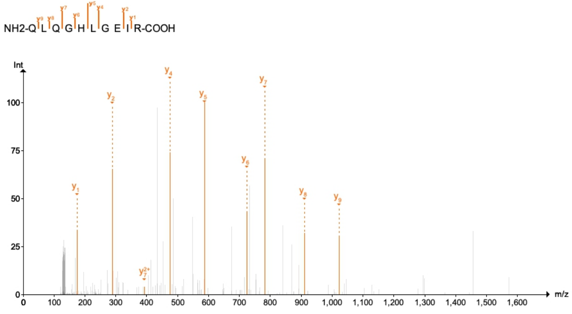
NH2-Q L Q G H L G E I R-COOH
y9 y8 y7 y6 y5 y4 y2 y1
Int
m/z
y1
y2
y7 2+
y4
y5
y6
y7
y8
y9
0
25
50
75
100
0
100
200
300
400
500
600
700
800
900
1,000
1,100
1,200
1,300
1,400
1,500
1,600

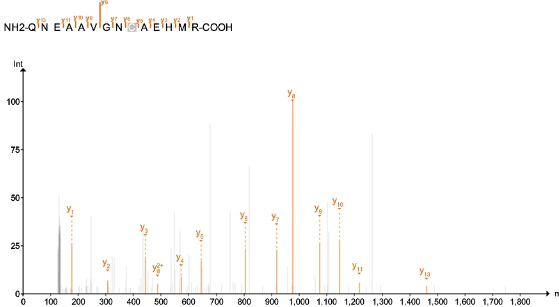
NH2-Q N E A A V G N C A E H M R-COOH
Int
m/z
y1
y2
y3
y4
y5
y6
y7
y8
y9
y10
y11
y13
y8 2+

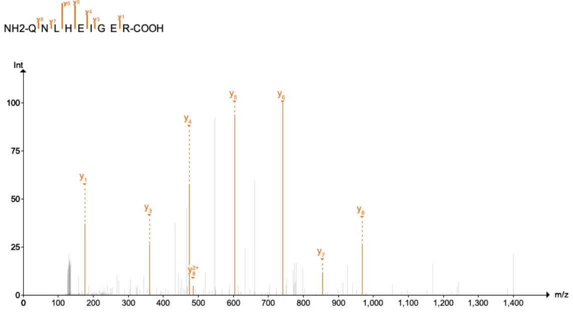

NH2-Q N L H E I G E R-COOH
y8 y7 y6 y5 y4 y3 y1
Int
100
75
50
25
0
y1
y3
y4
y5
y6
y7
y8
y8 2+
0 100 200 300 400 500 600 700 800 900 1,000 1,100 1,200 1,300 1,400
m/z

NH2-Q N P L D E Q H I F Q L P V R P T A V K-COOH

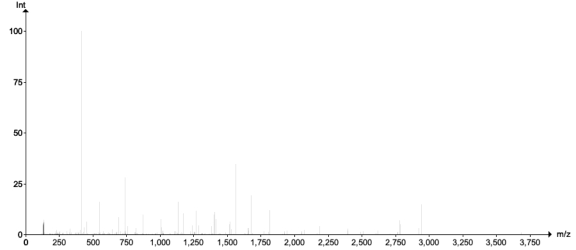

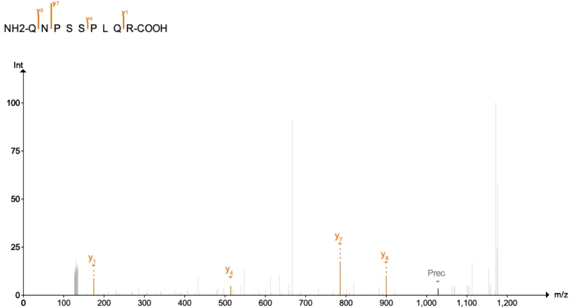

NH2-Q N P S S P L Q R-COOH
y8
y7
y4
y1
Int
100
75
50
25
0
y1
y4
y7
y8
Prec
0
100
200
300
400
500
600
700
800
900
1,000
1,100
1,200
m/z

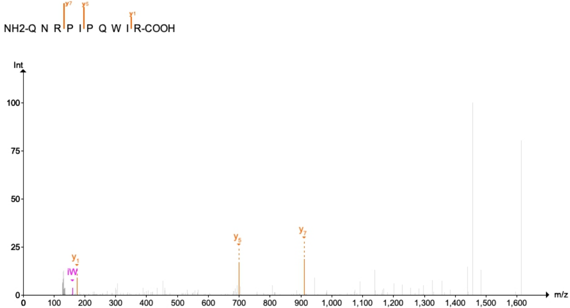

NH2-Q N R P I P Q W I R-COOH
y7
y5
y1
Int
100
75
50
25
0
y5
y7
y1
iW
0 100 200 300 400 500 600 700 800 900 1,000 1,100 1,200 1,300 1,400 1,500 1,600
m/z

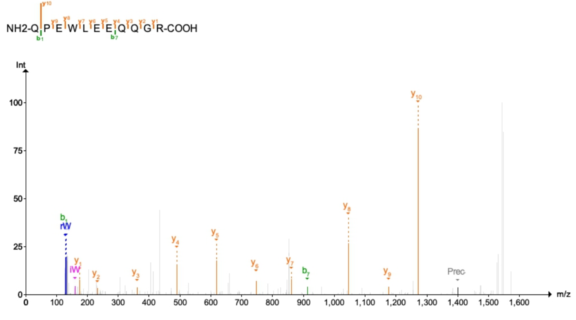

NH2-QPEWLEEQQGR-COOH
Int
m/z
y10
y8
y5
y4
y7
y6
y1
y2
y3
y9
b1
rW
iW
b7
Prec

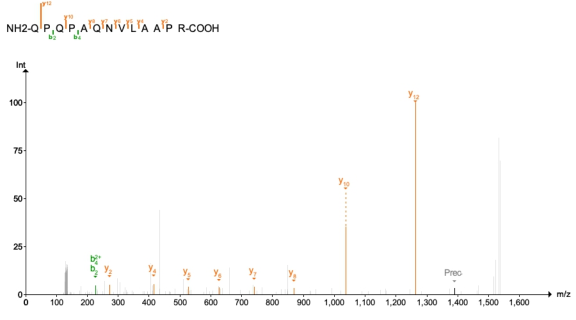
NH2-Q P Q P A Q N V L A A P R-COOH
y12
y10
y8 y7 y6 y5 y4 y2
b2 b4
Int
100
75
50
25
0
y12
y10
b4 2+
b2
y2
y4
y5
y6
y7
y8
Prec
0 100 200 300 400 500 600 700 800 900 1,000 1,100 1,200 1,300 1,400 1,500 1,600
m/z

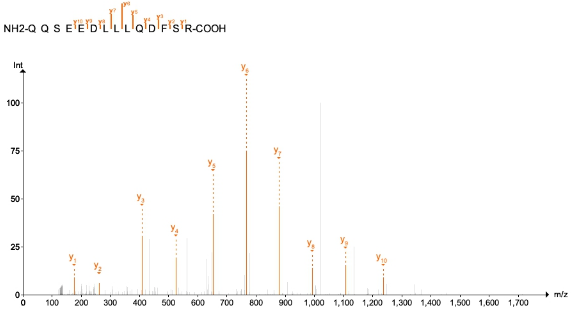

NH2-Q Q S E E D L L L Q D F S R-COOH
y10 y9 y8 y7 y6 y5 y4 y3 y2 y1
Int
100
75
50
25
0
y1
y2
y3
y4
y5
y6
y7
y8
y9
y10
0 100 200 300 400 500 600 700 800 900 1,000 1,100 1,200 1,300 1,400 1,500 1,600 1,700
m/z

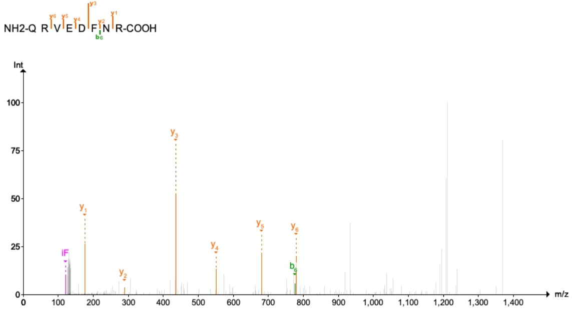
NH2-Q R V E D F N R-COOH
y6 y5 y4 y3 y2 y1 b6
Int
100
75
50
25
0
y1
y2
y3
y4
y5
y6
b6
iF
0 100 200 300 400 500 600 700 800 900 1,000 1,100 1,200 1,300 1,400
m/z

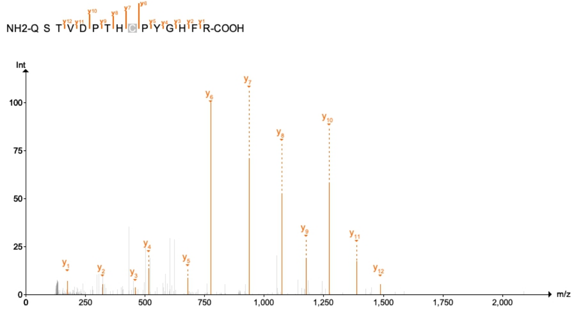
NH2-Q S T V D P T H C P Y G H F R-COOH
Int
m/z
y1
y2
y3
y4
y5
y6
y7
y8
y9
y10
y11
y12
0
25
50
75
100
250
500
750
1,000
1,250
1,500
1,750
2,000

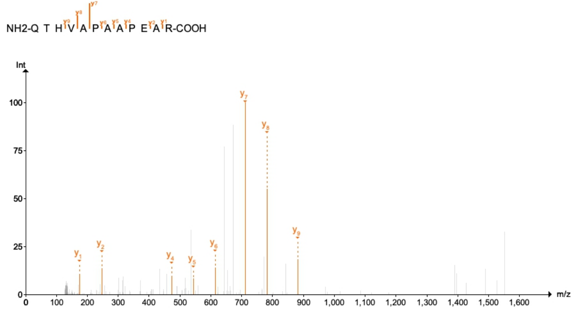
NH2-Q T H V A P A A P E A R-COOH
Int
m/z
y1
y2
y4
y5
y6
y7
y8
y9
0
25
50
75
100
0
100
200
300
400
500
600
700
800
900
1,000
1,100
1,200
1,300
1,400
1,500
1,600

NH2-Q V D Y E I N A H K-COOH

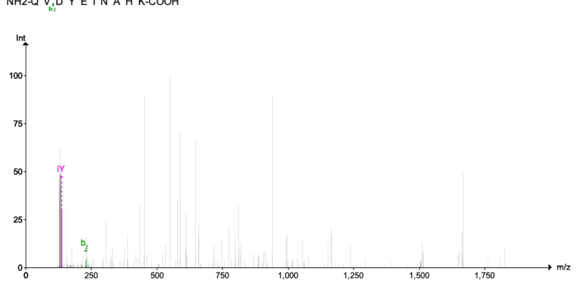

NH2-Q V G V N P T S I D S V V I G K-COOH

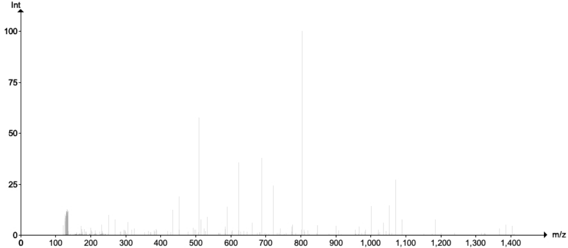

NH2-Q V H I G Q L Y S T D K-COOH

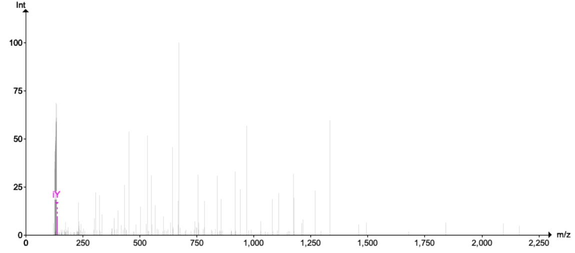

NH2-Q V L E H L L K-COOH

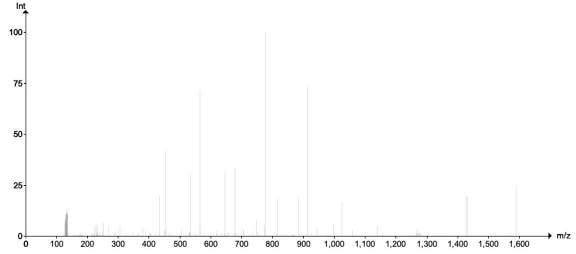

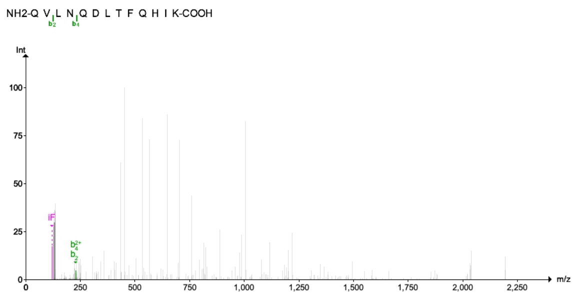

NH2-Q V L N Q D L T F Q H I K-COOH
b2
b4
Int
100
75
50
25
0
iF
b_4^{2+}
b_2
0
250
500
750
1,000
1,250
1,500
1,750
2,000
2,250
m/z

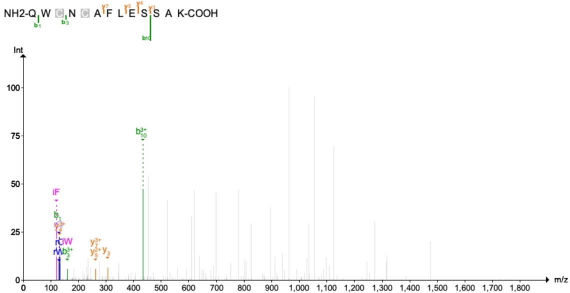
NH2-Q W C N C A F L E S S A K-COOH
b1
b3
b10
y7
y5
y4
y3
Int
100
75
50
25
0
m/z
0
100
200
300
400
500
600
700
800
900
1,000
1,100
1,200
1,300
1,400
1,500
1,600
1,700
1,800
b10 3+
iF
b1
iW
b3 3+
y7 3+
y5 2+
y3

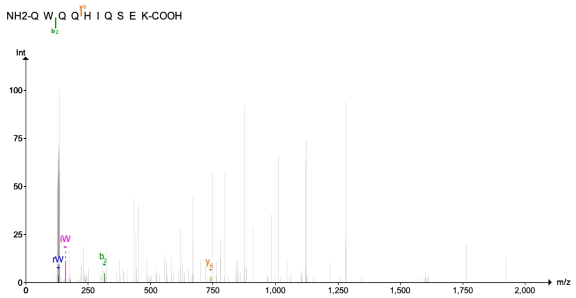
NH2-Q W Q Q H I Q S E K-COOH
y6
b2
Int
100
75
50
25
0
iW
rW
b2
y6
0
250
500
750
1,000
1,250
1,500
1,750
2,000
m/z

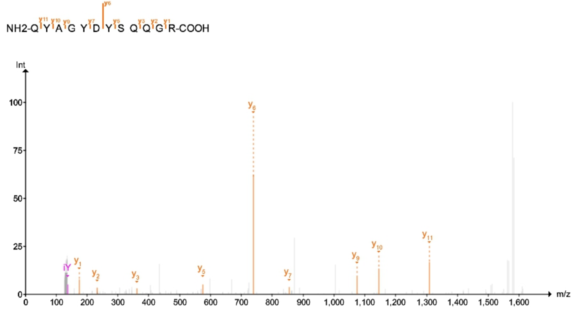

NH2-Q Y A G Y D Y S Q Q G R-COOH
y11 y10 y9 y7 y6 y5 y3 y2 y1
Int
100
75
50
25
0
iY
y1
y2
y3
y5
y6
y7
y9
y10
y11
0 100 200 300 400 500 600 700 800 900 1,000 1,100 1,200 1,300 1,400 1,500 1,600
m/z

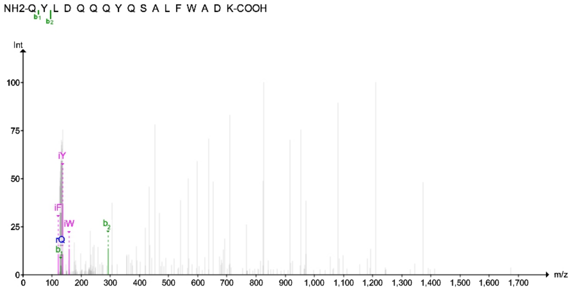

NH2-Q Y L D Q Q Q Y Q S A L F W A D K-COOH
b1
b2
Int
100
75
50
25
0
iY
iF
iW
rQ
b1
b2
0
100
200
300
400
500
600
700
800
900
1,000
1,100
1,200
1,300
1,400
1,500
1,600
1,700
m/z

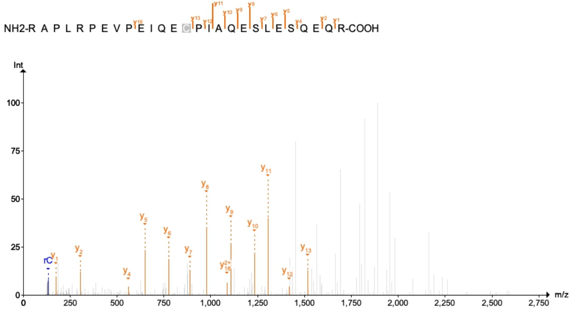
NH2-R A P L R P E V P E I Q E C P I A Q E S L E S Q E Q R-COOH
Int
m/z
rC
y1
y2
y4
y5
y6
y7
y8
y9
y10
y11
y12
y13
y2+ 18
0
25
50
75
100
250
500
750
1,000
1,250
1,500
1,750
2,000
2,250
2,500
2,750

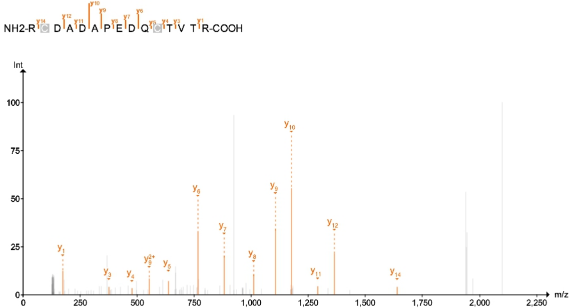

NH2-R C D A D A P E D Q C T V T R-COOH
Int
m/z
y1
y3
y4
y9 2+
y5
y6
y7
y8
y9
y10
y11
y12
y14

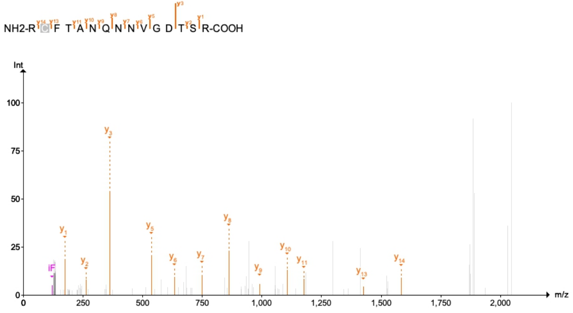

NH2-R C F T A N Q N N V G D T S R-COOH
y14 y13 y11 y10 y9 y8 y7 y6 y5 y3 y2 y1
Int
m/z
y1
y2
y3
y5
y6
y7
y8
y9
y10
y11
y13
y14
iF
0
25
50
75
100
250
500
750
1,000
1,250
1,500
1,750
2,000

NH2-R D P L D E D E D T D I S Y K-COOH

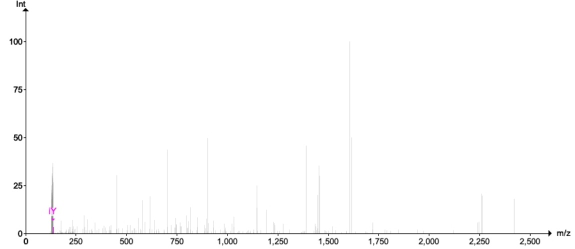

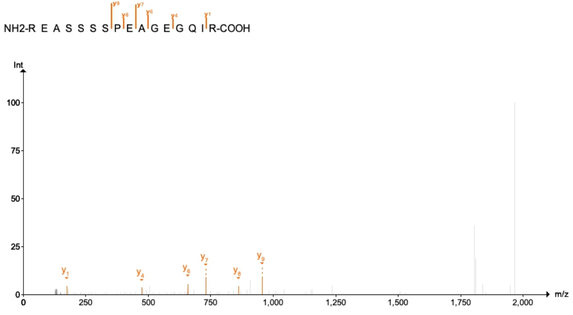

NH2-R E A S S S S P E A G E G Q I R-COOH
y9
y8
y7
y6
y4
y1
Int
100
75
50
25
0
y1
y4
y6
y7
y8
y9
0
250
500
750
1,000
1,250
1,500
1,750
2,000
m/z

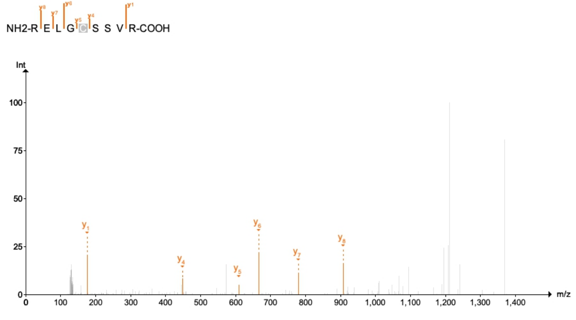

NH2-R E L G C S S V R-COOH
y8
y7
y6
y5
y4
y1
Int
100
75
50
25
0
m/z
y1
y4
y5
y6
y7
y8
0
100
200
300
400
500
600
700
800
900
1,000
1,100
1,200
1,300
1,400

NH2-R E L Q P S E E V T W K-COOH

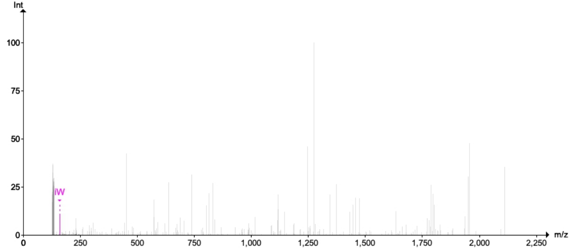

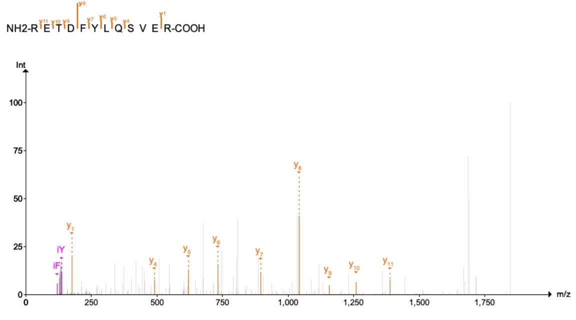
NH2-R E T D F Y L Q S V E R-COOH
y11 y10 y9 y8 y7 y6 y5 y4 y1
Int
100
75
50
25
0
y1
iY
iF
y4
y5
y6
y7
y8
y9
y10
y11
0
250
500
750
1,000
1,250
1,500
1,750
m/z

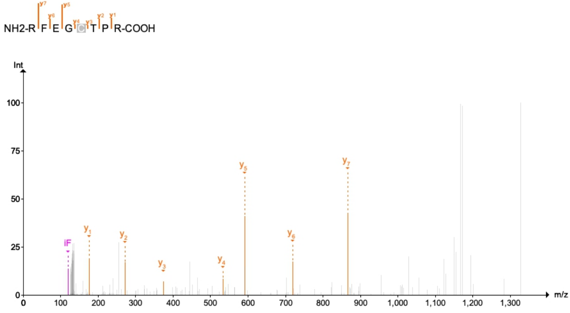

NH2-R F E G C T P R-COOH
y7
y6
y5
y4
y3
y2
y1
Int
100
75
50
25
0
iF
m/z
0
100
200
300
400
500
600
700
800
900
1,000
1,100
1,200
1,300

NH2-R G E I T I Q E G W E G V K-COOH

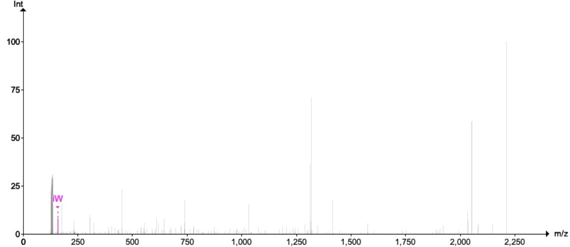

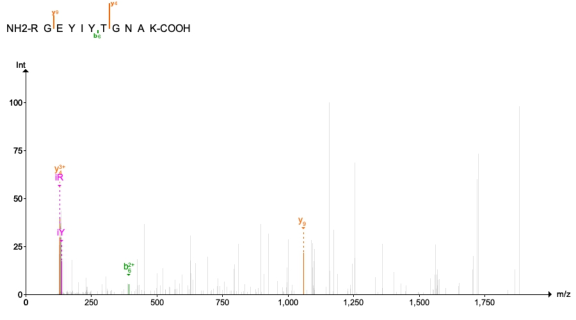
NH2-R G E Y I Y T G N A K-COOH
y9
y4
b6
Int
100
75
50
25
0
y4 3+
iR
iY
b6 2+
y9
0
250
500
750
1,000
1,250
1,500
1,750
m/z

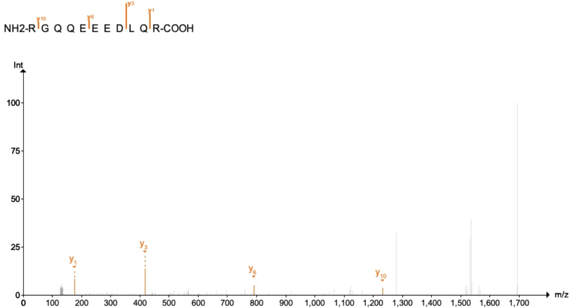

NH2-R G Q Q E E E D L Q R-COOH
y10
y6
y3
y1
Int
m/z
y1
y3
y6
y10

NH2-R G S L P D T Q P S Q G P S T P K-COOH

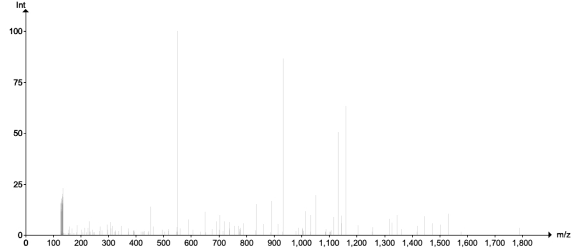

NH2-R H E A F E K-COOH

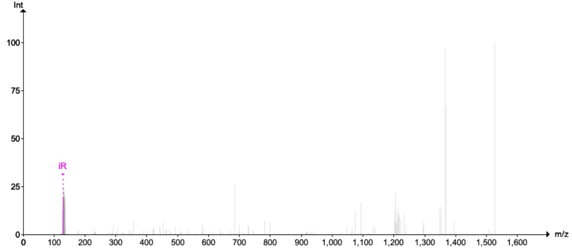

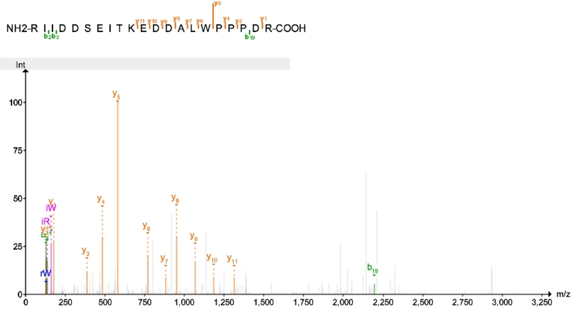

NH2-R I I D D S E I T K E D D A L W P P P D R-COOH
b2 b3
y11 y10 y9 y8 y7 y6 y5 y4 y3 y1
b19
Int
100
75
50
25
0
y5
y4
y8
y6
y9
y3
y7
y10
y11
b19
iW
iR
rW
m/z
0
250
500
750
1,000
1,250
1,500
1,750
2,000
2,250
2,500
2,750
3,000
3,250

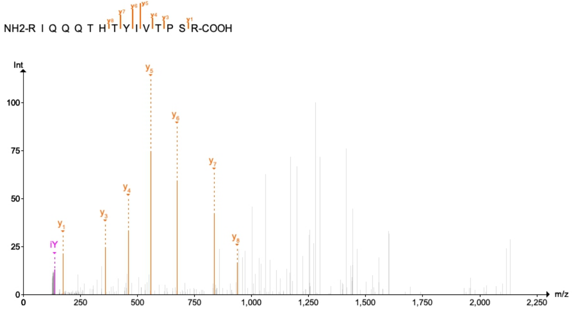
NH2-R I Q Q Q T H T Y I V T P S R-COOH
y8
y7
y6
y5
y4
y3
y1
Int
m/z
iY
y1
y3
y4
y5
y6
y7
y8
0
25
50
75
100
250
500
750
1,000
1,250
1,500
1,750
2,000
2,250

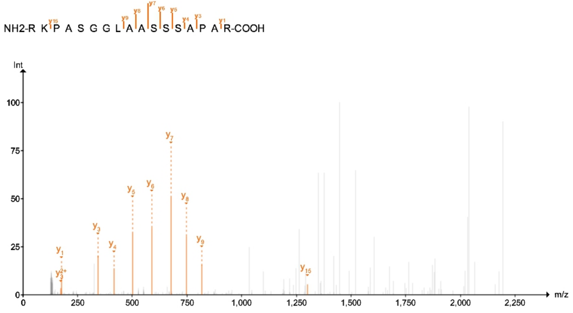
NH2-R K P A S G G L A A S S S A P A R-COOH
y15
y9
y8
y7
y6
y5
y4
y3
y1
Int
100
75
50
25
0
y1
y3 2+
y3
y4
y5
y6
y7
y8
y9
y15
0
250
500
750
1,000
1,250
1,500
1,750
2,000
2,250
m/z

NH2-R L E E E E A A A E K-COOH

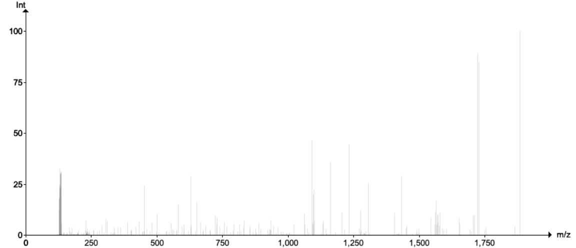

NH2-R L E G L S K-COOH

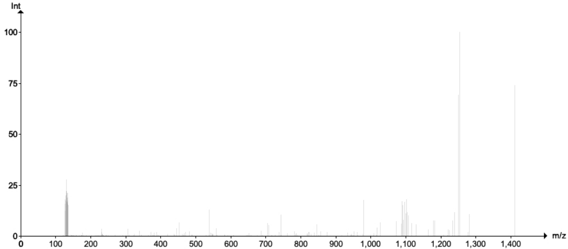

NH2-R L E L P E H L K-COOH

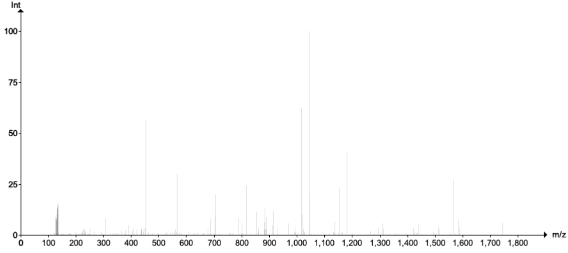

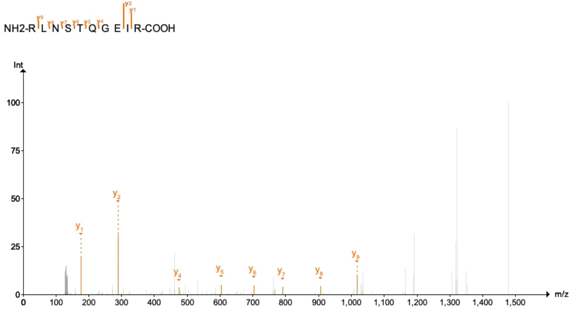

NH2-R L N S T Q G E I R-COOH
y9 y8 y7 y6 y5 y4 y2 y1
Int
100
75
50
25
0
y1
y2
y4
y5
y6
y7
y8
y9
0 100 200 300 400 500 600 700 800 900 1,000 1,100 1,200 1,300 1,400 1,500
m/z

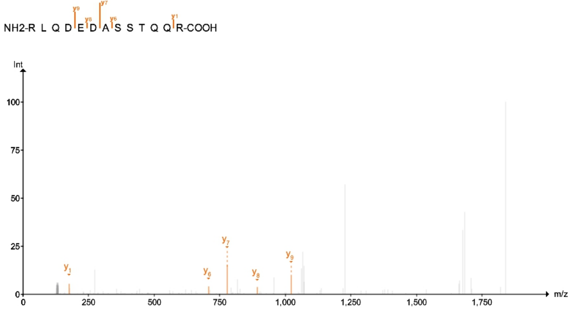

NH2-R L Q D E D A S S T Q Q R-COOH
y9
y8
y7
y6
y1
Int
100
75
50
25
0
y1
y6
y7
y8
y9
0
250
500
750
1,000
1,250
1,500
1,750
m/z

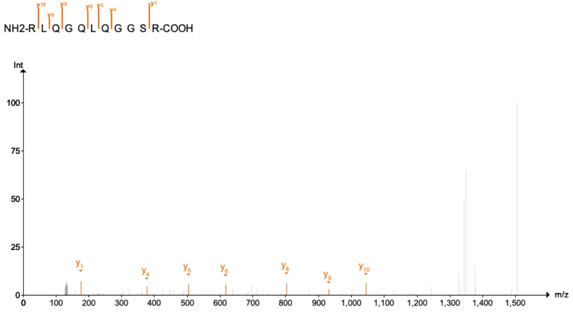

NH2-R L Q G Q L Q G G S R-COOH
y10 y9 y8 y6 y5 y4 y1
Int
100
75
50
25
0
y1 y4 y5 y6 y8 y9 y10
0 100 200 300 400 500 600 700 800 900 1,000 1,100 1,200 1,300 1,400 1,500
m/z

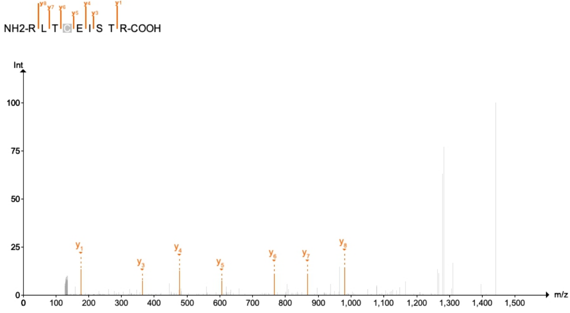

NH2-R L T C E I S T R-COOH
y8 y7 y6 y5 y4 y3 y1
Int
100
75
50
25
0
y1 y3 y4 y5 y6 y7 y8
0 100 200 300 400 500 600 700 800 900 1,000 1,100 1,200 1,300 1,400 1,500
m/z

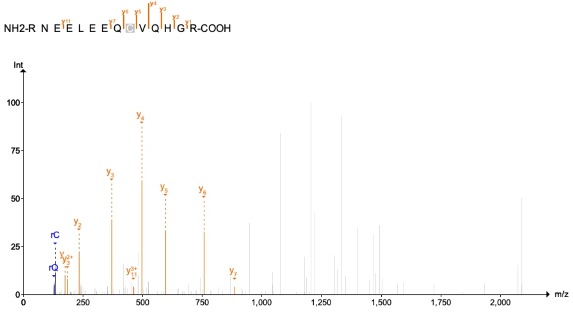

NH2-R N E E L E E Q C V Q H G R-COOH
y11 y7 y6 y5 y4 y3 y2 y1
Int
100
75
50
25
0
y4
y3
y5
y6
y2
rC
rQ
y3 2+
y11 3+
y7
0
250
500
750
1,000
1,250
1,500
1,750
2,000
m/z

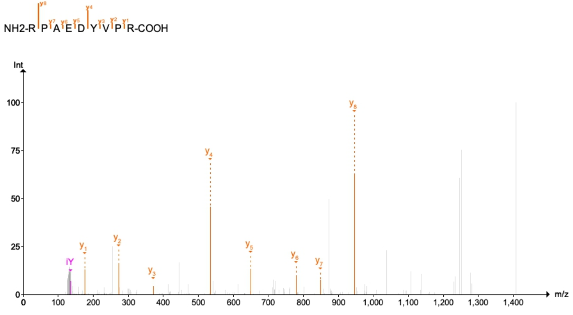
NH2-R P A E D Y V P R-COOH
y8
y7
y6
y5
y4
y3
y2
y1
Int
100
75
50
25
0
iY
y1
y2
y3
y4
y5
y6
y7
y8
0
100
200
300
400
500
600
700
800
900
1,000
1,100
1,200
1,300
1,400
m/z

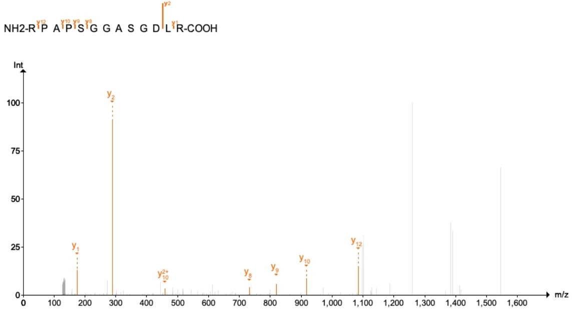
NH2-R P A P S G G A S G D L R-COOH
y12
y10
y9
y8
y2
y1
Int
m/z
y1
y2
y2+10
y8
y9
y10
y12

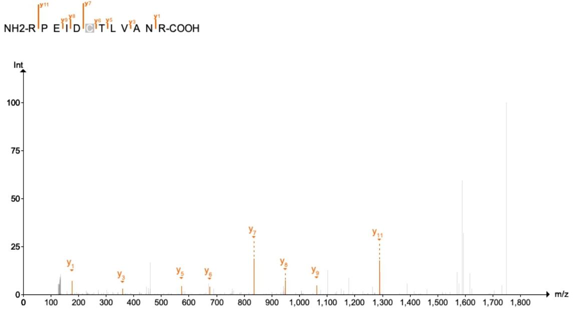
NH2-R P E I D C T L V A N R-COOH
y11
y9
y8
y7
y6
y5
y3
y1
Int
100
75
50
25
0
y1
y3
y5
y6
y7
y8
y9
y11
0
100
200
300
400
500
600
700
800
900
1,000
1,100
1,200
1,300
1,400
1,500
1,600
1,700
1,800
m/z

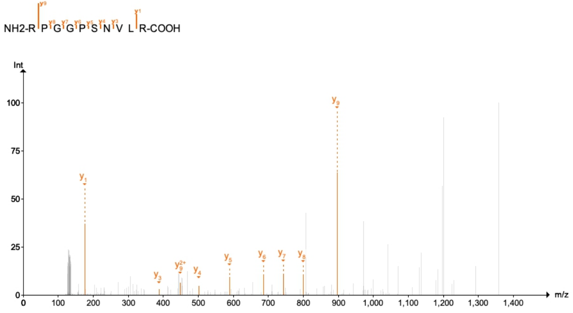
NH2-R P G G P S N V L R-COOH
y9 y8 y7 y6 y5 y4 y3 y1
Int
100
75
50
25
0
y1
y3
y9 2+
y4
y5
y6
y7
y8
y9
0 100 200 300 400 500 600 700 800 900 1,000 1,100 1,200 1,300 1,400
m/z

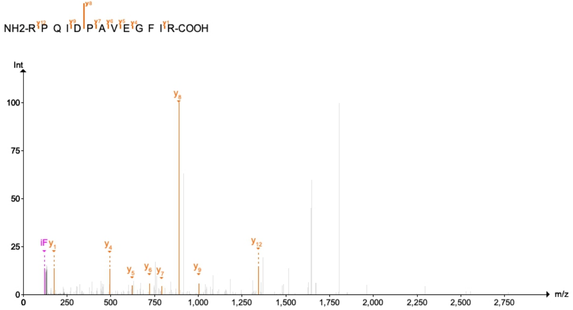
NH2-R P Q I D P A V E G F I R-COOH
y12 y9 y8 y7 y6 y5 y4 y1
Int
100
75
50
25
0
iF
y1
y4
y5
y6
y7
y8
y9
y12
0
250
500
750
1,000
1,250
1,500
1,750
2,000
2,250
2,500
2,750
m/z

NH2-R P T V S S D L E N I D T G V N S K-COOH

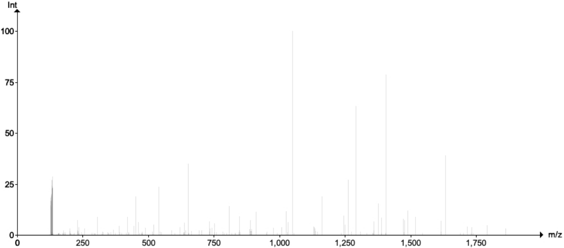

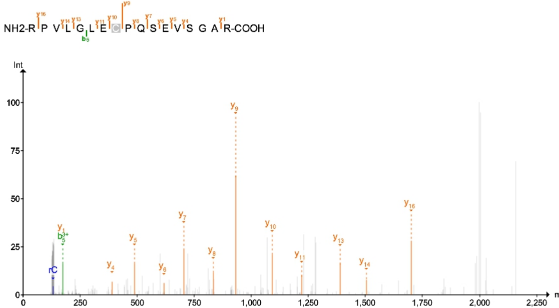

NH2-R P V L G L E C P Q S E V S G A R-COOH
y16
y14
y13
y11
y10
y9
y8
y7
y6
y5
y4
y1
b5
Int
100
75
50
25
0
y9
y16
y7
y10
y13
y1
b5 3+
y5
y8
y11
rC
y4
y6
y14
m/z
0
250
500
750
1,000
1,250
1,500
1,750
2,000
2,250

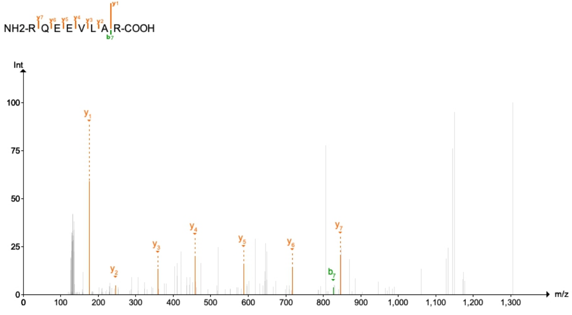
NH2-R Q E E V L A R-COOH
Int
m/z
y1
y2
y3
y4
y5
y6
y7
b7
0
25
50
75
100
0
100
200
300
400
500
600
700
800
900
1,000
1,100
1,200
1,300

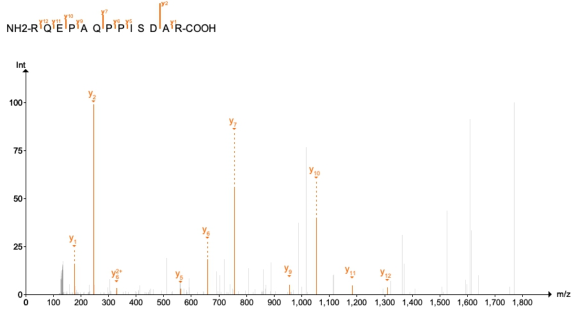

NH2-R Q E P A Q P P I S D A R-COOH
y12 y11 y10 y9 y7 y6 y5 y2 y1
Int
y2
y7
y10
y1
y6
y6 2+
y5
y9
y11
y12
m/z
0 25 50 75 100
0 100 200 300 400 500 600 700 800 900 1,000 1,100 1,200 1,300 1,400 1,500 1,600 1,700 1,800

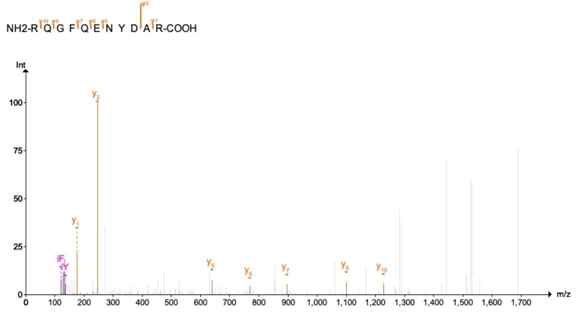

NH2-R Q G F Q E N Y D A R-COOH
y10 y9 y7 y6 y5 y2 y1
Int
100
75
50
25
0
y2
y1
iF
iY
y5
y6
y7
y9
y10
0 100 200 300 400 500 600 700 800 900 1,000 1,100 1,200 1,300 1,400 1,500 1,600 1,700
m/z

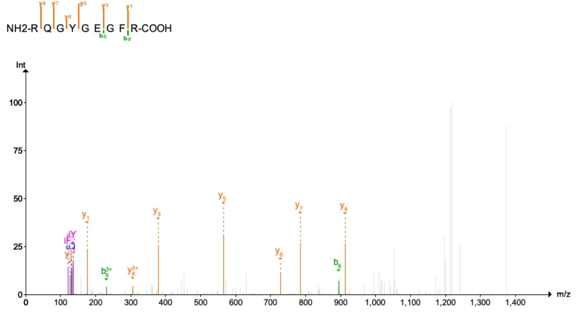

NH2-R Q G Y G E G F R-COOH
Int
m/z
y1
y3
y5
y6
y7
y8
b6
b8

NH2-R Q L Q Q L Q E G T L Q E Y Q K-COOH

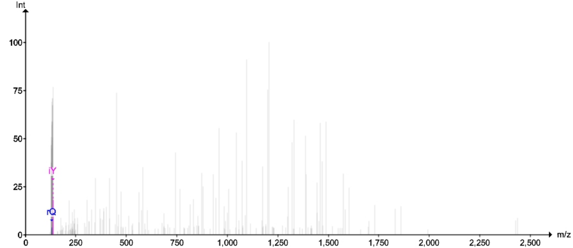

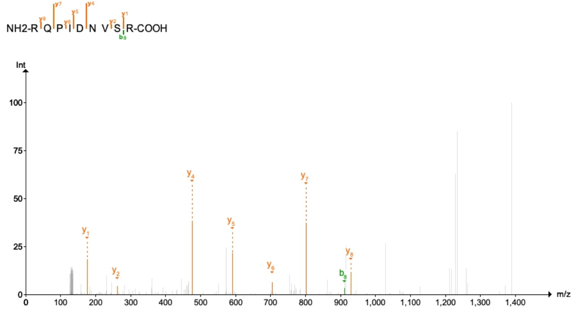

NH2-R Q P I D N V S R-COOH
y8
y7
y6
y5
y4
y2
y1
b8
Int
100
75
50
25
0
y1
y2
y4
y5
y6
y7
b8
y8
m/z
0
100
200
300
400
500
600
700
800
900
1,000
1,100
1,200
1,300
1,400

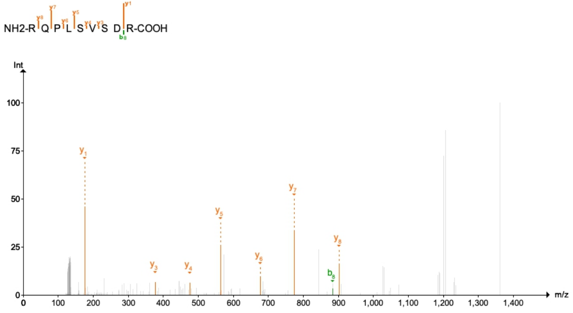

NH2-R Q P L S V S D R-COOH
Int
100
75
50
25
0
y1
y3
y4
y5
y6
y7
y8
b8
0
100
200
300
400
500
600
700
800
900
1,000
1,100
1,200
1,300
1,400
m/z

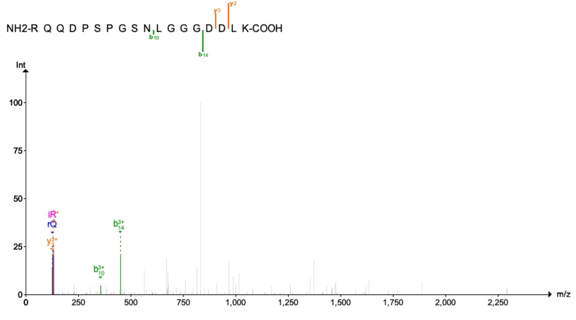
NH2-R Q Q D P S P G S N L G G G D D L K-COOH
b10
b14
y3
y2
Int
100
75
50
25
0
iR+
rQ
y3+
b3+ 14
b3+ 10
0
250
500
750
1,000
1,250
1,500
1,750
2,000
2,250
m/z

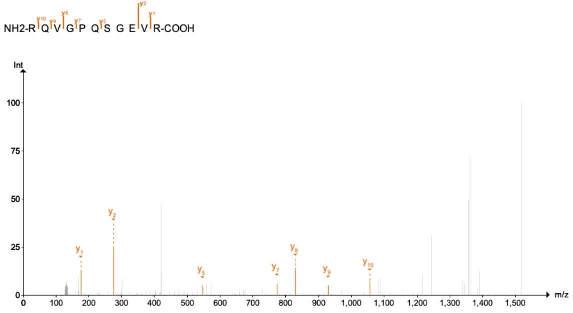

NH2-R Q V G P Q S G E V R-COOH
y10 y9 y8 y7 y5 y2 y1
Int
100
75
50
25
0
y1
y2
y5
y7
y8
y9
y10
0 100 200 300 400 500 600 700 800 900 1,000 1,100 1,200 1,300 1,400 1,500
m/z

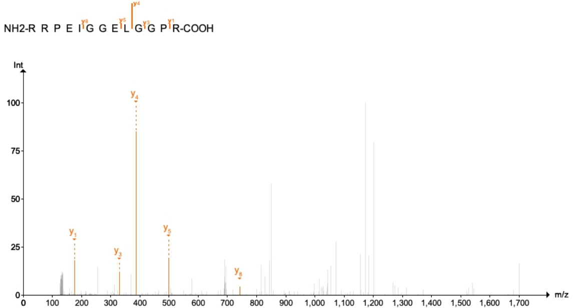

NH2-R R P E I G G E L G G P R-COOH
y8
y5
y4
y3
y1
Int
100
75
50
25
0
y1
y3
y4
y5
y8
m/z
0
100
200
300
400
500
600
700
800
900
1,000
1,100
1,200
1,300
1,400
1,500
1,600
1,700

NH2-R R P E S A P A E S S P S K-COOH

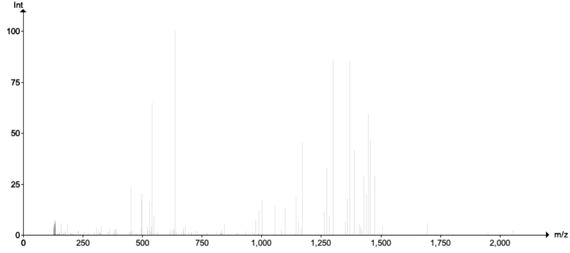

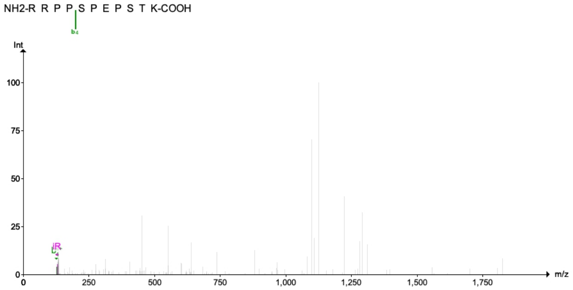
NH2-R R P P S P E P S T K-COOH
b4
Int
100
75
50
25
0
iR
0
250
500
750
1,000
1,250
1,500
1,750
m/z

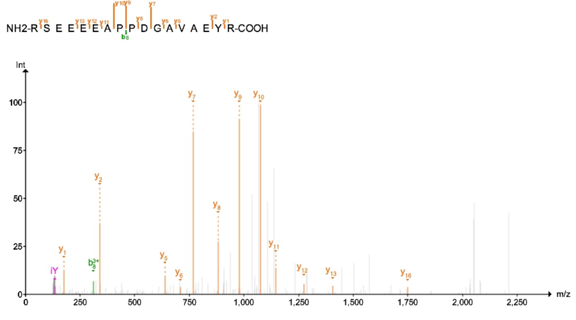

NH2-R S E E E E A P P D G A V A E Y R-COOH
y16 y13 y12 y11 y10 y9 y8 y7 y6 y5 y2 y1
b8
Int
100
75
50
25
0
y7
y9
y10
y2
y8
y1
y5
y6
y11
y12
y13
y16
iY
b_8^{3+}
0
250
500
750
1,000
1,250
1,500
1,750
2,000
2,250
m/z

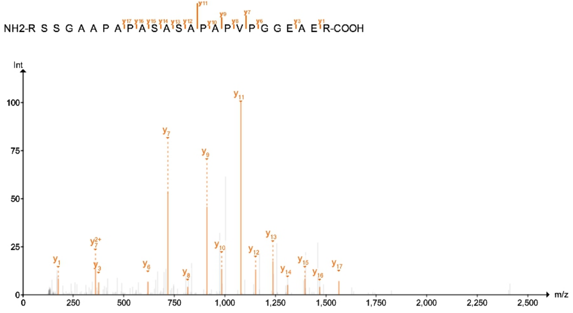

NH2-R S S G A A P A P A S A S A P A P V P G G E A E R-COOH
y17 y16 y15 y14 y13 y12 y11 y10 y9 y8 y7 y6 y3 y1
Int
100
75
50
25
0
y1
y7 2+
y3
y6
y7
y8
y9
y10
y11
y12
y13
y14
y15
y16
y17
0 250 500 750 1,000 1,250 1,500 1,750 2,000 2,250 2,500
m/z

NH2-R T G V A G S Q P V S E K-COOH

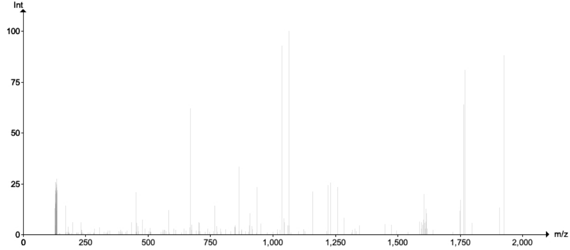

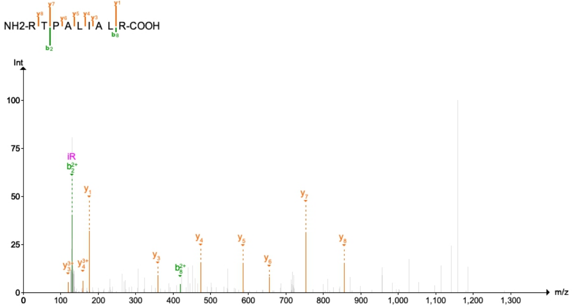

NH2-R T P A L I A L R-COOH
y8 y7 y6 y5 y4 y3 y1
b2 b8
Int
m/z
iR
b2 2+
y1
y3 3+
y4 3+
y3
b8 2+
y4
y5
y6
y7
y8
100
75
50
25
0
0 100 200 300 400 500 600 700 800 900 1,000 1,100 1,200 1,300

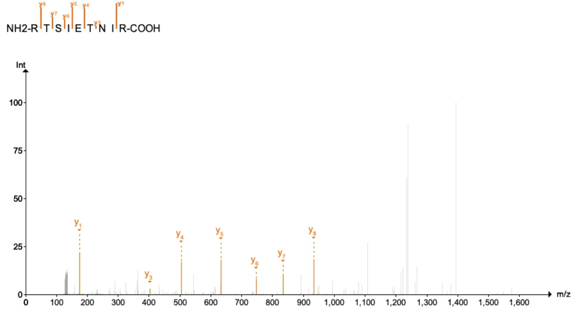
NH2-R T S I E T N I R-COOH
y8
y7
y6
y5
y4
y3
y1
Int
100
75
50
25
0
y1
y3
y4
y5
y6
y7
y8
0
100
200
300
400
500
600
700
800
900
1,000
1,100
1,200
1,300
1,400
1,500
1,600
m/z

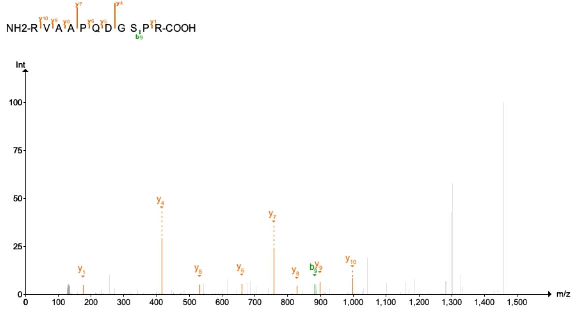
NH2-R V A A P Q D G S P R-COOH
Int
m/z
y1
y4
y5
y6
y7
y8
b9
y9
y10

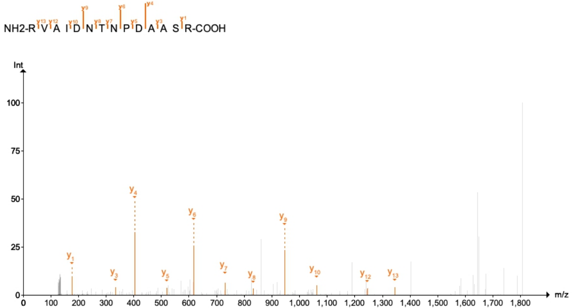
NH2-RVAIDNTNPDAASR-COOH
y13 y12 y10 y9 y8 y7 y6 y5 y4 y3 y1
Int
100
75
50
25
0
y1 y3 y4 y5 y6 y7 y8 y9 y10 y12 y13
0 100 200 300 400 500 600 700 800 900 1,000 1,100 1,200 1,300 1,400 1,500 1,600 1,700 1,800
m/z

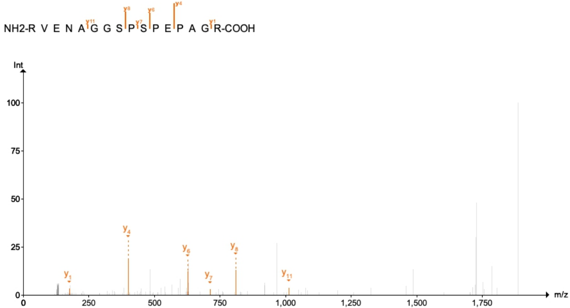
NH2-R V E N A G G S P S P E P A G R-COOH
y11
y8
y7
y6
y4
y1
Int
100
75
50
25
0
y1
y4
y6
y7
y8
y11
0
250
500
750
1,000
1,250
1,500
1,750
m/z

NH2-R V N A I E E V N N N V K-COOH

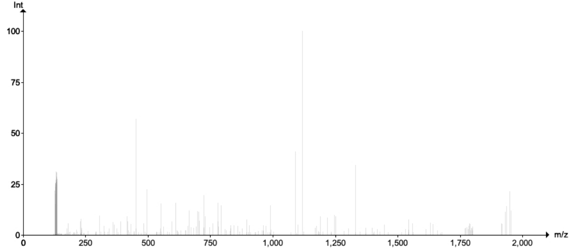

NH2-R V S P V G E T Y I H E G L K-COOH

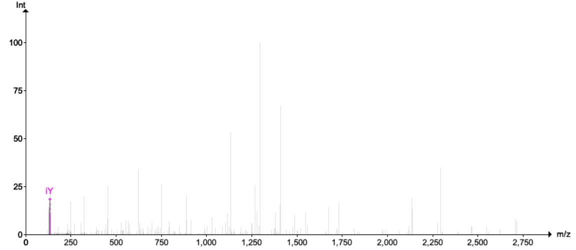

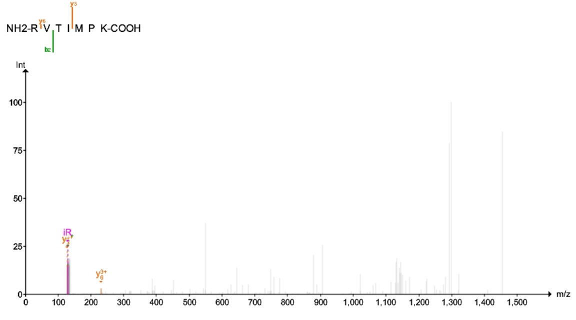

NH2-R V T I M P K-COOH
y6
y3
b2
Int
iR
m/z
100
75
50
25
0
100
200
300
400
500
600
700
800
900
1,000
1,100
1,200
1,300
1,400
1,500

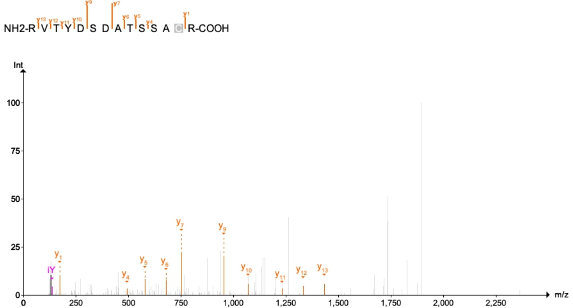

NH2-R V T Y D S D A T S S A C R-COOH
y13
y12
y11
y10
y9
y7
y6
y5
y4
y1
Int
100
75
50
25
0
iY
y1
y4
y5
y6
y7
y9
y10
y11
y12
y13
0
250
500
750
1,000
1,250
1,500
1,750
2,000
2,250
m/z

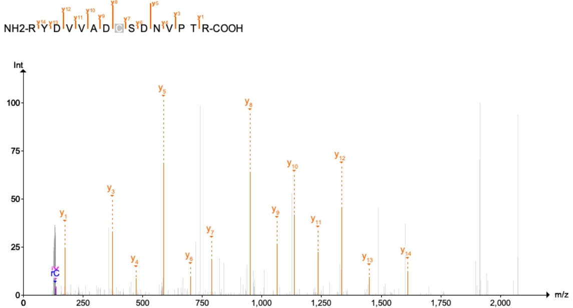

NH2-R Y D V V A D C S D N V P T R-COOH
y14 y13 y12 y11 y10 y9 y8 y7 y6 y5 y4 y3 y1
Int
100
75
50
25
0
y1 y3 y4 y5 y6 y7 y8 y9 y10 y11 y12 y13 y14
rC
0
250
500
750
1,000
1,250
1,500
1,750
2,000
m/z

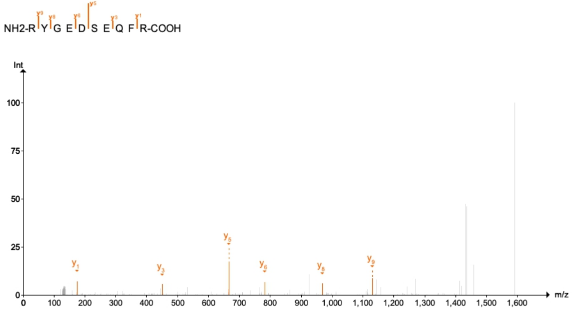

NH2-R Y G E D S E Q F R-COOH
y9 y8 y6 y5 y3 y1
Int
100
75
50
25
0
m/z
0 100 200 300 400 500 600 700 800 900 1,000 1,100 1,200 1,300 1,400 1,500 1,600
y1 y3 y5 y6 y8 y9

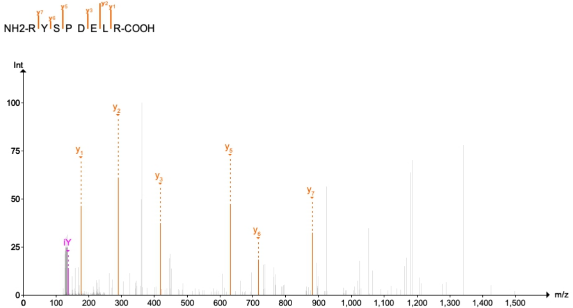

NH2-R Y S P D E L R-COOH
y7 y6 y5 y3 y2 y1
Int
100
75
50
25
0
y1 y2 y3 y5 y6 y7 IY
0 100 200 300 400 500 600 700 800 900 1,000 1,100 1,200 1,300 1,400 1,500
m/z

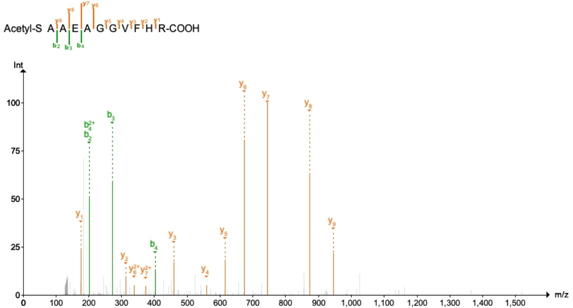

Acetyl-S A A E A G G V F H R-COOH
y9 y8 y7 y6 y5 y4 y3 y2 y1
b2 b3 b4
Int
100
75
50
25
0
y1
b4 2+
b2
b3
y2
y6 2+
y7 2+
b4
y3
y4
y5
y6
y7
y8
y9
m/z
0 100 200 300 400 500 600 700 800 900 1,000 1,100 1,200 1,300 1,400 1,500

NH2-S A A L Q S T Q S Q E E F K L E D L K-COOH

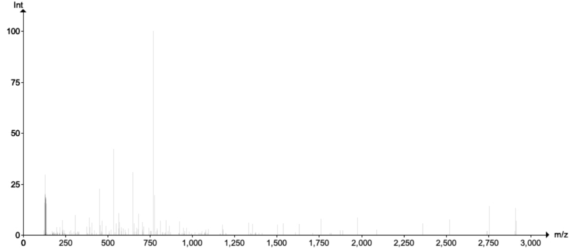

NH2-S A D E G Q T V F Y T C T N C K-COOH

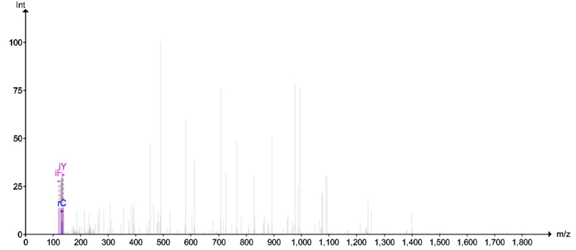

NH2-S A E S M S D G V G S F L P G K-COOH

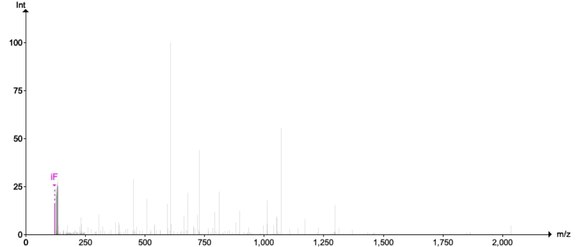

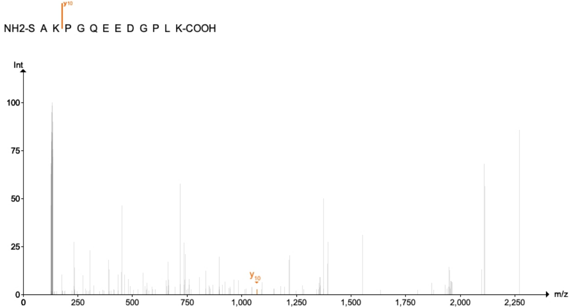

y10
NH2-S A K P G Q E E D G P L K-COOH
Int
100
75
50
25
0
0
250
500
750
1,000
1,250
1,500
1,750
2,000
2,250
m/z
y10

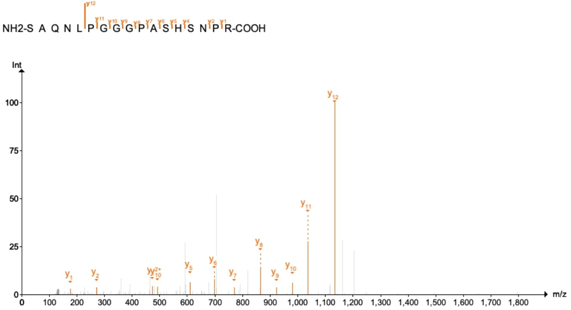

NH2-S A Q N L P G G G P A S H S N P R-COOH
y12 y11 y10 y9 y8 y7 y6 y5 y4 y2 y1
Int
100
75
50
25
0
y1 y2 y y2+ 10 y5 y6 y7 y8 y9 y10 y11 y12
0 100 200 300 400 500 600 700 800 900 1,000 1,100 1,200 1,300 1,400 1,500 1,600 1,700 1,800
m/z

NH2-S C D L A L L E T Y C A T P A K-COOH

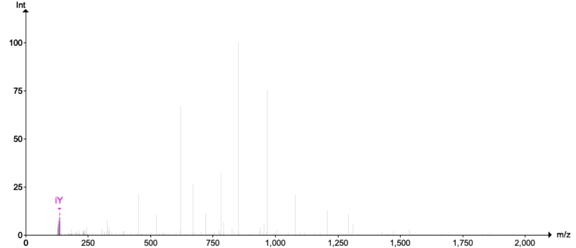

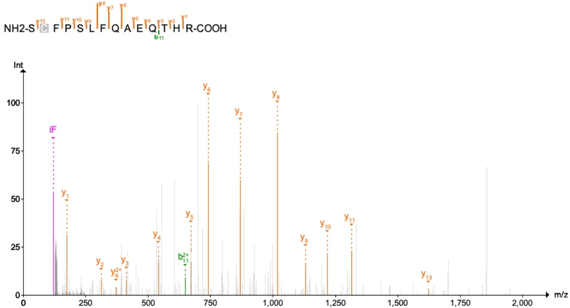
NH2-S C F P S L F Q A E Q T H R-COOH
Int
iF
y1
y2
y3
y4
y5
y6
y7
y8
y9
y10
y11
y13
y_6^{2+}
b_{11}^{2+}
b11
m/z
0
25
50
75
100
250
500
750
1,000
1,250
1,500
1,750
2,000

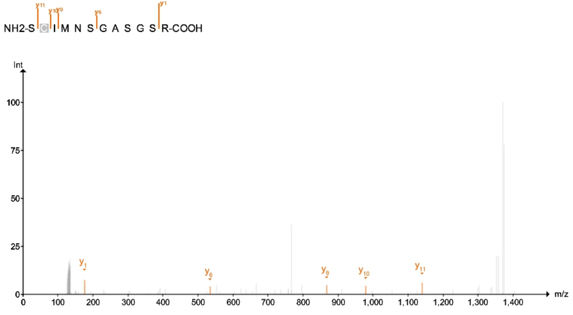

NH2-S C I M N S G A S G S R-COOH
y11
y10
y9
y6
y1
Int
100
75
50
25
0
y1
y6
y9
y10
y11
0
100
200
300
400
500
600
700
800
900
1,000
1,100
1,200
1,300
1,400
m/z

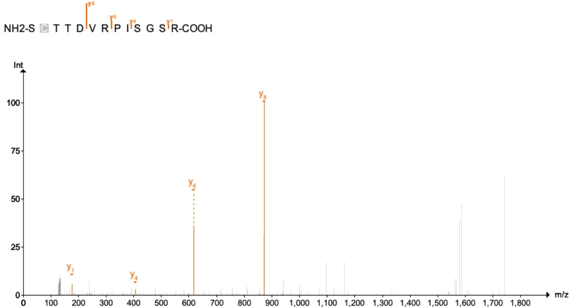
NH2-S C T T D V R P I S G S R-COOH
y8
y6
y4
y1
Int
100
75
50
25
0
m/z
0
100
200
300
400
500
600
700
800
900
1,000
1,100
1,200
1,300
1,400
1,500
1,600
1,700
1,800

NH2-S C V P L N T N E L N S N E N I H Y K-COOH

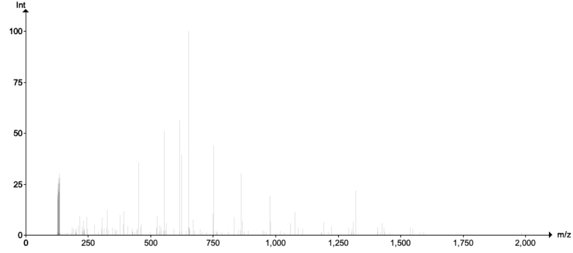

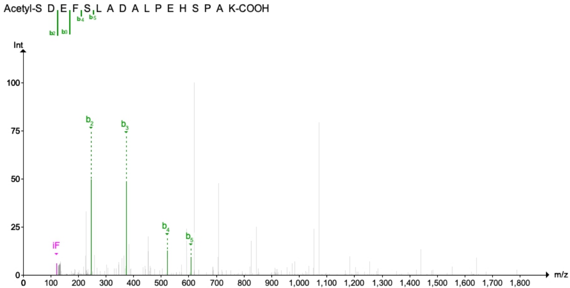

Acetyl-S D E F S L A D A L P E H S P A K-COOH
b2 b3 b4 b5
Int
100
75
50
25
0
b2
b3
b4
b5
iF
0 100 200 300 400 500 600 700 800 900 1,000 1,100 1,200 1,300 1,400 1,500 1,600 1,700 1,800
m/z

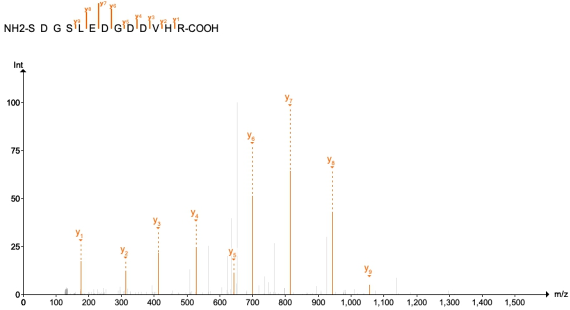
NH2-S D G S L E D G D D V H R-COOH
y9 y8 y7 y6 y5 y4 y3 y2 y1
Int
100
75
50
25
0
y1
y2
y3
y4
y5
y6
y7
y8
y9
0 100 200 300 400 500 600 700 800 900 1,000 1,100 1,200 1,300 1,400 1,500
m/z

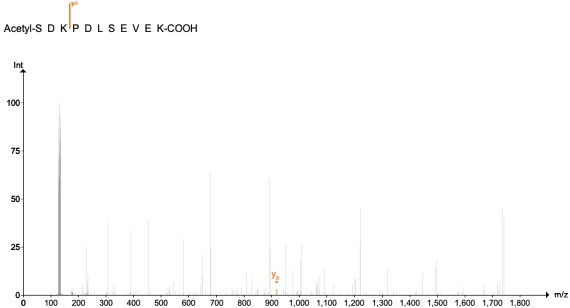

y8
Acetyl-S D K P D L S E V E K-COOH
Int
100
75
50
25
0
y8
0
100
200
300
400
500
600
700
800
900
1,000
1,100
1,200
1,300
1,400
1,500
1,600
1,700
1,800
m/z

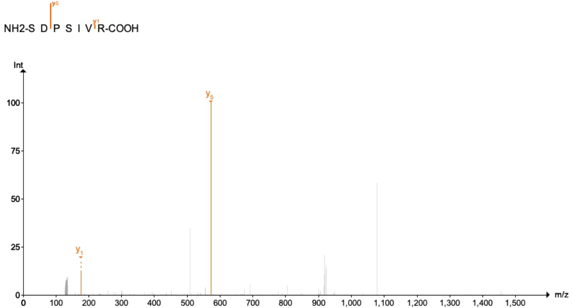

NH2-S D P S I V R-COOH
y5
y1
Int
m/z
y5
y1
0
25
50
75
100
0
100
200
300
400
500
600
700
800
900
1,000
1,100
1,200
1,300
1,400
1,500

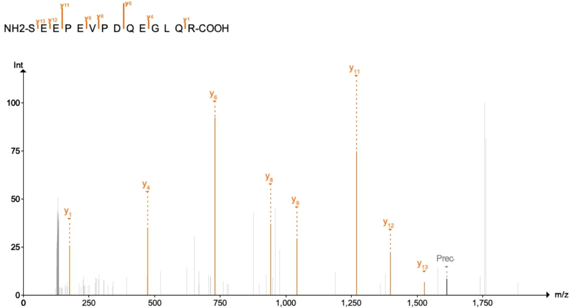

NH2-S E E P E V P D Q E G L Q R-COOH
y13
y12
y11
y9
y8
y6
y4
y1
Int
100
75
50
25
0
y1
y4
y6
y8
y9
y11
y12
y13
Prec
250
500
750
1,000
1,250
1,500
1,750
m/z

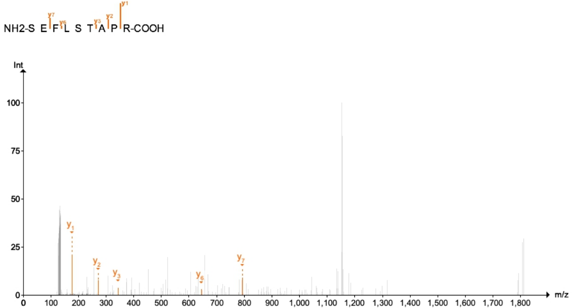

NH2-S E F L S T A P R-COOH
y7
y6
y3
y2
y1
Int
100
75
50
25
0
m/z
0
100
200
300
400
500
600
700
800
900
1,000
1,100
1,200
1,300
1,400
1,500
1,600
1,700
1,800
y1
y2
y3
y6
y7

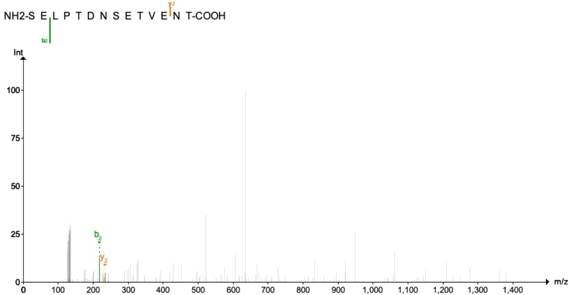

NH2-S E L P T D N S E T V E N T-COOH
b2
y2
Int
100
75
50
25
0
0 100 200 300 400 500 600 700 800 900 1,000 1,100 1,200 1,300 1,400
m/z

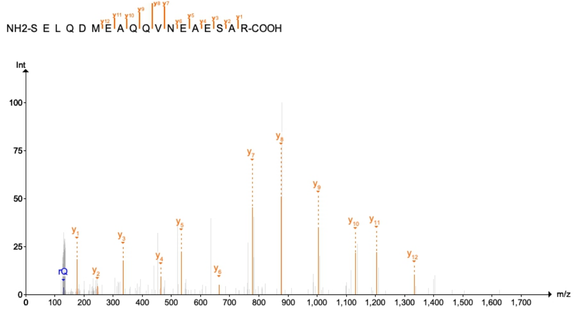

NH2-S E L Q D M E A Q Q V N E A E S A R-COOH
Int
m/z
y1
y2
y3
y4
y5
y6
y7
y8
y9
y10
y11
y12
rQ

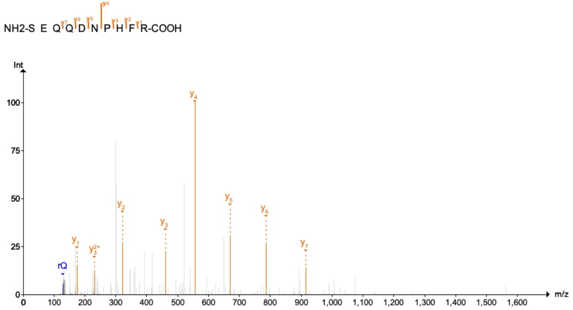
NH2-S E Q Q D N P H F R-COOH
y7 y6 y5 y4 y3 y2 y1
Int
100
75
50
25
0
y4
y2
y5
y6
y3
y1
y3 2+
y7
rQ
0 100 200 300 400 500 600 700 800 900 1,000 1,100 1,200 1,300 1,400 1,500 1,600
m/z

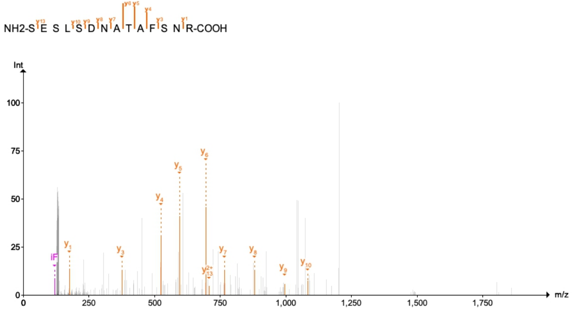

NH2-S E S L S D N A T A F S N R-COOH
y13 y10 y9 y8 y7 y6 y5 y4 y3 y1
Int
100
75
50
25
0
iF
y1
y3
y4
y5
y6
y2+13
y7
y8
y9
y10
0
250
500
750
1,000
1,250
1,500
1,750
m/z

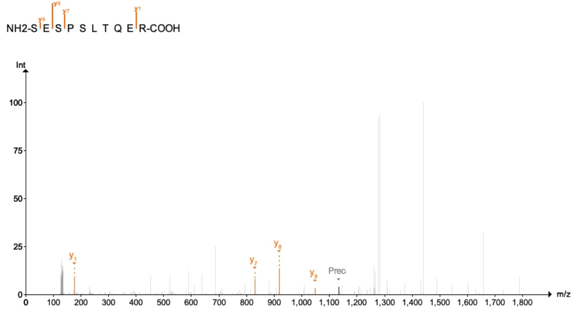

NH2-S E S P S L T Q E R-COOH
y9
y8
y7
y1
Int
100
75
50
25
0
y1
y7
y8
y9
Prec
0
100
200
300
400
500
600
700
800
900
1,000
1,100
1,200
1,300
1,400
1,500
1,600
1,700
1,800
m/z

NH2-S E T I S V P Q E I K-COOH

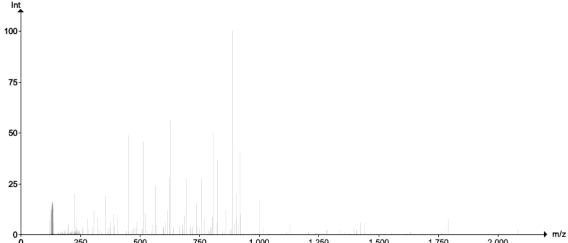

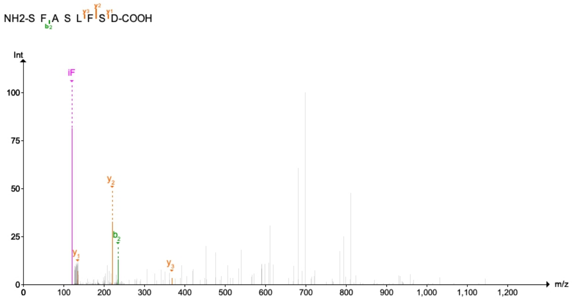

NH2-S F A S L F S D-COOH
b2
y3
y2
y1
Int
iF
y2
b2
y1
y3
100
75
50
25
0
0
100
200
300
400
500
600
700
800
900
1,000
1,100
1,200
m/z

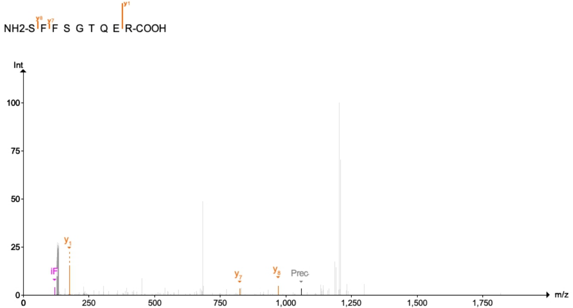

y8
y7
y1
NH2-S F F S G T Q E R-COOH
Int
100
75
50
25
0
y1
iF
y7
y8
Prec
0
250
500
750
1,000
1,250
1,500
1,750
m/z

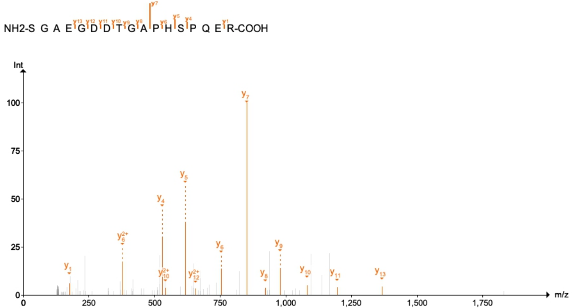

NH2-S G A E G D D T G A P H S P Q E R-COOH
y13 y12 y11 y10 y9 y8 y7 y6 y5 y4 y1
Int
100
75
50
25
0
y7
y5
y4
y6 2+
y6
y9
y1
y10 2+
y12 2+
y8
y10
y11
y13
0
250
500
750
1,000
1,250
1,500
1,750
m/z

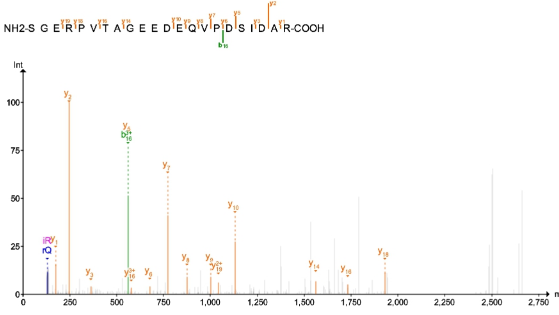

NH2-S G E R P V T A G E E D E Q V P D S I D A R-COOH
Int
m/z
y2
y5
b16 3+
y7
y10
iR
rQ
y1
y3
y16 3+
y6
y8
y9
y19 2+
y14
y16
y18

NH2-S G E S Q V L F E T E I S R K-COOH

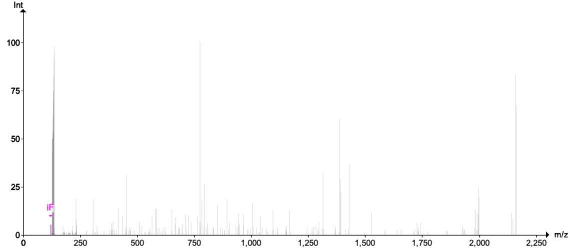

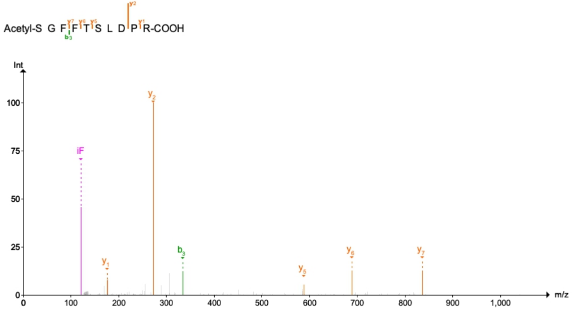
Acetyl-S G F F T S L D P R-COOH
y7 y6 y5 y2 y1
b3
Int
100
75
50
25
0
iF
y2
y1
b3
y5
y6
y7
0
100
200
300
400
500
600
700
800
900
1,000
m/z

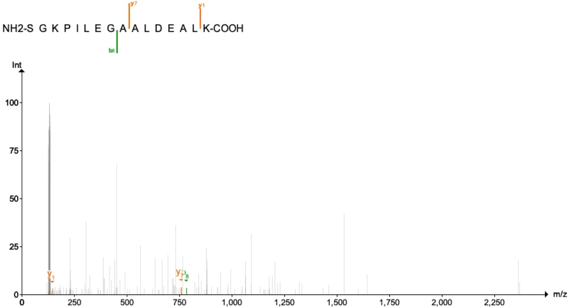

NH2-S G K P I L E G A A L D E A L K-COOH
y7
y1
b8
Int
100
75
50
25
0
y1
y7
b8
0
250
500
750
1,000
1,250
1,500
1,750
2,000
2,250
m/z

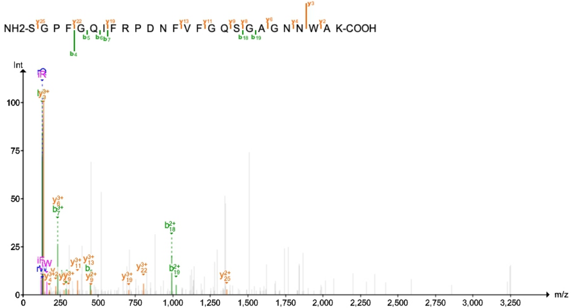
NH2-S G P F G Q I F R P D N F V F G Q S G A G N N W A K-COOH
Int
m/z
0
25
50
75
100
250
500
750
1,000
1,250
1,500
1,750
2,000
2,250
2,500
2,750
3,000
3,250

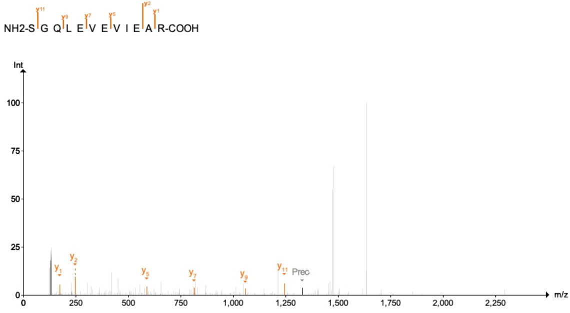

NH2-S G Q L E V E V I E A R-COOH
y11
y9
y7
y5
y2
y1
Int
100
75
50
25
0
m/z
0
250
500
750
1,000
1,250
1,500
1,750
2,000
2,250
Prec

NH2-S G T G I A A M S V M R P E Q I M K-COOH

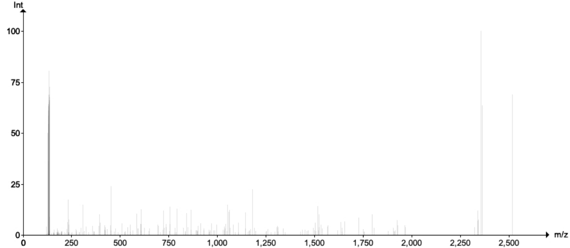

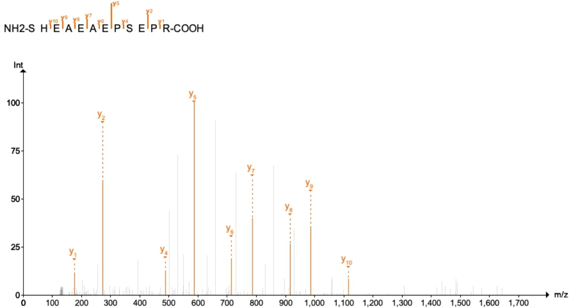

NH2-S H E A E A E P S E P R-COOH
y10 y9 y8 y7 y6 y5 y4 y2 y1
Int
100
75
50
25
0
y1 y2 y4 y5 y6 y7 y8 y9 y10
0 100 200 300 400 500 600 700 800 900 1,000 1,100 1,200 1,300 1,400 1,500 1,600 1,700
m/z

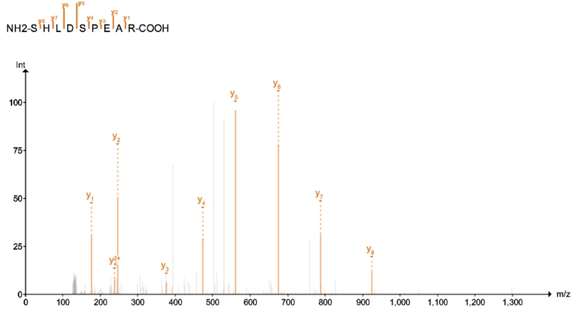
NH2-S H L D S P E A R-COOH
Int
m/z
y1
y2
y3
y4
y4 2+
y5
y6
y7
y8

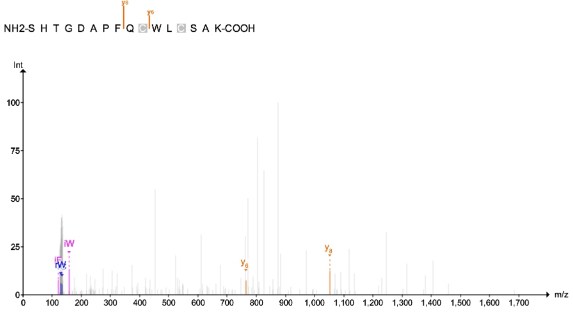

NH2-S H T G D A P F Q C W L C S A K-COOH
y8
y6
Int
100
75
50
25
0
iW
iF
rW
y6
y8
0 100 200 300 400 500 600 700 800 900 1,000 1,100 1,200 1,300 1,400 1,500 1,600 1,700
m/z

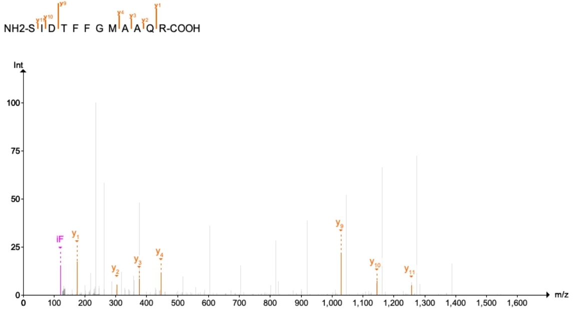
NH2-S I D T F F G M A A Q R-COOH
y11 y10 y9 y4 y3 y2 y1
Int
m/z
iF
y1
y2
y3
y4
y9
y10
y11
0
25
50
75
100
0 100 200 300 400 500 600 700 800 900 1,000 1,100 1,200 1,300 1,400 1,500 1,600

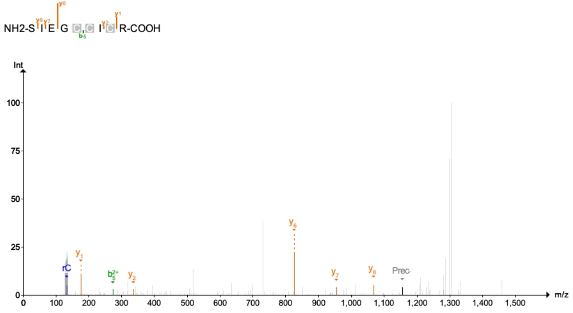

NH2-S I E G C C I C R-COOH
y8
y7
y6
y2
y1
b5
Int
m/z
rC
y1
b5 2+
y2
y6
y7
y8
Prec

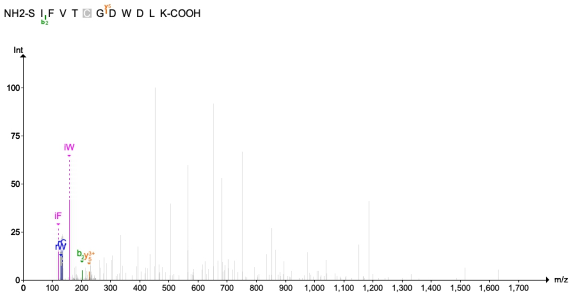
NH2-S I F V T C G D W D L K-COOH
b2
y5
Int
iW
iF
rC
rW
b2
y5 3+
m/z
0
25
50
75
100
0
100
200
300
400
500
600
700
800
900
1,000
1,100
1,200
1,300
1,400
1,500
1,600
1,700

NH2-S I L S E A P G K-COOH

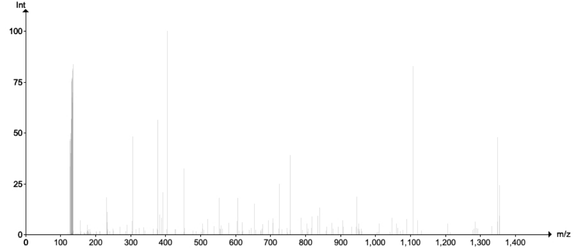

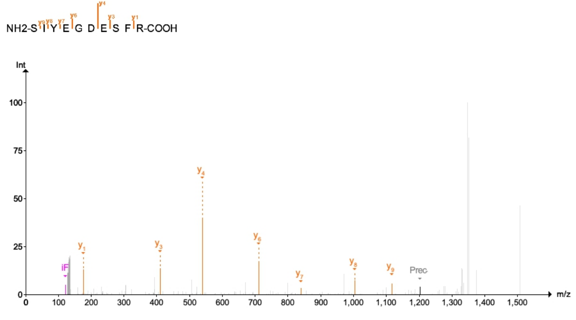

NH2-S I Y E G D E S F R-COOH
y9 y8 y7 y6 y4 y3 y1
Int
100
75
50
25
0
y1
y3
y4
y6
y7
y8
y9
iF
Prec
0
100
200
300
400
500
600
700
800
900
1,000
1,100
1,200
1,300
1,400
1,500
m/z

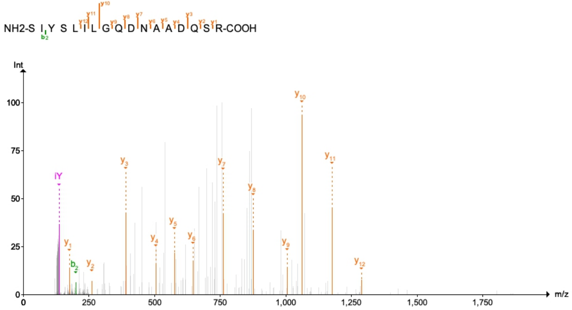

NH2-S I Y S L I L G Q D N A A D Q S R-COOH
b2
y12 y11 y10 y9 y8 y7 y6 y5 y4 y3 y2 y1
Int
100
75
50
25
0
iY
y1
b2
y2
y3
y4
y5
y6
y7
y8
y9
y10
y11
y12
0
250
500
750
1,000
1,250
1,500
1,750
m/z

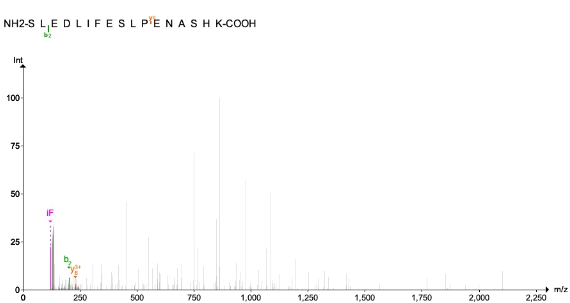
NH2-S L E D L I F E S L P E N A S H K-COOH
b2
y6
Int
100
75
50
25
0
iF
b2
y6 3+
0
250
500
750
1,000
1,250
1,500
1,750
2,000
2,250
m/z

NH2-S L E S A L K-COOH

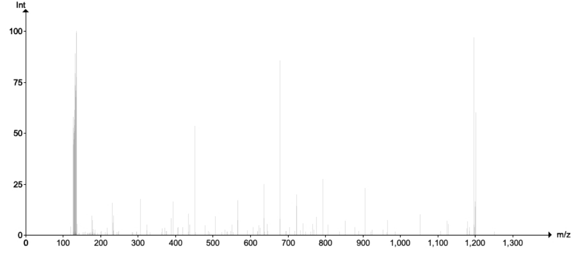

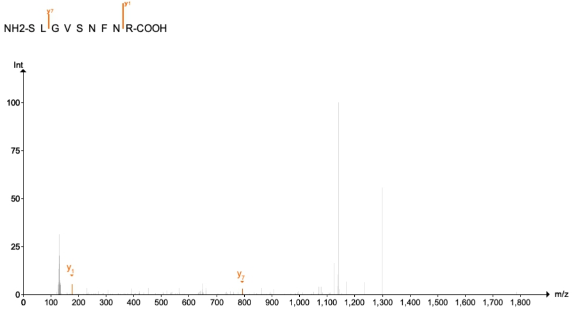

NH2-S L G V S N F N R-COOH
y7
y1
Int
100
75
50
25
0
y1
y7
0
100
200
300
400
500
600
700
800
900
1,000
1,100
1,200
1,300
1,400
1,500
1,600
1,700
1,800
m/z

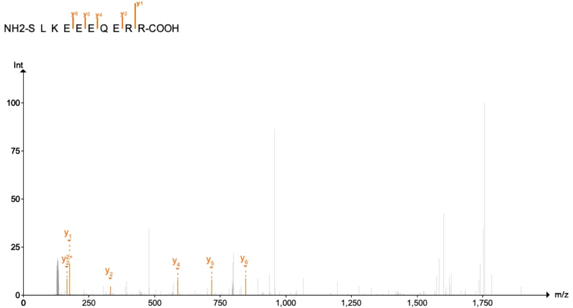

NH2-S L K E E E Q E R R-COOH
y6
y5
y4
y2
y1
Int
100
75
50
25
0
y1
y2 2+
y2
y4
y5
y6
0
250
500
750
1,000
1,250
1,500
1,750
m/z

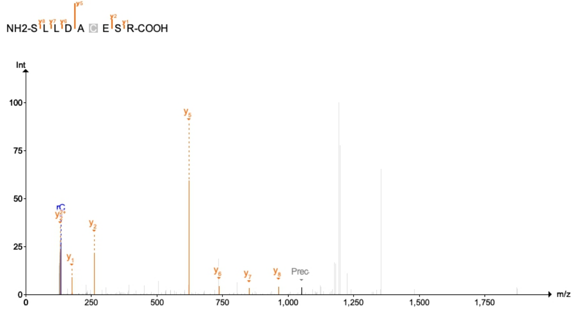

NH2-S L L D A C E S R-COOH
y8 y7 y6 y5 y2 y1
Int
100
75
50
25
0
rC
y_5^{2+}
y_1
y_2
y_5
y_6
y_7
y_8
Prec
0
250
500
750
1,000
1,250
1,500
1,750
m/z

NH2-S L L P T L E K-COOH

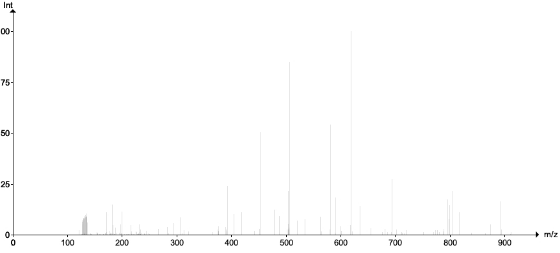

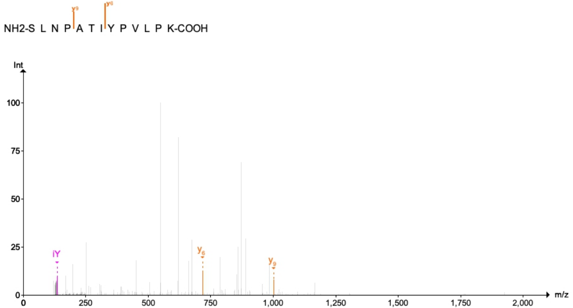

NH2-S L N P A T I Y P V L P K-COOH
y9
y6
Int
100
75
50
25
0
iY
y6
y9
0
250
500
750
1,000
1,250
1,500
1,750
2,000
m/z

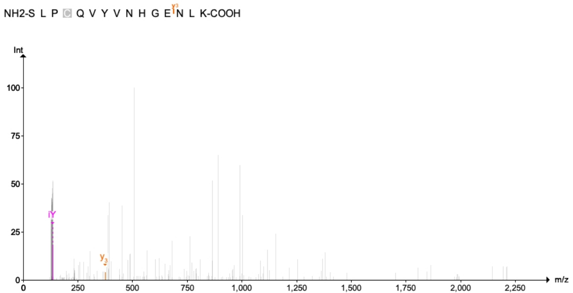
NH2-S L P C Q V Y V N H G E N L K-COOH
y3
Int
100
75
50
25
0
iY
y3
0
250
500
750
1,000
1,250
1,500
1,750
2,000
2,250
m/z

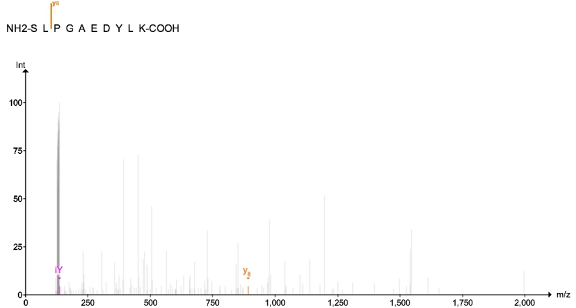

y8
NH2-S L P G A E D Y L K-COOH
Int
100
75
50
25
0
iY
y8
0
250
500
750
1,000
1,250
1,500
1,750
2,000
m/z

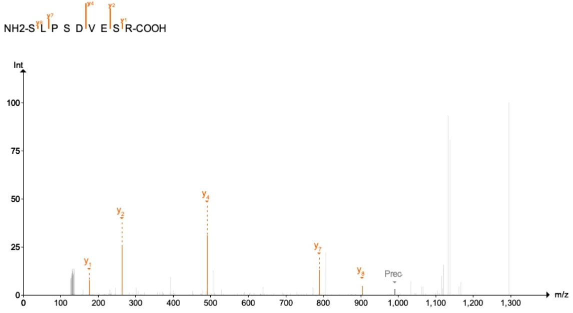

NH2-S L P S D V E S R-COOH
y8
y7
y4
y2
y1
Int
100
75
50
25
0
y1
y2
y4
y7
y8
Prec
0
100
200
300
400
500
600
700
800
900
1,000
1,100
1,200
1,300
m/z

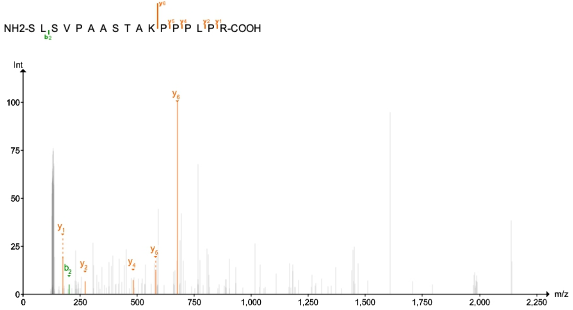
NH2-S L S V P A A S T A K P P P L P R-COOH
b2
y6
y5
y4
y2
y1
Int
100
75
50
25
0
y6
y1
b2
y2
y4
y5
0
250
500
750
1,000
1,250
1,500
1,750
2,000
2,250
m/z

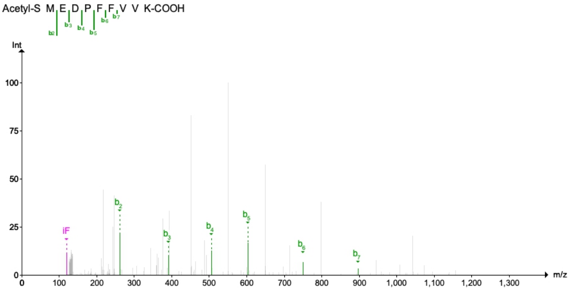
Acetyl-S M E D P F F V V K-COOH
b2
b3
b4
b5
b6
b7
Int
100
75
50
25
0
iF
b2
b3
b4
b5
b6
b7
0
100
200
300
400
500
600
700
800
900
1,000
1,100
1,200
1,300
m/z

NH2-S P F E Q H I K-COOH

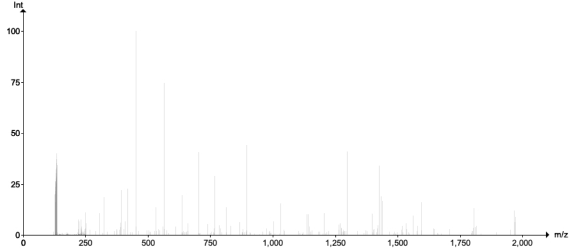

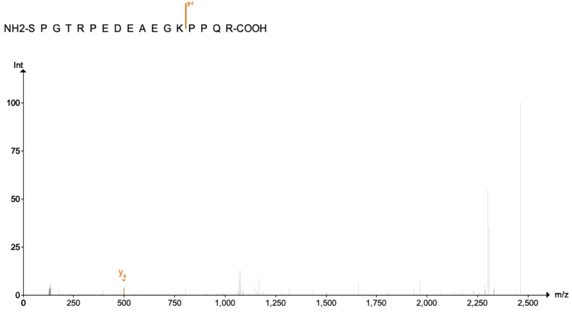

y4
NH2-S P G T R P E D E A E G K P P Q R-COOH
Int
100
75
50
25
0
y4
0
250
500
750
1,000
1,250
1,500
1,750
2,000
2,250
2,500
m/z

NH2-S P I S V A A A A I Y M A S Q A S A E K-COOH

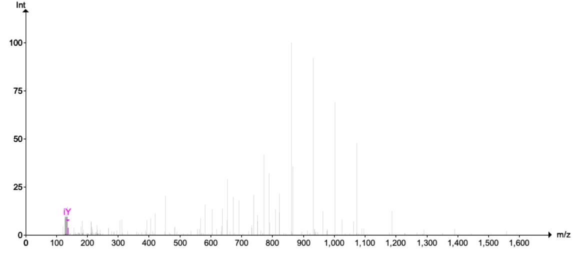

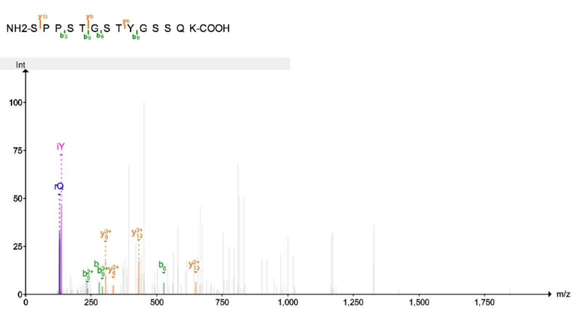
NH2-S P P S T G S T Y G S S Q K-COOH
y13
y9
y6
b3
b5
b6
b9
Int
100
75
50
25
0
iY
rQ
y9 3+
y13 3+
b5 2+
b9 3+
y6 2+
b6
y13 2+
0
250
500
750
1,000
1,250
1,500
1,750
m/z

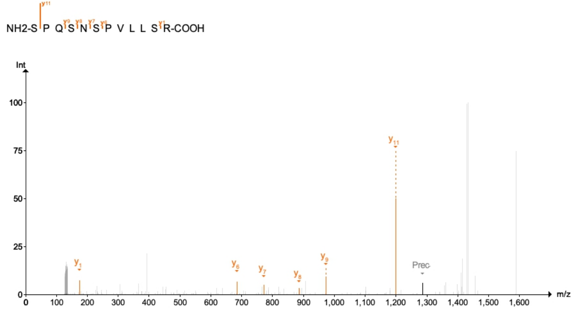

NH2-S P Q S N S P V L L S R-COOH
y11
y9
y8
y7
y6
y1
Int
100
75
50
25
0
y1
y6
y7
y8
y9
y11
Prec
0
100
200
300
400
500
600
700
800
900
1,000
1,100
1,200
1,300
1,400
1,500
1,600
m/z

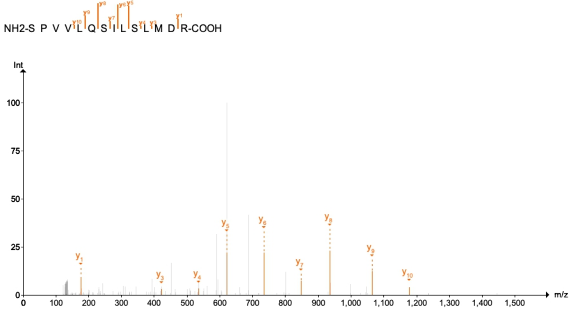
NH2-S P V V L Q S I L S L M D R-COOH
y10 y9 y8 y7 y6 y5 y4 y3 y1
Int
100
75
50
25
0
y1
y3
y4
y5
y6
y7
y8
y9
y10
0 100 200 300 400 500 600 700 800 900 1,000 1,100 1,200 1,300 1,400 1,500
m/z

NH2-S Q D L S L Q E A E T E Q S D T L D N K-COOH

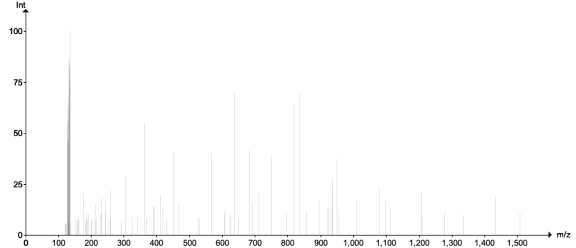

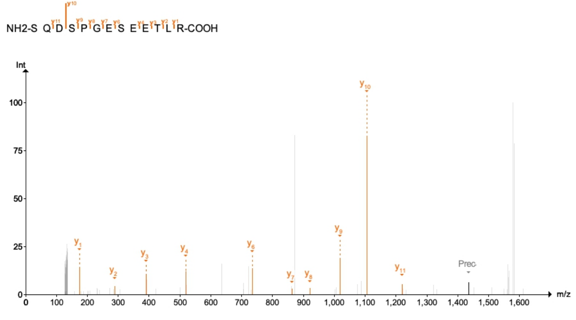

y10
NH2-S Q y11 D S y9 P y8 G y7 E y6 S E y4 E y3 T y2 L y1 R-COOH
Int
100
75
50
25
0
y1
y2
y3
y4
y6
y7
y8
y9
y10
y11
Prec
0 100 200 300 400 500 600 700 800 900 1,000 1,100 1,200 1,300 1,400 1,500 1,600
m/z

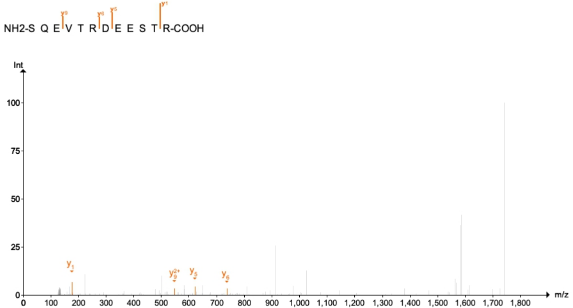

NH2-S Q E V T R D E E S T R-COOH
y9
y6
y5
y1
Int
100
75
50
25
0
y1
y9 2+
y5
y6
m/z
0
100
200
300
400
500
600
700
800
900
1,000
1,100
1,200
1,300
1,400
1,500
1,600
1,700
1,800

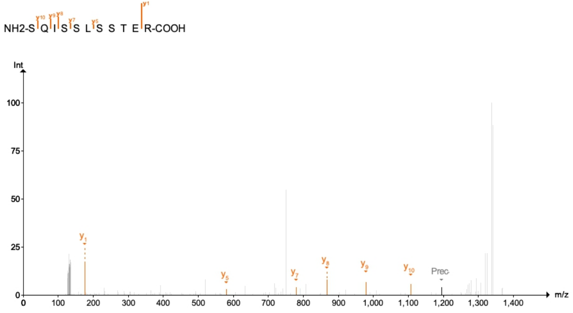

NH2-S Q I S S L S S T E R-COOH
y10 y9 y8 y7 y5 y1
Int
100
75
50
25
0
y1
y5
y7
y8
y9
y10
Prec
0 100 200 300 400 500 600 700 800 900 1,000 1,100 1,200 1,300 1,400
m/z

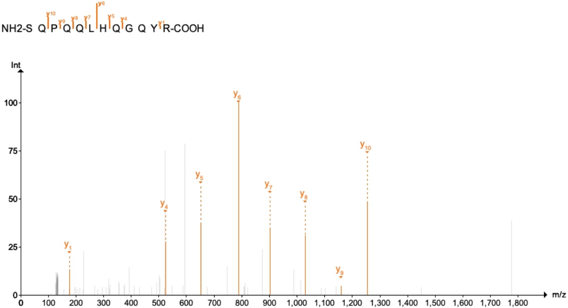

NH2-S Q P Q Q L H Q G Q Y R-COOH
y10 y9 y8 y7 y6 y5 y4 y1
Int
100
75
50
25
0
y1
y4
y5
y6
y7
y8
y9
y10
0 100 200 300 400 500 600 700 800 900 1,000 1,100 1,200 1,300 1,400 1,500 1,600 1,700 1,800
m/z

NH2-S Q Q Q F L I H Q Q S G E Q K-COOH

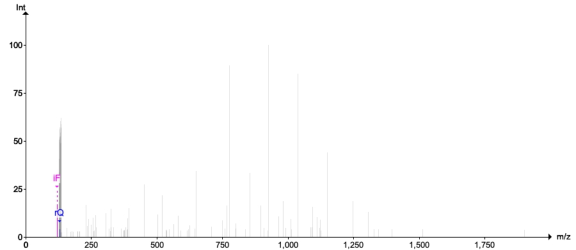

NH2-S Q W S P A L T V S K-COOH

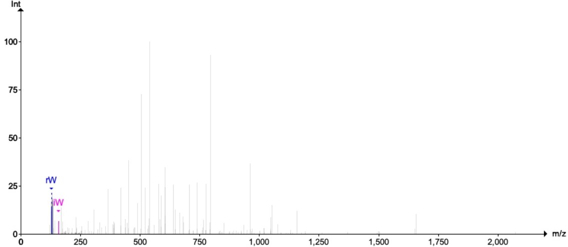

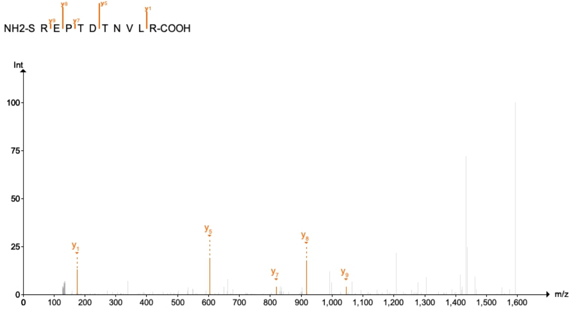

NH2-S R E P T D T N V L R-COOH
y9
y8
y7
y5
y1
Int
m/z
y1
y5
y7
y8
y9
100
75
50
25
0
0
100
200
300
400
500
600
700
800
900
1,000
1,100
1,200
1,300
1,400
1,500
1,600

NH2-S R P I Y V S T V R P Q S K-COOH

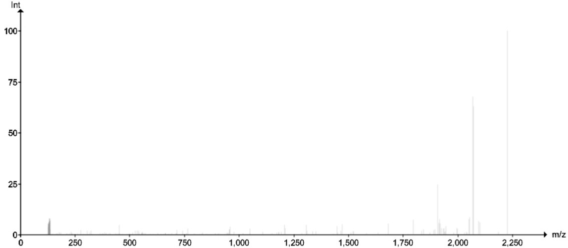

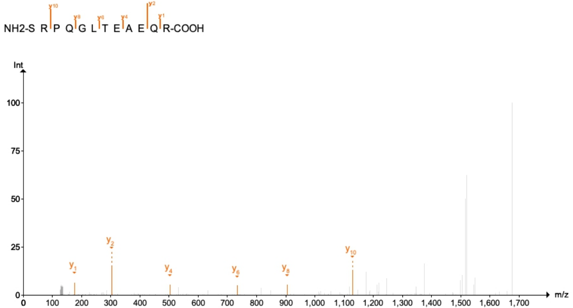
NH2-S R P Q G L T E A E Q R-COOH
y10
y8
y6
y4
y2
y1
Int
100
75
50
25
0
m/z
0
100
200
300
400
500
600
700
800
900
1,000
1,100
1,200
1,300
1,400
1,500
1,600
1,700

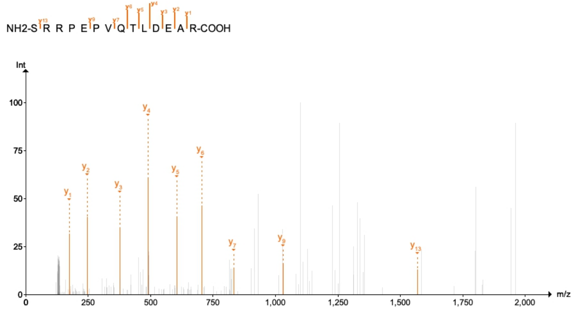

NH2-S R R P E P V Q T L D E A R-COOH
y13
y9
y7
y6
y5
y4
y3
y2
y1
Int
100
75
50
25
0
y1
y2
y3
y4
y5
y6
y7
y9
y13
m/z
0
250
500
750
1,000
1,250
1,500
1,750
2,000

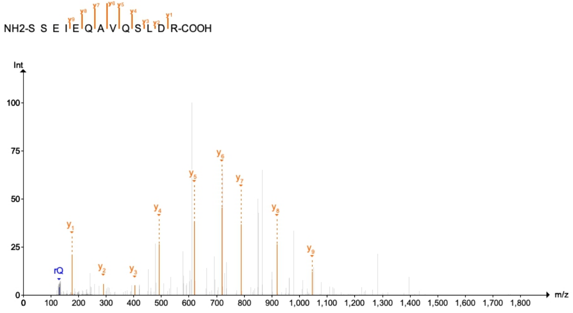
NH2-S S E I E Q A V Q S L D R-COOH
y9 y8 y7 y6 y5 y4 y3 y2 y1
Int
100
75
50
25
0
rQ
y1
y2
y3
y4
y5
y6
y7
y8
y9
0 100 200 300 400 500 600 700 800 900 1,000 1,100 1,200 1,300 1,400 1,500 1,600 1,700 1,800
m/z

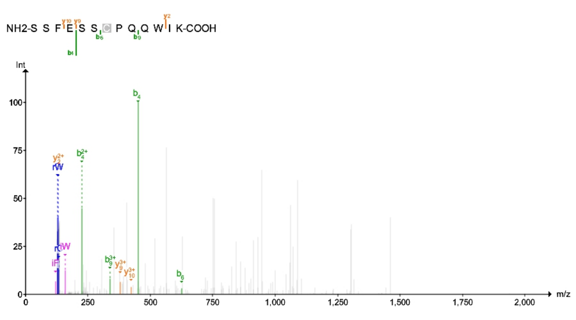
NH2-S S F E S S C P Q Q W I K-COOH
Int
m/z
b4
b4 2+
y2 2+
rW
rC
iW
iF
b9 3+
y9 3+
y10 3+
b6
0
25
50
75
100
250
500
750
1,000
1,250
1,500
1,750
2,000

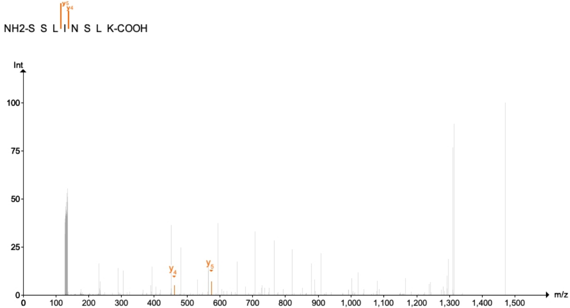

NH2-S S L I N S L K-COOH
y5
y4
Int
100
75
50
25
0
y4
y5
0
100
200
300
400
500
600
700
800
900
1,000
1,100
1,200
1,300
1,400
1,500
m/z

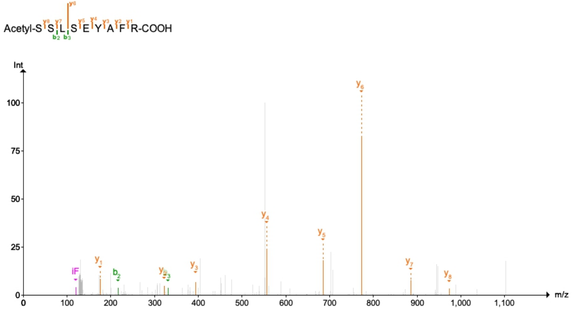

Acetyl-SSLSEYAFR-COOH
y8 y7 y6 y5 y4 y3 y2 y1
b2 b3
Int
100
75
50
25
0
iF
y1
b2
y2
b3
y3
y4
y5
y6
y7
y8
0
100
200
300
400
500
600
700
800
900
1,000
1,100
m/z

NH2-S S P E V S S I N Q E A L V L T A K-COOH

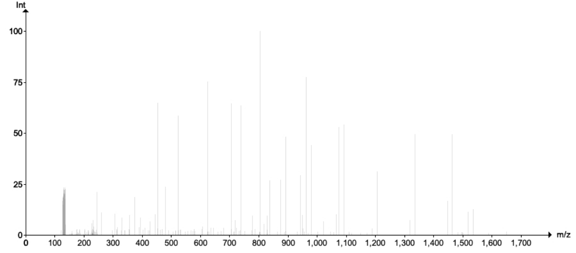

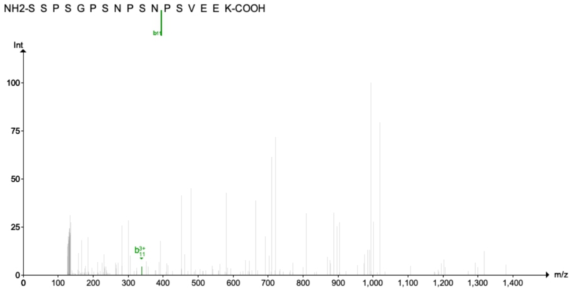

NH2-S S P S G P S N P S N P S V E E K-COOH
b11
Int
100
75
50
25
0
b^{3+}_{11}
0 100 200 300 400 500 600 700 800 900 1,000 1,100 1,200 1,300 1,400
m/z

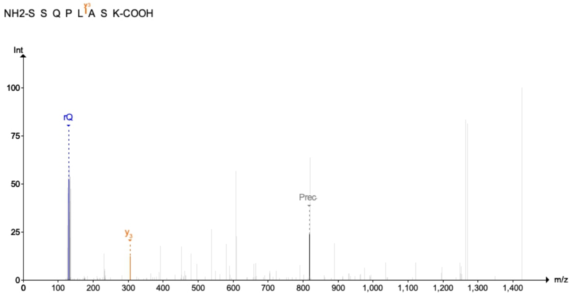
NH2-S S Q P L A S K-COOH
y3
Int
100
75
50
25
0
rQ
y3
Prec
0
100
200
300
400
500
600
700
800
900
1,000
1,100
1,200
1,300
1,400
m/z

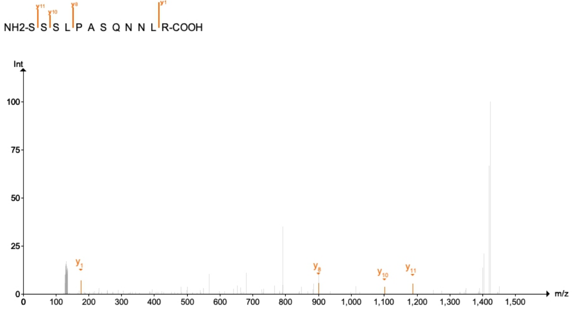

NH2-S S S L P A S Q N N L R-COOH
y11
y10
y8
y1
Int
100
75
50
25
0
y1
y8
y10
y11
0
100
200
300
400
500
600
700
800
900
1,000
1,100
1,200
1,300
1,400
1,500
m/z

NH2-S S S P T I A L P M L L Q A L A L S K-COOH

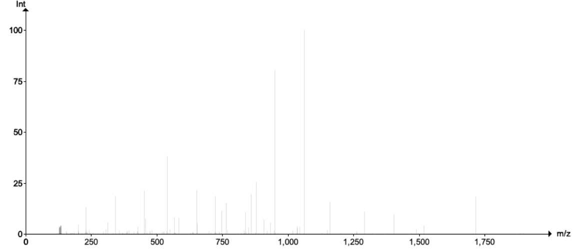

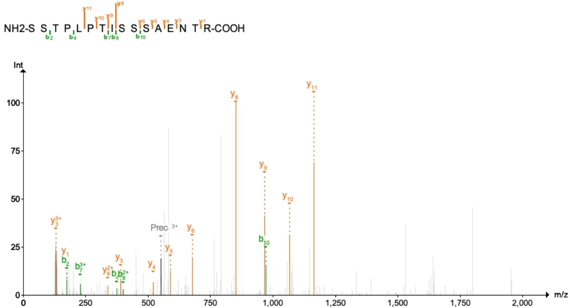

NH2-S S T P L P T I S S S A E N T R-COOH
Int
m/z
y8
y11
y9
y10
Prec 3+
y6
b10
y5
y4
y3
y1
b2
b7 3+
y6 2+
b4
b8 2+
y3 3+
0
25
50
75
100
0
250
500
750
1,000
1,250
1,500
1,750
2,000

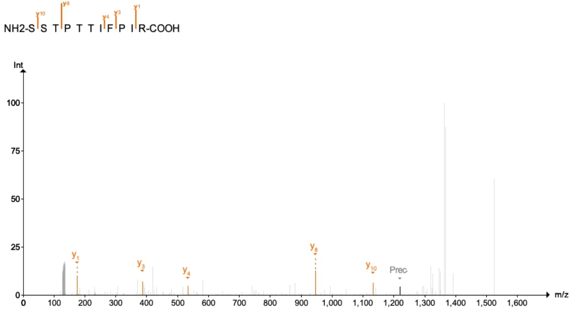

NH2-S S T P T T I F P I R-COOH
y10
y8
y4
y3
y1
Int
100
75
50
25
0
y1
y3
y4
y8
y10
Prec
0
100
200
300
400
500
600
700
800
900
1,000
1,100
1,200
1,300
1,400
1,500
1,600
m/z

NH2-S S T T T T P V Q S G A N V V K-COOH

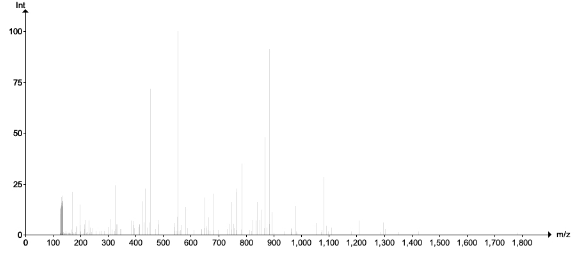

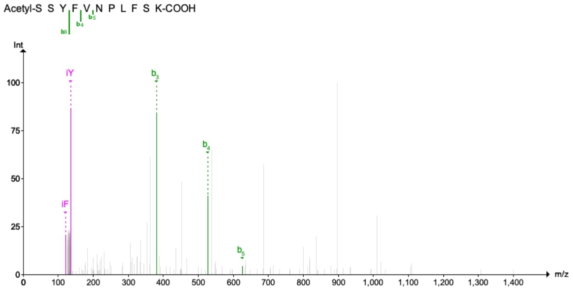

Acetyl-S S Y F V N P L F S K-COOH
b3
b4
b5
Int
iY
b3
b4
iF
b5
100
75
50
25
0
0
100
200
300
400
500
600
700
800
900
1,000
1,100
1,200
1,300
1,400
m/z

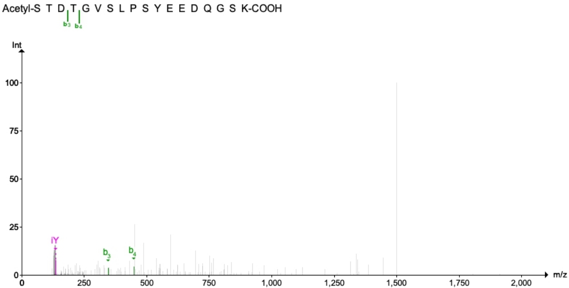

Acetyl-S T D T G V S L P S Y E E D Q G S K-COOH
b3
b4
Int
100
75
50
25
0
iY
b3
b4
0
250
500
750
1,000
1,250
1,500
1,750
2,000
m/z

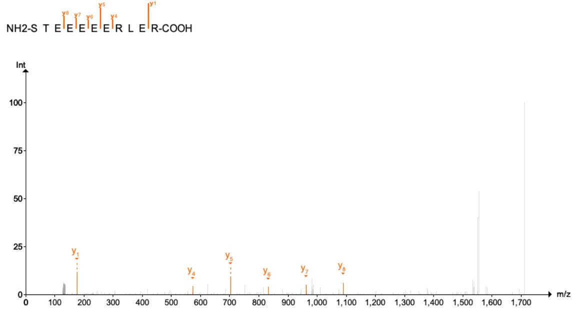

NH2-S T E E E E E R L E R-COOH
y8
y7
y6
y5
y4
y1
Int
m/z
100
75
50
25
0
0
100
200
300
400
500
600
700
800
900
1,000
1,100
1,200
1,300
1,400
1,500
1,600
1,700
y1
y4
y5
y6
y7
y8

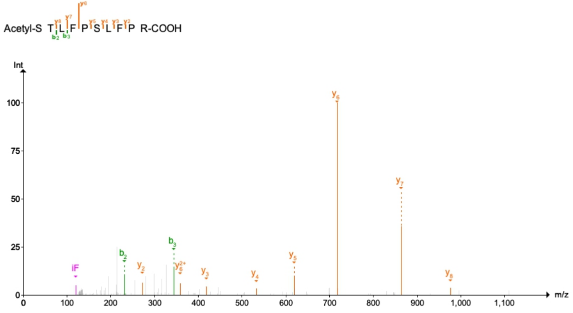

Acetyl-S T L F P S L F P R-COOH
y8 y7 y6 y5 y4 y3 y2
b2 b3
Int
100
75
50
25
0
iF
b2
y2
b3
y6 2+
y3
y4
y5
y6
y7
y8
0 100 200 300 400 500 600 700 800 900 1,000 1,100
m/z

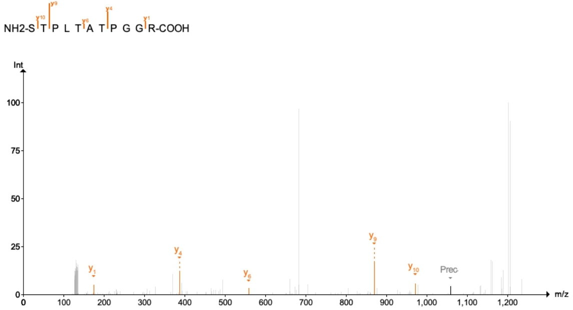

NH2-S T P L T A T P G G R-COOH
y10
y9
y6
y4
y1
Int
100
75
50
25
0
y1
y4
y6
y9
y10
Prec
100
200
300
400
500
600
700
800
900
1,000
1,100
1,200
m/z

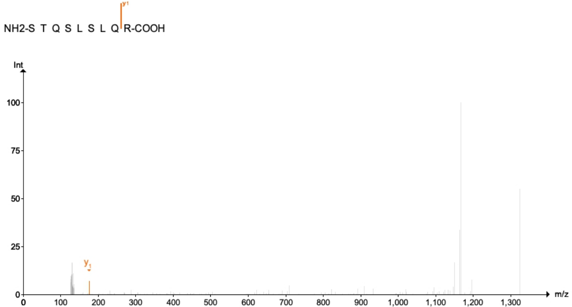

NH2-S T Q S L S L Q R-COOH
y1
Int
m/z
0
25
50
75
100
0
100
200
300
400
500
600
700
800
900
1,000
1,100
1,200
1,300

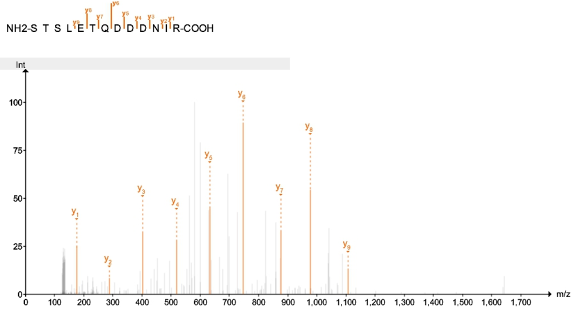
NH2-S T S L E T Q D D D N I R-COOH
y9 y8 y7 y6 y5 y4 y3 y2 y1
Int
y1 y2 y3 y4 y5 y6 y7 y8 y9
m/z
0
25
50
75
100
0 100 200 300 400 500 600 700 800 900 1,000 1,100 1,200 1,300 1,400 1,500 1,600 1,700

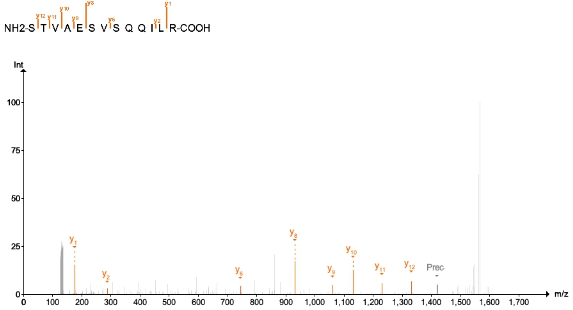
NH2-S T V A E S V S Q Q I L R-COOH
y12
y11
y10
y9
y8
y6
y2
y1
Int
100
75
50
25
0
y1
y2
y6
y8
y9
y10
y11
y12
Prec
0
100
200
300
400
500
600
700
800
900
1,000
1,100
1,200
1,300
1,400
1,500
1,600
1,700
m/z

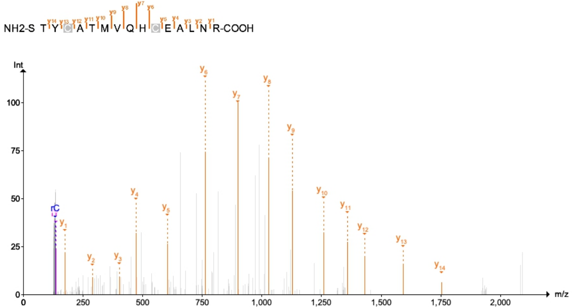
NH2-S T Y C A T M V Q H C E A L N R-COOH
Int
m/z
rC
y1
y2
y3
y4
y5
y6
y7
y8
y9
y10
y11
y12
y13
y14

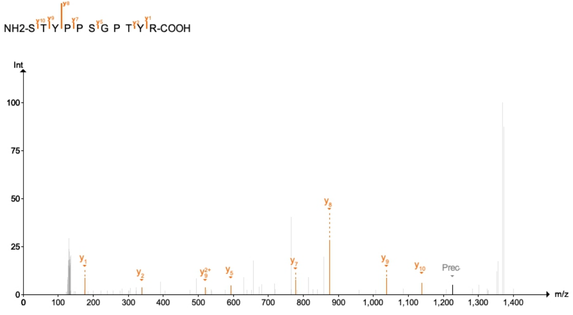
NH2-S T Y P P S G P T Y R-COOH
y10 y9 y8 y7 y5 y2 y1
Int
100
75
50
25
0
y1
y2
y9 2+
y5
y7
y8
y9
y10
Prec
m/z
0 100 200 300 400 500 600 700 800 900 1,000 1,100 1,200 1,300 1,400

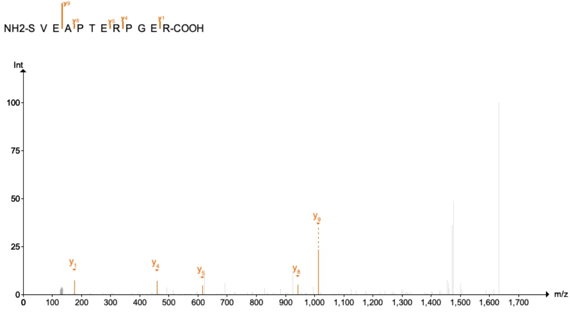
NH2-S V E A P T E R P G E R-COOH
y9
y8
y5
y4
y1
Int
100
75
50
25
0
m/z
0
100
200
300
400
500
600
700
800
900
1,000
1,100
1,200
1,300
1,400
1,500
1,600
1,700

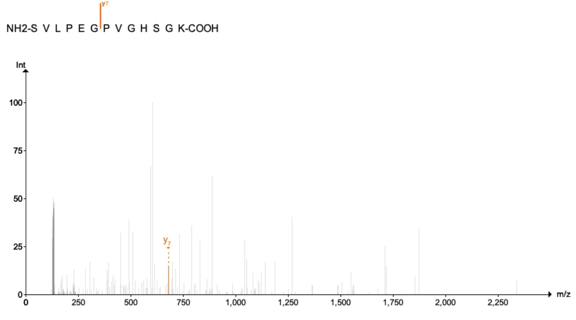

y7
NH2-S V L P E G P V G H S G K-COOH
Int
100
75
50
25
0
y7
0
250
500
750
1,000
1,250
1,500
1,750
2,000
2,250
m/z

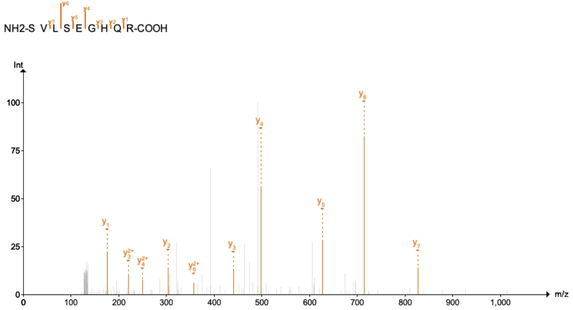

NH2-S V L S E G H Q R-COOH
y7 y6 y5 y4 y3 y2 y1
Int
100
75
50
25
0
y1
y3 2+
y4 2+
y2
y6 2+
y3
y4
y5
y6
y7
0 100 200 300 400 500 600 700 800 900 1,000
m/z

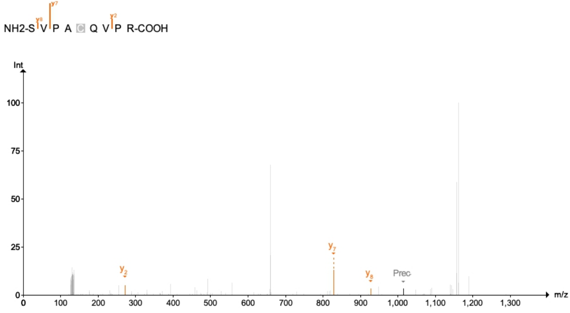
NH2-S V P A C Q V P R-COOH
y8
y7
y2
Int
100
75
50
25
0
y2
y7
y8
Prec
0
100
200
300
400
500
600
700
800
900
1,000
1,100
1,200
1,300
m/z

NH2-S V S F S D V F F K-COOH

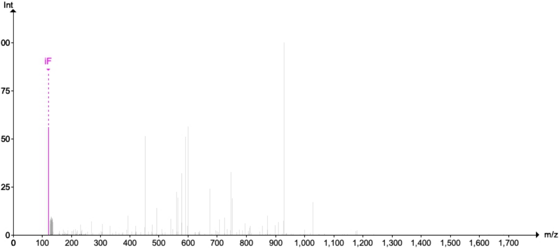

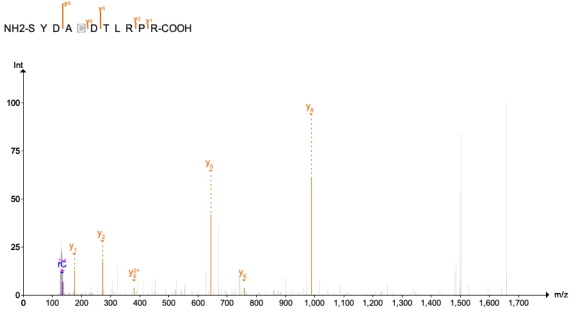
NH2-S Y D A C D T L R P R-COOH
Int
m/z
y8
y6
y5
y2
y1
rC
y2
y1
y6 2+
y5
y6
y8
100
75
50
25
0
0
100
200
300
400
500
600
700
800
900
1,000
1,100
1,200
1,300
1,400
1,500
1,600
1,700

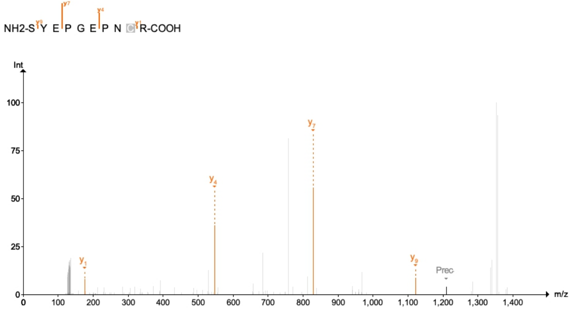

NH2-S Y E P G E P N C R-COOH
y9
y7
y4
y1
Int
100
75
50
25
0
y1
y4
y7
y9
Prec
0
100
200
300
400
500
600
700
800
900
1,000
1,100
1,200
1,300
1,400
m/z

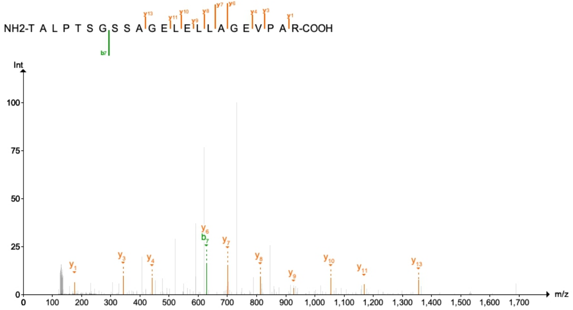

NH2-T A L P T S G S S A G E L E L L A G E V P A R-COOH
y13 y11 y10 y9 y8 y7 y6 y4 y3 y1
b7
Int
m/z
y1 y3 y4 y6 b7 y7 y8 y9 y10 y11 y13

NH2-T A S T S F T N I A Y D L C A K-COOH

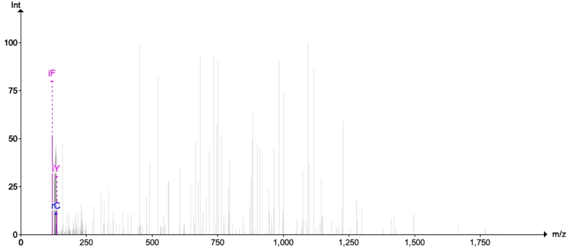

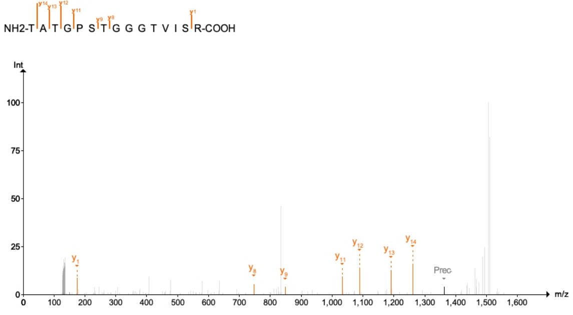

NH2-T A T G P S T G G G T V I S R-COOH
y14
y13
y12
y11
y9
y8
y1
Int
100
75
50
25
0
y1
y8
y9
y11
y12
y13
y14
Prec
m/z
0
100
200
300
400
500
600
700
800
900
1,000
1,100
1,200
1,300
1,400
1,500
1,600

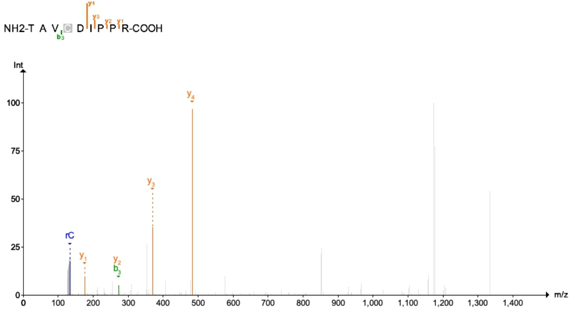
NH2-T A V C D I P P R-COOH
Int
m/z
y4
y3
y2
y1
rC
b3
0
25
50
75
100
0
100
200
300
400
500
600
700
800
900
1,000
1,100
1,200
1,300
1,400

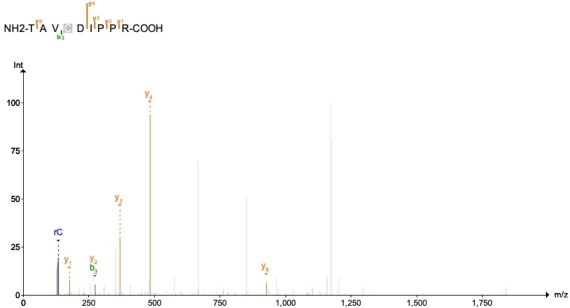

NH2-T A V C D I P P R-COOH
y8
b3
y4
y3
y2
y1
Int
100
75
50
25
0
rC
y1
y2
b3
y3
y4
y8
250
500
750
1,000
1,250
1,500
1,750
m/z

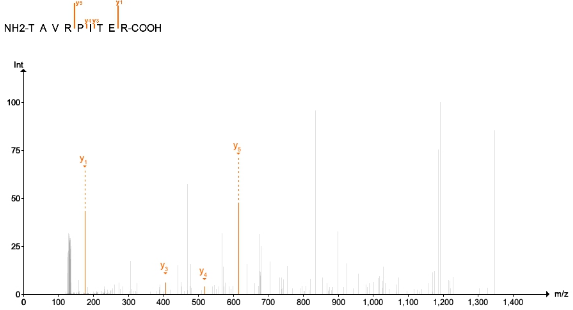

NH2-T A V R P I T E R-COOH
y5
y4
y3
y1
Int
100
75
50
25
0
y1
y3
y4
y5
0
100
200
300
400
500
600
700
800
900
1,000
1,100
1,200
1,300
1,400
m/z

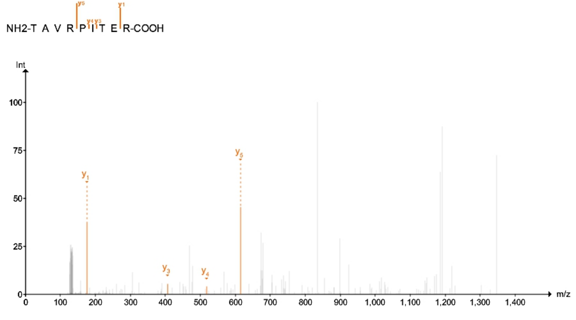

NH2-T A V R P I T E R-COOH
y5
y4
y3
y1
Int
100
75
50
25
0
y1
y3
y4
y5
0
100
200
300
400
500
600
700
800
900
1,000
1,100
1,200
1,300
1,400
m/z

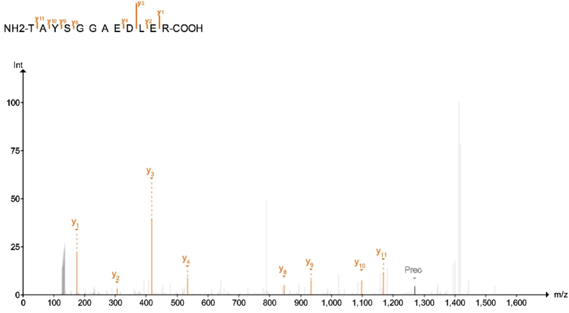
NH2-T A Y S G G A E D L E R-COOH
y11
y10
y9
y8
y4
y3
y2
y1
Int
100
75
50
25
0
y1
y2
y3
y4
y8
y9
y10
y11
Prec
0
100
200
300
400
500
600
700
800
900
1,000
1,100
1,200
1,300
1,400
1,500
1,600
m/z

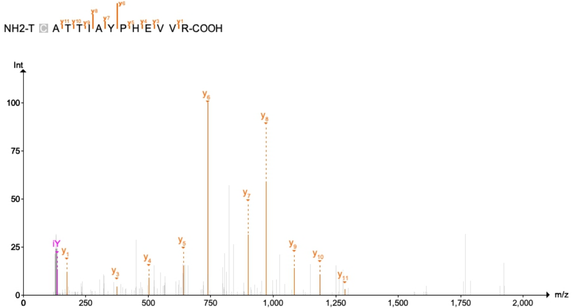

NH2-T C A T T I A Y P H E V V R-COOH
y11 y10 y9 y8 y7 y6 y5 y4 y3 y1
Int
100
75
50
25
0
iY
y1
y3
y4
y5
y6
y7
y8
y9
y10
y11
m/z
0
250
500
750
1,000
1,250
1,500
1,750
2,000

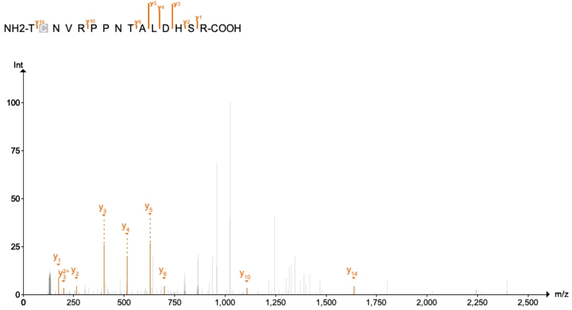

NH2-T C N V R P P N T A L D H S R-COOH
Int
100
75
50
25
0
y1
y3 2+
y2
y3
y4
y5
y6
y10
y14
y5
y4
y3
y2
y1
y6
y10
y14
0
250
500
750
1,000
1,250
1,500
1,750
2,000
2,250
2,500
m/z

NH2-T C P T C N D F H G L V Q K-COOH

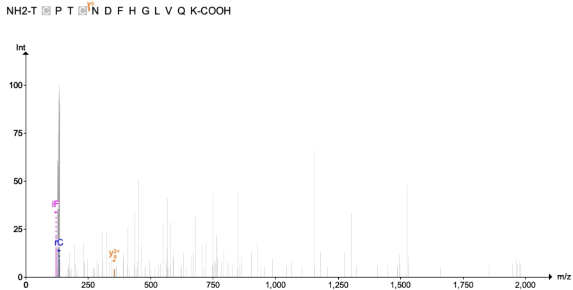

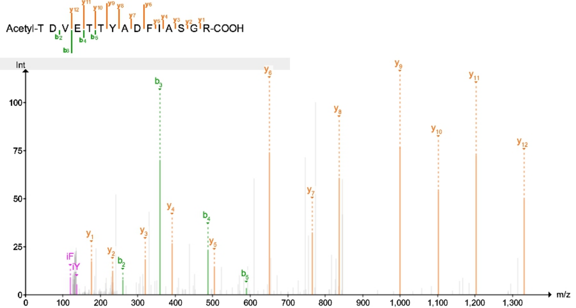

Acetyl-T D V E T T Y A D F I A S G R-COOH
y12 y11 y10 y9 y8 y7 y6 y5 y4 y3 y2 y1
b2 b3 b4 b5
Int
100
75
50
25
0
y6 y9 y11 y8 y10 y12 b3 y7 y4 b4 y5 y1 y3 y2 b2 b5 iF iY
0 100 200 300 400 500 600 700 800 900 1,000 1,100 1,200 1,300
m/z

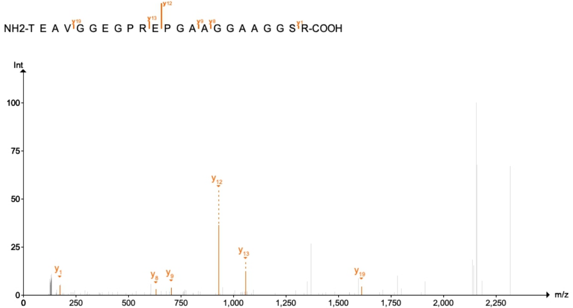
NH2-T E A V G G E G P R E P G A A G G A A G G S R-COOH
y19
y13
y12
y9
y8
y1
Int
100
75
50
25
0
m/z
0
250
500
750
1,000
1,250
1,500
1,750
2,000
2,250
y1
y8
y9
y12
y13
y19

NH2-T E D I H L F Q Q V K-COOH

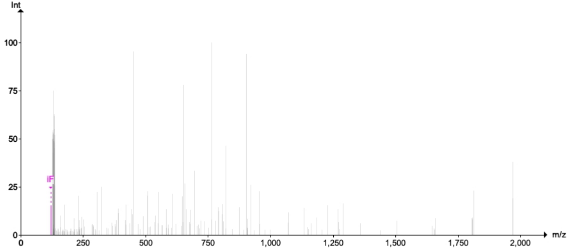

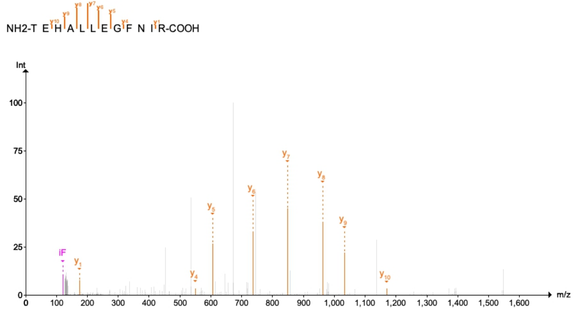

NH2-T E H A L L E G F N I R-COOH
y10 y9 y8 y7 y6 y5 y4 y1
Int
m/z
iF
y1 y4 y5 y6 y7 y8 y9 y10
0 25 50 75 100
0 100 200 300 400 500 600 700 800 900 1,000 1,100 1,200 1,300 1,400 1,500 1,600

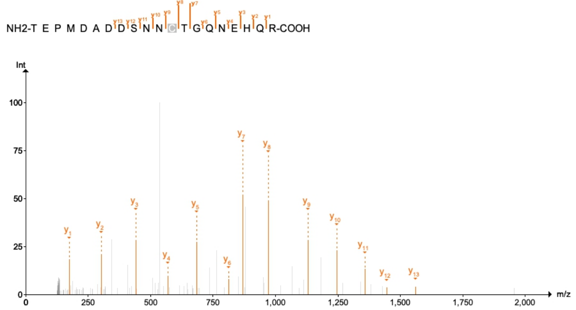

NH2-T E P M D A D D S N N C T G Q N E H Q R-COOH
Int
m/z
y1
y2
y3
y4
y5
y6
y7
y8
y9
y10
y11
y12
y13

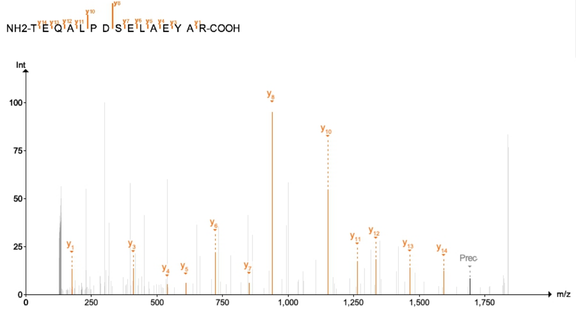

NH2-T E Q A L P D S E L A E Y A R-COOH
Int
m/z
Prec
y1
y3
y4
y5
y6
y7
y8
y10
y11
y12
y13
y14
0
250
500
750
1,000
1,250
1,500
1,750
25
50
75
100

NH2-T F S Y P L D L L L K-COOH

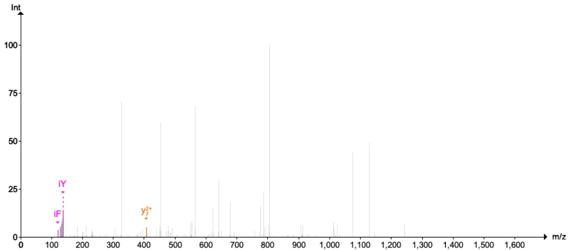

NH2-T H L P D F L I C K-COOH

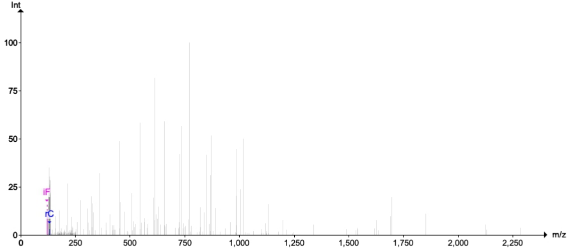

NH2-T H L Q D L K-COOH

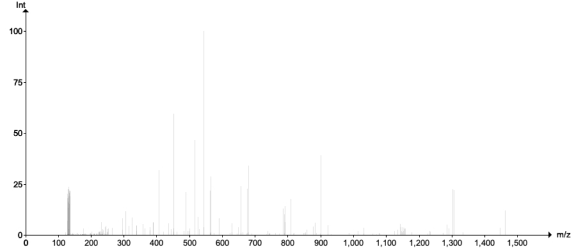

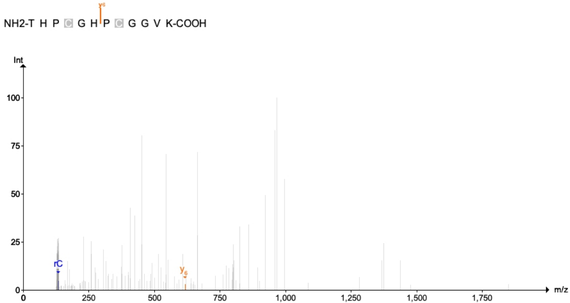
NH2-T H P C G H P C G G V K-COOH
y6
Int
100
75
50
25
0
rC
y6
0
250
500
750
1,000
1,250
1,500
1,750
m/z

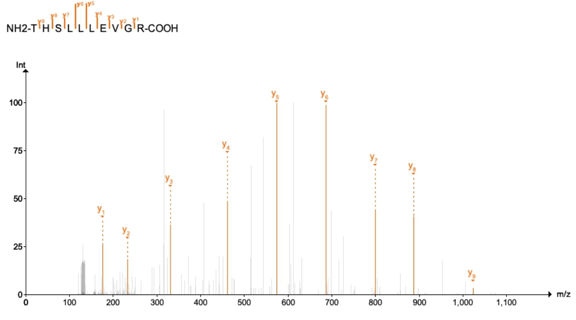

NH2-T H S L L L E V G R-COOH
y9 y8 y7 y6 y5 y4 y3 y2 y1
Int
100
75
50
25
0
y1
y2
y3
y4
y5
y6
y7
y8
y9
0 100 200 300 400 500 600 700 800 900 1,000 1,100
m/z

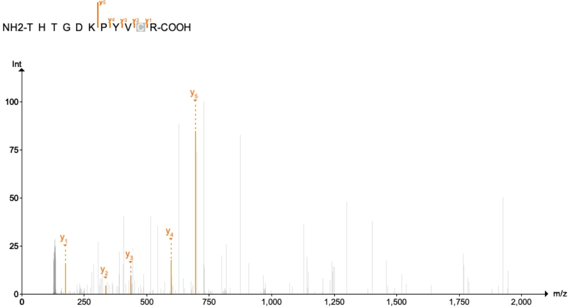
NH2-T H T G D K P Y V C R-COOH
y5
y4
y3
y2
y1
Int
100
75
50
25
0
m/z
0
250
500
750
1,000
1,250
1,500
1,750
2,000

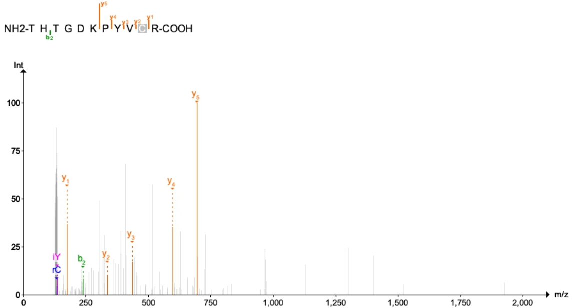
NH2-T H T G D K P Y V C R-COOH
y5
y4
y3
y2
y1
b2
Int
100
75
50
25
0
y5
y4
y1
y3
y2
b2
iY
rC
m/z
0
250
500
750
1,000
1,250
1,500
1,750
2,000

NH2-T H T G E K P Y E C K-COOH

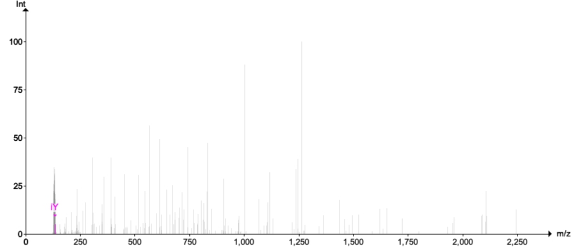

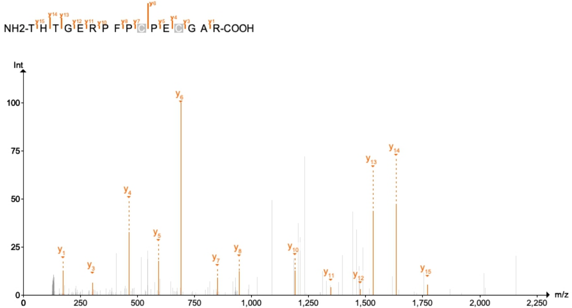
NH2-T H T G E R P F P C P E C G A R-COOH
y15 y14 y13 y12 y11 y10 y8 y7 y6 y5 y4 y3 y1
Int
100
75
50
25
0
y1
y3
y4
y5
y6
y7
y8
y10
y11
y12
y13
y14
y15
0
250
500
750
1,000
1,250
1,500
1,750
2,000
2,250
m/z

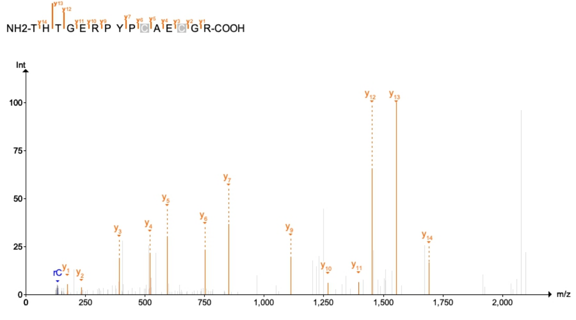

NH2-T H T G E R P Y P C A E C G R-COOH
y14 y13 y12 y11 y10 y9 y7 y6 y5 y4 y3 y2 y1
Int
m/z
rC
y1
y2
y3
y4
y5
y6
y7
y9
y10
y11
y12
y13
y14
0
25
50
75
100
250
500
750
1,000
1,250
1,500
1,750
2,000

NH2-T H T G E R P Y P C P Q C G K-COOH

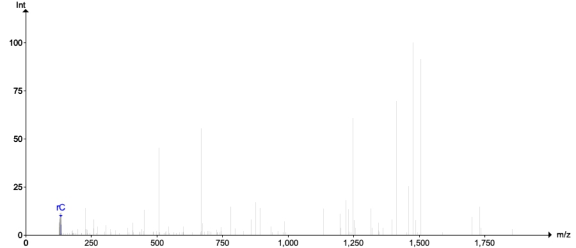

NH2-T I D Y I Q F L H K-COOH

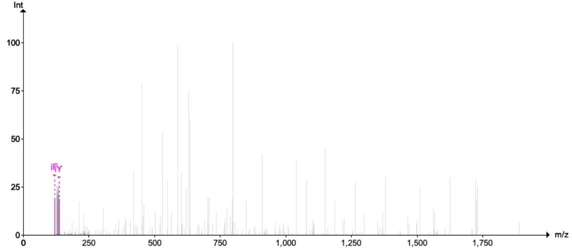

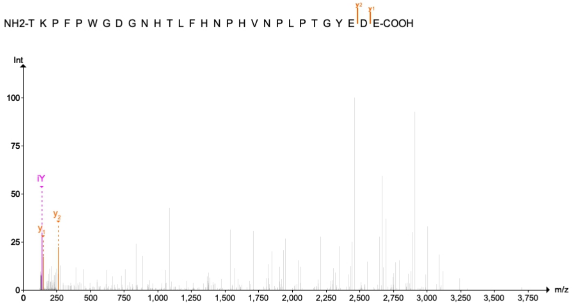

NH2-T K P F P W G D G N H T L F H N P H V N P L P T G Y E D E-COOH
y2
y1
Int
100
75
50
25
0
iY
y2
y1
0
250
500
750
1,000
1,250
1,500
1,750
2,000
2,250
2,500
2,750
3,000
3,250
3,500
3,750
m/z

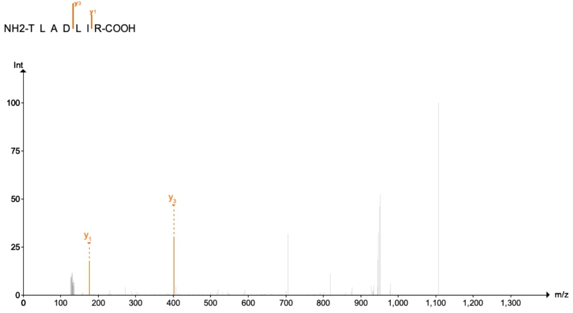
NH2-T L A D L I R-COOH
y3
y1
Int
100
75
50
25
0
y3
y1
0
100
200
300
400
500
600
700
800
900
1,000
1,100
1,200
1,300
m/z

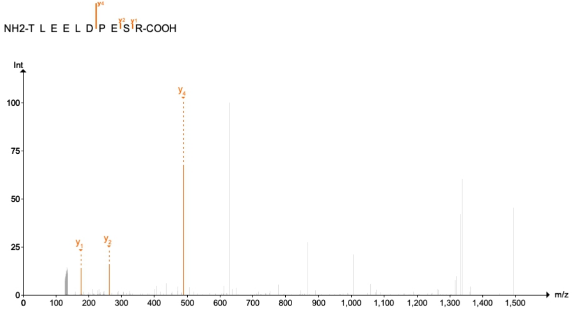

NH2-T L E E L D P E S R-COOH
y4
y2
y1
Int
100
75
50
25
0
y1
y2
y4
0
100
200
300
400
500
600
700
800
900
1,000
1,100
1,200
1,300
1,400
1,500
m/z

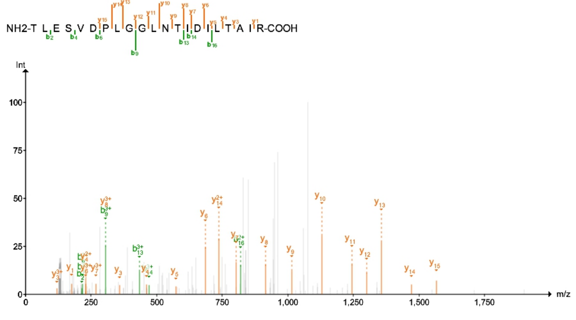
NH2-T L E S V D P L G G L N T I D I L T A I R-COOH
Int
m/z
y10
y13
y6
y$^{2+}_{14}$
y$^{3+}_{8}$
b$^{3+}_{9}$
y11
y12
y8
y9
y14
y15
y5
y3
y1
b$^{3+}_{13}$

NH2-T L L L N P K-COOH

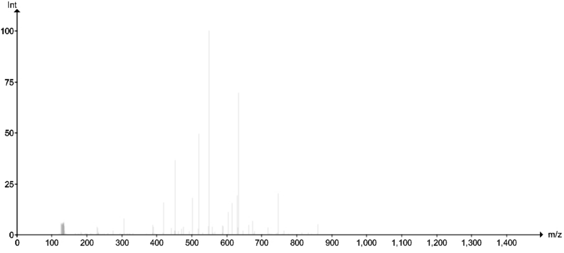

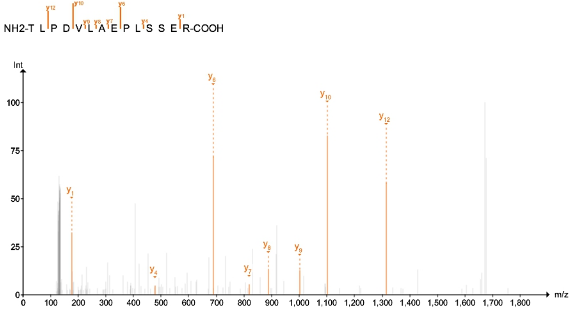
NH2-T L P D V L A E P L S S E R-COOH
y12
y10
y9
y8
y7
y6
y4
y1
Int
m/z
y1
y4
y6
y7
y8
y9
y10
y12
0
25
50
75
100
0
100
200
300
400
500
600
700
800
900
1,000
1,100
1,200
1,300
1,400
1,500
1,600
1,700
1,800

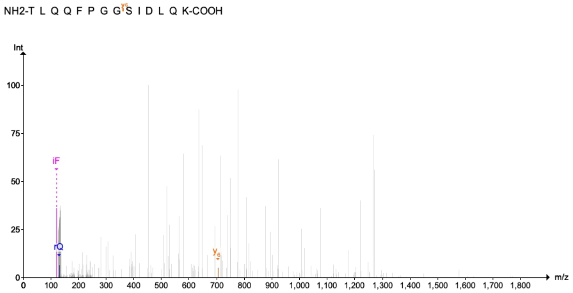
NH2-T L Q Q F P G G S I D L Q K-COOH
y6
Int
100
75
50
25
0
iF
rQ
y6
0
100
200
300
400
500
600
700
800
900
1,000
1,100
1,200
1,300
1,400
1,500
1,600
1,700
1,800
m/z

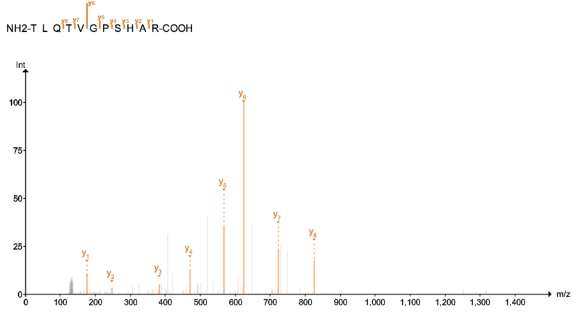

NH2-T L Q T V G P S H A R-COOH
y8 y7 y6 y5 y4 y3 y2 y1
Int
m/z
y1 y2 y3 y4 y5 y6 y7 y8
0 25 50 75 100
0 100 200 300 400 500 600 700 800 900 1,000 1,100 1,200 1,300 1,400

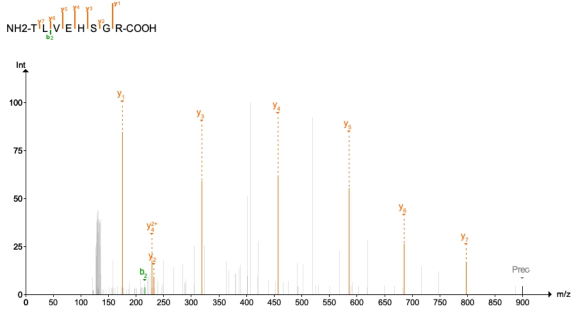
NH2-T L V E H S G R-COOH
y7 y6 y5 y4 y3 y2 y1
b2
Int
m/z
y1
y3
y4
y5
y6
y7
y4 2+
y2
b2
Prec

NH2-T N H Q G I Y V L Q D N K-COOH

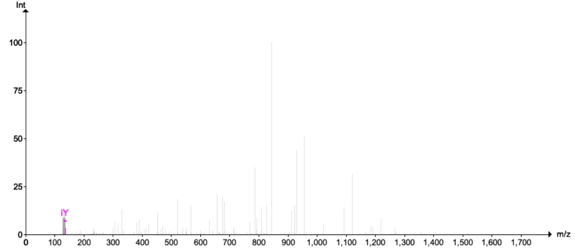

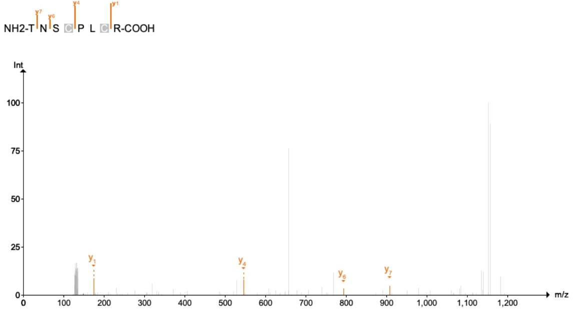
NH2-T N S C P L C R-COOH
y7
y6
y4
y1
Int
100
75
50
25
0
y1
y4
y6
y7
0
100
200
300
400
500
600
700
800
900
1,000
1,100
1,200
m/z

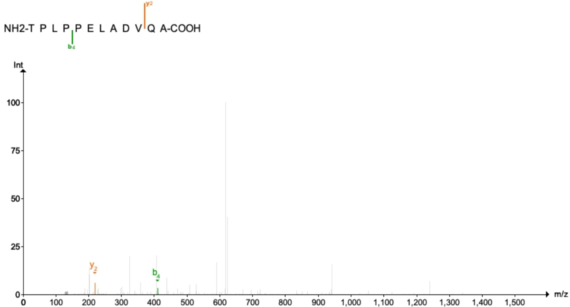
NH2-T P L P P E L A D V Q A-COOH
y2
b4
Int
100
75
50
25
0
0
100
200
300
400
500
600
700
800
900
1,000
1,100
1,200
1,300
1,400
1,500
m/z

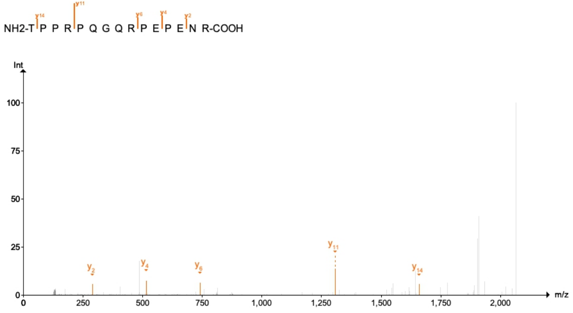

NH2-T P P R P Q G Q R P E P E N R-COOH
y14
y11
y6
y4
y2
Int
100
75
50
25
0
y2
y4
y6
y11
y14
0
250
500
750
1,000
1,250
1,500
1,750
2,000
m/z

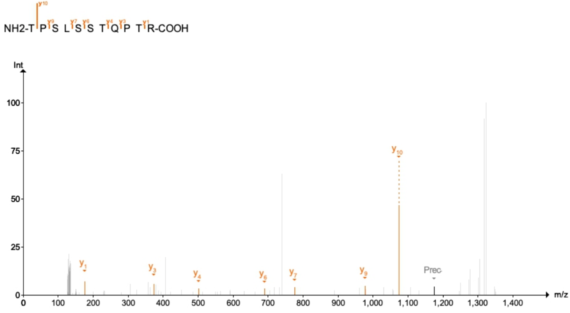
NH2-T P S L S S T Q P T R-COOH
y10
y9
y7
y6
y4
y3
y1
Int
100
75
50
25
0
y10
y1
y3
y4
y6
y7
y9
Prec
0
100
200
300
400
500
600
700
800
900
1,000
1,100
1,200
1,300
1,400
m/z

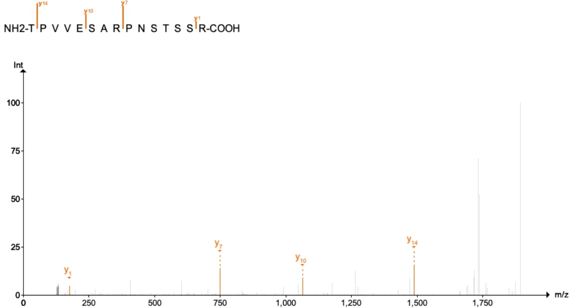

NH2-T P V V E S A R P N S T S S R-COOH
y14
y10
y7
y1
Int
100
75
50
25
0
250
500
750
1,000
1,250
1,500
1,750
m/z
y_1
y_7
y_{10}
y_{14}

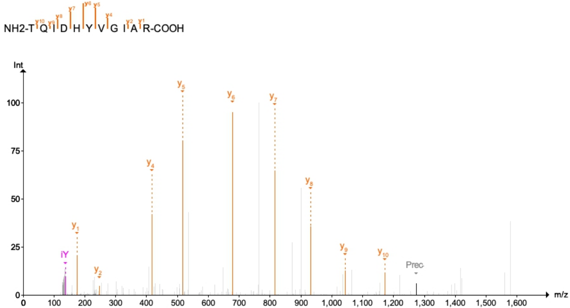

NH2-T Q I D H Y V G I A R-COOH
y10 y9 y8 y7 y6 y5 y4 y2 y1
Int
m/z
y5
y6
y7
y4
y8
y1
y2
y9
y10
iY
Prec

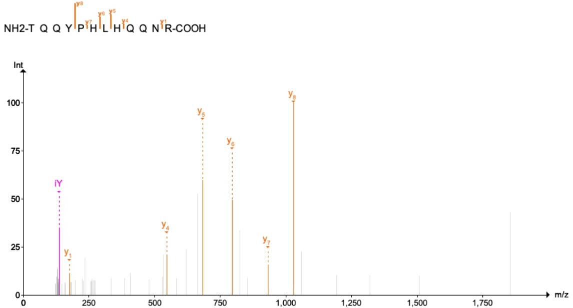

NH2-T Q Q Y P H L H Q Q N R-COOH
y8
y7
y6
y5
y4
y1
Int
iY
y1
y4
y5
y6
y7
y8
m/z
100
75
50
25
0
0
250
500
750
1,000
1,250
1,500
1,750

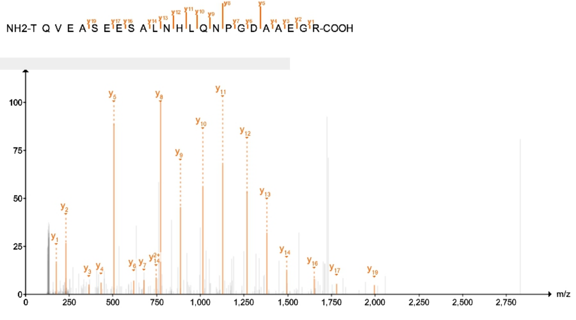
NH2-T Q V E A S E E S A L N H L Q N P G D A A E G R-COOH
y1
y2
y3
y4
y5
y6
y7
y8
y9
y10
y11
y12
y13
y14
y16
y17
y19
y2+ 14
100
75
50
25
0
250
500
750
1,000
1,250
1,500
1,750
2,000
2,250
2,500
2,750
m/z

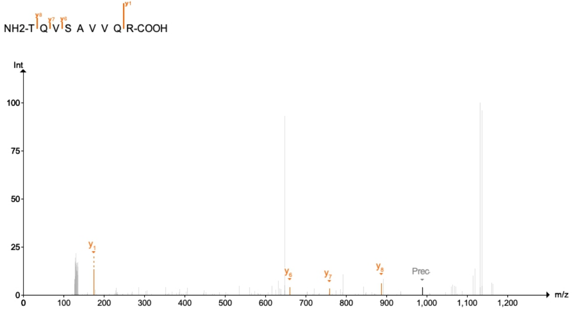
NH2-T Q V S A V V Q R-COOH
y8
y7
y6
y1
Int
100
75
50
25
0
y1
y6
y7
y8
Prec
0
100
200
300
400
500
600
700
800
900
1,000
1,100
1,200
m/z

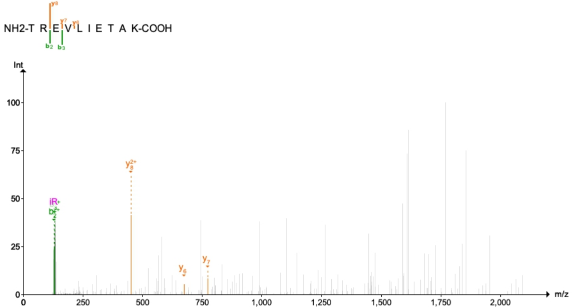

NH2-T R E V L I E T A K-COOH
y8
y7
y6
b2
b3
Int
100
75
50
25
0
y_8^{2+}
iR+
b_2^{+}
y_6
y_7
0
250
500
750
1,000
1,250
1,500
1,750
2,000
m/z

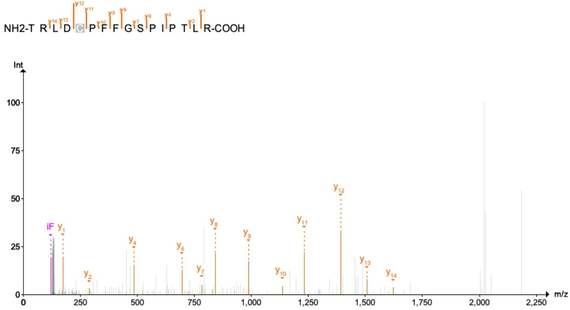

NH2-T R L D C P F F G S P I P T L R-COOH
Int
m/z
iF
y1
y2
y4
y6
y7
y8
y9
y10
y11
y12
y13
y14

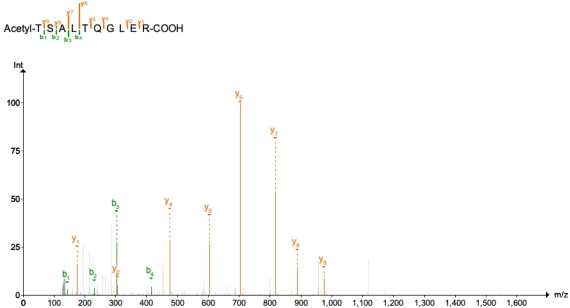

Acetyl-T S A L T Q G L E R-COOH
y9 y8 y7 y6 y5 y4 y2 y1
b1 b2 b3 b4
Int
m/z
y6
y7
b3
y4
y5
y1
y8
y9
b1
b2
y2
b4

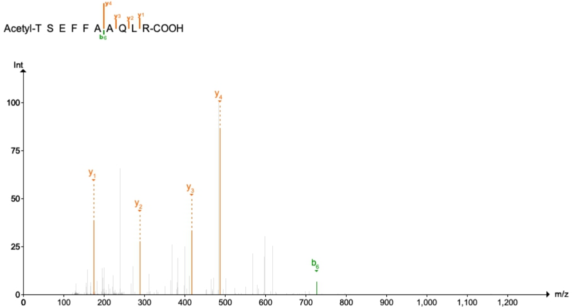
Acetyl-T S E F F A A Q L R-COOH
y4
y3
y2
y1
b6
Int
100
75
50
25
0
y1
y2
y3
y4
b6
0
100
200
300
400
500
600
700
800
900
1,000
1,100
1,200
m/z

NH2-T S L E D E E V F E Q K-COOH

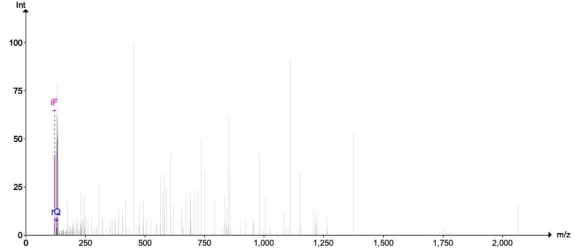

NH2-T S L E G Y P A G G S L P Y S I Q T A E K-COOH

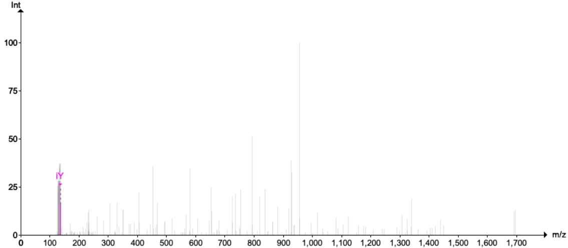

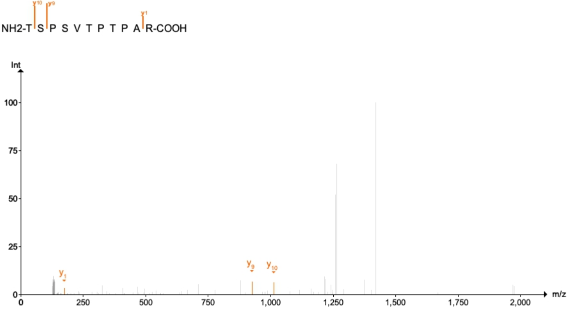

y10
y9
y1
NH2-T S P S V T P T P A R-COOH
Int
100
75
50
25
0
0
250
500
750
1,000
1,250
1,500
1,750
2,000
m/z
y1
y9
y10

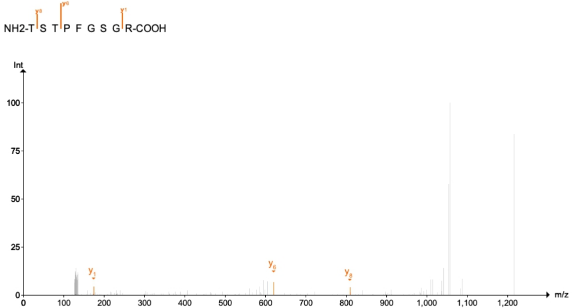

y8
y6
y1
NH2-T S T P F G S G R-COOH
Int
100
75
50
25
0
y1
y6
y8
0
100
200
300
400
500
600
700
800
900
1,000
1,100
1,200
m/z

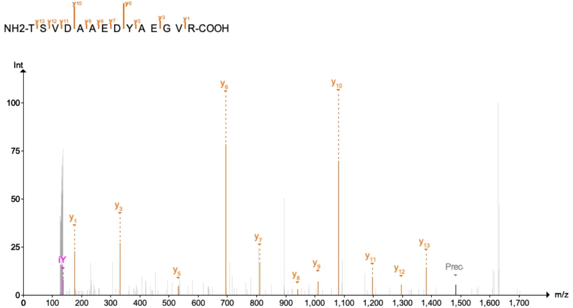

NH2-T S V D A A E D Y A E G V R-COOH
y13 y12 y11 y10 y9 y8 y7 y6 y5 y3 y1
Int
100
75
50
25
0
y1
y3
y5
y6
y7
y8
y9
y10
y11
y12
y13
iY
Prec
0 100 200 300 400 500 600 700 800 900 1,000 1,100 1,200 1,300 1,400 1,500 1,600 1,700
m/z

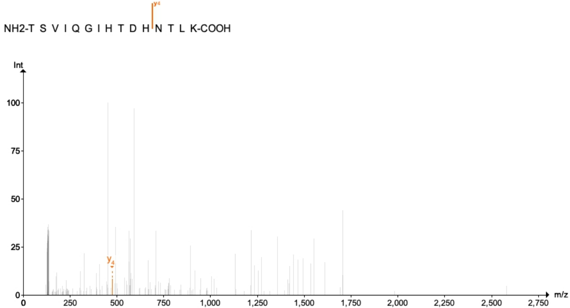

y4
NH2-T S V I Q G I H T D H N T L K-COOH
Int
100
75
50
25
0
y4
0
250
500
750
1,000
1,250
1,500
1,750
2,000
2,250
2,500
2,750
m/z

NH2-T T A A F Q Q G K-COOH

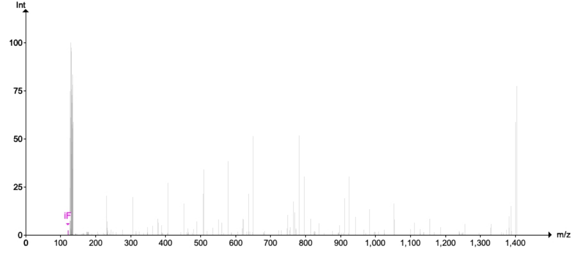

NH2-T T G F Y S G F S E V A E K-COOH

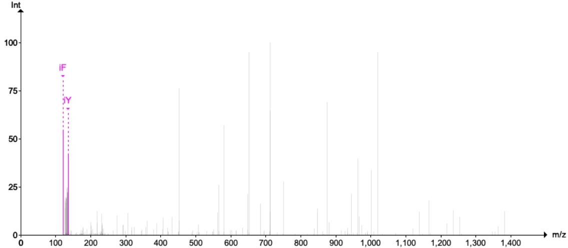

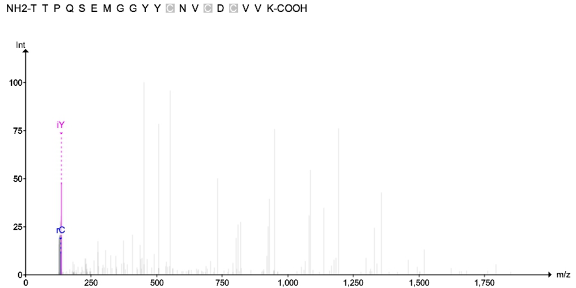
NH2-T T P Q S E M G G Y Y C N V C D C V V K-COOH
Int
100
75
50
25
0
iY
rC
0
250
500
750
1,000
1,250
1,500
1,750
m/z

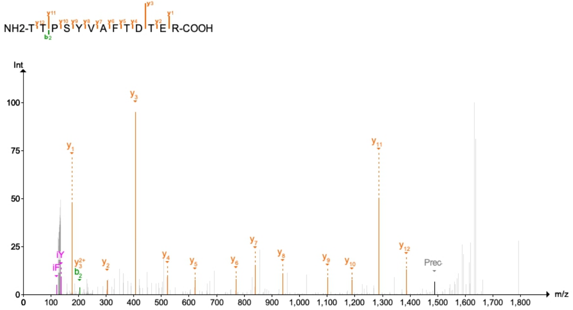

NH2-T T P S Y V A F T D T E R-COOH
Int
m/z
Prec

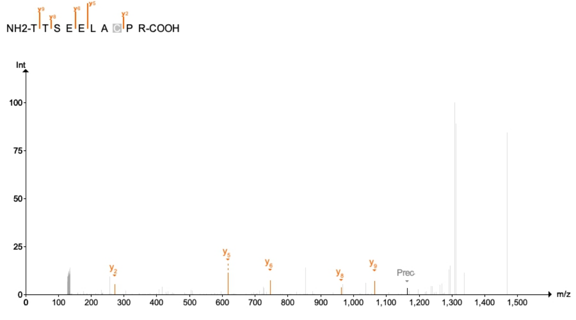
NH2-T T S E E L A C P R-COOH
y9
y8
y6
y5
y2
Int
100
75
50
25
0
y2
y5
y6
y8
y9
Prec
0
100
200
300
400
500
600
700
800
900
1,000
1,100
1,200
1,300
1,400
1,500
m/z

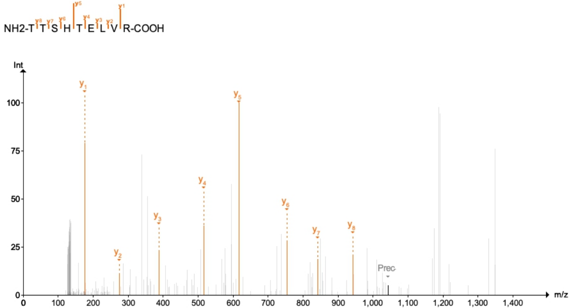
NH2-T T S H T E L V R-COOH
y8 y7 y6 y5 y4 y3 y2 y1
Int
100
75
50
25
0
y1
y2
y3
y4
y5
y6
y7
y8
Prec
0 100 200 300 400 500 600 700 800 900 1,000 1,100 1,200 1,300 1,400
m/z

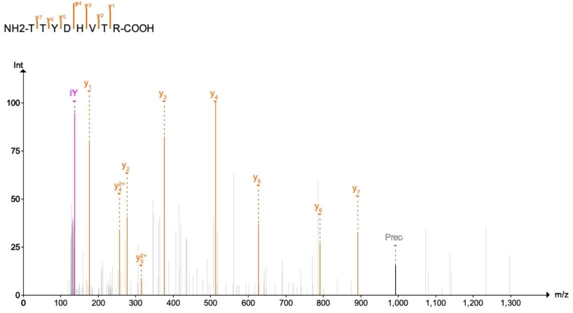
NH2-T T Y D H V T R-COOH
y7 y6 y5 y4 y3 y2 y1
Int
IY
y1
y3
y4
y2
y4 2+
y5
y6
y7
y5 2+
Prec
100
75
50
25
0
0
100
200
300
400
500
600
700
800
900
1,000
1,100
1,200
1,300
m/z

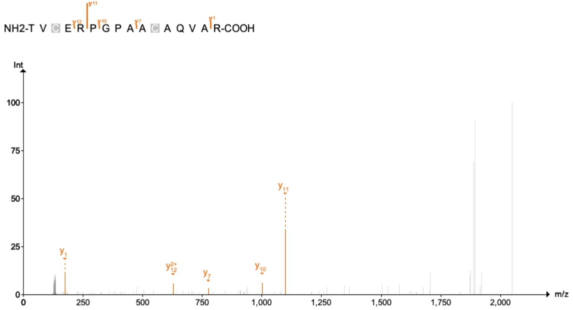

NH2-T V C E R P G P A A C A Q V A R-COOH
y11
y12
y10
y7
y1
Int
m/z
y1
y12 2+
y7
y10
y11

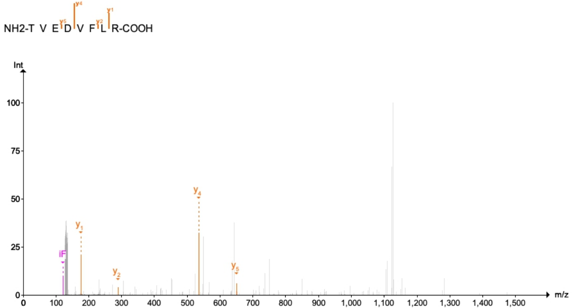
NH2-T V E D V F L R-COOH
y5
y4
y2
y1
Int
100
75
50
25
0
iF
m/z
0
100
200
300
400
500
600
700
800
900
1,000
1,100
1,200
1,300
1,400
1,500

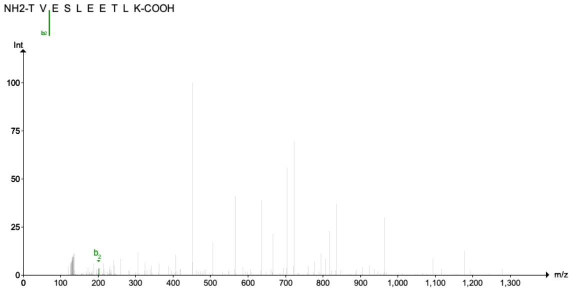
NH2-T V E S L E E T L K-COOH
b2
Int
100
75
50
25
0
b2
0
100
200
300
400
500
600
700
800
900
1,000
1,100
1,200
1,300
m/z

NH2-T V F S A D P N V V D L H K-COOH

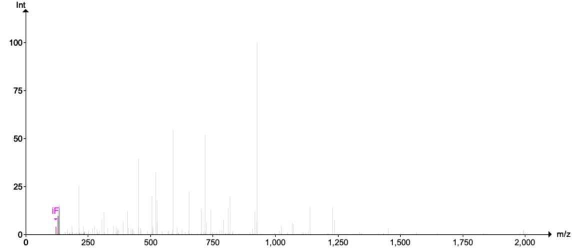

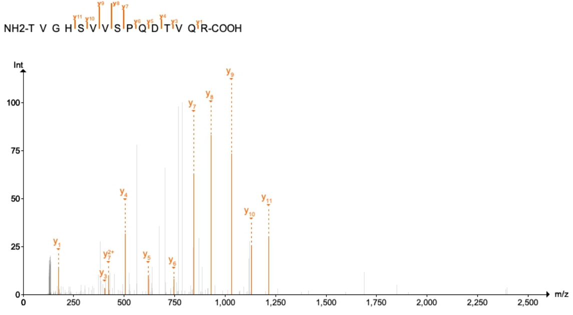

NH2-T V G H S V V S P Q D T V Q R-COOH
y11 y10 y9 y8 y7 y6 y5 y4 y3 y1
Int
100
75
50
25
0
y1 y3 y7 2+ y4 y5 y6 y7 y8 y9 y10 y11
0 250 500 750 1,000 1,250 1,500 1,750 2,000 2,250 2,500
m/z

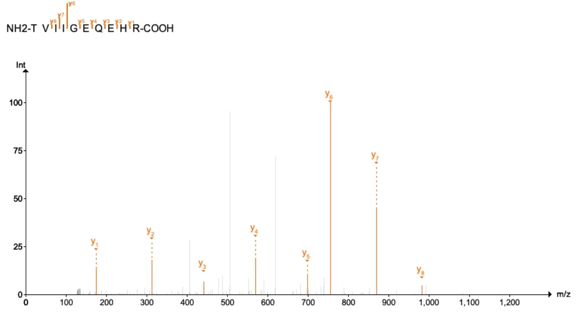
NH2-T V I I G E Q E H R-COOH
y8
y7
y6
y5
y4
y3
y2
y1
Int
100
75
50
25
0
y1
y2
y3
y4
y5
y6
y7
y8
0
100
200
300
400
500
600
700
800
900
1,000
1,100
1,200
m/z

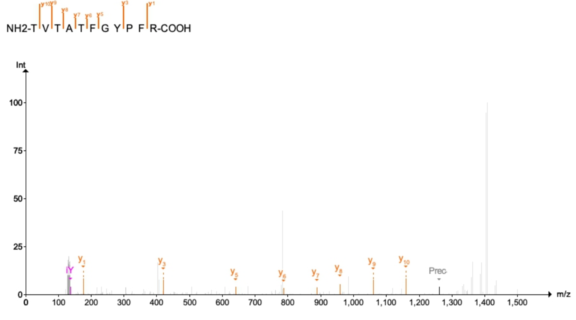

NH2-T V T A T F G Y P F R-COOH
y10
y9
y8
y7
y6
y5
y3
y1
Int
m/z
IY
Prec
0
25
50
75
100
100
200
300
400
500
600
700
800
900
1,000
1,100
1,200
1,300
1,400
1,500

NH2-T W V A S N E I K-COOH

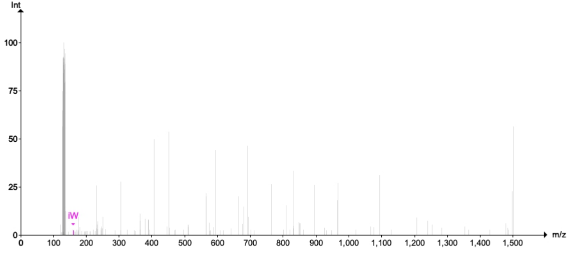

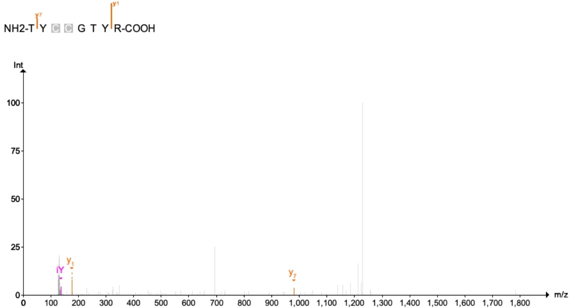

NH2-T Y C C G T Y R-COOH
y7
y1
Int
100
75
50
25
0
m/z
0
100
200
300
400
500
600
700
800
900
1,000
1,100
1,200
1,300
1,400
1,500
1,600
1,700
1,800
IY
y1
y7

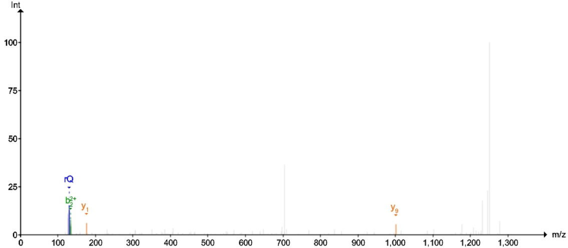

Int
100
75
50
25
0
rQ
b_2^{2+}
y_1
y_9
0
100
200
300
400
500
600
700
800
900
1,000
1,100
1,200
1,300
m/z

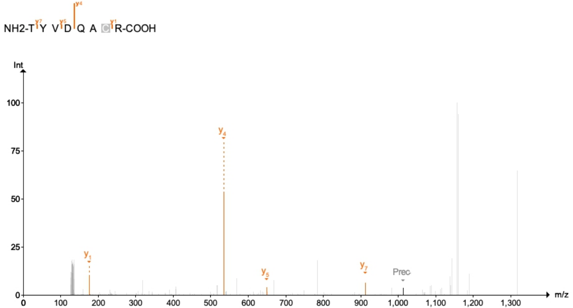
NH2-TYVDQACR-COOH
y7
y5
y4
y1
Int
100
75
50
25
0
y1
y4
y5
y7
Prec
0
100
200
300
400
500
600
700
800
900
1,000
1,100
1,200
1,300
m/z

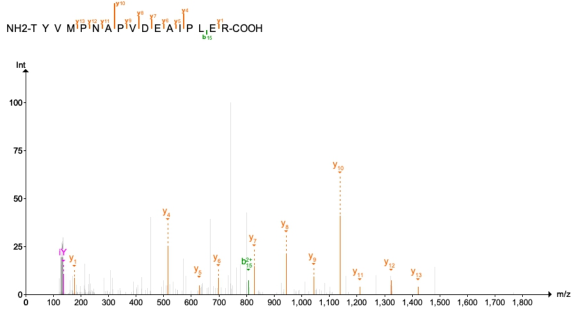
NH2-T Y V M P N A P V D E A I P L E R-COOH
y13 y12 y11 y10 y9 y8 y7 y6 y5 y4 y1
b15
Int
100
75
50
25
0
iY
y1
y4
y5
y6
b2+ 15
y7
y8
y9
y10
y11
y12
y13
m/z
0 100 200 300 400 500 600 700 800 900 1,000 1,100 1,200 1,300 1,400 1,500 1,600 1,700 1,800

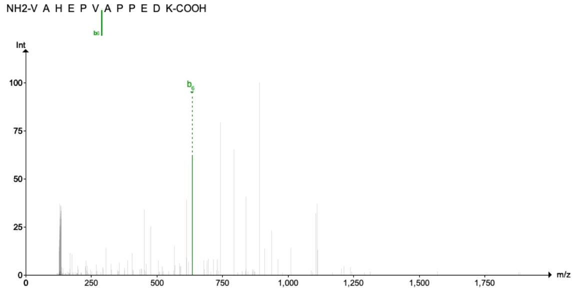
NH2-V A H E P V A P P E D K-COOH
b6
Int
100
75
50
25
0
b6
0
250
500
750
1,000
1,250
1,500
1,750
m/z

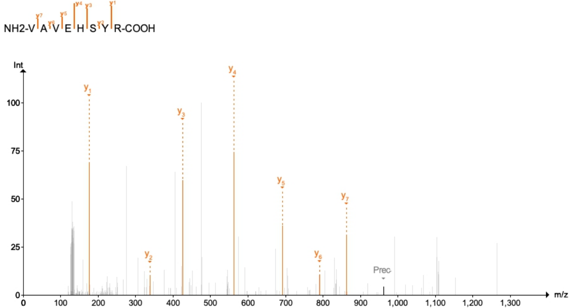
NH2-VAVEHSYR-COOH
y7
y6
y5
y4
y3
y2
y1
Int
100
75
50
25
0
y1
y2
y3
y4
y5
y6
y7
Prec
0
100
200
300
400
500
600
700
800
900
1,000
1,100
1,200
1,300
m/z

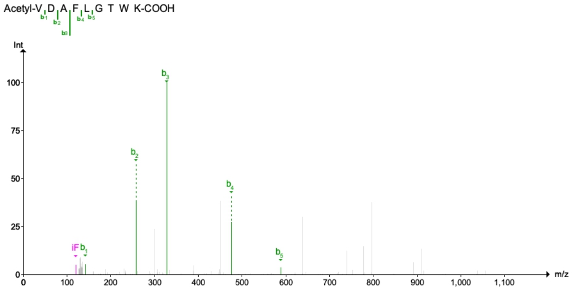
Acetyl-V D A F L G T W K-COOH
b1
b2
b3
b4
b5
Int
100
75
50
25
0
iF
m/z
100
200
300
400
500
600
700
800
900
1,000
1,100

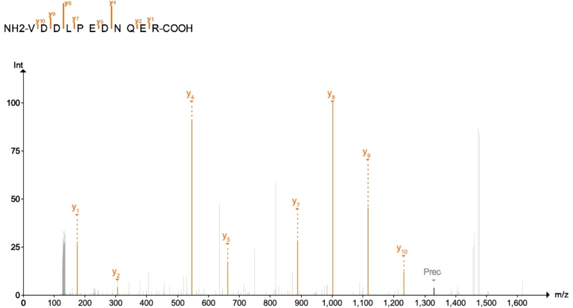

NH2-V D D L P E D N Q E R-COOH
y10
y9
y8
y7
y5
y4
y2
y1
Int
100
75
50
25
0
y1
y2
y4
y5
y7
y8
y9
y10
Prec
0
100
200
300
400
500
600
700
800
900
1,000
1,100
1,200
1,300
1,400
1,500
1,600
m/z

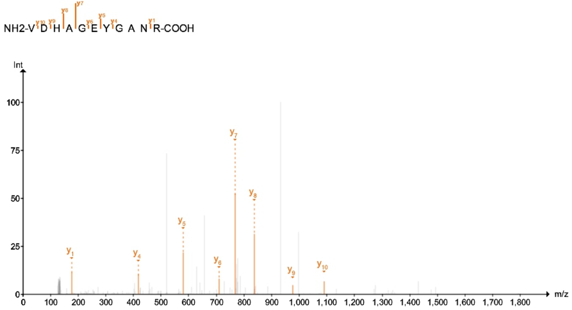
NH2-V D H A G E Y G A N R-COOH
y10
y9
y8
y7
y6
y5
y4
y1
Int
m/z
0
25
50
75
100
0
100
200
300
400
500
600
700
800
900
1,000
1,100
1,200
1,300
1,400
1,500
1,600
1,700
1,800

NH2-V E H S D L S F S K-COOH

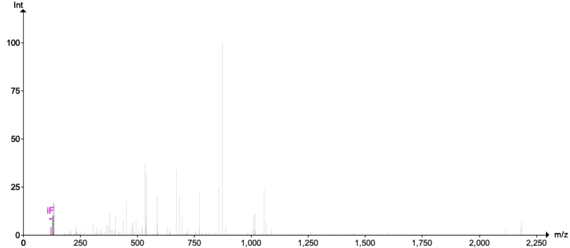

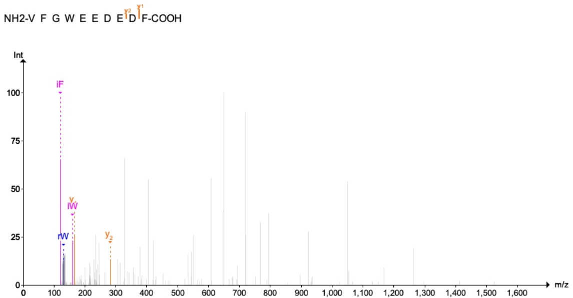

NH2-V F G W E E D E D F-COOH
y2
y1
Int
iF
y1
iW
rW
y2
m/z

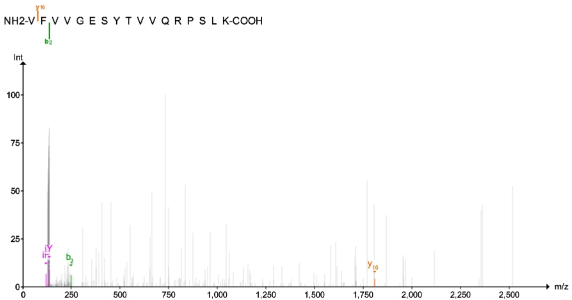

NH2-V F V V G E S Y T V V Q R P S L K-COOH
y16
b2
Int
100
75
50
25
0
iY
iF
b2
y16
0
250
500
750
1,000
1,250
1,500
1,750
2,000
2,250
2,500
m/z

NH2-V G E I F S A A G A A F T K-COOH

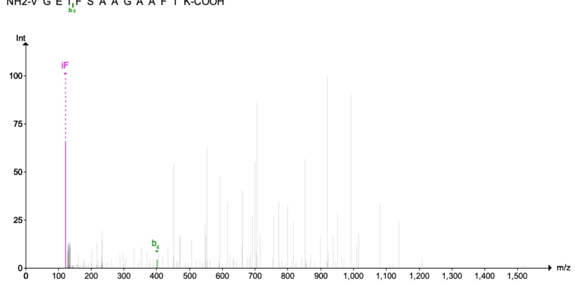

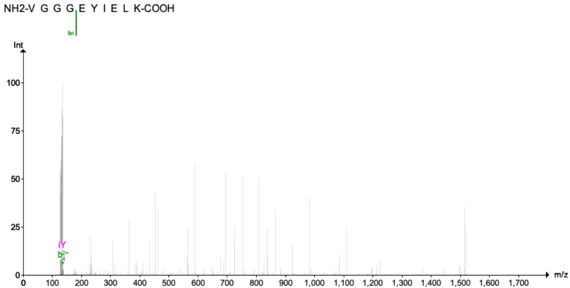

NH2-V G G G E Y I E L K-COOH
b4
Int
100
75
50
25
0
iY
b_4^{2+}
0 100 200 300 400 500 600 700 800 900 1,000 1,100 1,200 1,300 1,400 1,500 1,600 1,700
m/z

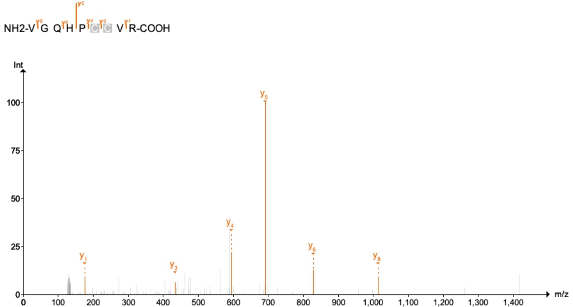

NH2-V G Q H P C C V R-COOH
Int
m/z
y1
y3
y4
y5
y6
y8
0
25
50
75
100
0
100
200
300
400
500
600
700
800
900
1,000
1,100
1,200
1,300
1,400

NH2-V H F P D P N K-COOH

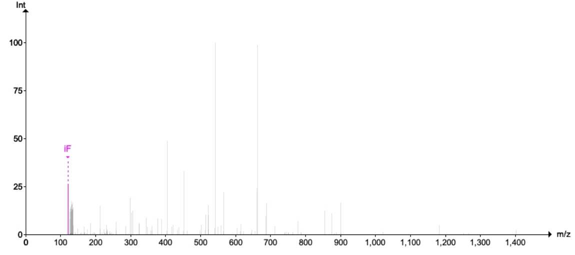

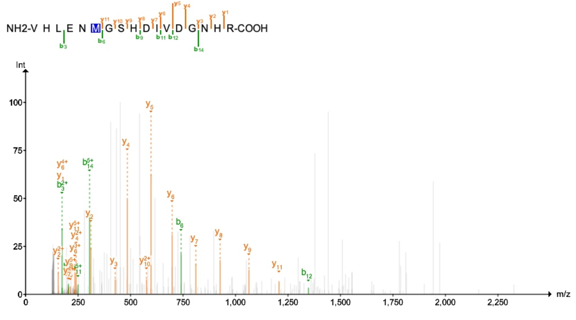

NH2-V H L E N M G S H D I V D G N H R-COOH
Int
m/z

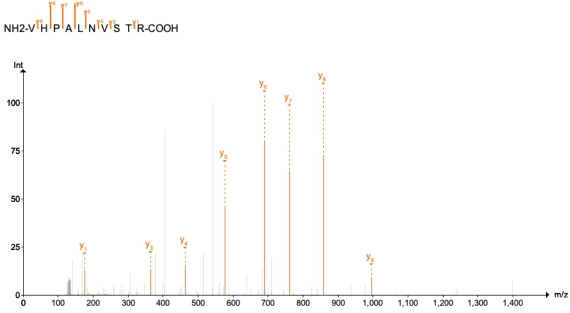
NH2-V H P A L N V S T R-COOH
y9 y8 y7 y6 y5 y4 y3 y1
Int
m/z
y1 y3 y4 y5 y6 y7 y8 y9
0 25 50 75 100
0 100 200 300 400 500 600 700 800 900 1,000 1,100 1,200 1,300 1,400

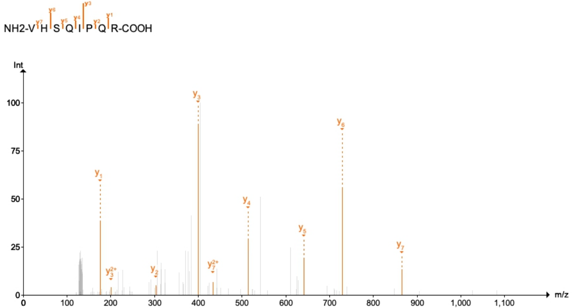
NH2-V H S Q I P Q R-COOH
y7 y6 y5 y4 y3 y2 y1
Int
100
75
50
25
0
y3
y6
y1
y4
y5
y7
y3 2+
y2
y7 2+
0 100 200 300 400 500 600 700 800 900 1,000 1,100
m/z

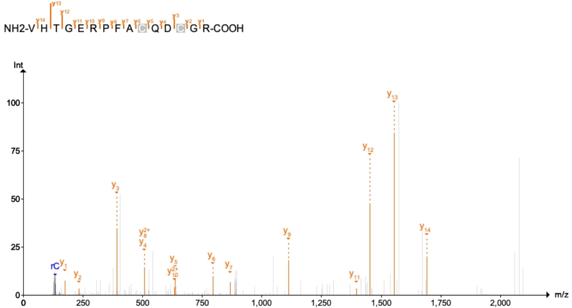
NH2-V H T G E R P F A C Q D C G R-COOH
y14 y13 y12 y11 y10 y9 y8 y7 y6 y5 y4 y3 y2 y1
Int
m/z
rC
y1
y2
y3
y4
y8 2+
y5
y10 2+
y6
y7
y9
y11
y12
y13
y14
0
25
50
75
100
250
500
750
1,000
1,250
1,500
1,750
2,000

NH2-V H T G E R P Y K-COOH

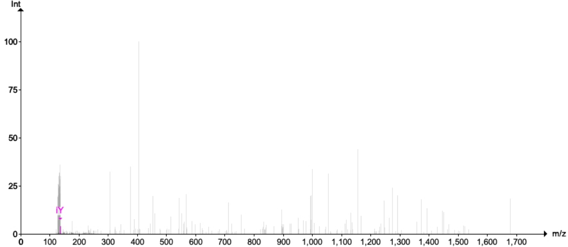

NH2-V I T I E S Q K-COOH

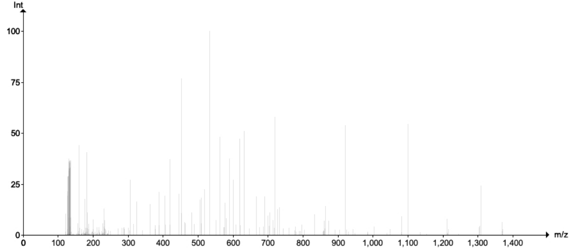

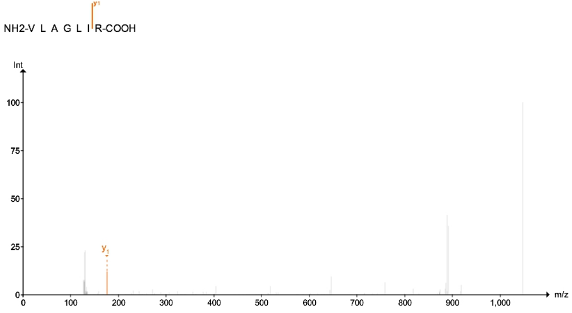

NH2-V L A G L I R-COOH
y1
Int
100
75
50
25
0
y1
0
100
200
300
400
500
600
700
800
900
1,000
m/z

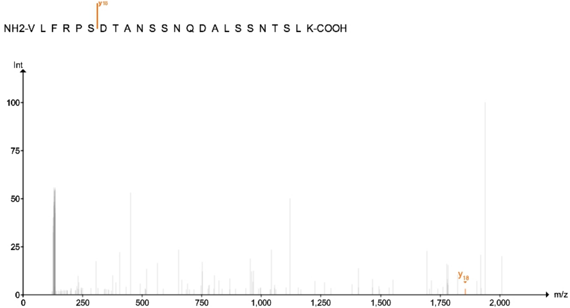

y18
NH2-V L F R P S D T A N S S N Q D A L S S N T S L K-COOH
Int
100
75
50
25
0
0
250
500
750
1,000
1,250
1,500
1,750
2,000
m/z
y18

NH2-V L F T S L A D N K-COOH

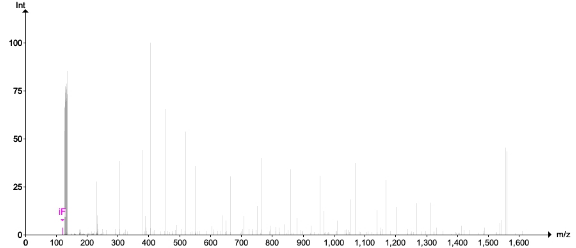

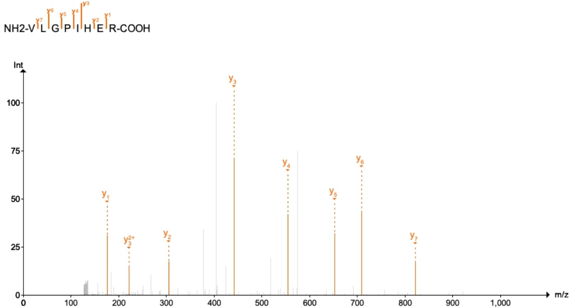

NH2-V L G P I H E R-COOH
y7
y6
y5
y4
y3
y2
y1
Int
100
75
50
25
0
y3
y4
y6
y5
y1
y2
y7
y3 2+
m/z
0
100
200
300
400
500
600
700
800
900
1,000

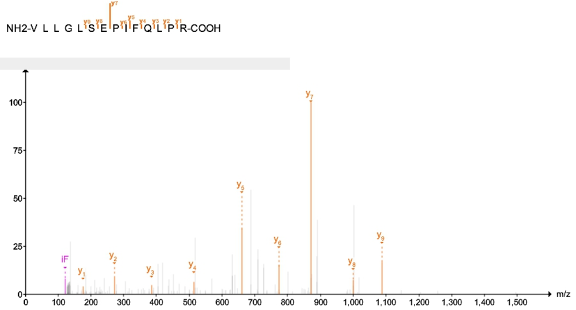
NH2-V L L G L S E P I F Q L P R-COOH
y9 y8 y7 y6 y5 y4 y3 y2 y1
iF
y1
y2
y3
y4
y5
y6
y7
y8
y9
0
25
50
75
100
0 100 200 300 400 500 600 700 800 900 1,000 1,100 1,200 1,300 1,400 1,500
m/z

NH2-V L L H L E E K-COOH

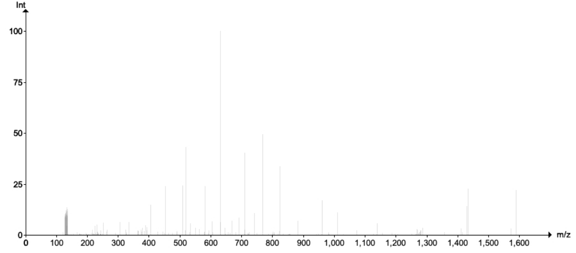

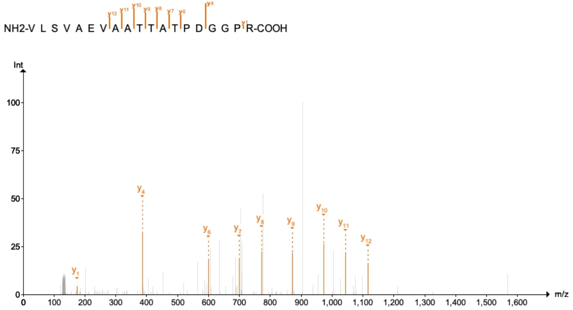

NH2-V L S V A E V A A T T A T P D G G P R-COOH
y12
y11
y10
y9
y8
y7
y6
y4
y1
Int
100
75
50
25
0
m/z
0
100
200
300
400
500
600
700
800
900
1,000
1,100
1,200
1,300
1,400
1,500
1,600

NH2-V L V N V L T S D Q T E D V L E A V A A K-COOH

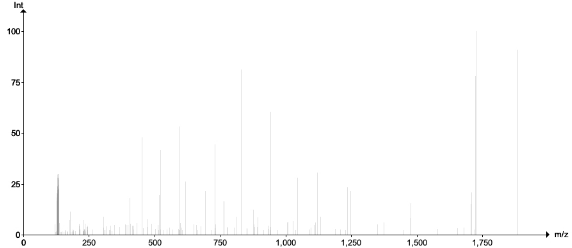

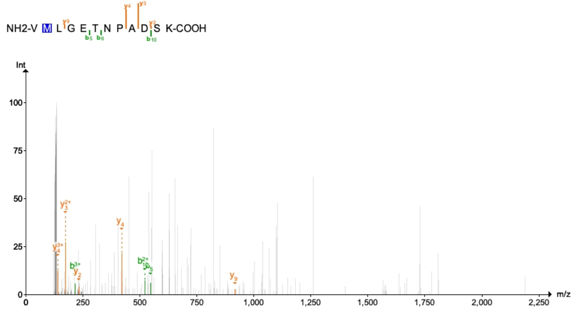
NH2-V M L G E T N P A D S K-COOH
Int
m/z
0
25
50
75
100
250
500
750
1,000
1,250
1,500
1,750
2,000
2,250
y4
y3
y9
y2
b5
b6
b10
b3+
b2+ 10

NH2-V M Q E E I F G P I L P I V P V K-COOH

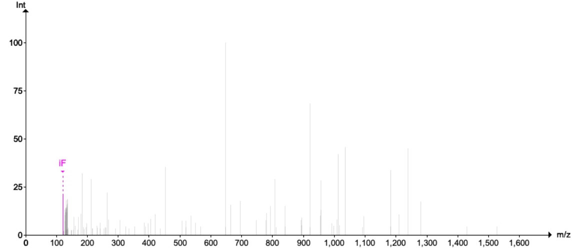

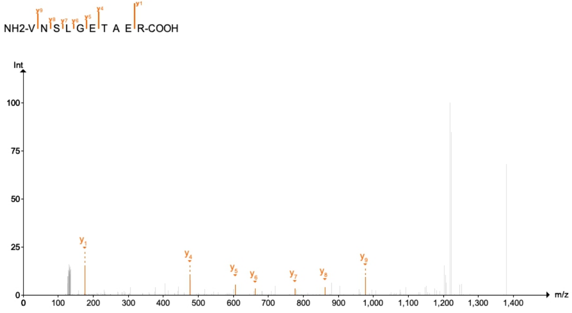

NH2-V N S L G E T A E R-COOH
y9
y8
y7
y6
y5
y4
y1
Int
100
75
50
25
0
y1
y4
y5
y6
y7
y8
y9
m/z
0
100
200
300
400
500
600
700
800
900
1,000
1,100
1,200
1,300
1,400

NH2-V N V C E H C L V A N H A K-COOH

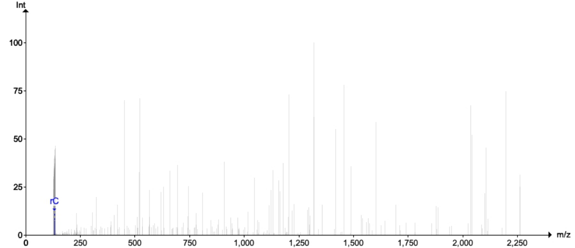

NH2-V P F L V L E C P N L K-COOH

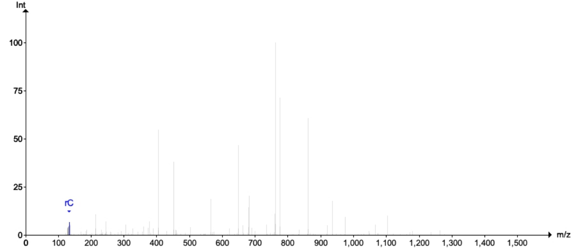

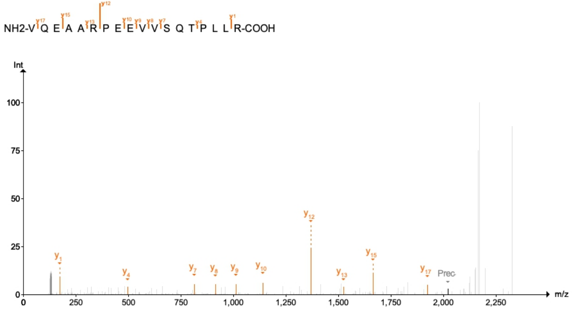
NH2-V Q E A A R P E E V V S Q T P L L R-COOH
y17
y15
y13
y12
y10
y9
y8
y7
y4
y1
Int
100
75
50
25
0
250
500
750
1,000
1,250
1,500
1,750
2,000
2,250
m/z
y1
y4
y7
y8
y9
y10
y12
y13
y15
y17
Prec

NH2-V Q N I H P V E S A K-COOH

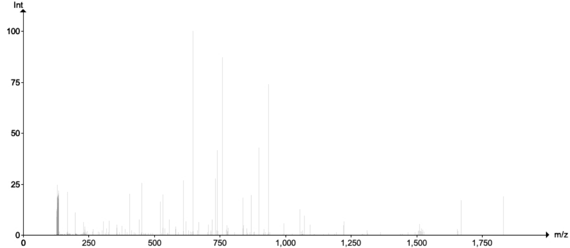

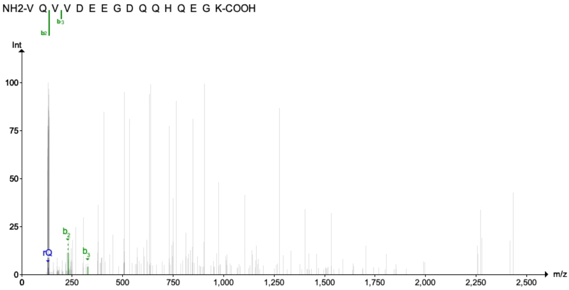
NH2-V Q V V D E E G D Q Q H Q E G K-COOH
b2
b3
Int
100
75
50
25
0
rQ
b2
b3
0
250
500
750
1,000
1,250
1,500
1,750
2,000
2,250
2,500
m/z

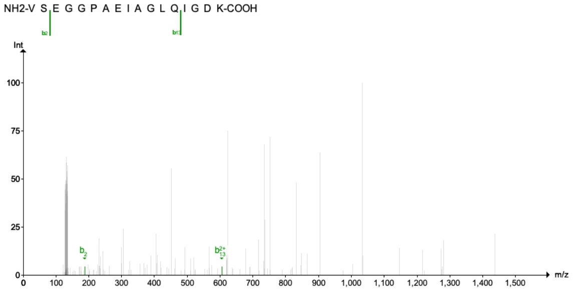
NH2-V S E G G P A E I A G L Q I G D K-COOH
b2
b13
Int
100
75
50
25
0
b2
b13 2+
0
100
200
300
400
500
600
700
800
900
1,000
1,100
1,200
1,300
1,400
1,500
m/z

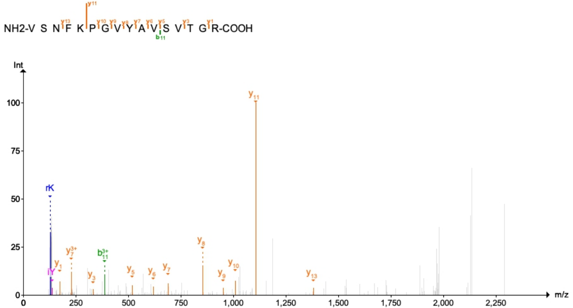

NH2-V S N F K P G V Y A V S V T G R-COOH
y11
y13
y10
y9
y8
y7
y6
y5
y3
y1
b11
Int
100
75
50
25
0
rK
iY
y1
y7 3+
b11 3+
y3
y5
y6
y7
y8
y9
y10
y11
y13
0
250
500
750
1,000
1,250
1,500
1,750
2,000
2,250
m/z

NH2-V S S A E G A A K E E P K-COOH

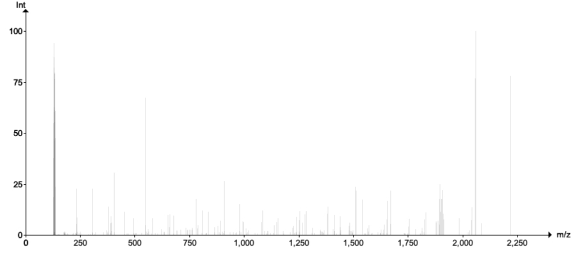

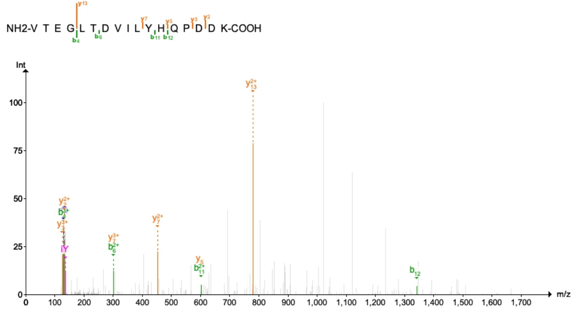

NH2-V T E G L T D V I L Y H Q P D D K-COOH
y13
y7
y5
y3
y2
b4
b6
b11
b12
Int
m/z
y13 2+
y2 2+
b3 2+
y3 3+
iY
y7 2+
y7 3+
b6 2+
y5
b11 2+
b12

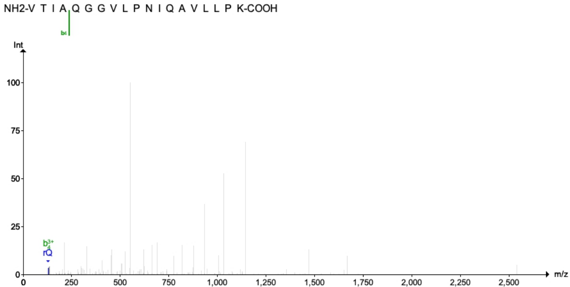
NH2-V T I A Q G G V L P N I Q A V L L P K-COOH
b4
Int
100
75
50
25
0
b$_4^{3+}$
rQ
0
250
500
750
1,000
1,250
1,500
1,750
2,000
2,250
2,500
m/z

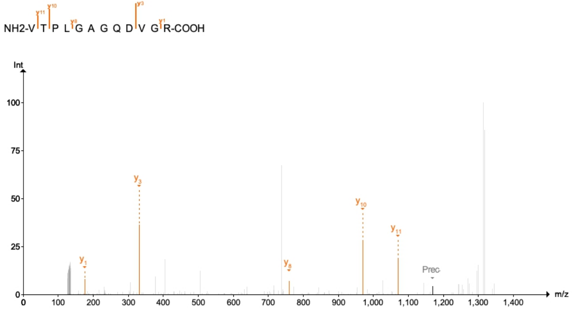

NH2-V T P L G A G Q D V G R-COOH
y11
y10
y8
y3
y1
Int
100
75
50
25
0
y3
y10
y11
y1
y8
Prec
0
100
200
300
400
500
600
700
800
900
1,000
1,100
1,200
1,300
1,400
m/z

NH2-V T V D N Q G E K P V L V K-COOH

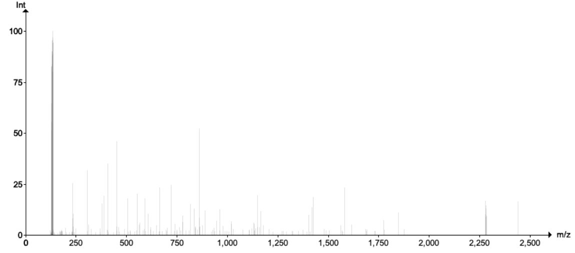

NH2-V V A V Y G T L S D L L S V A S S K-COOH

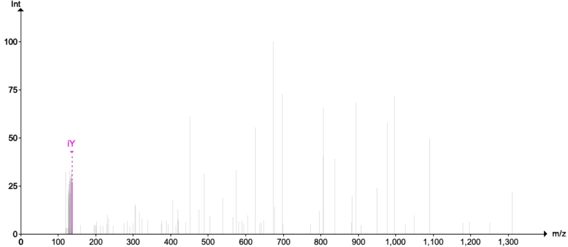

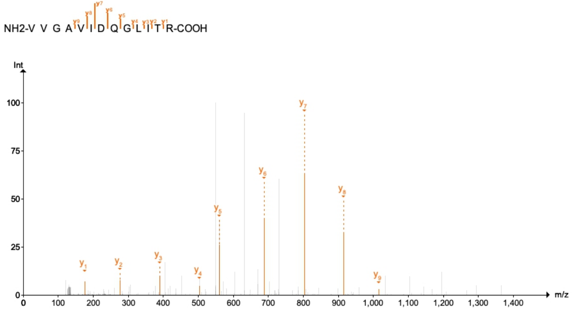

NH2-V V G A V I D Q G L I T R-COOH
Int
m/z
y1
y2
y3
y4
y5
y6
y7
y8
y9
0
25
50
75
100
100
200
300
400
500
600
700
800
900
1,000
1,100
1,200
1,300
1,400

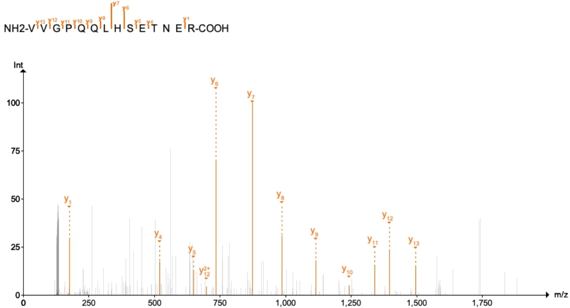

NH2-V V G P Q Q L H S E T N E R-COOH
y13 y12 y11 y10 y9 y8 y7 y6 y5 y4 y1
Int
100
75
50
25
0
y1
y4
y5
y2+12
y6
y7
y8
y9
y10
y11
y12
y13
0
250
500
750
1,000
1,250
1,500
1,750
m/z

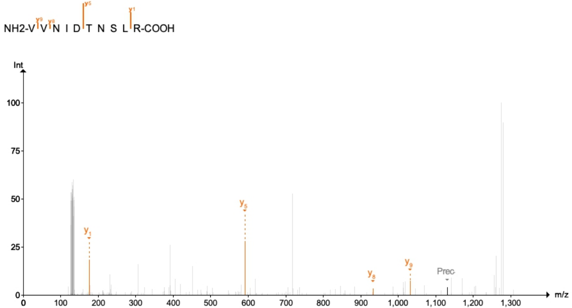

NH2-V V N I D T N S L R-COOH
y9
y8
y5
y1
Int
100
75
50
25
0
m/z
0
100
200
300
400
500
600
700
800
900
1,000
1,100
1,200
1,300
y1
y5
y8
y9
Prec

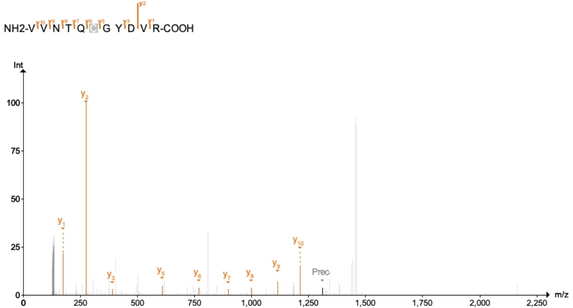
NH2-V V N T Q C G Y D V R-COOH
y10 y9 y8 y7 y6 y5 y3 y2 y1
Int
100
75
50
25
0
y2
y1
y3
y5
y6
y7
y8
y9
y10
Prec
0
250
500
750
1,000
1,250
1,500
1,750
2,000
2,250
m/z

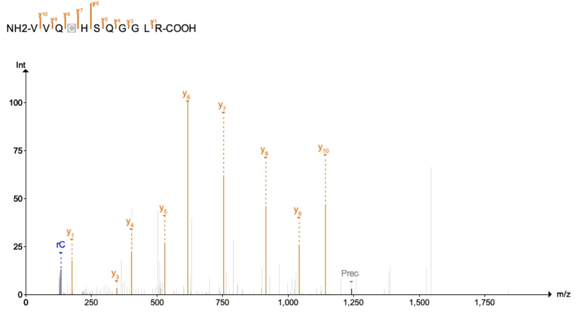
NH2-V V Q C H S Q G G L R-COOH
y10 y9 y8 y7 y6 y5 y4 y3 y1
Int
m/z
rC
Prec
0
25
50
75
100
250
500
750
1,000
1,250
1,500
1,750

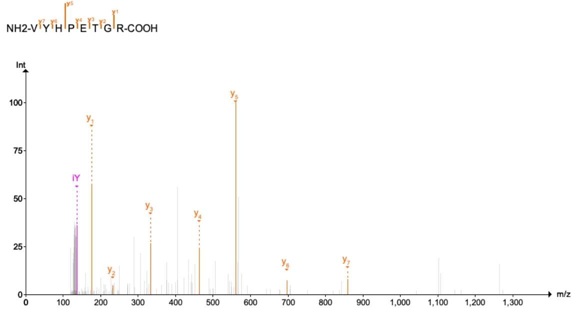

NH2-VYHPETGR-COOH
Int
100
75
50
25
0
y1
y2
y3
y4
y5
y6
y7
IY
0
100
200
300
400
500
600
700
800
900
1,000
1,100
1,200
1,300
m/z

NH2-W F V P W G P N H C D K-COOH

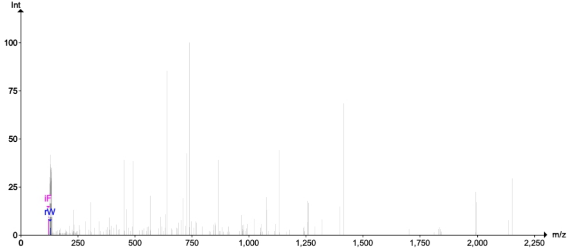

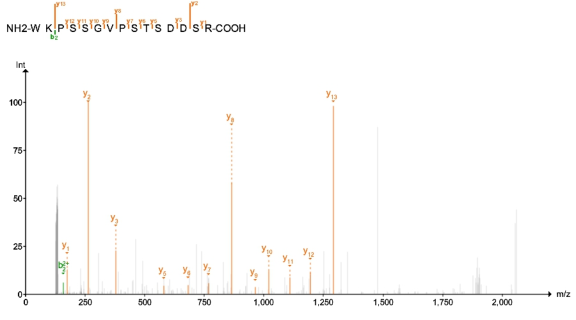

NH2-W K P S S G V P S T S D D S R-COOH
y13
y12
y11
y10
y9
y8
y7
y6
y5
y3
y2
y1
b2
Int
100
75
50
25
0
y2
y13
y8
y3
y1
b2 2+
y5
y6
y7
y9
y10
y11
y12
0
250
500
750
1,000
1,250
1,500
1,750
2,000
m/z

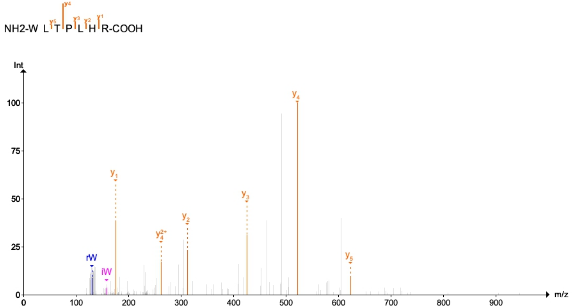
NH2-W L T P L H R-COOH
y5
y4
y3
y2
y1
Int
m/z
0
25
50
75
100
100
200
300
400
500
600
700
800
900
y1
y4 2+
y2
y3
y4
y5
rW
iW

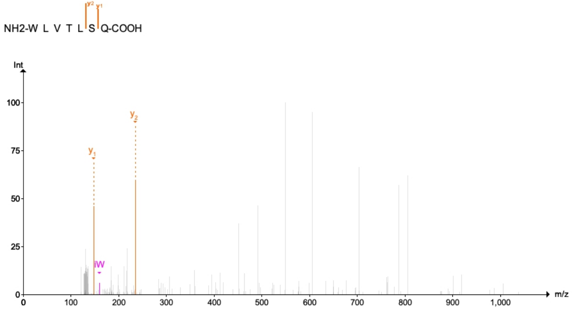

NH2-W L V T L S Q-COOH
y2
y1
Int
100
75
50
25
0
y1
y2
iW
0
100
200
300
400
500
600
700
800
900
1,000
m/z

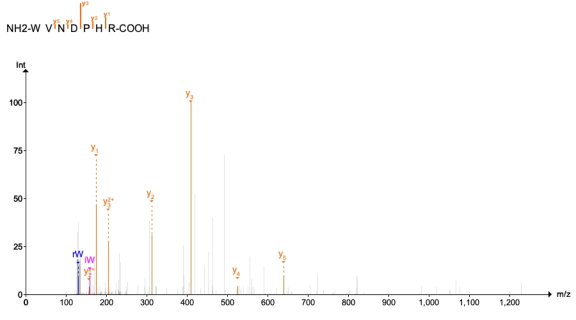
NH2-W V N D P H R-COOH
y5
y4
y3
y2
y1
Int
m/z
rW
iW
y1
y2
y3
y4
y5
y3 2+
0
25
50
75
100
100
200
300
400
500
600
700
800
900
1,000
1,100
1,200

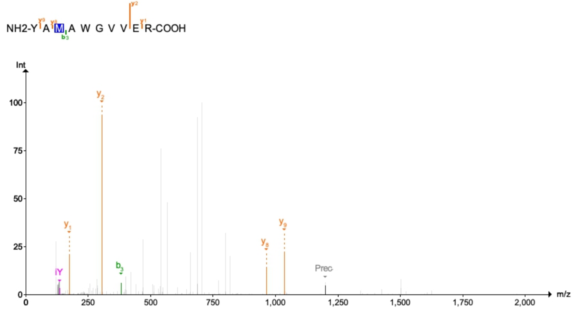

NH2-Y A M A W G V V E R-COOH
y9
y8
b3
y2
y1
Int
100
75
50
25
0
y2
y1
iY
b3
y8
y9
Prec
250
500
750
1,000
1,250
1,500
1,750
2,000
m/z

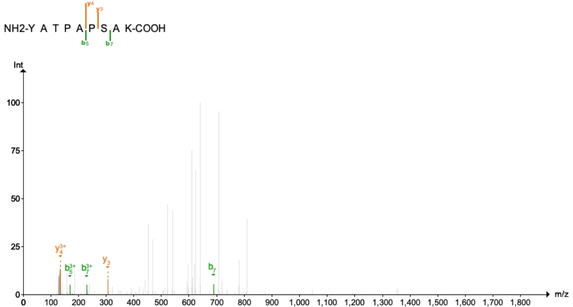
NH2-Y A T P A P S A K-COOH
y4
y3
b5
b7
Int
100
75
50
25
0
y4 3+
b5 3+
b7 3+
y3
b7
0
100
200
300
400
500
600
700
800
900
1,000
1,100
1,200
1,300
1,400
1,500
1,600
1,700
1,800
m/z

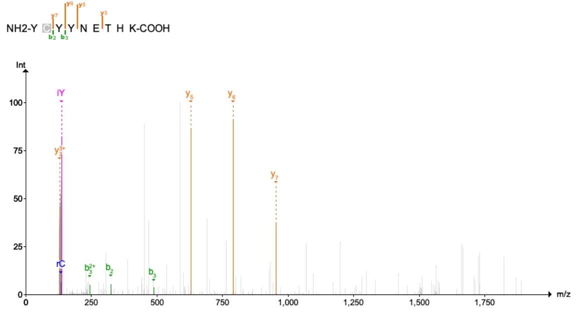
NH2-Y C Y Y N E T H K-COOH
y7
y6
y5
y3
b2
b3
Int
m/z
iY
y5
y6
y3 3+
y7
rC
b3 2+
b2
b3
100
75
50
25
0
0
250
500
750
1,000
1,250
1,500
1,750

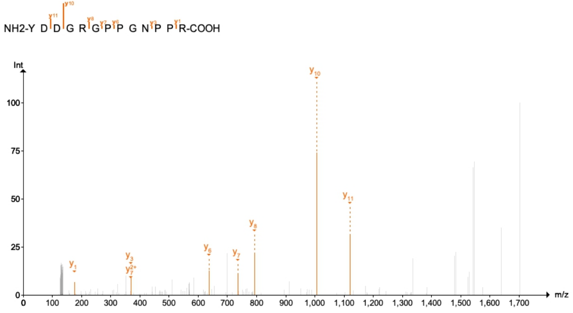

NH2-Y D D G R G P P G N P P R-COOH
y11
y10
y8
y7
y6
y3
y1
Int
m/z
y10
y11
y8
y6
y7
y3
y7 2+
y1
0
25
50
75
100
0
100
200
300
400
500
600
700
800
900
1,000
1,100
1,200
1,300
1,400
1,500
1,600
1,700

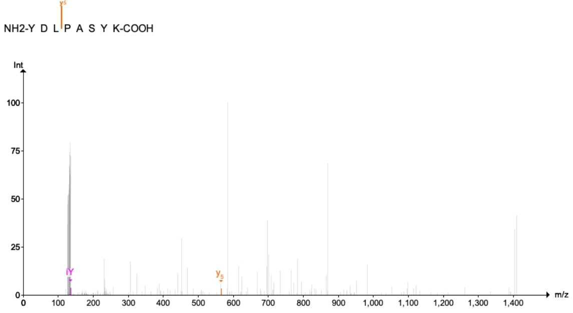

y5
NH2-Y D L P A S Y K-COOH
Int
100
75
50
25
0
iY
y5
0
100
200
300
400
500
600
700
800
900
1,000
1,100
1,200
1,300
1,400
m/z

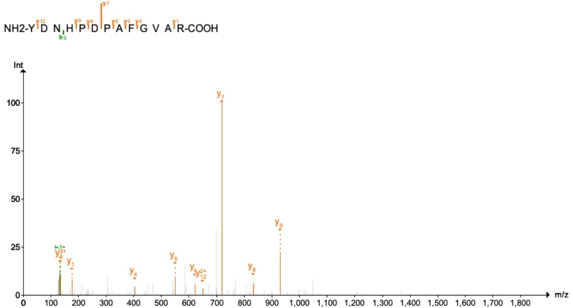

NH2-YDNHPDPAFGVAR-COOH
Int
m/z
y7
y9
y8
y5
y4
y1
y12 2+
b3 3+

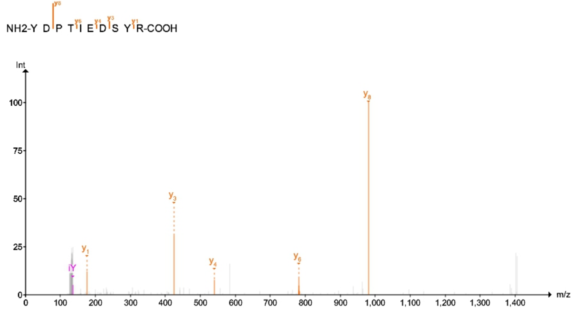
NH2-Y D P T I E D S Y R-COOH
y8
y6
y4
y3
y1
Int
100
75
50
25
0
y8
y3
y1
iY
y4
y6
0
100
200
300
400
500
600
700
800
900
1,000
1,100
1,200
1,300
1,400
m/z

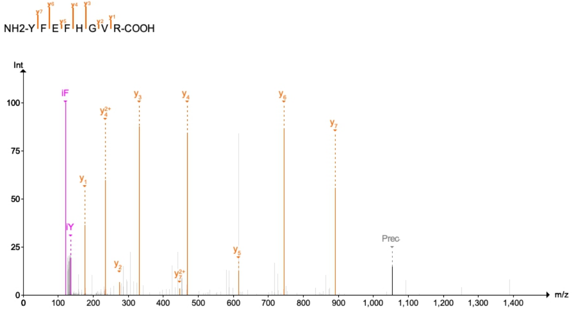

NH2-Y F E F H G V R-COOH
y7 y6 y5 y4 y3 y2 y1
Int
iF
iY
y1
y2
y3
y4
y4 2+
y5
y6
y7
y7 2+
Prec
100
75
50
25
0
0 100 200 300 400 500 600 700 800 900 1,000 1,100 1,200 1,300 1,400
m/z

NH2-Y F F A V D T A Y V A K-COOH

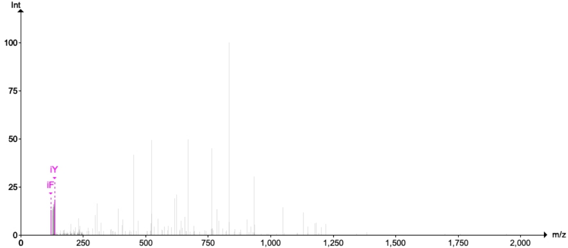

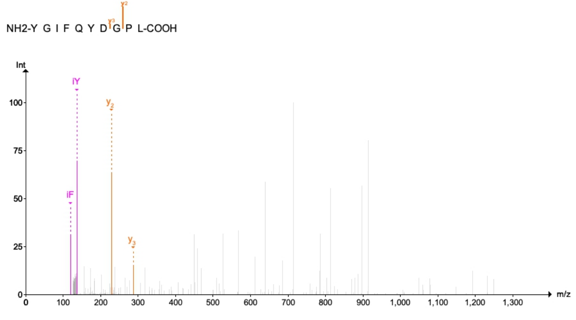

NH2-Y G I F Q Y D G P L-COOH
y2
y3
Int
iY
iF
y2
y3
100
75
50
25
0
0
100
200
300
400
500
600
700
800
900
1,000
1,100
1,200
1,300
m/z

NH2-Y G I P Y F E T S A A T G Q N V E K-COOH

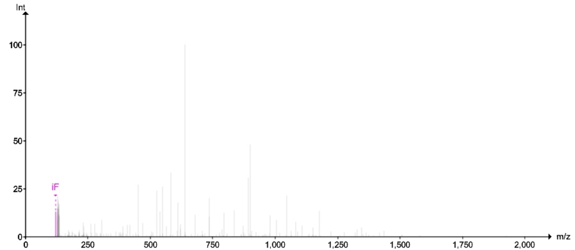

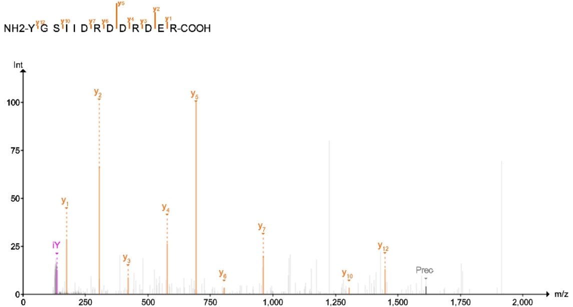
NH2-Y G S I I D R D D R D E R-COOH
y12 y10 y7 y6 y5 y4 y3 y2 y1
Int
m/z
y1 y2 y3 y4 y5 y6 y7 y10 y12
iY
Prec
0
25
50
75
100
0
250
500
750
1,000
1,250
1,500
1,750
2,000

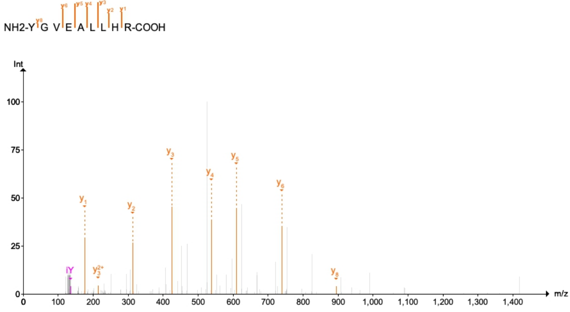
NH2-Y G V E A L L H R-COOH
y8 y6 y5 y4 y3 y2 y1
Int
m/z
y1
y2
y3
y4
y5
y6
y8
iY
y3 2+
0
25
50
75
100
0
100
200
300
400
500
600
700
800
900
1,000
1,100
1,200
1,300
1,400

NH2-Y H P D K N P D N P E A A D K-COOH

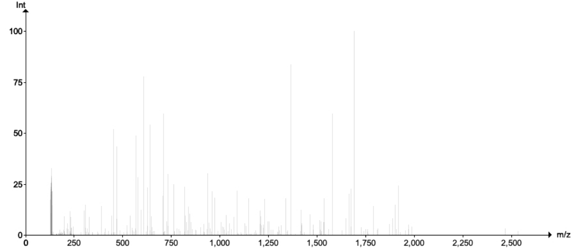

NH2-Y H Q Y I P P D Q K-COOH

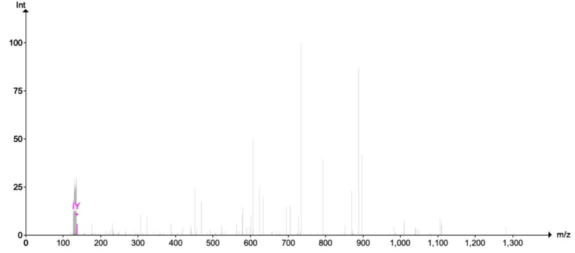

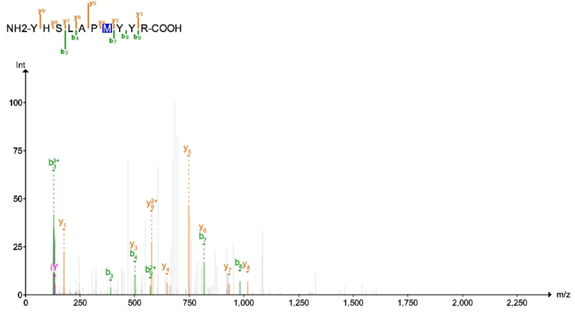
NH2-Y H S L A P M Y Y R-COOH
Int
m/z
y5
b3 3+
y1
y9 2+
y3
b4
y6
b7
b3
b9 2+
y4
y7
b8
y8
iY

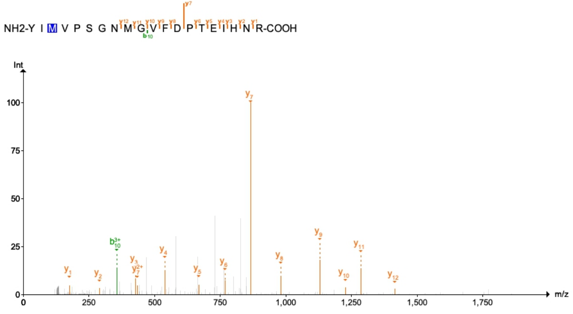
NH2-Y I M V P S G N M G V F D P T E I H N R-COOH
y12 y11 y10 y9 y8 y7 y6 y5 y4 y3 y2 y1
b10
Int
100
75
50
25
0
y7
b10 3+
y1
y2
y3
y7 2+
y4
y5
y6
y8
y9
y10
y11
y12
0
250
500
750
1,000
1,250
1,500
1,750
m/z

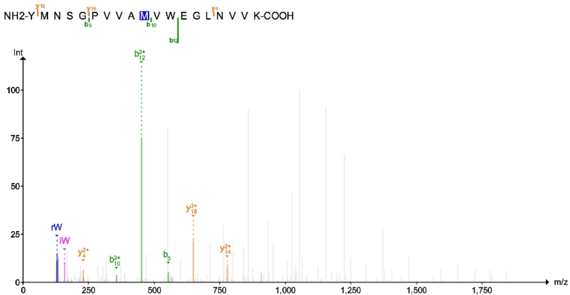

NH2-Y M N S G P V V A M V W E G L N V V K-COOH
y18 y14 y4
b5 b10 b12
Int
m/z
rW
iW
y_4^{2+}
b_{10}^{3+}
b_{12}^{3+}
b_5
y_{18}^{3+}
y_{14}^{2+}
0
25
50
75
100
250
500
750
1,000
1,250
1,500
1,750

NH2-Y P A L I G N L V K-COOH

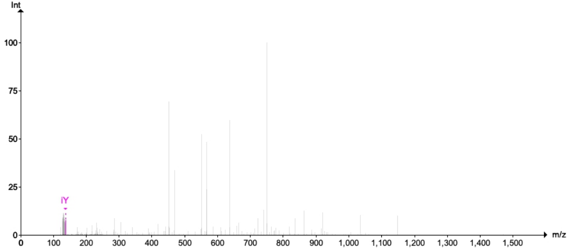

NH2-Y P D L Y P Q E D E D E E E E R E K-COOH

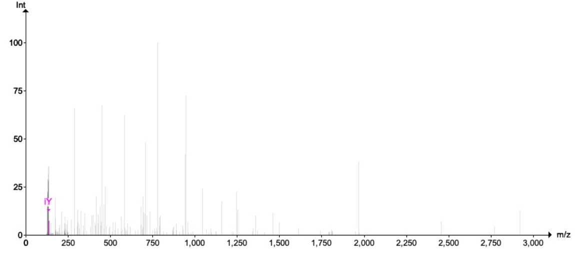

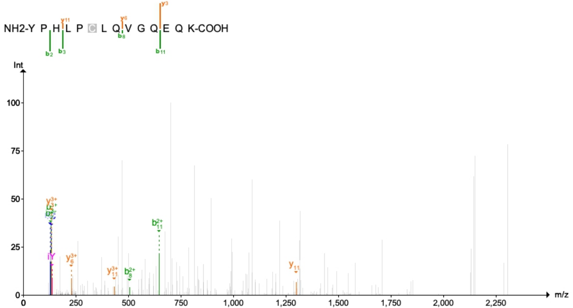

NH2-Y P H L P C L Q V G Q E Q K-COOH
Int
m/z
y3+
b2+
iY
y6 3+
y11 3+
b8 2+
b11 2+
y11

NH2-Y Q V E Y D A Y K-COOH

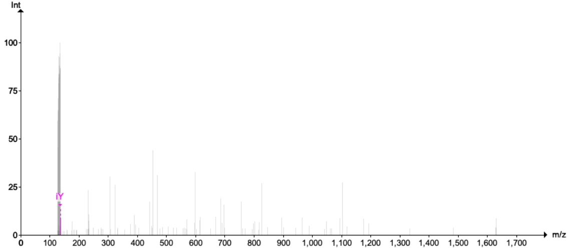

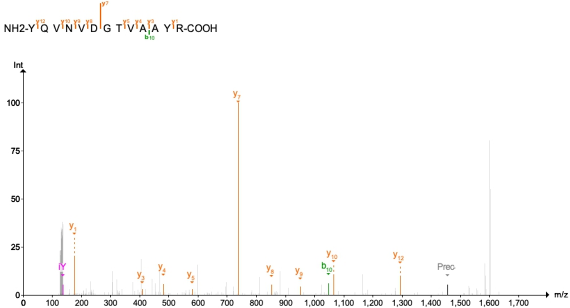
NH2-Y Q V N V D G T V A A Y R-COOH
y12 y10 y9 y8 y7 y5 y4 y3 y1
b10
Int
m/z
y1
y3
y4
y5
y7
y8
y9
y10
y12
b10
iY
Prec
0
25
50
75
100
100
200
300
400
500
600
700
800
900
1,000
1,100
1,200
1,300
1,400
1,500
1,600
1,700

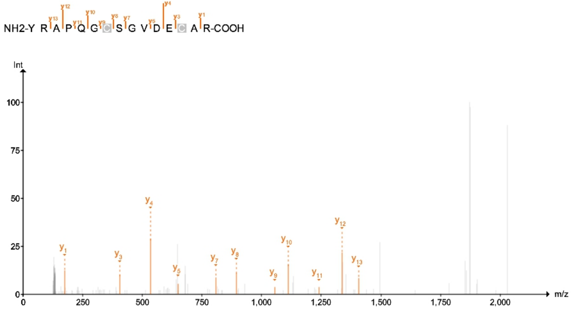

NH2-Y R A P Q G C S G V D E C A R-COOH
y13 y12 y11 y10 y9 y8 y7 y5 y4 y3 y1
Int
100
75
50
25
0
y1 y3 y4 y5 y7 y8 y9 y10 y11 y12 y13
0 250 500 750 1,000 1,250 1,500 1,750 2,000
m/z

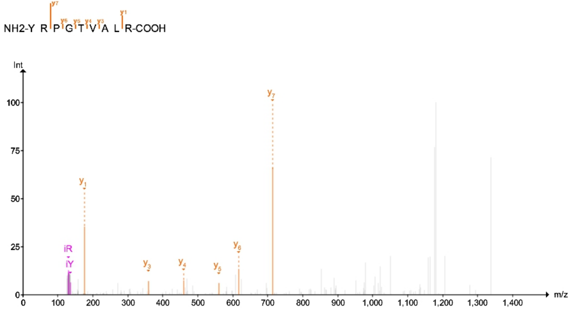
NH2-Y R P G T V A L R-COOH
y7
y6
y5
y4
y3
y1
Int
100
75
50
25
0
y7
y1
y3
y4
y5
y6
iR
iY
0
100
200
300
400
500
600
700
800
900
1,000
1,100
1,200
1,300
1,400
m/z

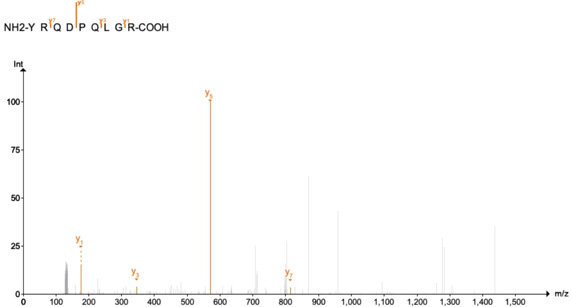
NH2-Y R Q D P Q L G R-COOH
y7
y5
y3
y1
Int
100
75
50
25
0
y5
y1
y3
y7
0
100
200
300
400
500
600
700
800
900
1,000
1,100
1,200
1,300
1,400
1,500
m/z

NH2-Y S Q I C A K-COOH

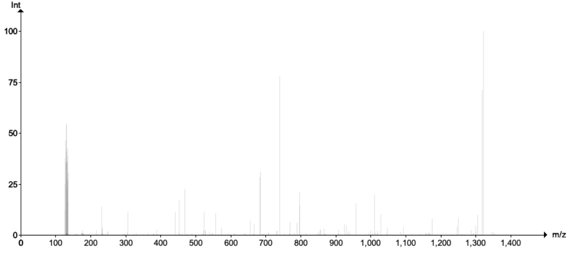

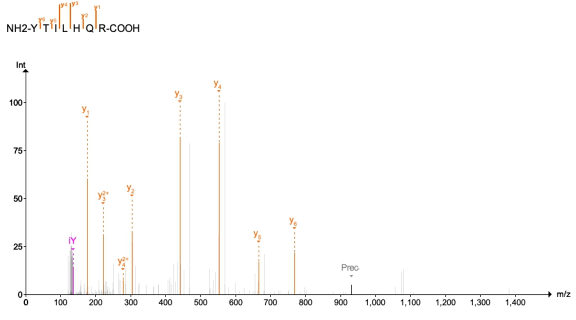

NH2-Y T I L H Q R-COOH
y6 y5 y4 y3 y2 y1
Int
100
75
50
25
0
y1
y2
y3
y4
y5
y6
y3 2+
y4 2+
iY
Prec
m/z
0 100 200 300 400 500 600 700 800 900 1,000 1,100 1,200 1,300 1,400

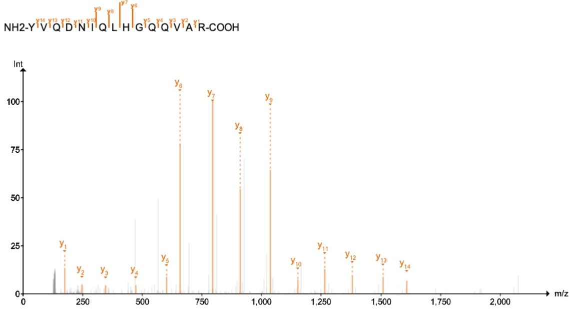
NH2-Y V Q D N I Q L H G Q Q V A R-COOH
Int
100
75
50
25
0
m/z
0
250
500
750
1,000
1,250
1,500
1,750
2,000
y1
y2
y3
y4
y5
y6
y7
y8
y9
y10
y11
y12
y13
y14
